# Genetic diversity of *Dioctophyme renale* in Southern South America

**DOI:** 10.1017/S0031182024001379

**Published:** 2025-07

**Authors:** Lucas F. Arce, Florencia Facelli Fernández, Nahili Giorello, Marcos Butti, Lucas L. Maldonado, Juan P. Arrabal, María B. Natalini, Martín Kowalewski, Daniela Pedrassani, Carolina Silveira Mascarenhas, Josaine C. da Silva Rappeti, Florencia Zilli, Pablo M. Beldomenico, Verónica Lia, Gisela R. Franchini, Laura Kamenetzky

**Affiliations:** 1 Laboratorio de Genomica y Bioinformatica de Patogenos, Departamento de Fisiologia y Biologia Molecular y Celular, Facultad de Ciencias Exactas y Naturales, Instituto de Biociencias, Biotecnologia y Biologia Traslacional (iB3), Universidad de Buenos Aires (UBA), Buenos Aires, Argentina; 2 Laboratorio de Biofísica y Biología Celular de Proteínas que Unen Lípidos, Facultad de Ciencias Médicas, Instituto de Investigaciones Bioquímicas de La Plata ‘Profesor Doctor Rodolfo R. Brenner’ (INIBIOLP), Universidad Nacional de La Plata, La Plata, Argentina; 3 Instituto Nacional de Limnología (INALICONICET-UNL), Ciudad Universitaria, Santa Fe, Argentina; 4 Laboratorio de Parasitosis Humanas y Zoonosis Parasitarias, Facultad de Ciencias Veterinarias, Universidad Nacional de La Plata (UNLP) - Comisión de Investigaciones Científicas (CIC), La Plata, Argentina; 5 Instituto de Biología Subtropical IBS-CONICET, Universidad Nacional de Misiones-UNAM, Puerto Iguazú, Misiones, Argentina; 6 Estación Biológica Corrientes, CECOAL-CONICET, San Cayetano, Corrientes, Argentina; 7 Universidade do Contestado - Campus Canoinhas, Canoinhas, SC, Brazil; 8 Instituto Federal Sul-rio-grandense (IFSul), Campus Pelotas, RS, Brazil; 9 Universidad Federal de Pelotas, UFPEL PELOTAS-RS, Brazil; 10 Laboratorio de Ecología de Enfermedades, Instituto de Ciencias Veterinarias del Litoral (ICIVET-CONICET-UNL), R.P Kreder Esperanza, Santa Fe, Argentina; 11 Facultad de Ciencias Exactas y Naturales Universidad de Buenos Aires, Ciudad Autónoma de Buenos Aires, Argentina

**Keywords:** COX1, *Dioctophyme*, kidney parasite nematode, mitochondrial, ND4, phylogeography, wild carnivores

## Abstract

*Dioctophyme renale*, the giant kidney worm, is a nematode related to *Trichuris* sp and is distributed worldwide. These parasites locate in the kidney of their definitive hosts (mainly belonging to the order Carnivora) and have an indirect life cycle with an annelid as the main intermediate host. Humans are rarely affected, but in those that are, 1 or both kidneys are destroyed. In South America, *D. renale* is widespread in riparian regions where changing climatic conditions, environmental degradation, and compromised sanitation are increasing the risk of distribution of this parasite, including humans. Here, we provide the descriptions of the genetic diversity of the parasite in the region by analysing 73 adult *D. renale* samples collected from domestic and wild carnivores. The most common hosts were (*Canis lupus familiaris)* and maned wolf (*Chrysocyon brachyurus* Fam. Canidae) among domestic and wild carnivores, respectively. This work shows the descriptions of the genetic diversity of this parasite complementing molecular methods and classical and probabilistic phylogeography. Our results strongly suggest that this parasite has been present on the continent long enough to develop local genetic variants. Also, the phylogenies show transmission between localities and bidirectional transmission between domestic and wild species. We now have new tools to understand the ecological dynamics of this parasite such as molecular markers to study its genetic diversity as well as for identification and reporting in cryptic cases.

## Graphical abstract

**Figure:**
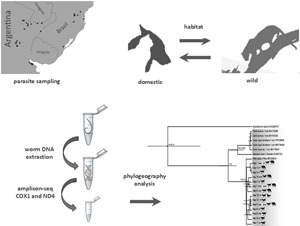

## Introduction

*Dioctophyme renale* (Goeze, 1782) is a zoonotic parasitic nematode that infects the kidneys of mammals, mainly carnivores. This parasite has a worldwide distribution and has been observed in various species, both domestic and wild. In dogs, the infection usually involves the right kidney often causing unilateral loss of the organ, and in some cases bilateral involvement can result in total renal failure. In addition, blockage of the ureters or renal pelvis by adult worms may result in hydronephrosis. Adult worms can also be found in the abdominal cavity, subcutaneous tissue and other organs such as the uterus and ovary (Khullar *et al*., 2022). Humans may be incidental hosts and develop unspecific clinical symptoms including back pain, fever, weight loss, urinary retention, hematuria and pyuria. Fatalities are rare but have been reported in extreme cases due to renal failure, sepsis, or coexisting medical conditions (Li *et al*., 2010; Norouzi *et al*., 2017; Khullar *et al*., 2022). The life cycle described by Mace and Anderson (1975) involves an oligochaete (*Lumbriculus variegatus*) in which *D. renale* develops the infective stage. This invertebrate presents a Holarctic distribution, with only two reports in South America: one in Argentine Patagonia (Miserendino, 2007) and another in Minas Gerais, Brazil (Marchese *et al*., 2015), and no evidence of the presence of larval stages of *D. renale*. Therefore, the life cycle outside the definitive host is unknown for this region.

Different biogeographical units converge in the region spanning northeastern Argentina and southern Brazil, specifically the sub-Brazilian domain, Paranaense and Chaco domains. These domains, which encompass more than 16 biogeographical districts (Arana *et al*., 2021) host a variety of ecosystems including swamps and marshes, fields and scrubland, pampas, gallery forests, espinal, Chaco forest and Parana jungle (Pereyra, 2003). Cities, rural villages and protected natural areas, all communities with different levels of anthropic influence coexist in this area and animals affected by dioctophymosis can be observed in all these systems. With regard to urban areas the most common domestic host is the dog with prevalences ranging between 0.03 and 35.3% (Radman *et al*., 2017). In the La Plata River riparian area, a prevalence of 42.1% is reported (Burgos *et al*., 2015). In addition, there are also reports of *D. renale* in cats (Pedrassani *et al*., 2014; Butti *et al*., 2019). Regarding wild ecosystems, we recently reported the first molecular characterization of *D. renale* in the pampas fox (*Lycalopex gymnocercus*) (Fernández *et al*., 2024). Also, there are reports of cases in other wild mammals including maned wolf (*Chrysocyon brachyurus*), Geoffroy's cat (*Leopardus geoffroyi*), Neotropical otter (*Lontra longicaudis*); bush dog (*Speothos venaticus*), crab-eating fox (*Cerdocyon thous*), little grison (*Galictis cuja*), coati (*Nasua nasua*), capuchin monkey (*Cebus apella*), two-toed sloth (*Choloepus hoffmanni*), among others (Measures, 2008; Pinto *et al*., 2011; Echenique et al., 2018; Trindade *et al*., 2018; Khullar *et al*., 2022). High prevalence of this parasitosis was observed in the species maned wolf, little grison and coati, with levels of 81.2, 36.6 and 72.4%, respectively (Eiras *et al*., 2021). In maned wolf, as in dogs, most parasites have been found in the right kidney in (85.7% of cases), followed by the abdominal cavity (28.6%). On the other hand, in coatis the most common site where the worms were found is the abdominal cavity, accounting for 66.7% of the cases (Milanelo *et al*., 2009). In general, there are no clinical signs associated with this infection (Mattos Varzone *et al*., 2008; Di Nucci *et al*., 2020). However, the actual impact of the parasite on natural populations remains poorly understood. Currently, there is no information on whether domestic and wild mammals in this region are infected by the same phylogentic lineages or whether there are host- and/or site-specific genetic variants. In addition, there is very little sequence information about this parasite in databases. *D. renale* belongs to Clade I in accordance with the classification of the Phylum Nematoda proposed by Blaxter and Koutsovoulos (2015), a group which is underrepresented in terms of molecular information. Clade I genome information is only available for a few species of the genera *Trichinella*, *Trichuris*, *Romanomermis* and *Sobolophyme* (Wormbase Parasite), with no genomic data for *D. renale*. In GenBank (https://www.ncbi.nlm.nih.gov/) there are only 27 entries for *D. renale* in the nucleotide database corresponding to 3 genes: small subunit ribosomal RNA, Dorylipophorin (a novel lipid binding protein) and mitochondrial cytochrome c oxidase subunit I (COX1). The limited molecular knowledge about this parasite hampers its identification, genetic characterization, outbreak monitoring and studies of host species interactions. In this work, we utilize genomic data to develop new molecular markers for investigating the population dynamics of *D. renale*, a prevalent zoonotic parasite, whose life cycle remains unknown in southern South America.

## Materials and methods

### Biological sample

**Figure 1.**
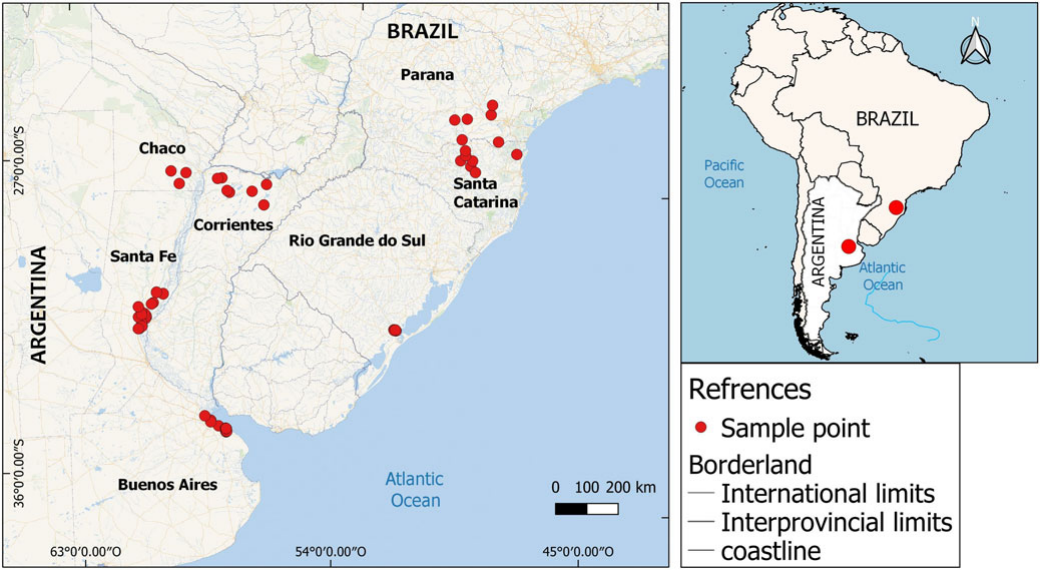
Geographic distribution of *Dioctophyme renale* samples Maps were plotted with Qgis version 3.16.3, OSGeo, layer (CRS) EPSG:4326 – WGS 84. Sample points (red circles).

Adults of *D. renale* from 73 different hosts were sampled. One parasite by each host was selected for genetic analysis. Fifty-seven of them were collected from domestic mammal surgeries at veterinary institutions. Also, 16 samples were from necropsies of road-killed wild fauna. Surgeries and animal necropsies were carried out under approved protocols by the corresponding authority (National Parks Administration technical office NEA 423 Rnv ex DCM 483 Dispo 23/2015; Ministry of Environment and Climate Change, Province of Santa Fe, Exp 02101-022465-3, Res No 025). The localities from which samples were received include Buenos Aires, Santa Fe, Chaco, and Corrientes provinces in Argentina and the states of Parana, Rio Grande do Sul and Santa Catarina in Brazil. Parasites were found in different host species including domestic dog (*Canis lupus familiaris*) and cat (*Felis catus*) and wild mammals, such as the lesser grison (*G. cuja*), the maned wolf (*C. brachyurus*) and the pampas fox (*L. gymnocercus*) (Table 1). Sampling locations are shown in Fig. 1.

**Table 1.** Total *Dioctophyme renale* samples analysed by molecular markers

Host	Province/State	Country	Samples received	COX1 short	COX1-XL	ND4
*Canis lupus familiaris*	Buenos Aires	Argentina	28	19	17	12
*Canis lupus familiaris*	Santa Fe	Argentina	11	10	10	10
*Canis lupus familiaris*	Chaco	Argentina	1	1	1	1
*Canis lupus familiaris*	Rio Grande do Sul	Brazil	6	5	5	5
*Canis lupus familiaris*	Santa Catarina	Brazil	4	2	2	1
*Canis lupus familiaris*	Parana	Brazil	4	2	2	1
*Felis silvestris catus*	Parana	Brazil	1	1	0	0
*Felis silvestris catus*	Santa Catarina	Brazil	2	1	0	0
**Total Domestic**	**–**	**–**	**57**	**41**	**37**	**30**
*Chrysocyon brachyurus*	Corrientes	Argentina	7	6	4	4
*Chrysocyon brachyurus*	Santa Fe	Argentina	4	4	4	4
*Chrysocyon brachyurus*	Chaco	Argentina	2	2	2	2
*Lycalopex gymnocercus*	Santa Fe	Argentina	1	1^a^	1	1
*Galictis cuja*	Santa Catarina	Brazil	2	1	0	0
**Total Wild**	**–**	**–**	**16**	**14**	**11**	**11**
**TOTAL**	**–**	**–**	**73**	**55**	**48**	**41**

Number of samples received and sequenced grouped by host and location.

a Sequence from Fernández *et al*. (2024).

### DNA extraction and genotyping

**Figure 2.**
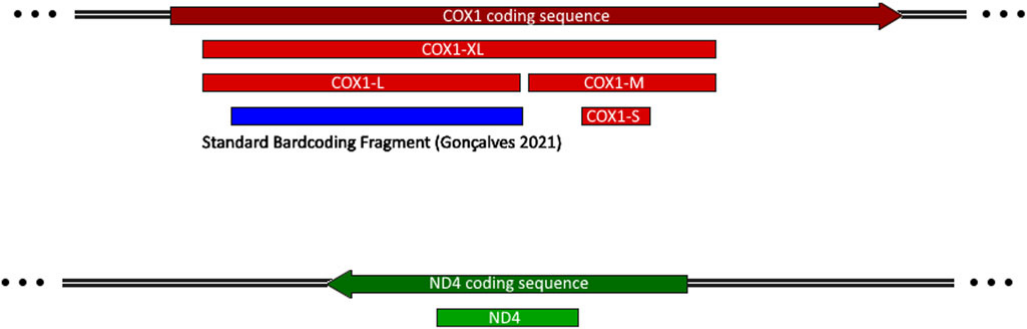
Genomic map showing the markers designed for this study. Arrows indicate the coding sequences and bars correspond to the expected PCR products (below) using SnapGene v1.1 (Glick *et al*., 2004). (A) COX1 gene. (B) ND4 gene.

For each parasite sample a transversal section was cut and 20 mg without cuticle were lysed using liquid nitrogen. Total genomic DNA was extracted using DNeasy® Blood & Tissue QIAGEN Kit. Two mitochondrial markers were selected from the draft assembly of *D. renale* mitogenome (Macchiaroli *et al*, in preparation) (Fig. 2). One of them was the barcode region of the cytochrome c oxidase subunit I gene (COX1), relevant in nematode taxonomy (Gonçalves *et al*., 2021). Primer pairs were designed encompassing 4 regions of COX1. Two of them (116 and 382 bp) correspond to those previously described in Koehler *et al*. (2009*a*) and Tokiwa *et al*. (2014), respectively. The third primer pair covered a 687 bp COX1 region not previously used in this species, and finally the fourth primer pair spanning all 3 regions (1133 bp). In order to select a second molecular marker, the draft mitogenome of *D. renale* was compared to the mitochondrial genomes of several *Trichuris* spp. (Genbank numbers: AP017703.1, AP017704.1, GU070737.1, GU385218.1, KT449822.1, KT449823.1, KT449824.1, KT449825.1, KT449826.1, LC050561.1, NC_017747.1, NC_017750.1, NC_018596.1, NC_018597.1, NC_028621.1) using multiple sequence alignment. The nicotinamide adenine dinucleotide dehydrogenase subunit 4 gene (ND4) was chosen as the second molecular marker because, when comparing these mitogenomes, it showed a greater mean distance from Tamura-Nei (0.53) than COX1 (0.30), which could result in a higher resolution in the phylogenies. ND4 also shows on average a higher percentage of variant sites (76%) than COX1 (39%) when comparing *Trichuris spp*. and *D. renale*. In addition, this gene is one of the most employed molecular markers in population genetics studies of parasitic nematodes (Blouin *et al*., 1998; Koehler *et al*., 2009*b*; Gharamah *et al*., 2012; Shen *et al*., 2017; Nguyen *et al*., 2019). ND4 primers were designed to encompass a 354 bp target region within the gene coding sequence and tested in-silico for specificity using the NCBI database, WormBase and *D. renale* genome scaffolds. COX1 and ND4 amplifications were performed in a final 50 μL volume per tube containing sample DNA (20–500 ng), 0.22 mm each dNTP (Pharmacia LKB, Uppsala, Sweden), 1.66 mm MgCl_2_, 0.55 mm of each primer (Table 2) and 0.04 U μL^−1^ of Taq Pegasus DNA polymerase in reaction buffer (Productos Bio-Lógicos, Argentina). The PCR conditions were as follows: an initial denaturation step (95°C for 5 min) followed by 35 cycles of 95°C for 60 s (denaturation), 50°C for 60 s (annealing), 72°C for 60 s (extension), and a final extension step (72°C for 10 min). The PCR products were quantified in 1% agarose gel using nucleic acid dye GelRed® and were revealed in an UV transilluminator. Amplicons with the expected size and concentration were Sanger sequenced for forward and reverse primers using the service provided by Macrogen (South Korea).

**Table 2.** Mitochondrial molecular marker amplification

Gene	Primer name	Primer sequence 5′–3′	Expected product size (pb)	Source
ND4	DRND4F	AGAAGAGGATCATATCTTAT	354	This work
	DRND4R	GCTACGAATTTTTTAATGTCG		
COX1-S	DRCOX1-SF	TGGTGTGCTTGGTTGTTTTG	116	Modified from Tokiwa *et al*. (2014)
	DRCOX1-SR	AACCTGCCCACCATACAAAG		
COX1-M	DRCOX1-MF	CATCCWGAGGTTTATATTYTAGC	382	Modified from Koehler *et al*. (2009*b*)
	DRCOX1-MR	ASWAAGAACAWARTGRAAATGACC		
COX1-L	DRCOX1-LF	CTTCAGTTATTGGTGGGTGT	687	This work
	DRCOX1-LR	GTTGGAATAGAACAGGGTCA		
COX1-XL	DRCOX1-LF	CTTCAGTTATTGGTGGGTGT	1133	This work and modified from Koehler *et al*. (2009*b*)
	DRCOX1-MR	ASWAAGAACAWARTGRAAATGACC		

Gene, primer name, sequence and expected PCR products size are shown.

### Sequence analysis

BioEdit software (Hall, 1999) was employed to perform sequence analysis. Primers and low-quality regions at the beginning and at the end of each sequence were removed. A consensus of forward and reverse reads per amplicon was made by using CAP Contig Assembly algorithm (Huang, 1992). All variable sites were confirmed by forward and reverse sequences. Consensus sequences are available in GenBank under accession numbers OP208282.1 to OP208330.1, and OP204915.1 to OP204944.1. Multiple sequence alignment was performed using ClustalW (Thompson *et al*., 1994) (Supplementary File). From each marker, variability measures were estimated including number of haplotypes, haplotype diversity (Hd) (Nei and Tajima, 1981), number of segregating sites, number of mutations, polymorphic sites proportion, nucleotide diversity through *θ_ℼ_* (Nei and Li, 1979) and *θ*_W_ (Watterson, 1975). To test for deviations from the neutral model and constant population size, Tajima's *D*’ test (1989) and Fu Fs's (1997) were used for both COX1 and ND4. In addition, Ramos y Onzis *R*^2^'s test (2002) was also performed to evaluate population growth. In all cases significance was determined through 1000 coalescent simulations (Hudson, 1990). All these analyses were made by using DnaSP v6 (Rozas *et al*., 2017).

To assess the factors influencing the distribution of genetic variation, we performed an analysis of molecular variance (AMOVA, Excoffier *et al*., 1992) using sequences of adults of *D. renale* from wild *vs* domesticated host species and geographical regions (province/state) as clustering criteria in the knowledge that the ecosystem between these localities is not contiguous and that Santa Catarina and Parana were excluded from the analysis because they present only one sequence each. Genetic distances matrix for the AMOVA was computed under the Tamura-Nei model (1993) and 10 000 permutations were performed to estimate significance using Arlequin v3.5 (Excoffier *et al*., 2005). With the objective of testing for isolation by distance a Mantel Test (Mantel, 1967) comparing between geographical distance matrix (obtained by the software QGIS v3.22, OSGeo) and genetic distances matrix (obtained by software MEGA) was performed using the software R 4.2.1 software with the graphical interface Rstudio v2022 (Allaire, 2012) as described in Banta *et al*. (2007).To investigate relationships among haplotypes, a phylogenetic tree was constructed by using Maximum Likelihood (ML) and Bayesian inference (BI) methods as implemented in MEGA v11 (Tamura *et al*., 2021) and BEAST v2 (Bouckaert *et al*., 2019), respectively. First, the partition scheme and the evolutionary model that best fit our data were selected with Partition Finder 2 (Lanfear *et al*., 2017). The models employed were Tamura & Nei model (1993) for COX1 and General Time Reversible model (Tavare, 1984) for ND4. Multiple alignment of COX1 includes all the homologous sequences from *D. renale* available in GenBank (Accession numbers: AB854727.1, EU394733.1, MH178399.1, MH178400.1, MH178401.1, MH181826.1 and MT246537.1). The substitution rate of 0.0259 × 10^−6^ per site per year previously reported by Zarlenga *et al*. (2006) for the genus *Trichinella*, was used to estimate divergence times. For ND4, only sequences from this work were used because there are no other sequences available in Genbank. One thousand bootstrap replicates were performed to assess branch support for the ML tree. The Markov Chain Monte Carlo (MCMC) run for the BI phylogeny consisted of one chain with 10 million generations and sampled every 10 000 generations. Program Tracer v1.7 (Rambaut and Drummond, 2003) was employed to verify that the effective sample size (ESS) of every parameter was over 200. Median Joining haplotype networks (Bandelt *et al*., 1999) from individual and concatenated markers were constructed using PopArt Software (http://popart.otago.ac.nz). The concatenated data resulted in a matrix of 1198 characters and 41 taxa.

## Results

### Domestic and wild carnivores from Argentina and Brazil are definitive host of *D. renale*

A total of 73 adult *D. renale* samples were collected from domestic and wild carnivores from different regions of Argentina and Brazil (Table 1). The most common host was the domestic dog (*C. l. familiaris*), from which 54 adult parasites were collected. Three additional samples were isolated from cats (*F. catus*), totaling 57 adult parasites from domestic host species. Among wild carnivores, the maned wolf (*C. brachyurus* Fam. Canidae*)* was the most frequent host, with 13 samples isolated. Also, adult parasites were collected from lesser grison (*G. cuja*, Fam. Mustelidae) and pampas fox (*L. gymnocercus*, *Fam. Canidae*), totalling 16 adult parasites from wild species (Table 1). In Argentina, we collected samples from 4 Provinces where domestic and wild hosts of *D. renale* are present. In Brazil, we collected parasite samples from domestic animals from 3 different States but parasites from wild hosts were only collected in Santa Catarina (Fig. 1). A total of 73 adults of *D. renale* were collected, of which 44% were males and 56% females. Similar proportions were observed when analysing *C.l. familiaris* and *C. brachyurus* separately. The main anatomical site affected was the right kidney (58% of the cases) followed by the abdominal cavity (39%).

### COX1 is a useful molecular marker for *D. renale* population genetics analysis

Four set of primers were applied in this work, named COX1-S, COX1-M, COX1-L and COX1-XL (Table 2). It is important to note that the region amplified by COX1-XL covers all the 3 smaller regions, therefore unless otherwise specified, the region used in all analyses is COX1-XL. A total of 55 samples were amplified with COX1-S set of primers and 48 with COX1-XL (Table 1). Notably, the 10 samples that could only be amplified with COX1-S primers allowed us to obtain sequences from new domestic (*F. catus*) and wild (*G. cuja*) hosts. By performing a BLAST search against the NCBI nucleotide database, these sequences showed high similarity with homologous sequences from other *D. renale* parasites, with identities ranging from 91.8 to 100% (accession number AB854727.1 and MN304733.1, respectively). On the other hand, a remarkable difference is observed with respect to the homologous sequences of related species such as *Sobolophyme baturini* and *Trichuris muris*, with identities ranging from 81.9 to 84.9% (accession number MZ675607.1 and EU394157.1, respectively) (Supplementary Files). Six genetic diversity metrics were determined for each mitochondrial marker analysed (Table 3). Even though the number of mutations and segregating sizes increases through different COX1 marker lengths, the proportion of polymorphic sites is higher in the region of COX1-M as expected due to being located in the variable region of the gene. Similarly, it has the greatest nucleotide diversity (*θ_ℼ_* and *θ*_w_). The COX1-XL comprises all the variable sites contained within the shorter COX1 markers, as a consequence it has the greatest haplotype richness and allows the best resolution of phylogenetic relationships. This marker will be referred to as COX1 in the following sections.

**Table 3.** Overall diversity measures across different mitochondrial markers employed^a^

Marker	Sample size	Sequence length (pb)	Number of Haplotypes	Eta	S	Ro	Hd	*θ_π_*	*θ_w_*
COX1-S	55	74	4	2	2	0.0270	0.488	0.00732	0.00591
COX1-M	52	259	14	13	12	0.0463	0.869	0.00992	0.01029
COX1-M^b^	41	259	12	13	12	0.0463	0.889	0.01081	0.01087
COX1-L	49	586	16	21	20	0.0341	0.878	0.00598	0.00765
COX1-XL	48	987	20	34	32	0.0324	0.909	0.00731	0.00854
ND4	41	354	13	23	23	0.0650	0.881	0.01048	0.01519
Concatenated (COX1 + ND4)	41	1343	23	56	54	0.0402	0.945	0.00850	0.01055

a Headers references: Eta, Number of mutations; S, Number of segregating sites; Ro, Proportion of polymorphic sites; Hd, Haplotype diversity; *θ_π_*, nucleotide diversity (Nei, 1987); *θ*_w_, nucleotide diversity (Watterson, 1975).

b Same sample set that has ND4 sequence.

### ND4 a new mitochondrial marker for *D. renale*

The ND4 gene region was selected as a result of the in-silico analysis of mitogenomic DNA variation in *Trichuris* species, a genus that shares Clade I with *D. renale* (Blaxter and Koutsovoulos, 2015). Greater variation in the proportion of polymorphic sites and mean genetic distance within a section of the ND4 gene, compared to other mitochondrial genes, was observed in these genera (Supplementary Files). Therefore, the ND4 marker could provide a better comprehension of intraspecific differences. As expected, ND4 showed the highest proportion of polymorphic sites (0.0650). Consistent with expectations under mutation-drift equilibrium, COX1-XL shows negligible differences between *θ_π_* and *θ_w_* (0.00731 and 0.00854 respectively). Conversely, ND4 shows higher values of *θ*_w_ in comparison with *θ_π_* (0.01519 and 0.01048 respectively), which is consistent with a process of population growth or purifying selection (Table 3).

The results obtained from the AMOVA revealed that no percentage of genetic diversity was explained by the differentiation between wild and domestic hosts, indicating a common source of infection between them. When individuals were categorized by locality (province or state), differentiation among groups accounted for approximately 4.4% of total variation. This percentage increased to 5.6% when combining Corrientes and Chaco provinces, where mammals share the same water resources from Paraná River (Supplementary file S6).

Concatenating COX1 and ND4 sequence information, Santa Fe had the highest number of haplotypes (*N* = 15) followed by Buenos Aires (*N* = 12). Buenos Aires showed higher nucleotide diversity than Santa Fe (*θ_π_* = 0.00856 and 0.00798 respectively) and the same is observed with haplotype diversity (Hd = 0.985 and 0.848 respectively). Only 2 haplotypes (H13 and H14) are shared between these 2 localities. This could point to the population of Buenos Aires as the oldest, but it would not be possible to establish any hypothesis as to how the dispersion between localities occurred and whether one population gave rise to the other. No differences of Hd were observed between countries nor domestic and wild host (Supplementary Files).

### Phylogenetic analysis

**Figure 3.**
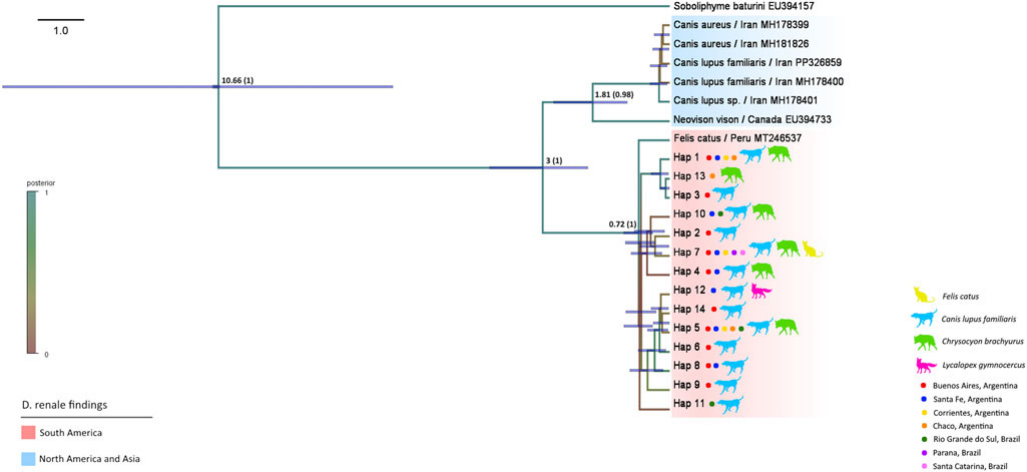
Bayesian inference tree obtained with BEAST v2.6.7 software and plotted with the iTOL online tool. The haplotypes found in our study for the COX1-M gene and those available in GenBank are shown. The GenBank sequence of *Sobolophyme baturini* was included as an outgroup. The nodes show the time in Mya and in brackets the posterior probability.

The COX1-M marker was selected to perform phylogenetic analysis, since this region presents the highest number of homologues in the GenBank database (Fig. 3). The phylogenetic tree topology shows that the sequences from Argentina and Brazil obtained in this work cluster with a sample from Perú (Genbank number MT246537.1) forming a South American clade. In other node are grouped those from Canada (Genbank number EU394733.1) and Iran (Genbank number MH178300.1, MH181826.1, PP326859.1, MH178400.1 and MH178401.1). The *Trichinella* substitution rate employed allows us to propose the first hypothesis of the divergence between these clades, which is estimated to have occurred approximately 3 million years ago. In this clade there is no differentiation by host species nor location. The same pattern is observed in the COX1-S phylogeny with the addition of the Japanese sequence reported by Tokiwa *et al*. (2014) (Supplementary File).

**Figure 4.**
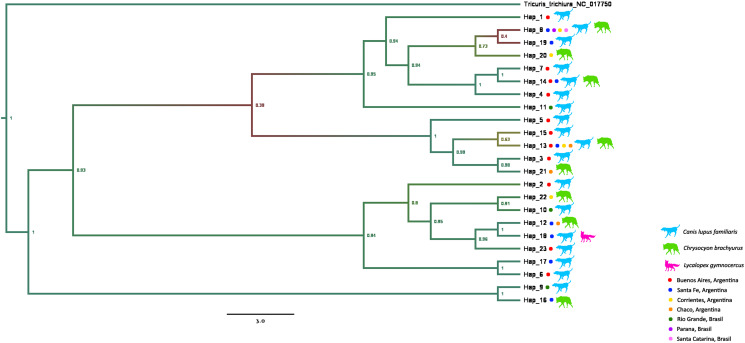
Proportional branch transformed phylogenetic tree from concatenated marker (COX1 + ND4) genes by using Bayesian Inference method. The GenBank sequence of *Trichuris trichiura* was included as an outgroup. Colour gradient is related to branch posterior probability and values are displayed at the side of nodes. At the tips is indicated localities and host species of samples included in every haplotype. Evolutionary analyses were conducted in BEASTv10.

The cladogram constructed using genetic information obtained from the concatenated matrix (COX1 + ND4) shows 21 haplotypes (Fig. 4). Four of them were observed both in wild and domestic hosts (Hap8, Hap13, Hap14 and Hap18). Hap13 and Hap8 showed the highest geographical dispersion (4 localities each) followed by Hap12 and Hap14 (2 localities each). In addition, every clade with high statistical support (posterior probability > 0.9) includes samples coming from different geographical regions and host species.

### Genetic and geographical relationships

**Figure 5.**
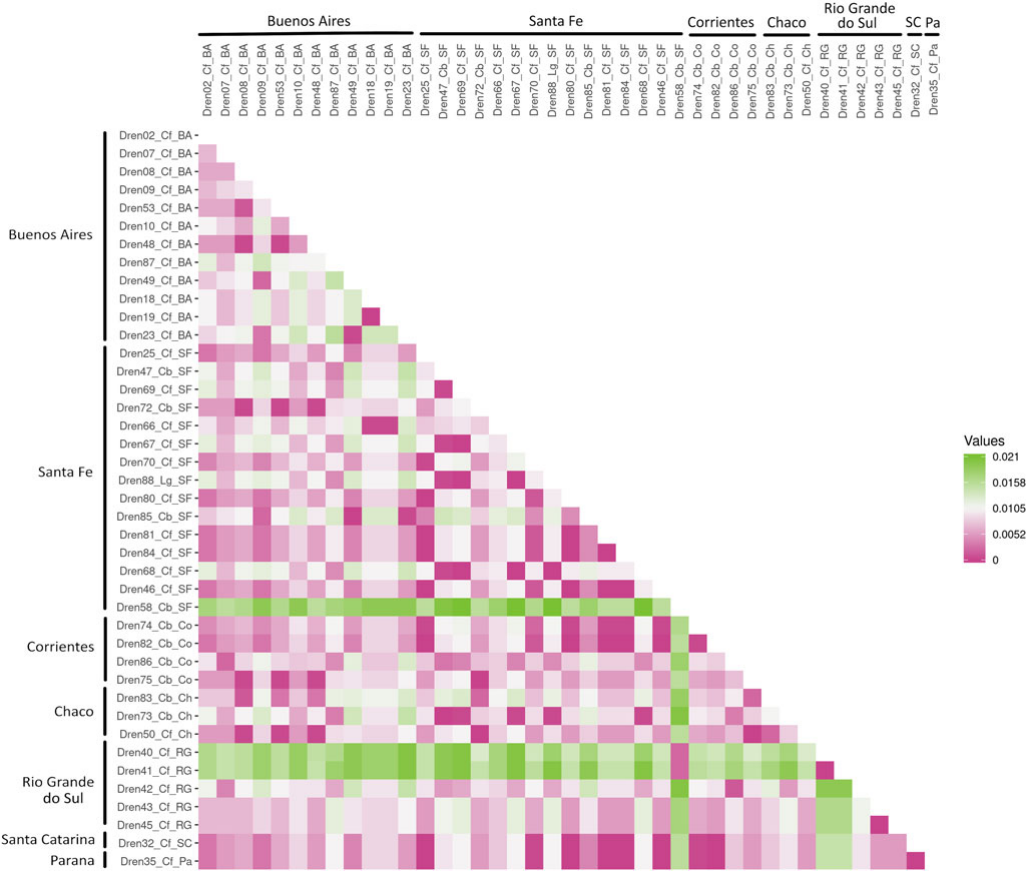
Isolation by distance analysis. Matrix among samples grouped by locality (distance: Tamura and Nei, 1993). The data was obtained by using MEGAv11 software.

The results of the Mantel test showed that there is no significant correlation between genetic and geographical distances, with 100 000 permutations, Mantel's *r* = 0.007, Pr(*r* = 0) = 0.148. This result showed that there was no evidence of isolation of genetic flow by distance in the region studied. A great variation of distances within locality is observed (Fig. 5).

**Figure 6.**
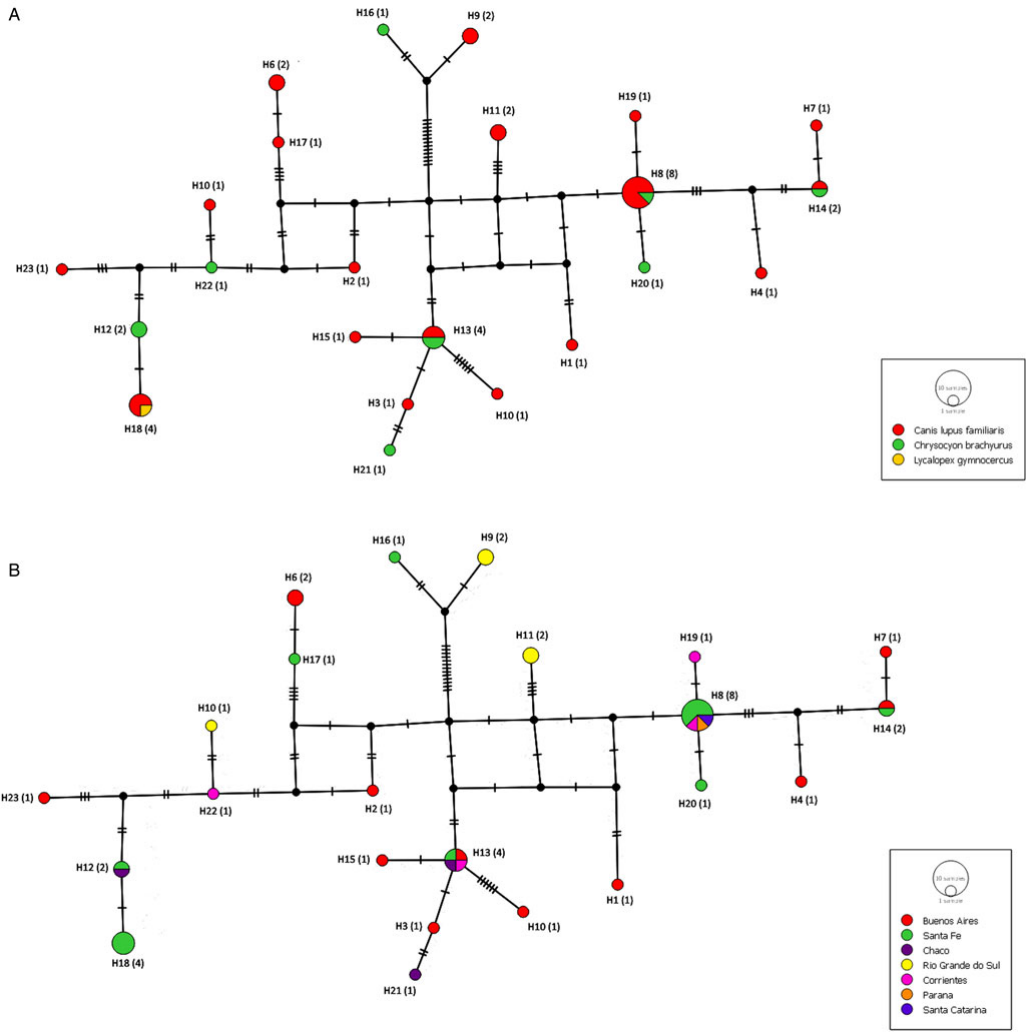
Haplotype network COX1-ND4 concatenating. A: Locations are indicated by different colours. B: Species are indicated by different colours. Networks build by Median Joining method (epsilon = 0). Every circle displays the haplotype marker ID and frequency (in brackets).

Three samples show high distance relative to the others: Dren40 and Dren41 coming from dogs located in Rio Grande do Sul and Dren58, from a maned wolf in Santa Fe. These samples are shown separated from the rest in haplotype networks, H9 (Dren40 y Dren41) and H16 (Dren58) (Fig. 6). These same haplotypes form the most ancestral branch compared to the rest in the concatenated phylogeny (posterior probability = 1, Fig. 4). In order to test if there is a growth in *D. renale* population, 3 statistical analyses were performed (Table 4). Tajima's *D*, Fu's *F* and Ramos Onsis and Rozas' *R*^2^ tests are consistent in that no deviation from what is expected under drift/mutation equilibrium is observed, and therefore no population growth is evident.

**Table 4.** *D. renale* population growth

Marker	*D*	*α* = 0.025		*F*	*α* = 0.05	*R*^2^	*α* = 0.05
		2.50%	97.50%		5.00%		5.00%
COX1	−0.700896	−1.69456	1.92159	−3.42948	−4.63243	0.092026	0.062865
ND4	−1.21541	−1.71761	1.82527	−1.77377	−3.98389	0.074853	0.0660138
Concatenated (COX1-ND4)	−0.961274	−1.75164	1.88549	−3.38549	−4.79482	0.086563	0.0673349

Values of *D*, *F* and *R*^2^ statistics obtained for different markers in contrast with what is expected for *α* = 0.05.

**Figure 7.**
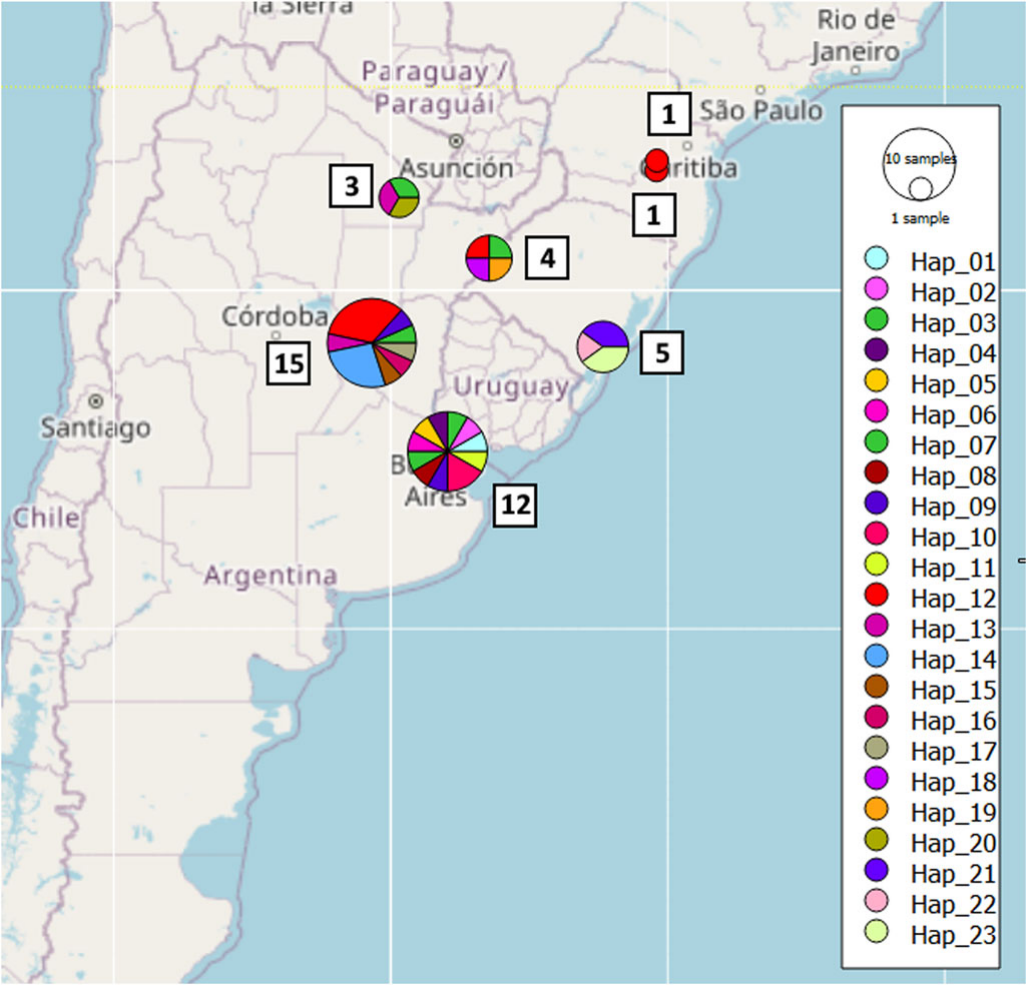
Distribution of the different haplotypes COX1-ND4 concatenated across the locations from which samples were received.

Haplotype networks obtained with concatenated marker (COX1 + ND4) do not show direct connection of haplotypes coming from the same node. There is no haplogroup differentiation by locality nor host species (Fig. 6). The haplotype network obtained with ND4 marker shows 1 central haplotype (H2) with highest frequency, widely distributed and present in both *C. brachyurus* and *Canis lupus familiaris*. This could be an ancestral haplotype from which emerges the rest (Supplementary Files). Although more samples were received from Santa Fe, the highest number of haplotypes was observed in Buenos Aires (Fig. 7).

## Discussion

Nematode parasites affecting wild animals are under-studied in South America. Frequently, the identification of these parasites is based on morphological determinations. Mitochondrial DNA-based markers employed in diagnosis offer the advantage of having thousands of mitochondria per cell and several mitochondrial genome copies per organelle, allowing the detection of parasites even when there is a limiting amount of DNA. Amplification and sequencing of COX1 gene is the barcoding approach currently and extensively used for high-throughput species delimitation and discovery (Hebert *et al*., 2003). However, the amplification of large fragments of COX1 and obtaining a good quality sequence is not always possible. Parasites collected from hosts that were road-killed and have spent several days decomposing, are often poorly preserved. In this case, PCRs with smaller targets are more sensitive than those with larger amplicons. Since both, specificity and sensitivity are required for species determination, we designed primers to amplify different sections of the COX1 gene. This approach allowed us to evaluate different alternatives. The COX1-L primers were designed to amplify the COX1 region standardized for barcoding (Hebert *et al*., 2003). This marker showed a lower proportion of polymorphic sites than other markers evaluated. Nevertheless, it has still proven useful for the molecular analysis of *D. renale* (this work) and other nematode species (Poon *et al*., 2017). The COX1-M marker showed the highest genetic diversity and could be the marker of choice when sample status prevents sequencing of the larger markers. However, extremely difficult samples with degraded DNA, poor quality and/or low mass could be analysed by COX1-S marker since has high sensitivity and is still retain sufficient genetic information. Based on these results the molecular markers COX1-S and COX1-M developed in this work has the best performance, particularly when morphological determinations are challenging due to small or poorly preserved samples and accurate species identification is required. The higher mutation rate due to mitochondrial oxidative stress, which is about 10 times higher than that of nuclear DNA, is an advantage for using mtDNA to study diversity. This increased mutation rate increases its potential for population studies without the bias of recombination events. The greatest variation is observed in the COX1-M section of the gene which, together with the ND4 marker, would be the best candidates for population genetics and phylogeography studies of this species. In order to evaluate the genetic diversity found in this work against genetic information from parasites sampled in other regions of the world, COX1-S was chosen as the marker since the available sequences from other countries are short. The phylogenetic trees obtained with COX1-S marker indicate that the South American samples, including the sample from Peru, are highly divergent from those found in other regions of the world (Canada and Iran). Furthermore, if this segregation would have occurred 3 million years ago, according to *Trichinella* mtDNA substitution rate, the variants found would have originated well before the arrival of domestic fauna in the region. This divergence time is consistent with Great American Biotic Interchange, a massive exchange of flora and fauna species between the North and South American landmasses resulting from the formation of the Isthmus of Panama. Then *D. renale* may have dispersed with its hosts between the north and south of the continent following the formation of the isthmus. The co-divergence of parasitic nematodes with the mastofauna they infect has already been proposed by Jimenez *et al*. (2017). Based on the limited sequence information available for this parasite, these preliminary results suggest that *D. renale* would have colonized the area in a gradual process dispersed with wildlife and would not be the result of an anthropogenic introduction. No genetic structuring by locality or host was observed in the studied area, as evidenced by data from Bayesian phylogenies and haplotype networks. *D. renale* does not seem to encounter geographical barriers that prevent its dispersal throughout the study area. However, the statistical analysis performed shows that geographical distribution is better at explaining genetic diversity than host species. This may indicate a small contribution of geographical distribution, which increases when the Chaco and Corrientes populations are considered as one, probably due to shared water resources from the Parana River but still less than 6% of the genetic diversity (Supplementary file S6). Furthermore, the Mantel test revealed no evidence of isolation by distance. Establishing a source/sink relationship between the studied localities is challenging, but it is worth noting that Buenos Aires Province exhibited higher haplotype diversity, but lower nucleotide diversity compared to Santa Fe Province in Argentina. On the other hand, Santa Fe has haplotypes absent in Buenos Aires Province and in some cases shares haplotypes with Brazil. This suggests that Santa Fe Province population is a contact zone between 2 regions, Buenos Aires Province (more temperate areas) and northern Argentina/southern Brazil (more tropical areas). This would also explain the greater nucleotide diversity that is observed when populations are structured. No differences in haplotype diversities were observed between Brazil and Argentina, making it difficult to establish a clear direction of transmission between the two countries. This may be attributed to the distant evolutionary time during which this process occurred and the large scale of migrations. The observed pattern supports the idea of multiple transmissions of parasites between domestic and wild animals, with several haplotypes being shared among hosts. Since no significant differences were found in the haplotype diversities between domestic and wild species, it cannot be assumed that there is unidirectional transmission. Instead, this pattern is indicative of a high rate of transmission occurring in both directions between domestic and wild species. Spillover of parasites at the domestic animal – wildlife interface is a pervasive threat to animal and human health. This information is crucial for future studies aiming to understand the ecology of the parasite in South America and its impact on wild populations, as well as risk of transmission to humans. Population expansion was examined in this study using 2 commonly used marker genes in nematode population studies. Our results showed no significant evidence of population growth in the studied area. Considering the number of informative sites studied, results suggest that *D. renale* has been present in the region for a considerable period of time, enough to lose the genetic imprint of their colonization.

Prior to this work, very little information was available on the diversity of *D. renale*, a zoonotic parasite that is widespread in riparian regions of Argentina and southern Brazil. Here we provide the descriptions of the genetic diversity of the parasite in the region by complementing molecular methods and classical and probabilistic phylogeography. Our results strongly suggest that this parasite has been present on the continent long enough to develop local genetic variants, rather than being the product of an introduction from the countries where it is reported. Although it is widely distributed in the molecular data, there is no significant evidence of population expansion. Also, the phylogenies show transmission between localities and bidirectional transmission between domestic and wild species.

## Supporting information

Arce et al. supplementary material 1 Arce et al. supplementary material

Arce et al. supplementary material 2 Arce et al. supplementary material

## Data Availability

All nucleotide sequences are available at GenBank under accession numbers OP208282.1 to OP208330.1, and OP204915.1 to OP204944.

## Supplementary material

The supplementary material for this article can be found at https://doi.org/10.1017/S0031182024001379.
