# Supplementary material for: Genetic diversity of Dioctophyme renale in Southern South America
Source: Parasitology. 2024 Oct 28;152(8):776–86. doi: 10.1017/S0031182024001379 (PMC12644968; doi:10.1017/S0031182024001379)
Supplement: Arce et al. supplementary material 1 — Arce et al. supplementary material [file S0031182024001379sup001.pdf]

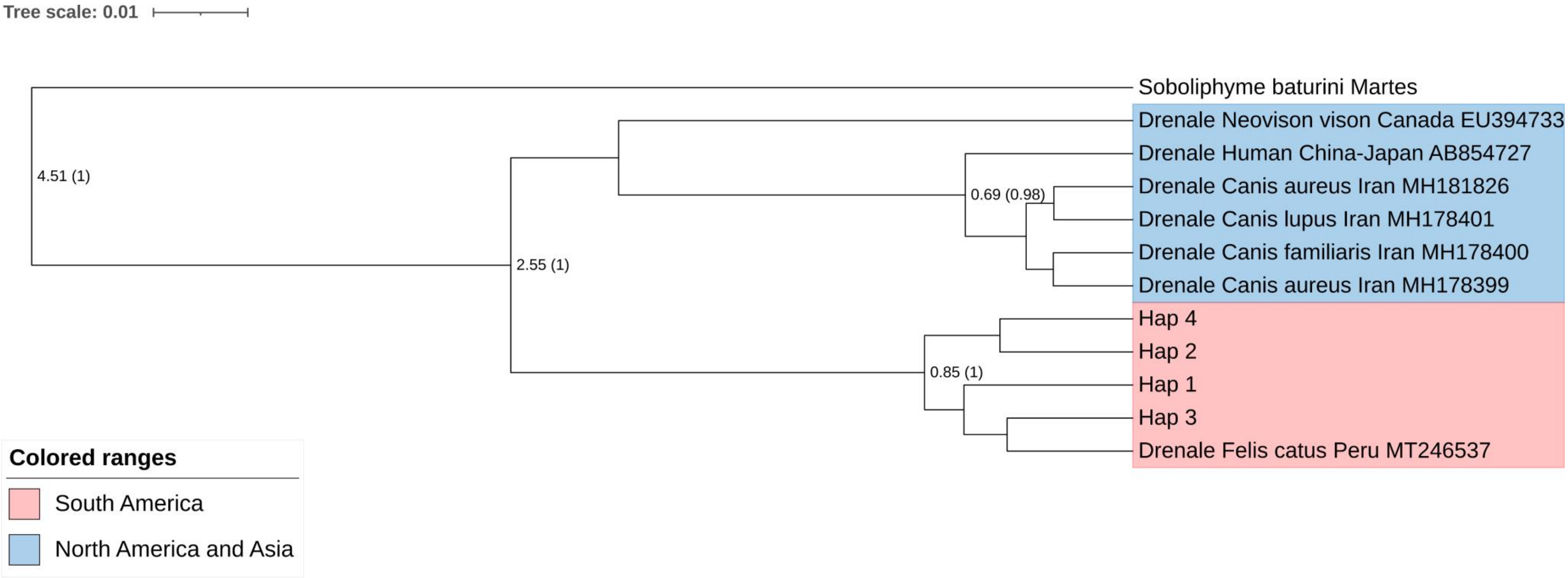

Tree scale: 0.01
Soboliphyme baturini Martes
Drenale Neovison vison Canada EU394733
Drenale Human China-Japan AB854727
Drenale Canis aureus Iran MH181826
Drenale Canis lupus Iran MH178401
Drenale Canis familiaris Iran MH178400
Drenale Canis aureus Iran MH178399
Hap 4
Hap 2
Hap 1
Hap 3
Drenale Felis catus Peru MT246537
4.51 (1)
2.55 (1)
0.69 (0.98)
0.85 (1)
Colored ranges
South America
North America and Asia

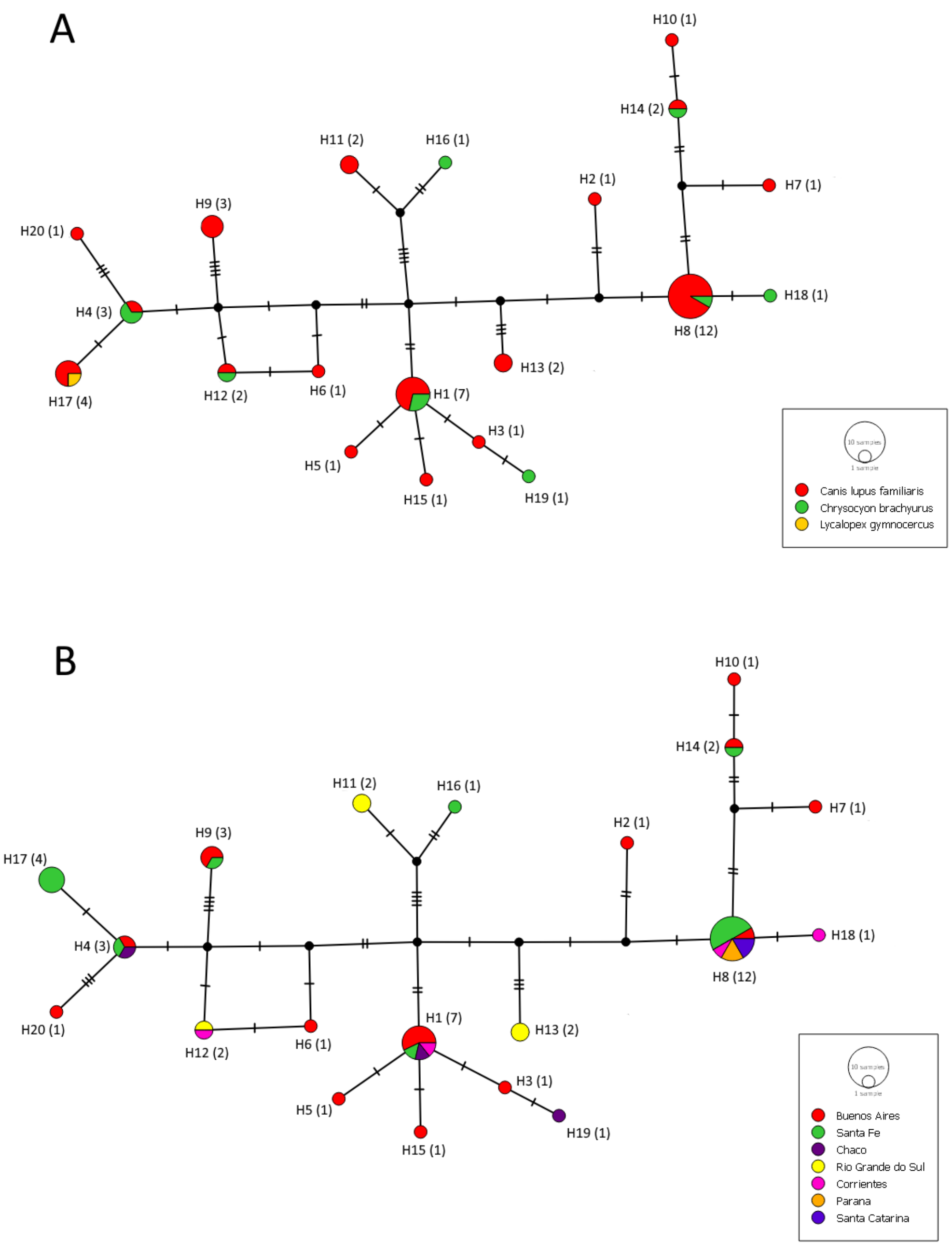
A
H10 (1)
H14 (2)
H11 (2)
H16 (1)
H2 (1)
H7 (1)
H9 (3)
H20 (1)
H4 (3)
H18 (1)
H8 (12)
H13 (2)
H17 (4)
H12 (2)
H6 (1)
H1 (7)
H3 (1)
H5 (1)
H15 (1)
H19 (1)
Canis lupus familiaris
Chrysocyon brachyurus
Lycalopex gymnocercus
B
H10 (1)
H14 (2)
H11 (2)
H16 (1)
H7 (1)
H2 (1)
H9 (3)
H17 (4)
H4 (3)
H18 (1)
H8 (12)
H20 (1)
H12 (2)
H6 (1)
H1 (7)
H13 (2)
H3 (1)
H5 (1)
H15 (1)
H19 (1)
Buenos Aires
Santa Fe
Chaco
Rio Grande do Sul
Corrientes
Parana
Santa Catarina

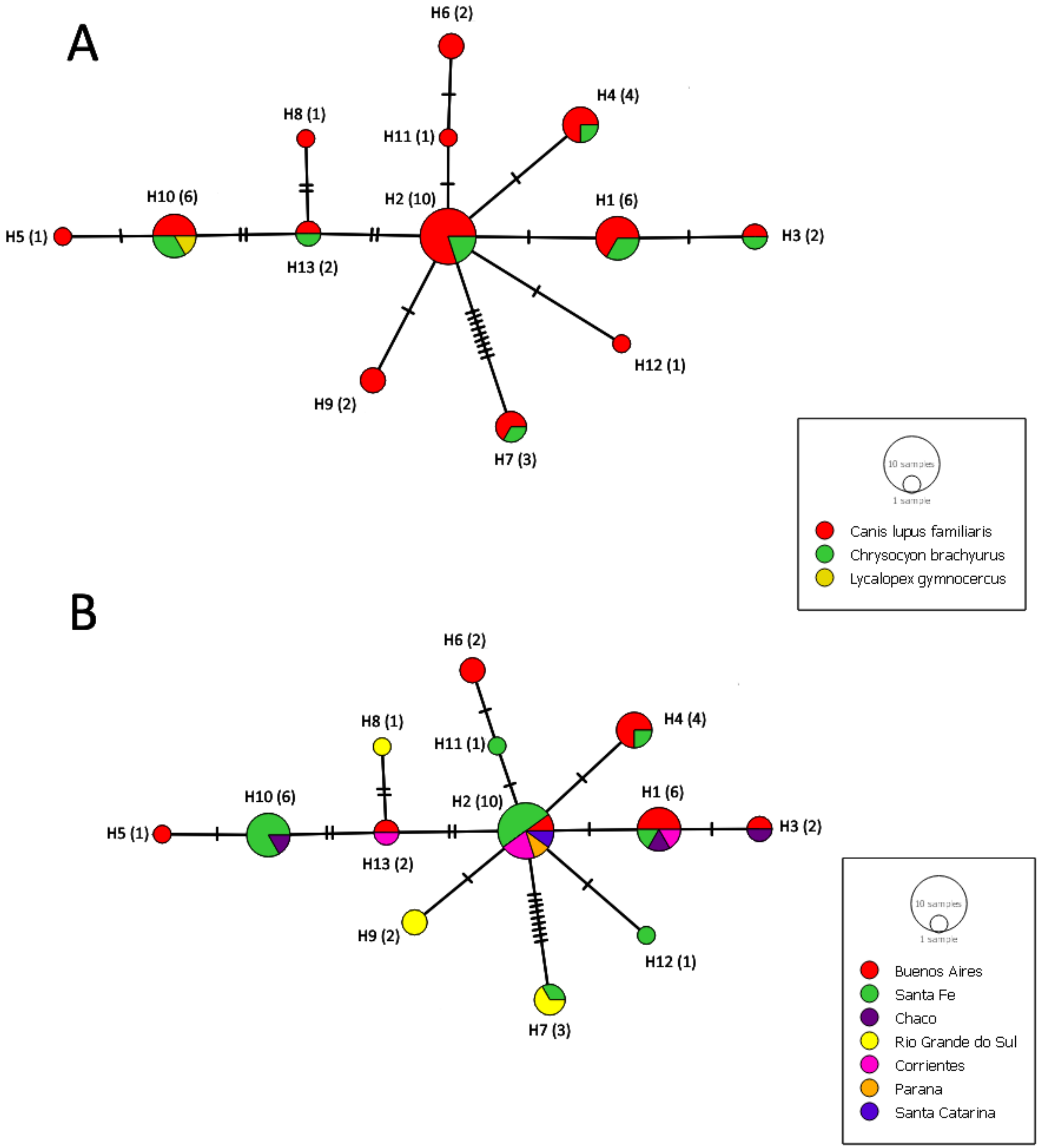
A
H6 (2)
H4 (4)
H8 (1)
H11 (1)
H10 (6)
H2 (10)
H1 (6)
H5 (1)
H3 (2)
H13 (2)
H12 (1)
H9 (2)
H7 (3)
10 samples
1 sample
Canis lupus familiaris
Chrysocyon brachyurus
Lycalopex gymnocercus
B
H6 (2)
H4 (4)
H8 (1)
H11 (1)
H10 (6)
H2 (10)
H1 (6)
H5 (1)
H3 (2)
H13 (2)
H9 (2)
H12 (1)
H7 (3)
10 samples
1 sample
Buenos Aires
Santa Fe
Chaco
Rio Grande do Sul
Corrientes
Parana
Santa Catarina

COX1-S Multiple Sequence Alignment

```
                ....|....| ....|....| ....|....| ....|....| ....|....|
                    5         15         25         35         45
Dren01_Cf_BA    AGGTTTAGAT GTTGATACGC GTGCGTATTT TGCTGCTGCG AGTATAATCA
Dren02_Cf_BA    AGGTTTAGAT GTTGATACGC GTGCGTATTT TGCTGCTGCG AGTATAATCA
Dren03_Cf_BA    AGGTTTAGAT GTTGATACGC GTGCGTATTT TGCTGCTGCG AGTATAATCA
Dren04_Cf_BA    AGGTTTAGAT GTTGATACGC GTGCGTATTT TGCTGCTGCG AGTATAATCA
Dren05_Cf_BA    AGGTTTAGAT GTTGATACGC GTGCGTATTT TGCTGCTGCG AGTATAATCA
Dren06_Cf_BA    AGGTTTAGAT GTTGATACGC GTGCGTATTT TGCTGCTGCG AGTATAATCA
Dren07_Cf_BA    AGGTTTAGAT GTTGATACGC GTGCGTATTT TGCTGCTGCG AGTATAATCA
Dren08_Cf_BA    AGGTTTAGAT GTTGATACGC GTGCGTATTT TGCTGCTGCG AGTATAATCA
Dren09_Cf_BA    AGGTTTAGAT GTTGATACGC GTGCGTATTT TGCTGCTGCG AGTATAATCA
Dren10_Cf_BA    AGGTTTAGAT GTTGATACGC GTGCGTATTT TGCTGCTGCG AGTATAATCA
Dren11_Cf_BA    AGGTTTAGAT GTTGATACGC GTGCGTATTT TGCTGCTGCG AGTATAATCA
Dren18_Cf_BA    AGGTTTAGAT GTTGATACGC GTGCGTATTT TGCTGCTGCG AGTATAATCA
Dren19_Cf_BA    AGGTTTAGAT GTTGATACGC GTGCGTATTT TGCTGCTGCG AGTATAATCA
Dren23_Cf_BA    AGGTTTAGAT GTTGATACGC GTGCGTATTT TGCTGCTGCG AGTATAATCA
Dren24_Cf_BA    AGGTTTAGAT GTTGATACGC GTGCGTATTT TGCTGCTGCG AGTATAATCA
Dren25_Cf_SF    AGGTTTAGAT GTTGATACGC GTGCGTATTT TGCTGCTGCG AGTATAATCA
Dren28_Fc_Pa    AGGTTTAGAT GTTGATACGC GTGCGTATTT TGCTGCTGCG AGTATAATCA
Dren29_Fc_SC    AGGTTTAGAT GTTGATACGC GTGCGTATTT TGCTGCTGCG AGTATAATCA
Dren30_Gc_SC    AGGTTTAGAT GTTGATACGC GTGCGTATTT TGCTGCTGCG AGTATAATCA
Dren32_Cf_SC    AGGTTTAGAT GTTGATACGC GTGCGTATTT TGCTGCTGCG AGTATAATCA
Dren33_Cf_SC    AGGTTTAGAT GTTGATACGC GTGCGTATTT TGCTGCTGCG AGTATAATCA
Dren34_Cf_Pa    AGGTTTAGAT GTTGATACGC GTGCGTATTT TGCTGCTGCG AGTATAATCA
Dren35_Cf_Pa    AGGTTTAGAT GTTGATACGC GTGCGTATTT TGCTGCTGCG AGTATAATCA
Dren40_Cf_RG    AGGTTTAGAT GTTGATACGC GTGCGTATTT TGCTGCTGCG AGTATAATCA
Dren41_Cf_RG    AGGTTTAGAT GTTGATACGC GTGCGTATTT TGCTGCTGCG AGTATAATCA
Dren42_Cf_RG    AGGTTTAGAT GTTGATACGC GTGCGTATTT TGCTGCTGCG AGTATAATCA
Dren43_Cf_RG    AGGTTTAGAT GTTGATACGC GTGCGTATTT TGCTGCTGCG AGTATAATCA
Dren45_Cf_RG    AGGTTTAGAT GTTGATACGC GTGCGTATTT TGCTGCTGCG AGTATAATCA
Dren46_Cf_SF    AGGTTTAGAT GTTGATACGC GTGCGTATTT TGCTGCTGCG AGTATAATCA
Dren47_Cb_SF    AGGTTTAGAT GTTGATACGC GTGCGTATTT TGCTGCTGCG AGTATAATCA
Dren48_Cf_BA    AGGTTTAGAT GTTGATACGC GTGCGTATTT TGCTGCTGCG AGTATAATCA
Dren49_Cf_BA    AGGTTTAGAT GTTGATACGC GTGCGTATTT TGCTGCTGCG AGTATAATCA
Dren50_Cf_Ch    AGGTTTAGAT GTTGATACGC GTGCGTATTT TGCTGCTGCG AGTATAATCA
Dren53_Cf_BA    AGGTTTAGAT GTTGATACGC GTGCGTATTT TGCTGCTGCG AGTATAATCA
Dren55_Cb_Co    AGGTTTAGAT GTTGATACGC GTGCGTATTT TGCTGCTGCG AGTATAATCA
Dren56_Cb_Co    AGGTTTAGAT GTTGATACGC GTGCGTATTT TGCTGCTGCG AGTATAATCA
Dren58_Cb_SF    AGGTTTAGAT GTTGATACGC GTGCGTATTT TGCTGCTGCG AGTATAATCA
Dren66_Cf_SF    AGGTTTAGAT GTTGATACGC GTGCGTATTT TGCTGCTGCG AGTATAATCA
Dren67_Cf_SF    AGGTTTAGAT GTTGATACGC GTGCGTATTT TGCTGCTGCG AGTATAATCA
Dren68_Cf_SF    AGGTTTAGAT GTTGATACGC GTGCGTATTT TGCTGCTGCG AGTATAATCA
Dren69_Cf_SF    AGGTTTAGAT GTTGATACGC GTGCGTATTT TGCTGCTGCG AGTATAATCA
Dren70_Cf_SF    AGGTTTAGAT GTTGATACGC GTGCGTATTT TGCTGCTGCG AGTATAATCA
Dren72_Cb_SF    AGGTTTAGAT GTTGATACGC GTGCGTATTT TGCTGCTGCG AGTATAATCA
Dren73_Cb_Ch    AGGTTTAGAT GTTGATACGC GTGCGTATTT TGCTGCTGCG AGTATAATCA
Dren74_Cb_Co    AGGTTTAGAT GTTGATACGC GTGCGTATTT TGCTGCTGCG AGTATAATCA
Dren75_Cb_Co    AGGTTTAGAT GTTGATACGC GTGCGTATTT TGCTGCTGCG AGTATAATCA
Dren80_Cf_SF    AGGTTTAGAT GTTGATACGC GTGCGTATTT TGCTGCTGCG AGTATAATCA
Dren81_Cf_SF    AGGTTTAGAT GTTGATACGC GTGCGTATTT TGCTGCTGCG AGTATAATCA
Dren82_Cb_Co    AGGTTTAGAT GTTGATACGC GTGCGTATTT TGCTGCTGCG AGTATAATCA
Dren83_Cb_Ch    AGGTTTAGAT GTTGATACGC GTGCGTATTT TGCTGCTGCG AGTATAATCA
Dren84_Cf_SF    AGGTTTAGAT GTTGATACGC GTGCGTATTT TGCTGCTGCG AGTATAATCA
Dren85_Cb_SF    AGGTTTAGAT GTTGATACGC GTGCGTATTT TGCTGCTGCG AGTATAATCA
Dren86_Cb_Co    AGGTTTAGAT GTTGATACGC GTGCGTATTT TGCTGCTGCG AGTATAATCA
Dren87_Cf_BA    AGGTTTAGAT GTTGATACGC GTGCGTATTT TGCTGCTGCG AGTATAATCA
Dren88_Lg_SF    AGGTTTAGAT GTTGATACGC GTGCGTATTT TGCTGCTGCG AGTATAATCA

                ....|....| ....|....| ....
                    55         65
```

```
Dren01_Cf_BA   TCGGTATTCC GACGGGGGTA AAAG
Dren02_Cf_BA   TCGGTATTCC GACGGGGGTA AAAG
Dren03_Cf_BA   TCGGTATTCC GACGGGGGTA AAAG
Dren04_Cf_BA   TCGGTATTCC GACGGGGGTA AAAG
Dren05_Cf_BA   TCGGTATTCC GACAGGGGTA AAAG
Dren06_Cf_BA   TCGGTATTCC GACGGGGGTA AAAG
Dren07_Cf_BA   TCGGTATTCC GACAGGGGTA AAAG
Dren08_Cf_BA   TCGGTATTCC GACGGGGGTA AAAG
Dren09_Cf_BA   TCGGTATTCC GACGGGGGTA AAAG
Dren10_Cf_BA   TCGGTATTCC GACGGGGGTA AAAG
Dren11_Cf_BA   TCGGTATTCC GACGGGGGTA AAAG
Dren18_Cf_BA   TCGGTATTCC GACAGGGGTA AAAG
Dren19_Cf_BA   TCGGTATTCC GACAGGGGTA AAAG
Dren23_Cf_BA   TCGGTATTCC GACGGGGGTA AAAG
Dren24_Cf_BA   TCGGTATTCC GACGGGGGTA AAAG
Dren25_Cf_SF   TCGGTATTCC GACGGGGGTA AAAG
Dren28_Fc_Pa   TCGGTATTCC GACGGGGGTA AAAG
Dren29_Fc_SC   TCGGTATTCC GACGGGGGTA AAAG
Dren30_Gc_SC   TCGGTATTCC GACGGGGGTA AAAG
Dren32_Cf_SC   TCGGTATTCC GACGGGGGTA AAAG
Dren33_Cf_SC   TCGGTATTCC GACGGGGGTA AAAG
Dren34_Cf_Pa   TCGGTATTCC GACGGGGGTA AAAG
Dren35_Cf_Pa   TCGGTATTCC GACGGGGGTA AAAG
Dren40_Cf_RG   TCGGTATCCC GACGGGGGTA AAAG
Dren41_Cf_RG   TCGGTATCCC GACGGGGGTA AAAG
Dren42_Cf_RG   TCGGTATTCC GACAGGGGTA AAAG
Dren43_Cf_RG   TCGGTATTCC GACGGGGGTA AAAG
Dren45_Cf_RG   TCGGTATTCC GACGGGGGTA AAAG
Dren46_Cf_SF   TCGGTATTCC GACGGGGGTA AAAG
Dren47_Cb_SF   TCGGTATTCC GACAGGGGTA AAAG
Dren48_Cf_BA   TCGGTATTCC GACGGGGGTA AAAG
Dren49_Cf_BA   TCGGTATTCC GACGGGGGTA AAAG
Dren50_Cf_Ch   TCGGTATTCC GACGGGGGTA AAAG
Dren53_Cf_BA   TCGGTATTCC GACGGGGGTA AAAG
Dren55_Cb_Co   TCGGTATTCC GACGGGGGTA AAAG
Dren56_Cb_Co   TCGGTATTCC GACAGGGGTA AAAG
Dren58_Cb_SF   TCGGTATCCC GACGGGGGTA AAAG
Dren66_Cf_SF   TCGGTATTCC GACAGGGGTA AAAG
Dren67_Cf_SF   TCGGTATTCC GACAGGGGTA AAAG
Dren68_Cf_SF   TCGGTATTCC GACAGGGGTA AAAG
Dren69_Cf_SF   TCGGTATTCC GACAGGGGTA AAAG
Dren70_Cf_SF   TCGGTATTCC GACGGGGGTA AAAG
Dren72_Cb_SF   TCGGTATTCC GACGGGGGTA AAAG
Dren73_Cb_Ch   TCGGTATTCC GACAGGGGTA AAAG
Dren74_Cb_Co   TCGGTATTCC GACGGGGGTA AAAG
Dren75_Cb_Co   TCGGTATTCC GACGGGGGTA AAAG
Dren80_Cf_SF   TCGGTATTCC GACGGGGGTA AAAG
Dren81_Cf_SF   TCGGTATTCC GACGGGGGTA AAAG
Dren82_Cb_Co   TCGGTATTCC GACGGGGGTA AAAG
Dren83_Cb_Ch   TCGGTATTCC GACGGGGGTA AAAG
Dren84_Cf_SF   TCGGTATTCC GACGGGGGTA AAAG
Dren85_Cb_SF   TCGGTATTCC GACGGGGGTA AAAG
Dren86_Cb_Co   TCGGTATTCC GACAGGGGTA AAAG
Dren87_Cf_BA   TCGGTATCCC GACAGGGGTA AAAG
Dren88_Lg_SF   TCGGTATTCC GACAGGGGTA AAAG
```

COX1-M Multiple Sequence Alignment

```
                ....|....| ....|....| ....|....| ....|....| ....|....|
                    5         15         25         35         45
Dren01_Cf_BA   TAGAGGCAAG CCAAAGGTGT TTGGACCTTT GGGTATAATC TACGCTATAA
Dren02_Cf_BA   TAGTGGCAAG CCAAAGGTGT TTGGACCTTT GGGTATAATT TACGCTATAA
Dren03_Cf_BA   TAGAGGCAAG CCAAAGGTGT TTGGACCTTT GGGTATAATC TACGCTATAA
Dren04_Cf_BA   TAGTGGCAAG CCAAAGGTGT TTGGACCTTT GGGTATAATC TACGCTATAA
Dren05_Cf_BA   TAGTGGCAAG CCAAAGGTGT TTGGGCCTTT GGGCATAATC TACGCTATAA
Dren06_Cf_BA   TAGAGGCAAG CCAAAGGTGT TTGGACCTTT GGGTATAATC TACGCTATAA
Dren07_Cf_BA   TAGTGGCAAG CCAAAGGTGT TTGGGCCTTT GGGTATAATC TACGCTATAA
Dren08_Cf_BA   TAGAGGCAAG CCAAAGGTGT TTGGACCTTT GGGTATAATC TACGCTATAA
Dren09_Cf_BA   TAGTGGCAAG CCAAAGGTGT TTGGACCTTT GGGTATAATC TACGCTATAA
Dren10_Cf_BA   TAGAGGCAAG CCAAAGGTGT TTGGACCTTT GGGTATAATC TACGCTATAA
Dren11_Cf_BA   TAGTGGCAAG CCAAAGGTGT TTGGACCTTT GGGTATAATC TACGCTATAA
Dren18_Cf_BA   TAGTGGTAAG CCAAAGGTGT TTGGACCTTT GGGCATAATC TACGCTATAA
Dren19_Cf_BA   TAGTGGTAAG CCAAAGGTGT TTGGACCTTT GGGCATAATC TACGCTATAA
Dren23_Cf_BA   TAGTGGCAAG CCAAAGGTGT TTGGACCTTT GGGTATAATC TACGCTATAA
Dren24_Cf_BA   TAGTGGCAAG CCAAAGGTGT TTGGACCTTT GGGCATAATC TACGCTATAA
Dren25_Cf_SF   TAGTGGCAAG CCAAAGGTGT TTGGACCTTT GGGTATAATC TACGCTATAA
Dren28_Fc_Pa   TAGTGGCAAG CCAAAGGTGT TTGGACCTTT GGGTATAATC TACGCTATAA
Dren29_Fc_SC   TAGTGGCAAG CCAAAGGTGT TTGGACCTTT GGGTATAATC TACGCTATAA
Dren32_Cf_SC   TAGTGGCAAG CCAAAGGTGT TTGGACCTTT GGGTATAATC TACGCTATAA
Dren33_Cf_SC   TAGTGGCAAG CCAAAGGTGT TTGGACCTTT GGGTATAATC TACGCTATAA
Dren34_Cf_Pa   TAGTGGCAAG CCAAAGGTGT TTGGACCTTT GGGTATAATC TACGCTATAA
Dren35_Cf_Pa   TAGTGGCAAG CCAAAGGTGT TTGGACCTTT GGGTATAATC TACGCTATAA
Dren40_Cf_RG   TAGTGGCAAG CCAAAGGTGT TTGGACCTTT GGGTATAATC TACGCTATAA
Dren41_Cf_RG   TAGTGGCAAG CCAAAGGTGT TTGGACCTTT GGGTATAATC TACGCTATAA
Dren42_Cf_RG   TAGTGGCAAG CCAAAGGTGT TTGGGCCTTT GGGCATAATC TACGCTATAA
Dren43_Cf_RG   TAGCGGTAAG CCAAAGGTGT TTGGACCTTT GGGTATAATC TACGCTATAA
Dren45_Cf_RG   TAGCGGTAAG CCAAAGGTGT TTGGACCTTT GGGTATAATC TACGCTATAA
Dren46_Cf_SF   TAGTGGCAAG CCAAAGGTGT TTGGACCTTT GGGTATAATC TACGCTATAA
Dren47_Cb_SF   TAGTGGCAAG CCAAAGGTGT TTGGGCCTTT GGGCATAATC TACGCTATAA
Dren48_Cf_BA   TAGAGGCAAG CCAAAGGTGT TTGGACCTTT GGGTATAATC TACGCTATAA
Dren49_Cf_BA   TAGTGGCAAG CCAAAGGTGT TTGGACCTTT GGGTATAATC TACGCTATAA
Dren50_Cf_Ch   TAGAGGCAAG CCAAAGGTGT TTGGACCTTT GGGTATAATC TACGCTATAA
Dren53_Cf_BA   TAGAGGCAAG CCAAAGGTGT TTGGACCTTT GGGTATAATC TACGCTATAA
Dren58_Cb_SF   TAGTGGCAAG CCAAAGGTGT TTGGACCTTT GGGTATAATC TACGCTATAA
Dren66_Cf_SF   TAGTGGTAAG CCAAAGGTGT TTGGACCTTT GGGCATAATC TACGCTATAA
Dren67_Cf_SF   TAGTGGCAAG CCAAAGGTGT TTGGGCCTTT GGGCATAATC TACGCTATAA
Dren68_Cf_SF   TAGTGGCAAG CCAAAGGTGT TTGGGCCTTT GGGCATAATC TACGCTATAA
Dren69_Cf_SF   TAGTGGCAAG CCAAAGGTGT TTGGGCCTTT GGGCATAATC TACGCTATAA
Dren70_Cf_SF   TAGTGGCAAG CCAAAGGTGT TTGGACCTTT GGGTATAATC TACGCTATAA
Dren72_Cb_SF   TAGAGGCAAG CCAAAGGTGT TTGGACCTTT GGGTATAATC TACGCTATAA
Dren73_Cb_Ch   TAGTGGCAAG CCAAAGGTGT TTGGGCCTTT GGGCATAATC TACGCTATAA
Dren74_Cb_Co   TAGTGGCAAG CCAAAGGTGT TTGGACCTTT GGGTATAATC TACGCTATAA
Dren75_Cb_Co   TAGAGGCAAG CCAAAGGTGT TTGGACCTTT GGGTATAATC TACGCTATAA
Dren80_Cf_SF   TAGTGGCAAG CCAAAGGTGT TTGGACCTTT GGGTATAATC TACGCTATAA
Dren81_Cf_SF   TAGTGGCAAG CCAAAGGTGT TTGGACCTTT GGGTATAATC TACGCTATAA
Dren82_Cb_Co   TAGTGGCAAG CCAAAGGTGT TTGGACCTTT GGGTATAATC TACGCTATAA
Dren83_Cb_Ch   TAGAGGCAAG CCAAAGGTGT TTGGACCTTT GGGTATAATC TATGCTATAA
Dren84_Cf_SF   TAGTGGCAAG CCAAAGGTGT TTGGACCTTT GGGTATAATC TACGCTATAA
Dren85_Cb_SF   TAGTGGCAAG CCAAAGGTGT TTGGACCTTT GGGTATAATC TACGCTATAA
Dren86_Cb_Co   TAGTGGCAAG CCAAAGGTGT TTGGGCCTTT GGGCATAATC TACGCTATAA
Dren87_Cf_BA   TAGTGGCAAG CCAAAGGTGT TTGGGCCTTT GGGCATAATC TACGCTATAA
Dren88_Lg_SF   TAGTGGCAAG CCAAAGGTGT TTGGGCCTTT GGGCATAATC TACGCTATAA

                ....|....| ....|....| ....|....| ....|....| ....|....|
                    55         65         75         85         95
Dren01_Cf_BA   TTAGAATTGG CGTGCTTGGT TGTTTTGTGT GGGTACATCA CATGTTTACT
Dren02_Cf_BA   TTAGAATTGG CGTGCTTGGT TGTTTTGTGT GGGTACATCA CATGTTTACT
Dren03_Cf_BA   TTAGAATTGG CGTGCTTGGT TGTTTTGTGT GGGTACATCA CATGTTTACT
```

```
Dren04_Cf_BA   TTAGAATTGG CGTGCTTGGT TGTTTTGTGT GGGTGCATCA CATGTTTACT
Dren05_Cf_BA   TTAGAATTGG CGTGCTTGGT TGTTTTGTGT GGGTACATCA CATGTTTACT
Dren06_Cf_BA   TTAGAATTGG CGTGCTTGGT TGTTTTGTGT GGGTACATCA CATGTTTACT
Dren07_Cf_BA   TTAGAATTGG CGTGCTTGGT TGTTTTGTGT GGGTACATCA CATGTTTACT
Dren08_Cf_BA   TTAGAATTGG CGTGCTTGGT TGTTTTGTGT GGGTACATCA CATGTTTACT
Dren09_Cf_BA   TTAGAATTGG CGTGCTTGGT TGTTTTGTGT GGGTGCATCA CATGTTTACT
Dren10_Cf_BA   TTAGAATTGG CGTGCTTGGT TGTTTTGTGT GGGTACATCA CATGTTTACT
Dren11_Cf_BA   TTAGAATTGG CGTGCTTGGT TGTTTTGTGT GGGTACATCA CATGTTTACT
Dren18_Cf_BA   TTAGAATTGG CGTGCTTGGT TGTTTTGTGT GGGTACATCA CATGTTTACT
Dren19_Cf_BA   TTAGAATTGG CGTGCTTGGT TGTTTTGTGT GGGTACATCA CATGTTTACT
Dren23_Cf_BA   TTAGAATTGG CGTGCTTGGT TGTTTTGTGT GGGTGCATCA CATGTTTACT
Dren24_Cf_BA   TTAGAATTGG CGTGCTTGGT TGTTTTGTGT GGGTACATCA CATGTTTACT
Dren25_Cf_SF   TTAGAATTGG CGTGCTTGGT TGTTTTGTGT GGGTACATCA CATGTTTACT
Dren28_Fc_Pa   TTAGAATTGG CGTGCTTGGT TGTTTTGTGT GGGTACATCA CATGTTTACT
Dren29_Fc_SC   TTAGAATTGG CGTGCTTGGT TGTTTTGTGT GGGTACATCA CATGTTTACT
Dren32_Cf_SC   TTAGAATTGG CGTGCTTGGT TGTTTTGTGT GGGTACATCA CATGTTTACT
Dren33_Cf_SC   TTAGAATTGG CGTGCTTGGT TGTTTTGTGT GGGTACATCA CATGTTTACT
Dren34_Cf_Pa   TTAGAATTGG CGTGCTTGGT TGTTTTGTGT GGGTACATCA CATGTTTACT
Dren35_Cf_Pa   TTAGAATTGG CGTGCTTGGT TGTTTTGTGT GGGTACATCA CATGTTTACT
Dren40_Cf_RG   TTAGAATTGG CGTGCTTGGT TGTTTTGTGT GGGTACATCA CATGTTTACT
Dren41_Cf_RG   TTAGAATTGG CGTGCTTGGT TGTTTTGTGT GGGTACATCA CATGTTTACT
Dren42_Cf_RG   TTAGAATTGG CGTGCTTGGT TGTTTTGTGT GGGTACATCA CATGTTTACT
Dren43_Cf_RG   TTAGAATTGG CGTGCTTGGT TGTTTTGTGT GGGTACATCA CATGTTTACT
Dren45_Cf_RG   TTAGAATTGG CGTGCTTGGT TGTTTTGTGT GGGTACATCA CATGTTTACT
Dren46_Cf_SF   TTAGAATTGG CGTGCTTGGT TGTTTTGTGT GGGTACATCA CATGTTTACT
Dren47_Cb_SF   TTAGAATTGG CGTGCTTGGT TGTTTTGTGT GGGTACATCA CATGTTTACT
Dren48_Cf_BA   TTAGAATTGG CGTGCTTGGT TGTTTTGTGT GGGTACATCA CATGTTTACT
Dren49_Cf_BA   TTAGAATTGG CGTGCTTGGT TGTTTTGTGT GGGTGCATCA CATGTTTACT
Dren50_Cf_Ch   TTAGAATTGG CGTGCTTGGT TGTTTTGTGT GGGTACATCA CATGTTTACT
Dren53_Cf_BA   TTAGAATTGG CGTGCTTGGT TGTTTTGTGT GGGTACATCA CATGTTTACT
Dren58_Cb_SF   TTAGAATTGG CGTGCTTGGT TGTTTTGTGT GGGTACATCA CATGTTTACT
Dren66_Cf_SF   TTAGAATTGG CGTGCTTGGT TGTTTTGTGT GGGTACATCA CATGTTTACT
Dren67_Cf_SF   TTAGAATTGG CGTGCTTGGT TGTTTTGTGT GGGTGCATCA CATGTTTACT
Dren68_Cf_SF   TTAGAATTGG CGTGCTTGGT TGTTTTGTGT GGGTGCATCA CATGTTTACT
Dren69_Cf_SF   TTAGAATTGG CGTGCTTGGT TGTTTTGTGT GGGTGCATCA CATGTTTACT
Dren70_Cf_SF   TTAGAATTGG CGTGCTTGGT TGTTTTGTGT GGGTACATCA CATGTTTACT
Dren72_Cb_SF   TTAGAATTGG CGTGCTTGGT TGTTTTGTGT GGGTACATCA CATGTTTACT
Dren73_Cb_Ch   TTAGAATTGG CGTGCTTGGT TGTTTTGTGT GGGTACATCA CATGTTTACT
Dren74_Cb_Co   TTAGAATTGG CGTGCTTGGT TGTTTTGTGT GGGTACATCA CATGTTTACT
Dren75_Cb_Co   TTAGAATTGG CGTGCTTGGT TGTTTTGTGT GGGTACATCA CATGTTTACT
Dren80_Cf_SF   TTAGAATTGG CGTGCTTGGT TGTTTTGTGT GGGTACATCA CATGTTTACT
Dren81_Cf_SF   TTAGAATTGG CGTGCTTGGT TGTTTTGTGT GGGTACATCA CATGTTTACT
Dren82_Cb_Co   TTAGAATTGG CGTGCTTGGT TGTTTTGTGT GGGTACATCA CATGTTTACT
Dren83_Cb_Ch   TTAGAATTGG CGTGCTTGGT TGTTTTGTGT GGGTACATCA CATGTTTACT
Dren84_Cf_SF   TTAGAATTGG CGTGCTTGGT TGTTTTGTGT GGGTACATCA CATGTTTACT
Dren85_Cb_SF   TTAGAATTGG CGTGCTTGGT TGTTTTGTGT GGGTGCATCA CATGTTTACT
Dren86_Cb_Co   TTAGAATTGG CGTGCTTGGT TGTTTTGTGT GGGTACATCA CATGTTTACT
Dren87_Cf_BA   TTAGAATTGG CGTGCTTGGT TGTTTTGTGT GGGTACATCA CATGTTTACT
Dren88_Lg_SF   TTAGAATTGG CGTGCTTGGT TGTTTTGTGT GGGTGCATCA CATGTTTACT

               ....|....| ....|....| ....|....| ....|....| ....|....|
                   105        115        125        135        145
Dren01_Cf_BA   GTAGGTTTAG ATGTTGATAC GCGTGCGTAT TTTGCTGCTG CGAGTATAAT
Dren02_Cf_BA   GTAGGTTTAG ATGTTGATAC GCGTGCGTAT TTTGCTGCTG CGAGTATAAT
Dren03_Cf_BA   GTAGGTTTAG ATGTTGATAC GCGTGCGTAT TTTGCTGCTG CGAGTATAAT
Dren04_Cf_BA   GTAGGTTTAG ATGTTGATAC GCGTGCGTAT TTTGCTGCTG CGAGTATAAT
Dren05_Cf_BA   GTAGGTTTAG ATGTTGATAC GCGTGCGTAT TTTGCTGCTG CGAGTATAAT
Dren06_Cf_BA   GTAGGTTTAG ATGTTGATAC GCGTGCGTAT TTTGCTGCTG CGAGTATAAT
Dren07_Cf_BA   GTAGGTTTAG ATGTTGATAC GCGTGCGTAT TTTGCTGCTG CGAGTATAAT
Dren08_Cf_BA   GTAGGTTTAG ATGTTGATAC GCGTGCGTAT TTTGCTGCTG CGAGTATAAT
Dren09_Cf_BA   GTAGGTTTAG ATGTTGATAC GCGTGCGTAT TTTGCTGCTG CGAGTATAAT
Dren10_Cf_BA   GTAGGTTTAG ATGTTGATAC GCGTGCGTAT TTTGCTGCTG CGAGTATAAT
Dren11_Cf_BA   GTAGGTTTAG ATGTTGATAC GCGTGCGTAT TTTGCTGCTG CGAGTATAAT
Dren18_Cf_BA   GTAGGTTTAG ATGTTGATAC GCGTGCGTAT TTTGCTGCTG CGAGTATAAT
```

```
Dren19_Cf_BA   GTAGGTTTAG ATGTTGATAC GCGTGCGTAT TTTGCTGCTG CGAGTATAAT
Dren23_Cf_BA   GTAGGTTTAG ATGTTGATAC GCGTGCGTAT TTTGCTGCTG CGAGTATAAT
Dren24_Cf_BA   GTAGGTTTAG ATGTTGATAC GCGTGCGTAT TTTGCTGCTG CGAGTATAAT
Dren25_Cf_SF   GTAGGTTTAG ATGTTGATAC GCGTGCGTAT TTTGCTGCTG CGAGTATAAT
Dren28_Fc_Pa   GTAGGTTTAG ATGTTGATAC GCGTGCGTAT TTTGCTGCTG CGAGTATAAT
Dren29_Fc_SC   GTAGGTTTAG ATGTTGATAC GCGTGCGTAT TTTGCTGCTG CGAGTATAAT
Dren32_Cf_SC   GTAGGTTTAG ATGTTGATAC GCGTGCGTAT TTTGCTGCTG CGAGTATAAT
Dren33_Cf_SC   GTAGGTTTAG ATGTTGATAC GCGTGCGTAT TTTGCTGCTG CGAGTATAAT
Dren34_Cf_Pa   GTAGGTTTAG ATGTTGATAC GCGTGCGTAT TTTGCTGCTG CGAGTATAAT
Dren35_Cf_Pa   GTAGGTTTAG ATGTTGATAC GCGTGCGTAT TTTGCTGCTG CGAGTATAAT
Dren40_Cf_RG   GTAGGTTTAG ATGTTGATAC GCGTGCGTAT TTTGCTGCTG CGAGTATAAT
Dren41_Cf_RG   GTAGGTTTAG ATGTTGATAC GCGTGCGTAT TTTGCTGCTG CGAGTATAAT
Dren42_Cf_RG   GTAGGTTTAG ATGTTGATAC GCGTGCGTAT TTTGCTGCTG CGAGTATAAT
Dren43_Cf_RG   GTAGGTTTAG ATGTTGATAC GCGTGCGTAT TTTGCTGCTG CGAGTATAAT
Dren45_Cf_RG   GTAGGTTTAG ATGTTGATAC GCGTGCGTAT TTTGCTGCTG CGAGTATAAT
Dren46_Cf_SF   GTAGGTTTAG ATGTTGATAC GCGTGCGTAT TTTGCTGCTG CGAGTATAAT
Dren47_Cb_SF   GTAGGTTTAG ATGTTGATAC GCGTGCGTAT TTTGCTGCTG CGAGTATAAT
Dren48_Cf_BA   GTAGGTTTAG ATGTTGATAC GCGTGCGTAT TTTGCTGCTG CGAGTATAAT
Dren49_Cf_BA   GTAGGTTTAG ATGTTGATAC GCGTGCGTAT TTTGCTGCTG CGAGTATAAT
Dren50_Cf_Ch   GTAGGTTTAG ATGTTGATAC GCGTGCGTAT TTTGCTGCTG CGAGTATAAT
Dren53_Cf_BA   GTAGGTTTAG ATGTTGATAC GCGTGCGTAT TTTGCTGCTG CGAGTATAAT
Dren58_Cb_SF   GTAGGTTTAG ATGTTGATAC GCGTGCGTAT TTTGCTGCTG CGAGTATAAT
Dren66_Cf_SF   GTAGGTTTAG ATGTTGATAC GCGTGCGTAT TTTGCTGCTG CGAGTATAAT
Dren67_Cf_SF   GTAGGTTTAG ATGTTGATAC GCGTGCGTAT TTTGCTGCTG CGAGTATAAT
Dren68_Cf_SF   GTAGGTTTAG ATGTTGATAC GCGTGCGTAT TTTGCTGCTG CGAGTATAAT
Dren69_Cf_SF   GTAGGTTTAG ATGTTGATAC GCGTGCGTAT TTTGCTGCTG CGAGTATAAT
Dren70_Cf_SF   GTAGGTTTAG ATGTTGATAC GCGTGCGTAT TTTGCTGCTG CGAGTATAAT
Dren72_Cb_SF   GTAGGTTTAG ATGTTGATAC GCGTGCGTAT TTTGCTGCTG CGAGTATAAT
Dren73_Cb_Ch   GTAGGTTTAG ATGTTGATAC GCGTGCGTAT TTTGCTGCTG CGAGTATAAT
Dren74_Cb_Co   GTAGGTTTAG ATGTTGATAC GCGTGCGTAT TTTGCTGCTG CGAGTATAAT
Dren75_Cb_Co   GTAGGTTTAG ATGTTGATAC GCGTGCGTAT TTTGCTGCTG CGAGTATAAT
Dren80_Cf_SF   GTAGGTTTAG ATGTTGATAC GCGTGCGTAT TTTGCTGCTG CGAGTATAAT
Dren81_Cf_SF   GTAGGTTTAG ATGTTGATAC GCGTGCGTAT TTTGCTGCTG CGAGTATAAT
Dren82_Cb_Co   GTAGGTTTAG ATGTTGATAC GCGTGCGTAT TTTGCTGCTG CGAGTATAAT
Dren83_Cb_Ch   GTAGGTTTAG ATGTTGATAC GCGTGCGTAT TTTGCTGCTG CGAGTATAAT
Dren84_Cf_SF   GTAGGTTTAG ATGTTGATAC GCGTGCGTAT TTTGCTGCTG CGAGTATAAT
Dren85_Cb_SF   GTAGGTTTAG ATGTTGATAC GCGTGCGTAT TTTGCTGCTG CGAGTATAAT
Dren86_Cb_Co   GTAGGTTTAG ATGTTGATAC GCGTGCGTAT TTTGCTGCTG CGAGTATAAT
Dren87_Cf_BA   GTAGGTTTAG ATGTTGATAC GCGTGCGTAT TTTGCTGCTG CGAGTATAAT
Dren88_Lg_SF   GTAGGTTTAG ATGTTGATAC GCGTGCGTAT TTTGCTGCTG CGAGTATAAT

               ....|....| ....|....| ....|....| ....|....| ....|....|
                  155        165        175        185        195
Dren01_Cf_BA   CATCGGTATT CCGACGGGGG TAAAAGTATT TAGTTGATTA GCTACTTTGT
Dren02_Cf_BA   CATCGGTATT CCGACGGGGG TAAAAGTATT TAGTTGATTA GCTACTTTGT
Dren03_Cf_BA   CATCGGTATT CCGACGGGGG TAAAAGTATT TAGTTGGTTA GCTACTTTGT
Dren04_Cf_BA   CATCGGTATT CCGACGGGGG TAAAAGTATT TAGCTGATTA GCTACTTTGT
Dren05_Cf_BA   CATCGGTATT CCGACAGGGG TAAAAGTATT TAGTTGATTA GCTACTTTGT
Dren06_Cf_BA   CATCGGTATT CCGACGGGGG TAAAAGTATT TAGTTGATTA GCTACTTTGT
Dren07_Cf_BA   CATCGGTATT CCGACAGGGG TAAAAGTATT TAGTTGATTA GCTACTTTGT
Dren08_Cf_BA   CATCGGTATT CCGACGGGGG TAAAAGTATT TAGTTGATTA GCTACTTTGT
Dren09_Cf_BA   CATCGGTATT CCGACGGGGG TAAAAGTATT TAGCTGATTA GCTACTTTGT
Dren10_Cf_BA   CATCGGTATT CCGACGGGGG TAAAAGTATT TAGTTGATTA GCTACTTTGT
Dren11_Cf_BA   CATCGGTATT CCGACGGGGG TAAAAGTATT TAGTTGATTA GCTACTTTGT
Dren18_Cf_BA   CATCGGTATT CCGACAGGGG TAAAAGTATT TAGTTGATTA GCTACTTTGT
Dren19_Cf_BA   CATCGGTATT CCGACAGGGG TAAAAGTATT TAGTTGATTA GCTACTTTGT
Dren23_Cf_BA   CATCGGTATT CCGACGGGGG TAAAAGTATT TAGCTGATTA GCTACTTTGT
Dren24_Cf_BA   CATCGGTATT CCGACGGGGG TAAAAGTATT TAGTTGATTA GCTACTTTGT
Dren25_Cf_SF   CATCGGTATT CCGACGGGGG TAAAAGTATT TAGTTGATTA GCTACTTTGT
Dren28_Fc_Pa   CATCGGTATT CCGACGGGGG TAAAAGTATT TAGTTGATTA GCTACTTTGT
Dren29_Fc_SC   CATCGGTATT CCGACGGGGG TAAAAGTATT TAGTTGATTA GCTACTTTGT
Dren32_Cf_SC   CATCGGTATT CCGACGGGGG TAAAAGTATT TAGTTGATTA GCTACTTTGT
Dren33_Cf_SC   CATCGGTATT CCGACGGGGG TAAAAGTATT TAGTTGATTA GCTACTTTGT
Dren34_Cf_Pa   CATCGGTATT CCGACGGGGG TAAAAGTATT TAGTTGATTA GCTACTTTGT
```

```
Dren35_Cf_Pa   CATCGGTATT CCGACGGGGG TAAAAGTATT TAGTTGATTA GCTACTTTGT
Dren40_Cf_RG   CATCGGTATC CCGACGGGGG TAAAAGTATT TAGTTGATTA GCTACTTTGT
Dren41_Cf_RG   CATCGGTATC CCGACGGGGG TAAAAGTATT TAGTTGATTA GCTACTTTGT
Dren42_Cf_RG   CATCGGTATT CCGACAGGGG TAAAAGTATT TAGTTGATTA GCTACTTTGT
Dren43_Cf_RG   CATCGGTATT CCGACGGGGG TAAAAGTATT TAGTTGATTA GCTACTTTGT
Dren45_Cf_RG   CATCGGTATT CCGACGGGGG TAAAAGTATT TAGTTGATTA GCTACTTTGT
Dren46_Cf_SF   CATCGGTATT CCGACGGGGG TAAAAGTATT TAGTTGATTA GCTACTTTGT
Dren47_Cb_SF   CATCGGTATT CCGACAGGGG TAAAAGTATT TAGTTGATTA GCTACTTTGT
Dren48_Cf_BA   CATCGGTATT CCGACGGGGG TAAAAGTATT TAGTTGATTA GCTACTTTGT
Dren49_Cf_BA   CATCGGTATT CCGACGGGGG TAAAAGTATT TAGCTGATTA GCTACTTTGT
Dren50_Cf_Ch   CATCGGTATT CCGACGGGGG TAAAAGTATT TAGTTGATTA GCTACTTTGT
Dren53_Cf_BA   CATCGGTATT CCGACGGGGG TAAAAGTATT TAGTTGATTA GCTACTTTGT
Dren58_Cb_SF   CATCGGTATC CCGACGGGGG TAAAAGTATT TAGTTGATTA GCTACTTTGT
Dren66_Cf_SF   CATCGGTATT CCGACAGGGG TAAAAGTATT TAGTTGATTA GCTACTTTGT
Dren67_Cf_SF   CATCGGTATT CCGACAGGGG TAAAAGTATT TAGTTGATTA GCTACTTTGT
Dren68_Cf_SF   CATCGGTATT CCGACAGGGG TAAAAGTATT TAGTTGATTA GCTACTTTGT
Dren69_Cf_SF   CATCGGTATT CCGACAGGGG TAAAAGTATT TAGTTGATTA GCTACTTTGT
Dren70_Cf_SF   CATCGGTATT CCGACGGGGG TAAAAGTATT TAGTTGATTA GCTACTTTGT
Dren72_Cb_SF   CATCGGTATT CCGACGGGGG TAAAAGTATT TAGTTGATTA GCTACTTTGT
Dren73_Cb_Ch   CATCGGTATT CCGACAGGGG TAAAAGTATT TAGTTGATTA GCTACTTTGT
Dren74_Cb_Co   CATCGGTATT CCGACGGGGG TAAAAGTATT TAGTTGATTA GCTACTTTGT
Dren75_Cb_Co   CATCGGTATT CCGACGGGGG TAAAAGTATT TAGTTGATTA GCTACTTTGT
Dren80_Cf_SF   CATCGGTATT CCGACGGGGG TAAAAGTATT TAGTTGATTA GCTACTTTGT
Dren81_Cf_SF   CATCGGTATT CCGACGGGGG TAAAAGTATT TAGTTGATTA GCTACTTTGT
Dren82_Cb_Co   CATCGGTATT CCGACGGGGG TAAAAGTATT TAGTTGATTA GCTACTTTGT
Dren83_Cb_Ch   CATCGGTATT CCGACGGGGG TAAAAGTATT TAGTTGGTTA GCTACTTTGT
Dren84_Cf_SF   CATCGGTATT CCGACGGGGG TAAAAGTATT TAGTTGATTA GCTACTTTGT
Dren85_Cb_SF   CATCGGTATT CCGACGGGGG TAAAAGTATT TAGCTGATTA GCTACTTTGT
Dren86_Cb_Co   CATCGGTATT CCGACAGGGG TAAAAGTATT TAGTTGATTA GCTACTTTGT
Dren87_Cf_BA   CATCGGTATC CCGACAGGGG TAAAAGTATT TAGTTGATTA GCTACTTTGT
Dren88_Lg_SF   CATCGGTATT CCGACAGGGG TAAAAGTATT TAGTTGATTA GCTACTTTGT

               ....|....| ....|....| ....|....| ....|....| ....|....|
                    205        215        225        235        245
Dren01_Cf_BA   ATGGTGGTCA GGTTATCTTT TCACCTTTGT ATTGTTGGAC TGTAGGGTTT
Dren02_Cf_BA   ATGGTGGTCA GGTTATCTTT TCACCTTTGT ATTGTTGGAC TGTAGGGTTT
Dren03_Cf_BA   ATGGTGGTCA GGTTATCTTT TCACCTTTGT ATTGTTGGAC TGTAGGGTTT
Dren04_Cf_BA   ATGGTGGTCA GGTTATCTTT TCACCTTTGT ATTGTTGGAC TGTAGGGTTT
Dren05_Cf_BA   ATGGTGGTCA GGTTATCTTT TCACCTTTGT ATTGTTGGAC TGTAGGGTTT
Dren06_Cf_BA   ATGGTGGTCA GGTTATCTTT TCACCTTTGT ATTGTTGGAC TGTAGGGTTT
Dren07_Cf_BA   ATGGTGGTCA GGTTATCTTT TCACCTTTGT ATTGTTGGAC TGTAGGGTTT
Dren08_Cf_BA   ATGGTGGTCA GGTTATCTTT TCACCTTTGT ATTGTTGGAC TGTAGGGTTT
Dren09_Cf_BA   ATGGTGGTCA GGTTATCTTT TCACCTTTGT ATTGTTGGAC TGTAGGGTTT
Dren10_Cf_BA   ATGGTGGTCA GGTTATCTTT TCACCTTTGT ATTGTTGGAC TGTAGGGTTT
Dren11_Cf_BA   ATGGTGGTCA GGTTATCTTT TCACCTTTGT ATTGTTGGAC TGTAGGGTTT
Dren18_Cf_BA   ATGGTGGTCA GGTTATCTTT TCACCTTTGT ATTGTTGGAC TGTAGGGTTT
Dren19_Cf_BA   ATGGTGGTCA GGTTATCTTT TCACCTTTGT ATTGTTGGAC TGTAGGGTTT
Dren23_Cf_BA   ATGGTGGTCA GGTTATCTTT TCACCTTTGT ATTGTTGGAC TGTAGGGTTT
Dren24_Cf_BA   ATGGTGGTCA GGTTATCTTT TCACCTTTGT ATTGTTGGAC TGTAGGGTTT
Dren25_Cf_SF   ATGGTGGTCA GGTTATCTTT TCACCTTTGT ATTGTTGGAC TGTAGGGTTT
Dren28_Fc_Pa   ATGGTGGTCA GGTTATCTTT TCACCTTTGT ATTGTTGGAC TGTAGGGTTT
Dren29_Fc_SC   ATGGTGGTCA GGTTATCTTT TCACCTTTGT ATTGTTGGAC TGTAGGGTTT
Dren32_Cf_SC   ATGGTGGTCA GGTTATCTTT TCACCTTTGT ATTGTTGGAC TGTAGGGTTT
Dren33_Cf_SC   ATGGTGGTCA GGTTATCTTT TCACCTTTGT ATTGTTGGAC TGTAGGGTTT
Dren34_Cf_Pa   ATGGTGGTCA GGTTATCTTT TCACCTTTGT ATTGTTGGAC TGTAGGGTTT
Dren35_Cf_Pa   ATGGTGGTCA GGTTATCTTT TCACCTTTGT ATTGTTGGAC TGTAGGGTTT
Dren40_Cf_RG   ATGGTGGTCA GGTTATCTTT TCACCTTTGT ATTGTTGGAC TGTAGGGTTT
Dren41_Cf_RG   ATGGTGGTCA GGTTATCTTT TCACCTTTGT ATTGTTGGAC TGTAGGGTTT
Dren42_Cf_RG   ATGGTGGTCA GGTTATCTTT TCACCTTTGT ATTGTTGGAC TGTAGGGTTT
Dren43_Cf_RG   ATGGTGGTCA GGTTATCTTT TCACCCTTGT ATTGTTGGAC TGTAGGGTTT
Dren45_Cf_RG   ATGGTGGTCA GGTTATCTTT TCACCCTTGT ATTGTTGGAC TGTAGGGTTT
Dren46_Cf_SF   ATGGTGGTCA GGTTATCTTT TCACCTTTGT ATTGTTGGAC TGTAGGGTTT
Dren47_Cb_SF   ATGGTGGTCA GGTTATCTTT TCACCTTTGT ATTGTTGGAC TGTAGGGTTT
Dren48_Cf_BA   ATGGTGGTCA GGTTATCTTT TCACCTTTGT ATTGTTGGAC TGTAGGGTTT
```

```
Dren49_Cf_BA   ATGGTGGTCA GGTTATCTTT TCACCTTTGT ATTGTTGGAC TGTAGGGTTT
Dren50_Cf_Ch   ATGGTGGTCA GGTTATCTTT TCACCTTTGT ATTGTTGGAC TGTAGGGTTT
Dren53_Cf_BA   ATGGTGGTCA GGTTATCTTT TCACCTTTGT ATTGTTGGAC TGTAGGGTTT
Dren58_Cb_SF   ATGGTGGTCA GGTTATCTTT TCACCTTTGT ATTGTTGGAC TGTAGGGTTT
Dren66_Cf_SF   ATGGTGGTCA GGTTATCTTT TCACCTTTGT ATTGTTGGAC TGTAGGGTTT
Dren67_Cf_SF   ATGGTGGTCA GGTTATCTTT TCACCTTTGT ATTGTTGGAC TGTAGGGTTT
Dren68_Cf_SF   ATGGTGGTCA GGTTATCTTT TCACCTTTGT ATTGTTGGAC TGTAGGGTTT
Dren69_Cf_SF   ATGGTGGTCA GGTTATCTTT TCACCTTTGT ATTGTTGGAC TGTAGGGTTT
Dren70_Cf_SF   ATGGTGGTCA GGTTATCTTT TCACCTTTGT ATTGTTGGAC TGTAGGGTTT
Dren72_Cb_SF   ATGGTGGTCA GGTTATCTTT TCACCTTTGT ATTGTTGGAC TGTAGGGTTT
Dren73_Cb_Ch   ATGGTGGTCA GGTTATCTTT TCACCTTTGT ATTGTTGGAC TGTAGGGTTT
Dren74_Cb_Co   ATGGTGGTCA GGTTATCTTT TCACCTTTGT ATTGTTGGAC TGTAGGGTTT
Dren75_Cb_Co   ATGGTGGTCA GGTTATCTTT TCACCTTTGT ATTGTTGGAC TGTAGGGTTT
Dren80_Cf_SF   ATGGTGGTCA GGTTATCTTT TCACCTTTGT ATTGTTGGAC TGTAGGGTTT
Dren81_Cf_SF   ATGGTGGTCA GGTTATCTTT TCACCTTTGT ATTGTTGGAC TGTAGGGTTT
Dren82_Cb_Co   ATGGTGGTCA GGTTATCTTT TCACCTTTGT ATTGTTGGAC TGTAGGGTTT
Dren83_Cb_Ch   ATGGTGGTCA GGTTATCTTT TCACCTTTGT ATTGTTGGAC TGTAGGGTTT
Dren84_Cf_SF   ATGGTGGTCA GGTTATCTTT TCACCTTTGT ATTGTTGGAC TGTAGGGTTT
Dren85_Cb_SF   ATGGTGGTCA GGTTATCTTT TCACCTTTGT ATTGTTGGAC TGTAGGGTTT
Dren86_Cb_Co   ATGGTGGTCA GGTTATCTTT TCACCTTTGT ATTGTTGGAC TGTAGGGTTT
Dren87_Cf_BA   ATGGTGGTCA GGTTATCTTT TCACCTTTGT ATTGTTGGAC TGTAGGGTTT
Dren88_Lg_SF   ATGGTGGTCA GGTTATCTTT TCACCTTTGT ATTGTTGGAC TGTAGGGTTT

               ....|....
                  255
Dren01_Cf_BA   ATTTCTGTG
Dren02_Cf_BA   ATTTCTGTG
Dren03_Cf_BA   ATTTCTGTG
Dren04_Cf_BA   ATTTCTGTG
Dren05_Cf_BA   ATTTCTGTG
Dren06_Cf_BA   ATTTCTGTG
Dren07_Cf_BA   ATTTCTGTG
Dren08_Cf_BA   ATTTCTGTG
Dren09_Cf_BA   ATTTCTGTG
Dren10_Cf_BA   ATTTCTGTG
Dren11_Cf_BA   ATTTCTGTG
Dren18_Cf_BA   ATTTCTGTG
Dren19_Cf_BA   ATTTCTGTG
Dren23_Cf_BA   ATTTCTGTG
Dren24_Cf_BA   ATTTCTGTG
Dren25_Cf_SF   ATTTCTGTG
Dren28_Fc_Pa   ATTTCTGTG
Dren29_Fc_SC   ATTTCTGTG
Dren32_Cf_SC   ATTTCTGTG
Dren33_Cf_SC   ATTTCTGTG
Dren34_Cf_Pa   ATTTCTGTG
Dren35_Cf_Pa   ATTTCTGTG
Dren40_Cf_RG   ATTTCTGTG
Dren41_Cf_RG   ATTTCTGTG
Dren42_Cf_RG   ATTTCTGTG
Dren43_Cf_RG   ATTTCTGTG
Dren45_Cf_RG   ATTTCTGTG
Dren46_Cf_SF   ATTTCTGTG
Dren47_Cb_SF   ATTTCTGTG
Dren48_Cf_BA   ATTTCTGTG
Dren49_Cf_BA   ATTTCTGTG
Dren50_Cf_Ch   ATTTCTGTG
Dren53_Cf_BA   ATTTCTGTG
Dren58_Cb_SF   ATTTCTGTG
Dren66_Cf_SF   ATTTCTGTG
Dren67_Cf_SF   ATTTCTGTG
Dren68_Cf_SF   ATTTCTGTG
Dren69_Cf_SF   ATTTCTGTG
Dren70_Cf_SF   ATTTCTGTG
```

```
Dren72_Cb_SF   ATTTCTGTG
Dren73_Cb_Ch   ATTTCTGTG
Dren74_Cb_Co   ATTTCTGTG
Dren75_Cb_Co   ATTTCTGTG
Dren80_Cf_SF   ATTTCTGTG
Dren81_Cf_SF   ATTTCTGTG
Dren82_Cb_Co   ATTTCTGTG
Dren83_Cb_Ch   ATTTCTGTG
Dren84_Cf_SF   ATTTCTGTG
Dren85_Cb_SF   ATTTCTGTG
Dren86_Cb_Co   ATTTCTGTG
Dren87_Cf_BA   ATTTCTGTG
Dren88_Lg_SF   ATTTCTGTG
```

COX1-L Multiple Sequence Alignment

```
               ....|....| ....|....| ....|....| ....|....| ....|....|
                   5         15         25         35         45
Dren01_Cf_BA   GGTGTGTGAG CAGGGTTTGT TGGGTTGGGG TTATCAGTTA TTATTCGGTA
Dren02_Cf_BA   GGTGTGTGAG CAGGGTTTGT TGGGTTGGGG TTATCAGTTA TTATTCGGTA
Dren03_Cf_BA   GGTGTGTGAG CAGGGTTTGT TGGGTTGGGG TTATCAGTTA TTATTCGGTA
Dren05_Cf_BA   GGTGTGTGAG CAGGGTTTGT TGGGTTGGGG TTATCAGTTA TTATTCGGTA
Dren06_Cf_BA   GGTGTGTGAG CAGGGTTTGT TGGGTTGGGG TTATCAGTTA TTATTCGGTA
Dren07_Cf_BA   GGTGTGTGAG CAGGGTTTGT TGGGTTGGGG TTATCAGTTA TTATTCGGTA
Dren08_Cf_BA   GGTGTGTGAG CAGGGTTTGT TGGGTTGGGG TTATCAGTTA TTATTCGGTA
Dren09_Cf_BA   GGTGTGTGAG CAGGGTTTGT TGGGTTGGGG TTATCAGTTA TTATTCGGTA
Dren10_Cf_BA   GGTGTGTGAG CAGGGTTTGT TGGGTTGGGG TTATCAGTTA TTATTCGGTA
Dren11_Cf_BA   GGTGTGTGAG CAGGGTTTGT TGGGTTGGGG TTATCAGTTA TTATTCGGTA
Dren18_Cf_BA   GGTGTGTGAG CAGGGTTTGT TGGGTTGGGG TTATCAGTTA TTATTCGGTA
Dren19_Cf_BA   GGTGTGTGAG CAGGGTTTGT TGGGTTGGGG TTATCAGTTA TTATTCGGTA
Dren23_Cf_BA   GGTGTGTGAG CAGGGTTTGT TGGGTTGGGG TTATCAGTTA TTATTCGGTA
Dren25_Cf_SF   GGTGTGTGAG CAGGGTTTGT TGGGTTGGGG TTATCAGTTA TTATTCGGTA
Dren32_Cf_SC   GGTGTGTGAG CAGGGTTTGT TGGGTTGGGG TTATCAGTTA TTATTCGGTA
Dren33_Cf_SC   GGTGTGTGAG CAGGGTTTGT TGGGTTGGGG TTATCAGTTA TTATTCGGTA
Dren34_Cf_Pa   GGTGTGTGAG CAGGGTTTGT TGGGTTGGGG TTATCAGTTA TTATTCGGTA
Dren35_Cf_Pa   GGTGTGTGAG CAGGGTTTGT TGGGTTGGGG TTATCAGTTA TTATTCGGTA
Dren36_Cf_SC   GGTGTGTGAG CAGGGTTTGT TGGGTTGGGG TTATCAGTTA TTATTCGGTA
Dren40_Cf_RG   GGTGTGTGAG CAGGGTTTGT TGGGTTGGGG TTATCAGTTA TTATTCGGTA
Dren41_Cf_RG   GGTGTGTGAG CAGGGTTTGT TGGGTTGGGG TTATCAGTTA TTATTCGGTA
Dren42_Cf_RG   GGTGTGTGAG CAGGGTTTGT TGGGTTGGGG TTATCAGTTA TTATTCGGTA
Dren43_Cf_RG   GGTGTGTGAG CAGGGTTTGT TGGGTTGGGG TTATCAGTTA TTATTCGGTA
Dren45_Cf_RG   GGTGTGTGAG CAGGGTTTGT TGGGTTGGGG TTATCAGTTA TTATTCGGTA
Dren46_Cf_SF   GGTGTGTGAG CAGGGTTTGT TGGGTTGGGG TTATCAGTTA TTATTCGGTA
Dren47_Cb_SF   GGTGTGTGAG CAGGGTTTGT TGGGTTGGGG TTATCAGTTA TTATTCGGTA
Dren48_Cf_BA   GGTGTGTGAG CAGGGTTTGT TGGGTTGGGG TTATCAGTTA TTATTCGGTA
Dren49_Cf_BA   GGTGTGTGAG CAGGGTTTGT TGGGTTGGGG TTATCAGTTA TTATTCGGTA
Dren50_Cf_Ch   GGTGTGTGAG CAGGGTTTGT TGGGTTGGGG TTATCAGTTA TTATTCGGTA
Dren53_Cf_BA   GGTGTGTGAG CAGGGTTTGT TGGGTTGGGG TTATCAGTTA TTATTCGGTA
Dren58_Cb_SF   GGTGTGTGAG CAGGGTTTGT TGGGTTGGGG TTATCAGTTA TTATTCGGTA
Dren66_Cf_SF   GGTGTGTGAG CAGGGTTTGT TGGGTTGGGG TTATCAGTTA TTATTCGGTA
Dren67_Cf_SF   GGTGTGTGAG CAGGGTTTGT TGGGTTGGGG TTATCAGTTA TTATTCGGTA
Dren68_Cf_SF   GGTGTGTGAG CAGGGTTTGT TGGGTTGGGG TTATCAGTTA TTATTCGGTA
Dren69_Cf_SF   GGTGTGTGAG CAGGGTTTGT TGGGTTGGGG TTATCAGTTA TTATTCGGTA
Dren70_Cf_SF   GGTGTGTGAG CAGGGTTTGT TGGGTTGGGG TTATCAGTTA TTATTCGGTA
Dren72_Cb_SF   GGTGTGTGAG CAGGGTTTGT TGGGTTGGGG TTATCAGTTA TTATTCGGTA
Dren73_Cb_Ch   GGTGTGTGAG CAGGGTTTGT TGGGTTGGGG TTATCAGTTA TTATTCGGTA
Dren74_Cb_Co   GGTGTGTGAG CAGGGTTTGT TGGGTTGGGG TTATCAGTTA TTATTCGGTA
Dren75_Cb_Co   GGTGTGTGAG CAGGGTTTGT TGGGTTGGGG TTATCAGTTA TTATTCGGTA
Dren80_Cf_SF   GGTGTGTGAG CAGGGTTTGT TGGGTTGGGG TTATCAGTTA TTATTCGGTA
Dren81_Cf_SF   GGTGTGTGAG CAGGGTTTGT TGGGTTGGGG TTATCAGTTA TTATTCGGTA
Dren82_Cb_Co   GGTGTGTGAG CAGGGTTTGT TGGGTTGGGG TTATCAGTTA TTATTCGGTA
Dren83_Cb_Ch   GGTGTGTGAG CAGGGTTTGT TGGGTTGGGG TTATCAGTTA TTATTCGGTA
Dren84_Cf_SF   GGTGTGTGAG CAGGGTTTGT TGGGTTGGGG TTATCAGTTA TTATTCGGTA
Dren85_Cb_SF   GGTGTGTGAG CAGGGTTTGT TGGGTTGGGG TTATCAGTTA TTATTCGGTA
Dren86_Cb_Co   GGTGTGTGAG CAGGGTTTGT TGGGTTGGGG TTATCAGTTA TTATTCGGTA
Dren87_Cf_BA   GGTGTGTGAG CAGGGTTTGT TGGGTTGGGG TTATCAGTTA TTATTCGGTA
Dren88_Lg_SF   GGTGTGTGAG CAGGGTTTGT TGGGTTGGGG TTATCAGTTA TTATTCGGTA
```

```
                    ....|....| ....|....| ....|....| ....|....| ....|....|
                           55         65         75         85         95
Dren01_Cf_BA        TGTGTTGGCA TCACCGTGGT CTATAGGGTG AAGGCCTTTT ATTAGGCAGT
Dren02_Cf_BA        TGTGTTGGCA TCACCGTGGT CTATAGGGTG AAGGCCTTTT ATTAGGCAGT
Dren03_Cf_BA        TGTGTTGGCA TCACCGTGGT CTATAGGGTG AAGGCCTTTT ATTAGGCAGT
Dren05_Cf_BA        TGTGTTGGCA TCACCGTGGT CTATAGGGTG AAGGCCTTTT ATTAGGCAGT
Dren06_Cf_BA        TGTGTTGGCA TCACCGTGGT CTATAGGGTG AAGGCCTTTT ATTAGGCAGT
Dren07_Cf_BA        TGTGTTGGCA TCACCGTGGT CTATAGGGTG AAGGCCTTTT ATTAGGCAGT
Dren08_Cf_BA        TGTGTTGGCA TCACCGTGGT CTATAGGGTG AAGGCCTTTT ATTAGGCAGT
Dren09_Cf_BA        TGTGTTGGCA TCACCGTGGT CTATAGGGTG AAGGCCTTTT ATTAGGCAGT
Dren10_Cf_BA        TGTGTTGGCA TCACCGTGGT CTATAGGGTG AAGGCCTTTT ATTAGGCAGT
Dren11_Cf_BA        TGTGTTGGCA TCACCGTGGT CTATAGGGTG AAGGCCTTTT ATTAGGCAGT
Dren18_Cf_BA        TGTGTTGGCA TCACCGTGGT CTATAGGGTG AAGGCCTTTT ATTAGGCAGT
Dren19_Cf_BA        TGTGTTGGCA TCACCGTGGT CTATAGGGTG AAGGCCTTTT ATTAGGCAGT
Dren23_Cf_BA        TGTGTTGGCA TCACCGTGGT CTATAGGGTG AAGGCCTTTT ATTAGGCAGT
Dren25_Cf_SF        TGTGTTGGCA TCACCGTGGT CTATAGGGTG AAGGCCTTTT ATTAGGCAGT
Dren32_Cf_SC        TGTGTTGGCA TCACCGTGGT CTATAGGGTG AAGGCCTTTT ATTAGGCAGT
Dren33_Cf_SC        TGTGTTGGCA TCACCGTGGT CTATAGGGTG AAGGCCTTTT ATTAGGCAGT
Dren34_Cf_Pa        TGTGTTGGCA TCACCGTGGT CTATAGGGTG AAGGCCTTTT ATTAGGCAGT
Dren35_Cf_Pa        TGTGTTGGCA TCACCGTGGT CTATAGGGTG AAGGCCTTTT ATTAGGCAGT
Dren36_Cf_SC        TGTGTTGGCA TCACCGTGGT CTATAGGGTG AAGGCCTTTT ATTAGGCAGT
Dren40_Cf_RG        CGTGTTGGCA TCACCATGGT CTATAGGGTG AAGGCCTTTT ATTAGGCAGT
Dren41_Cf_RG        CGTGTTGGCA TCACCATGGT CTATAGGGTG AAGGCCTTTT ATTAGGCAGT
Dren42_Cf_RG        TGTGTTGGCA TCACCGTGGT CTATAGGGTG AAGGCCTTTT ATTAGGCAGT
Dren43_Cf_RG        TGTGTTGGCA TCACCGTGGT CTATAGGGTG AAGGCCTTTT ATTAGGCAGT
Dren45_Cf_RG        TGTGTTGGCA TCACCGTGGT CTATAGGGTG AAGGCCTTTT ATTAGGCAGT
Dren46_Cf_SF        TGTGTTGGCA TCACCGTGGT CTATAGGGTG AAGGCCTTTT ATTAGGCAGT
Dren47_Cb_SF        TGTGTTGGCA TCACCGTGGT CTATAGGGTG AAGGCCTTTT ATTAGGCAGT
Dren48_Cf_BA        TGTGTTGGCA TCACCGTGGT CTATAGGGTG AAGGCCTTTT ATTAGGCAGT
Dren49_Cf_BA        TGTGTTGGCA TCACCGTGGT CTATAGGGTG AAGGCCTTTT ATTAGGCAGT
Dren50_Cf_Ch        TGTGTTGGCA TCACCGTGGT CTATAGGGTG AAGGCCTTTT ATTAGGCAGT
Dren53_Cf_BA        TGTGTTGGCA TCACCGTGGT CTATAGGGTG AAGGCCTTTT ATTAGGCAGT
Dren58_Cb_SF        CGTGTTGGCA TCACCATGGT CTATAGGGTG AAGGCCTTTT ATTAGGCAGT
Dren66_Cf_SF        TGTGTTGGCA TCACCGTGGT CTATAGGGTG AAGGCCTTTT ATTAGGCAGT
Dren67_Cf_SF        TGTGTTGGCA TCACCGTGGT CTATAGGGTG AAGGCCTTTT ATTAGGCAGT
Dren68_Cf_SF        TGTGTTGGCA TCACCGTGGT CTATAGGGTG AAGGCCTTTT ATTAGGCAGT
Dren69_Cf_SF        TGTGTTGGCA TCACCGTGGT CTATAGGGTG AAGGCCTTTT ATTAGGCAGT
Dren70_Cf_SF        TGTGTTGGCA TCACCGTGGT CTATAGGGTG AAGGCCTTTT ATTAGGCAGT
Dren72_Cb_SF        TGTGTTGGCA TCACCGTGGT CTATAGGGTG AAGGCCTTTT ATTAGGCAGT
Dren73_Cb_Ch        TGTGTTGGCA TCACCGTGGT CTATAGGGTG AAGGCCTTTT ATTAGGCAGT
Dren74_Cb_Co        TGTGTTGGCA TCACCGTGGT CTATAGGGTG AAGGCCTTTT ATTAGGCAGT
Dren75_Cb_Co        TGTGTTGGCA TCACCGTGGT CTATAGGGTG AAGGCCTTTT ATTAGGCAGT
Dren80_Cf_SF        TGTGTTGGCA TCACCGTGGT CTATAGGGTG AAGGCCTTTT ATTAGGCAGT
Dren81_Cf_SF        TGTGTTGGCA TCACCGTGGT CTATAGGGTG AAGGCCTTTT ATTAGGCAGT
Dren82_Cb_Co        TGTGTTGGCA TCACCGTGGT CTATAGGGTG AAGGCCTTTT ATTAGGCAGT
Dren83_Cb_Ch        TGTGTTGGCA TCACCGTGGT CTATAGGGTG AAGGCCTTTT ATTAGGCAGT
Dren84_Cf_SF        TGTGTTGGCA TCACCGTGGT CTATAGGGTG AAGGCCTTTT ATTAGGCAGT
Dren85_Cb_SF        TGTGTTGGCA TCACCGTGGT CTATAGGGTG AAGGCCTTTT ATTAGGCAGT
Dren86_Cb_Co        TGTGTTGGCA TCACCGTGGT CTATAGGGTG AAGGCCTTTT ATTAGGCAGT
Dren87_Cf_BA        TGTGTTGGCA TCACCGTGGT CTATAGGGTG AAGGCCTTTT ATTAGGCAGT
Dren88_Lg_SF        TGTGTTGGCA TCACCGTGGT CTATAGGGTG AAGGCCTTTT ATTAGGCAGT

                    ....|....| ....|....| ....|....| ....|....| ....|....|
```

```
                     105        115        125        135        145
Dren01_Cf_BA   ATTATAATGC TATTATTTCC GCGCATGGCA TTATTATAAT CTTTTATATA
Dren02_Cf_BA   ATTATAATGC TATTATTTCC GCGCATGGCA TTATTATAAT CTTTTATATA
Dren03_Cf_BA   ATTATAATGC TATTATTTCC GCGCATGGCA TTATTATAAT CTTTTATATA
Dren05_Cf_BA   ATTATAATGC TATTATTTCC GCGCATGGCA TTATTATAAT CTTTTATATA
Dren06_Cf_BA   ATTATAATGC TATTATTTCC GCGCATGGCA TTATTATAAT CTTTTATATA
Dren07_Cf_BA   ATTATAATGC TATTATTTCC GCGCACGGCA TTATTATAAT CTTTTATATA
Dren08_Cf_BA   ATTATAATGC TATTATTTCC GCGCATGGCA TTATTATAAT CTTTTATATA
Dren09_Cf_BA   ATTATAATGC TATTATTTCC GCGCATGGCA TTATTATAAT CTTTTATATA
Dren10_Cf_BA   ATTATAATGC TATTATTTCC GCGCATGGCA TTATTATAAT CTTTTATATA
Dren11_Cf_BA   ATTATAATGC TATTATTTCC GCGCATGGCA TTATTATAAT CTTTTATATA
Dren18_Cf_BA   ATTATAATGC TATTATTTCC GCGCATGGCA TTATTATAAT CTTTTATATA
Dren19_Cf_BA   ATTATAATGC TATTATTTCC GCGCATGGCA TTATTATAAT CTTTTATATA
Dren23_Cf_BA   ATTATAATGC TATTATTTCC GCGCATGGCA TTATTATAAT CTTTTATATA
Dren25_Cf_SF   ATTATAATGC TATTATTTCC GCGCATGGCA TTATTATAAT CTTTTATATA
Dren32_Cf_SC   ATTATAATGC TATTATTTCC GCGCATGGCA TTATTATAAT CTTTTATATA
Dren33_Cf_SC   ATTATAATGC TATTATTTCC GCGCATGGCA TTATTATAAT CTTTTATATA
Dren34_Cf_Pa   ATTATAATGC TATTATTTCC GCGCATGGCA TTATTATAAT CTTTTATATA
Dren35_Cf_Pa   ATTATAATGC TATTATTTCC GCGCATGGCA TTATTATAAT CTTTTATATA
Dren36_Cf_SC   ATTATAATGC TATTATTTCC GCGCATGGCA TTATTATAAT CTTTTATATA
Dren40_Cf_RG   ATTATAATGC TATTATTTCC GCGCATGGCA TTATTATAAT CTTTTATATA
Dren41_Cf_RG   ATTATAATGC TATTATTTCC GCGCATGGCA TTATTATAAT CTTTTATATA
Dren42_Cf_RG   ATTATAATGC TATTATTTCC GCGCACGGCA TTATTATAAT CTTTTATATA
Dren43_Cf_RG   ATTATAATGC TATTATTTCC GCGCATGGCA TTATTATAAT CTTTTATATA
Dren45_Cf_RG   ATTATAATGC TATTATTTCC GCGCATGGCA TTATTATAAT CTTTTATATA
Dren46_Cf_SF   ATTATAATGC TATTATTTCC GCGCATGGCA TTATTATAAT CTTTTATATA
Dren47_Cb_SF   ATTATAATGC TATTATTTCC GCGCATGGCA TTATTATAAT CTTTTATATA
Dren48_Cf_BA   ATTATAATGC TATTATTTCC GCGCATGGCA TTATTATAAT CTTTTATATA
Dren49_Cf_BA   ATTATAATGC TATTATTTCC GCGCATGGCA TTATTATAAT CTTTTATATA
Dren50_Cf_Ch   ATTATAATGC TATTATTTCC GCGCATGGCA TTATTATAAT CTTTTATATA
Dren53_Cf_BA   ATTATAATGC TATTATTTCC GCGCATGGCA TTATTATAAT CTTTTATATA
Dren58_Cb_SF   ATTATAATGC TATTATTTCC GCGCATGGCA TTATTATAAT CTTTTATATA
Dren66_Cf_SF   ATTATAATGC TATTATTTCC GCGCATGGCA TTATTATAAT CTTTTATATA
Dren67_Cf_SF   ATTATAATGC TATTATTTCC GCGCATGGCA TTATTATAAT CTTTTATATA
Dren68_Cf_SF   ATTATAATGC TATTATTTCC GCGCATGGCA TTATTATAAT CTTTTATATA
Dren69_Cf_SF   ATTATAATGC TATTATTTCC GCGCATGGCA TTATTATAAT CTTTTATATA
Dren70_Cf_SF   ATTATAATGC TATTATTTCC GCGCATGGCA TTATTATAAT CTTTTATATA
Dren72_Cb_SF   ATTATAATGC TATTATTTCC GCGCATGGCA TTATTATAAT CTTTTATATA
Dren73_Cb_Ch   ATTATAATGC TATTATTTCC GCGCATGGCA TTATTATAAT CTTTTATATA
Dren74_Cb_Co   ATTATAATGC TATTATTTCC GCGCATGGCA TTATTATAAT CTTTTATATA
Dren75_Cb_Co   ATTATAATGC TATTATTTCC GCGCATGGCA TTATTATAAT CTTTTATATA
Dren80_Cf_SF   ATTATAATGC TATTATTTCC GCGCATGGCA TTATTATAAT CTTTTATATA
Dren81_Cf_SF   ATTATAATGC TATTATTTCC GCGCATGGCA TTATTATAAT CTTTTATATA
Dren82_Cb_Co   ATTATAATGC TATTATTTCC GCGCATGGCA TTATTATAAT CTTTTATATA
Dren83_Cb_Ch   ATTATAATGC TATTATTTCC GCGCATGGCA TTATTATAAT CTTTTATATA
Dren84_Cf_SF   ATTATAATGC TATTATTTCC GCGCATGGCA TTATTATAAT CTTTTATATA
Dren85_Cb_SF   ATTATAATGC TATTATTTCC GCGCATGGCA TTATTATAAT CTTTTATATA
Dren86_Cb_Co   ATTATAATGC TATTATTTCC GCGCACGGCA TTATTATAAT CTTTTATATA
Dren87_Cf_BA   ATTATAATGC TATTATTTCC GCGCATGGCA TTATTATAAT CTTTTATATA
Dren88_Lg_SF   ATTATAATGC TATTATTTCC GCGCATGGCA TTATTATAAT CTTTTATATA

               ....|....| ....|....| ....|....| ....|....| ....|....|
                     155        165        175        185        195
Dren01_Cf_BA   ATGATGCCTA TTATAATAGG TGGGATGGGT AATATTATAG TGCCTGTTAT
```

```
Dren02_Cf_BA   ATGATGCCTA TTATAATAGG TGGGATGGGT AATATTATAG TGCCTGTTAT
Dren03_Cf_BA   ATGATGCCTA TTATAATAGG TGGGATGGGT AATATTATAG TGCCTGTTAT
Dren05_Cf_BA   ATAATGCCTA TTATAATAGG TGGGATGGGT AATATTATAG TGCCTGTTAT
Dren06_Cf_BA   ATGATGCCTA TTATAATAGG TGGGATGGGT AATATTATAG TGCCTGTTAT
Dren07_Cf_BA   ATGATGCCTA TTATAATAGG TGGGATGGGT AATATTATAG TGCCTGTTAT
Dren08_Cf_BA   ATGATGCCTA TTATAATAGG TGGGATGGGT AATATTATAG TGCCTGTTAT
Dren09_Cf_BA   ATGATGCCTA TTATAATAGG TGGGATGGGT AATATTATAG TGCCTGTTAT
Dren10_Cf_BA   ATGATGCCTA TTATAATAGG TGGGATGGGT AATATTATAG TGCCTGTTAT
Dren11_Cf_BA   ATGATGCCTA TTATAATAGG TGGGATGGGT AATATTATAG TGCCTGTTAT
Dren18_Cf_BA   ATGATGCCTA TTATAATAGG TGGGATGGGT AATATTATAG TGCCTGTTAT
Dren19_Cf_BA   ATGATGCCTA TTATAATAGG TGGGATGGGT AATATTATAG TGCCTGTTAT
Dren23_Cf_BA   ATGATGCCTA TTATAATAGG TGGGGTGGGT AATATTATAG TGCCTGTTAT
Dren25_Cf_SF   ATGATGCCTA TTATAATAGG TGGGATGGGT AATATTATAG TGCCTGTTAT
Dren32_Cf_SC   ATGATGCCTA TTATAATAGG TGGGATGGGT AATATTATAG TGCCTGTTAT
Dren33_Cf_SC   ATGATGCCTA TTATAATAGG TGGGATGGGT AATATTATAG TGCCTGTTAT
Dren34_Cf_Pa   ATGATGCCTA TTATAATAGG TGGGATGGGT AATATTATAG TGCCTGTTAT
Dren35_Cf_Pa   ATGATGCCTA TTATAATAGG TGGGATGGGT AATATTATAG TGCCTGTTAT
Dren36_Cf_SC   ATGATGCCTA TTATAATAGG TGGGATGGGT AATATTATAG TGCCTGTTAT
Dren40_Cf_RG   ATGATGCCTA TTATAATAGG TGGGATGGGT AATATTATAG TGCCTGTTAT
Dren41_Cf_RG   ATGATGCCTA TTATAATAGG TGGGATGGGT AATATTATAG TGCCTGTTAT
Dren42_Cf_RG   ATGATGCCTA TTATAATAGG TGGGATGGGT AATATTATAG TGCCTGTTAT
Dren43_Cf_RG   ATGATGCCTA TTATAATAGG TGGGATGGGT AATATTATAG TGCCTGTTAT
Dren45_Cf_RG   ATGATGCCTA TTATAATAGG TGGGATGGGT AATATTATAG TGCCTGTTAT
Dren46_Cf_SF   ATGATGCCTA TTATAATAGG TGGGATGGGT AATATTATAG TGCCTGTTAT
Dren47_Cb_SF   ATAATGCCTA TTATAATAGG TGGGATGGGT AATATTATAG TGCCTGTTAT
Dren48_Cf_BA   ATGATGCCTA TTATAATAGG TGGGATGGGT AATATTATAG TGCCTGTTAT
Dren49_Cf_BA   ATGATGCCTA TTATAATAGG TGGGGTGGGT AATATTATAG TGCCTGTTAT
Dren50_Cf_Ch   ATGATGCCTA TTATAATAGG TGGGATGGGT AATATTATAG TGCCTGTTAT
Dren53_Cf_BA   ATGATGCCTA TTATAATAGG TGGGATGGGT AATATTATAG TGCCTGTTAT
Dren58_Cb_SF   ATGATGCCTA TTATAATAGG TGGGGTGGGT AATGTTATAG TGCCTGTTAT
Dren66_Cf_SF   ATGATGCCTA TTATAATAGG TGGGATGGGT AATATTATAG TGCCTGTTAT
Dren67_Cf_SF   ATAATGCCTA TTATAATAGG TGGGATGGGT AATATTATAG TGCCTGTTAT
Dren68_Cf_SF   ATAATGCCTA TTATAATAGG TGGGATGGGT AATATTATAG TGCCTGTTAT
Dren69_Cf_SF   ATAATGCCTA TTATAATAGG TGGGATGGGT AATATTATAG TGCCTGTTAT
Dren70_Cf_SF   ATGATGCCTA TTATAATAGG TGGGATGGGT AATATTATAG TGCCTGTTAT
Dren72_Cb_SF   ATGATGCCTA TTATAATAGG TGGGATGGGT AATATTATAG TGCCTGTTAT
Dren73_Cb_Ch   ATAATGCCTA TTATAATAGG TGGGATGGGT AATATTATAG TGCCTGTTAT
Dren74_Cb_Co   ATGATGCCTA TTATAATAGG TGGGATGGGT AATATTATAG TGCCTGTTAT
Dren75_Cb_Co   ATGATGCCTA TTATAATAGG TGGGATGGGT AATATTATAG TGCCTGTTAT
Dren80_Cf_SF   ATGATGCCTA TTATAATAGG TGGGATGGGT AATATTATAG TGCCTGTTAT
Dren81_Cf_SF   ATGATGCCTA TTATAATAGG TGGGATGGGT AATATTATAG TGCCTGTTAT
Dren82_Cb_Co   ATGATGCCTA TTATAATAGG TGGGATGGGT AATATTATAG TGCCTGTTAT
Dren83_Cb_Ch   ATGATGCCTA TTATAATAGG TGGGATGGGT AATATTATAG TGCCTGTTAT
Dren84_Cf_SF   ATGATGCCTA TTATAATAGG TGGGATGGGT AATATTATAG TGCCTGTTAT
Dren85_Cb_SF   ATGATGCCTA TTATAATAGG TGGGGTGGGT AATATTATAG TGCCTGTTAT
Dren86_Cb_Co   ATGATGCCTA TTATAATAGG TGGGATGGGT AATATTATAG TGCCTGTTAT
Dren87_Cf_BA   ATAATGCCTA TTATAATAGG TGGGATGGGT AATGTTATAG TGCCTGTTAT
Dren88_Lg_SF   ATAATGCCTA TTATAATAGG TGGGATGGGT AATATTATAG TGCCTGTTAT

               ....|....| ....|....| ....|....| ....|....| ....|....|
                   205        215        225        235        245
Dren01_Cf_BA   GTTGGGGCTT CCTGATATAG CTTTTCCACG TCTTAATAAT TTGGGGTTTT
Dren02_Cf_BA   GTTGGGGCTT CCTGATATAG CTTTTCCACG TCTTAATAAT TTGGGGTTTT
Dren03_Cf_BA   GTTGGGGCTT CCTGATATAG CTTTTCCACG TCTTAATAAT TTGGGGTTTT
```

```
Dren05_Cf_BA   GTTGGGGCTT CCTGATATAG CTTTTCCACG TCTTAATAAT TTGGGGTTTT
Dren06_Cf_BA   GTTGGGGCTT CCTGATATAG CTTTTCCACG TCTTAATAAT TTGGGGTTTT
Dren07_Cf_BA   GTTGGGGCTT CCTGATATAG CTTTTCCACG TCTTAATAAT TTGGGGTTTT
Dren08_Cf_BA   GTTGGGGCTT CCTGATATAG CTTTTCCACG TCTTAATAAT TTGGGGTTTT
Dren09_Cf_BA   GTTGGGGCTT CCTGATATAG CTTTTCCACG TCTTAATAAT TTGGGGTTTT
Dren10_Cf_BA   GTTGGGGCTT CCTGATATAG CTTTTCCACG TCTTAATAAT TTGGGGTTTT
Dren11_Cf_BA   GTTGGGGCTT CCTGATATAG CTTTTCCACG TCTTAATAAT TTGGGGTTTT
Dren18_Cf_BA   GTTGGGGCTT CCTGATATAG CTTTTCCACG TCTTAATAAT TTGGGGTTTT
Dren19_Cf_BA   GTTGGGGCTT CCTGATATAG CTTTTCCACG TCTTAATAAT TTGGGGTTTT
Dren23_Cf_BA   GTTGGGGCTT CCTGATATAG CTTTTCCACG TCTTAATAAT TTGGGGTTTT
Dren25_Cf_SF   GTTGGGGCTT CCTGATATAG CTTTTCCACG TCTTAATAAT TTGGGGTTTT
Dren32_Cf_SC   GTTGGGGCTT CCTGATATAG CTTTTCCACG TCTTAATAAT TTGGGGTTTT
Dren33_Cf_SC   GTTGGGGCTT CCTGATATAG CTTTTCCACG TCTTAATAAT TTGGGGTTTT
Dren34_Cf_Pa   GTTGGGGCTT CCTGATATAG CTTTTCCACG TCTTAATAAT TTGGGGTTTT
Dren35_Cf_Pa   GTTGGGGCTT CCTGATATAG CTTTTCCACG TCTTAATAAT TTGGGGTTTT
Dren36_Cf_SC   GTTGGGGCTT CCTGATATAG CTTTTCCACG TCTTAATAAT TTGGGGTTTT
Dren40_Cf_RG   GTTGGGGCTT CCTGATATAG CTTTTCCACG TCTTAATAAT TTGGGGTTTT
Dren41_Cf_RG   GTTGGGGCTT CCTGATATAG CTTTTCCACG TCTTAATAAT TTGGGGTTTT
Dren42_Cf_RG   GTTGGGGCTT CCTGATATAG CTTTTCCACG TCTTAATAAT TTGGGGTTTT
Dren43_Cf_RG   GTTGGGGCTT CCTGATATAG CTTTTCCACG TCTTAATAAT TTGGGGTTTT
Dren45_Cf_RG   GTTGGGGCTT CCTGATATAG CTTTTCCACG TCTTAATAAT TTGGGGTTTT
Dren46_Cf_SF   GTTGGGGCTT CCTGATATAG CTTTTCCACG TCTTAATAAT TTGGGGTTTT
Dren47_Cb_SF   GTTGGGGCTT CCTGATATAG CTTTTCCACG TCTTAATAAT TTGGGGTTTT
Dren48_Cf_BA   GTTGGGGCTT CCTGATATAG CTTTTCCACG TCTTAATAAT TTGGGGTTTT
Dren49_Cf_BA   GTTGGGGCTT CCTGATATAG CTTTTCCACG TCTTAATAAT TTGGGGTTTT
Dren50_Cf_Ch   GTTGGGGCTT CCTGATATAG CTTTTCCACG TCTTAATAAT TTGGGGTTTT
Dren53_Cf_BA   GTTGGGGCTT CCTGATATAG CTTTTCCACG TCTTAATAAT TTGGGGTTTT
Dren58_Cb_SF   GTTGGGGCTT CCTGATATAG CTTTTCCACG TCTTAATAAT TTGGGGTTTT
Dren66_Cf_SF   GTTGGGGCTT CCTGATATAG CTTTTCCACG TCTTAATAAT TTGGGGTTTT
Dren67_Cf_SF   GTTGGGGCTT CCTGATATAG CTTTTCCACG TCTTAATAAT TTGGGGTTTT
Dren68_Cf_SF   GTTGGGGCTT CCTGATATAG CTTTTCCACG TCTTAATAAT TTGGGGTTTT
Dren69_Cf_SF   GTTGGGGCTT CCTGATATAG CTTTTCCACG TCTTAATAAT TTGGGGTTTT
Dren70_Cf_SF   GTTGGGGCTT CCTGATATAG CTTTTCCACG TCTTAATAAT TTGGGGTTTT
Dren72_Cb_SF   GTTGGGGCTT CCTGATATAG CTTTTCCACG TCTTAATAAT TTGGGGTTTT
Dren73_Cb_Ch   GTTGGGGCTT CCTGATATAG CTTTTCCACG TCTTAATAAT TTGGGGTTTT
Dren74_Cb_Co   GTTGGGGCTT CCTGATATAG CTTTTCCACG TCTTAATAAT TTGGGGTTTT
Dren75_Cb_Co   GTTGGGGCTT CCTGATATAG CTTTTCCACG TCTTAATAAT TTGGGGTTTT
Dren80_Cf_SF   GTTGGGGCTT CCTGATATAG CTTTTCCACG TCTTAATAAT TTGGGGTTTT
Dren81_Cf_SF   GTTGGGGCTT CCTGATATAG CTTTTCCACG TCTTAATAAT TTGGGGTTTT
Dren82_Cb_Co   GTTGGGGCTT CCTGATATAG CTTTTCCACG TCTTAATAAT TTGGGGTTTT
Dren83_Cb_Ch   GTTGGGGCTT CCTGATATAG CTTTTCCACG TCTTAATAAT TTGGGGTTTT
Dren84_Cf_SF   GTTGGGGCTT CCTGATATAG CTTTTCCACG TCTTAATAAT TTGGGGTTTT
Dren85_Cb_SF   GTTGGGGCTT CCTGATATAG CTTTTCCACG TCTTAATAAT TTGGGGTTTT
Dren86_Cb_Co   GTTGGGGCTT CCTGATATAG CTTTTCCACG TCTTAATAAT TTGGGGTTTT
Dren87_Cf_BA   GTTGGGGCTT CCTGATATAG CTTTTCCACG TCTTAATAAT TTGGGGTTTT
Dren88_Lg_SF   GTTGGGGCTT CCTGATATAG CTTTTCCACG TCTTAATAAT TTGGGGTTTT

               ....|....| ....|....| ....|....| ....|....| ....|....|
                  255        265        275        285        295
Dren01_Cf_BA   GGTTGTTGCC TCCTTCATTC TTATTGTTTG TAAGTTCCAT AATGTTTTCG
Dren02_Cf_BA   GGTTATTGCC TCCTTCATTC TTATTGTTTG TAAGTTCCAT AATGTTTTCG
Dren03_Cf_BA   GGTTGTTGCC TCCTTCATTC TTATTGTTTG TAAGTTCCAT AATGTTTTCG
Dren05_Cf_BA   GGTTATTGCC TCCTTCATTC TTATTGTTTG TAAGTTCCAT AATGTTTTCG
Dren06_Cf_BA   GGTTGTTGCC TCCTTCATTC TTATTGTTTG TAAGTTCCAT AATGTTTTCG
```

```
Dren07_Cf_BA   GGTTATTGCC TCCTTCATTC TTATTGTTTG TAAGTTCCAT AATGTTTTCG
Dren08_Cf_BA   GGTTGTTGCC TCCTTCATTC TTATTGTTTG TAAGTTCCAT AATGTTTTCG
Dren09_Cf_BA   GGTTATTGCC TCCTTCATTC TTATTGTTTG TAAGTTCCAT AATGTTTTCG
Dren10_Cf_BA   GGTTGTTGCC TCCTTCATTC TTATTGTTTG TAAGTTCCAT AATGTTTTCG
Dren11_Cf_BA   GGTTATTGCC TCCTTCATTC TTATTGTTTG TAAGTTCCAT AATGTTTTCG
Dren18_Cf_BA   GGTTATTGCC TCCTTCATTC TTATTGTTTG TAAGTTCCAT AATGTTTTCG
Dren19_Cf_BA   GGTTATTGCC TCCTTCATTC TTATTGTTTG TAAGTTCCAT AATGTTTTCG
Dren23_Cf_BA   GGTTATTGCC TCCTTCATTC TTATTGTTTG TAAGTTCCAT AATGTTTTCG
Dren25_Cf_SF   GGTTATTGCC TCCTTCATTC TTATTGTTTG TAAGTTCCAT AATGTTTTCG
Dren32_Cf_SC   GGTTATTGCC TCCTTCATTC TTATTGTTTG TAAGTTCCAT AATGTTTTCG
Dren33_Cf_SC   GGTTATTGCC TCCTTCATTC TTATTGTTTG TAAGTTCCAT AATGTTTTCG
Dren34_Cf_Pa   GGTTATTGCC TCCTTCATTC TTATTGTTTG TAAGTTCCAT AATGTTTTCG
Dren35_Cf_Pa   GGTTATTGCC TCCTTCATTC TTATTGTTTG TAAGTTCCAT AATGTTTTCG
Dren36_Cf_SC   GGTTATTGCC TCCTTCATTC TTATTGTTTG TAAGTTCCAT AATGTTTTCG
Dren40_Cf_RG   GGTTATTGCC TCCTTCATTC TTATTGTTTG TAAGTTCCAC GATGTTTTCG
Dren41_Cf_RG   GGTTATTGCC TCCTTCATTC TTATTGTTTG TAAGTTCCAC GATGTTTTCG
Dren42_Cf_RG   GGTTATTGCC TCCTTCATTC TTATTGTTTG TAAGTTCCAT AATGTTTTCG
Dren43_Cf_RG   GGTTATTGCC TCCTTCATTC TTATTGTTTG TAAGTTCCAT AATGTTTTCG
Dren45_Cf_RG   GGTTATTGCC TCCTTCATTC TTATTGTTTG TAAGTTCCAT AATGTTTTCG
Dren46_Cf_SF   GGTTATTGCC TCCTTCATTC TTATTGTTTG TAAGTTCCAT AATGTTTTCG
Dren47_Cb_SF   GGTTATTGCC TCCTTCATTC TTATTGTTTG TAAGTTCCAT AATGTTTTCG
Dren48_Cf_BA   GGTTGTTGCC TCCTTCATTC TTATTGTTTG TAAGTTCCAT AATGTTTTCG
Dren49_Cf_BA   GGTTATTGCC TCCTTCATTC TTATTGTTTG TAAGTTCCAT AATGTTTTCG
Dren50_Cf_Ch   GGTTGTTGCC TCCTTCATTC TTATTGTTTG TAAGTTCCAT AATGTTTTCG
Dren53_Cf_BA   GGTTGTTGCC TCCTTCATTC TTATTGTTTG TAAGTTCCAT AATGTTTTCG
Dren58_Cb_SF   GGTTATTGCC TCCTTCATTC TTATTGTTTG TAAGTTCCAT GATGTTTTCG
Dren66_Cf_SF   GGTTATTGCC TCCTTCATTC TTATTGTTTG TAAGTTCCAT AATGTTTTCG
Dren67_Cf_SF   GGTTATTGCC TCCTTCATTC TTATTGTTTG TAAGTTCCAT AATGTTTTCG
Dren68_Cf_SF   GGTTATTGCC TCCTTCATTC TTATTGTTTG TAAGTTCCAT AATGTTTTCG
Dren69_Cf_SF   GGTTATTGCC TCCTTCATTC TTATTGTTTG TAAGTTCCAT AATGTTTTCG
Dren70_Cf_SF   GGTTATTGCC TCCTTCATTC TTATTGTTTG TAAGTTCCAT AATGTTTTCG
Dren72_Cb_SF   GGTTGTTGCC TCCTTCATTC TTATTGTTTG TAAGTTCCAT AATGTTTTCG
Dren73_Cb_Ch   GGTTATTGCC TCCTTCATTC TTATTGTTTG TAAGTTCCAT AATGTTTTCG
Dren74_Cb_Co   GGTTATTGCC TCCTTCATTC TTATTGTTTG TAAGTTCCAT AATGTTTTCG
Dren75_Cb_Co   GGTTGTTGCC TCCTTCATTC TTATTGTTTG TAAGTTCCAT AATGTTTTCG
Dren80_Cf_SF   GGTTATTGCC TCCTTCATTC TTATTGTTTG TAAGTTCCAT AATGTTTTCG
Dren81_Cf_SF   GGTTATTGCC TCCTTCATTC TTATTGTTTG TAAGTTCCAT AATGTTTTCG
Dren82_Cb_Co   GGTTATTGCC TCCTTCATTC TTATTGTTTG TAAGTTCCAT AATGTTTTCG
Dren83_Cb_Ch   GGTTGTTGCC TCCTTCATTC TTATTGTTTG TAAGTTCCAT AATGTTTTCG
Dren84_Cf_SF   GGTTATTGCC TCCTTCATTC TTATTGTTTG TAAGTTCCAT AATGTTTTCG
Dren85_Cb_SF   GGTTATTGCC TCCTTCATTC TTATTGTTTG TAAGTTCCAT AATGTTTTCG
Dren86_Cb_Co   GGTTATTGCC TCCTTCATTC TTATTGTTTG TAAGTTCCAT AATGTTTTCG
Dren87_Cf_BA   GGTTATTGCC TCCTTCATTC TTATTGTTTG TAAGTTCCAT AATGTTTTCG
Dren88_Lg_SF   GGTTATTGCC TCCTTCATTC TTATTGTTTG TAAGTTCCAT AATGTTTTCG

               ....|....| ....|....| ....|....| ....|....| ....|....|
                   305        315        325        335        345
Dren01_Cf_BA   GTTTCTGGCC CGGGCACAGG TTGAACCCTT TACCCTCCTC TATCTTTATT
Dren02_Cf_BA   GTTTCTGGCC CGGGCACAGG TTGAACCCTT TACCCTCCTT TATCTTTATT
Dren03_Cf_BA   GTTTCTGGCC CGGGCACAGG TTGAACCCTT TACCCTCCTC TATCTTTATT
Dren05_Cf_BA   GTTTCTGGCC CGGGCACAGG TTGAACCCTT TACCCTCCTC TATCTTTATT
Dren06_Cf_BA   GTTTCTGGCC CGGGCACAGG TTGAACCCTT TACCCTCCTC TATCTTTATT
Dren07_Cf_BA   GTTTCTGGCC CGGGCACAGG TTGAACCCTT TACCCTCCTC TATCTTTATT
Dren08_Cf_BA   GTTTCTGGCC CGGGCACAGG TTGAACCCTT TACCCTCCTC TATCTTTATT
```

```
Dren09_Cf_BA   GTTTCTGGCC CGGGCACAGG TTGGACCCTT TACCCTCCTC TATCTTTATT
Dren10_Cf_BA   GTTTCTGGCC CGGGCACAGG TTGAACCCTT TACCCTCCTC TATCTTTATT
Dren11_Cf_BA   GTTTCTGGCC CGGGCACAGG TTGGACCCTT TACCCTCCTC TATCTTTATT
Dren18_Cf_BA   GTTTCTGGCC CGGGCACAGG TTGAACCCTT TACCCTCCTC TATCTTTATT
Dren19_Cf_BA   GTTTCTGGCC CGGGCACAGG TTGAACCCTT TACCCTCCTC TATCTTTATT
Dren23_Cf_BA   GTTTCTGGCC CGGGCACAGG TTGGACCCTC TACCCTCCTC TATCTTTATT
Dren25_Cf_SF   GTTTCTGGCC CGGGCACAGG TTGGACCCTT TACCCTCCTC TATCTTTATT
Dren32_Cf_SC   GTTTCTGGCC CGGGCACAGG TTGGACCCTT TACCCTCCTC TATCTTTATT
Dren33_Cf_SC   GTTTCTGGCC CGGGCACAGG TTGGACCCTT TACCCTCCTC TATCTTTATT
Dren34_Cf_Pa   GTTTCTGGCC CGGGCACAGG TTGGACCCTT TACCCTCCTC TATCTTTATT
Dren35_Cf_Pa   GTTTCTGGCC CGGGCACAGG TTGGACCCTT TACCCTCCTC TATCTTTATT
Dren36_Cf_SC   GTTTCTGGCC CGGGCACAGG TTGGACCCTT TACCCTCCTC TATCTTTATT
Dren40_Cf_RG   GTTTCTGGCC CGGGCACAGG TTGAACCCTT TACCCTCCTC TATCTTTATT
Dren41_Cf_RG   GTTTCTGGCC CGGGCACAGG TTGAACCCTT TACCCTCCTC TATCTTTATT
Dren42_Cf_RG   GTTTCTGGCC CGGGCACAGG TTGAACCCTT TACCCTCCTC TATCTTTATT
Dren43_Cf_RG   GTTTCTGGCC CGGGCACAGG TTGAACCCTT TACCCTCCTC TATCTTTATT
Dren45_Cf_RG   GTTTCTGGCC CGGGCACAGG TTGAACCCTT TACCCTCCTC TATCTTTATT
Dren46_Cf_SF   GTTTCTGGCC CGGGCACAGG TTGGACCCTT TACCCTCCTC TATCTTTATT
Dren47_Cb_SF   GTTTCTGGCC CGGGCACAGG TTGAACCCTT TACCCTCCTC TATCTTTATT
Dren48_Cf_BA   GTTTCTGGCC CGGGCACAGG TTGAACCCTT TACCCTCCTC TATCTTTATT
Dren49_Cf_BA   GTTTCTGGCC CGGGCACAGG TTGGACCCTC TACCCTCCTC TATCTTTATT
Dren50_Cf_Ch   GTTTCTGGCC CGGGCACAGG TTGAACCCTT TACCCTCCTC TATCTTTATT
Dren53_Cf_BA   GTTTCTGGCC CGGGCACAGG TTGAACCCTT TACCCTCCTC TATCTTTATT
Dren58_Cb_SF   GTTTCTGGCC CGGGCACAGG TTGAACCCTT TACCCTCCTC TATCTTTATT
Dren66_Cf_SF   GTTTCTGGCC CGGGCACAGG TTGAACCCTT TACCCTCCTC TATCTTTATT
Dren67_Cf_SF   GTTTCTGGCC CGGGCACAGG TTGAACCCTT TACCCTCCTC TATCTTTATT
Dren68_Cf_SF   GTTTCTGGCC CGGGCACAGG TTGAACCCTT TACCCTCCTC TATCTTTATT
Dren69_Cf_SF   GTTTCTGGCC CGGGCACAGG TTGAACCCTT TACCCTCCTC TATCTTTATT
Dren70_Cf_SF   GTTTCTGGCC CGGGCACAGG TTGGACCCTT TACCCTCCTC TATCTTTATT
Dren72_Cb_SF   GTTTCTGGCC CGGGCACAGG TTGAACCCTT TACCCTCCTC TATCTTTATT
Dren73_Cb_Ch   GTTTCTGGCC CGGGCACAGG TTGAACCCTT TACCCTCCTC TATCTTTATT
Dren74_Cb_Co   GTTTCTGGCC CGGGCACAGG TTGGACCCTT TACCCTCCTC TATCTTTATT
Dren75_Cb_Co   GTTTCTGGCC CGGGCACAGG TTGAACCCTT TACCCTCCTC TATCTTTATT
Dren80_Cf_SF   GTTTCTGGCC CGGGCACAGG TTGGACCCTT TACCCTCCTC TATCTTTATT
Dren81_Cf_SF   GTTTCTGGCC CGGGCACAGG TTGGACCCTT TACCCTCCTC TATCTTTATT
Dren82_Cb_Co   GTTTCTGGCC CGGGCACAGG TTGGACCCTT TACCCTCCTC TATCTTTATT
Dren83_Cb_Ch   GTTTCTGGCC CGGGCACAGG TTGAACCCTT TACCCTCCTC TATCTTTATT
Dren84_Cf_SF   GTTTCTGGCC CGGGCACAGG TTGGACCCTT TACCCTCCTC TATCTTTATT
Dren85_Cb_SF   GTTTCTGGCC CGGGCACAGG TTGGACCCTC TACCCTCCTC TATCTTTATT
Dren86_Cb_Co   GTTTCTGGCC CGGGCACAGG TTGAACCCTT TACCCTCCTC TATCTTTATT
Dren87_Cf_BA   GTTTCTGGCC CGGGCACAGG TTGAACCCTT TACCCTCCTC TATCTTTATT
Dren88_Lg_SF   GTTTCTGGCC CGGGCACAGG TTGAACCCTT TACCCTCCTC TATCTTTATT

               ....|....| ....|....| ....|....| ....|....| ....|....|
                   355        365        375        385        395
Dren01_Cf_BA   TACTGGGCAT CCTGATGTTT GCGTGGATTT TGTGATCTTT TCTCTACATT
Dren02_Cf_BA   TACTGGGCAT CCTGATGTTT GCGTGGATTT TGTGATCTTT TCTCTACATT
Dren03_Cf_BA   TACTGGGCAT CCTGATGTTT GCGTGGATTT TGTGATCTTT TCTCTACATT
Dren05_Cf_BA   TACTGGGCAT CCTGATGTTT GCGTGGATTT TGTGATCTTT TCTCTACATT
Dren06_Cf_BA   TACTGGGCAT CCTGATGTTT GCGTGGATTT TGTGATCTTT TCTCTACATT
Dren07_Cf_BA   TACTGGGCAT CCTGATGTTT GCGTGGATTT TGTGATCTTT TCTCTACATT
Dren08_Cf_BA   TACTGGGCAT CCTGATGTTT GCGTGGATTT TGTGATCTTT TCTCTACATT
Dren09_Cf_BA   TACTGGGCAT CCTGATGTTT GCGTGGATTT TGTGATCTTT TCTCTACATT
Dren10_Cf_BA   TACTGGGCAT CCTGATGTTT GCGTGGATTT TGTGATCTTT TCTCTACATT
```

```
Dren11_Cf_BA   TACTGGGCAT CCTGATGTTT GCGTGGATTT TGTGATCTTT TCTCTACATT
Dren18_Cf_BA   TACTGGACAT CCTGATGTCT GCGTGGATTT TGTGATCTTT TCTCTACATT
Dren19_Cf_BA   TACTGGACAT CCTGATGTCT GCGTGGATTT TGTGATCTTT TCTCTACATT
Dren23_Cf_BA   TACTGGGCAT CCTGATGTTT GCGTGGATTT TGTGATCTTT TCTCTACATT
Dren25_Cf_SF   TACTGGGCAT CCTGATGTTT GCGTGGATTT TGTGATCTTT TCTCTACATT
Dren32_Cf_SC   TACTGGGCAT CCTGATGTTT GCGTGGATTT TGTGATCTTT TCTCTACATT
Dren33_Cf_SC   TACTGGGCAT CCTGATGTTT GCGTGGATTT TGTGATCTTT TCTCTACATT
Dren34_Cf_Pa   TACTGGGCAT CCTGATGTTT GCGTGGATTT TGTGATCTTT TCTCTACATT
Dren35_Cf_Pa   TACTGGGCAT CCTGATGTTT GCGTGGATTT TGTGATCTTT TCTCTACATT
Dren36_Cf_SC   TACTGGGCAT CCTGATGTTT GCGTGGATTT TGTGATCTTT TCTCTACATT
Dren40_Cf_RG   TACTGGGCAT CCTGATGTTT GCGTGGATTT TGTGATCTTT TCTCTACATT
Dren41_Cf_RG   TACTGGGCAT CCTGATGTTT GCGTGGATTT TGTGATCTTT TCTCTACATT
Dren42_Cf_RG   TACTGGGCAT CCTGATGTTT GCGTGGATTT TGTGATCTTT TCTCTACATT
Dren43_Cf_RG   TACTGGGCAT CCTGATGTTT GCGTGGATTT TGTGATCTTT TCTCTACATT
Dren45_Cf_RG   TACTGGGCAT CCTGATGTTT GCGTGGATTT TGTGATCTTT TCTCTACATT
Dren46_Cf_SF   TACTGGGCAT CCTGATGTTT GCGTGGATTT TGTGATCTTT TCTCTACATT
Dren47_Cb_SF   TACTGGGCAT CCTGATGTTT GCGTGGATTT TGTGATCTTT TCTCTACATT
Dren48_Cf_BA   TACTGGGCAT CCTGATGTTT GCGTGGATTT TGTGATCTTT TCTCTACATT
Dren49_Cf_BA   TACTGGGCAT CCTGATGTTT GCGTGGATTT TGTGATCTTT TCTCTACATT
Dren50_Cf_Ch   TACTGGGCAT CCTGATGTTT GCGTGGATTT TGTGATCTTT TCTCTACATT
Dren53_Cf_BA   TACTGGACAT CCTGATGTTT GCGTGGATTT TGTGATCTTT TCTCTACATT
Dren58_Cb_SF   TACTGGGCAT CCTGATGTTT GCGTGGATTT TGTGATCTTT TCTCTACATT
Dren66_Cf_SF   TACTGGACAT CCTGATGTCT GCGTGGATTT TGTGATCTTT TCTCTACATT
Dren67_Cf_SF   TACTGGGCAT CCTGATGTTT GCGTGGATTT TGTGATCTTT TCTCTACATT
Dren68_Cf_SF   TACTGGGCAT CCTGATGTTT GCGTGGATTT TGTGATCTTT TCTCTACATT
Dren69_Cf_SF   TACTGGGCAT CCTGATGTTT GCGTGGATTT TGTGATCTTT TCTCTACATT
Dren70_Cf_SF   TACTGGGCAT CCTGATGTTT GCGTGGATTT TGTGATCTTT TCTCTACATT
Dren72_Cb_SF   TACTGGGCAT CCTGATGTTT GCGTGGATTT TGTGATCTTT TCTCTACATT
Dren73_Cb_Ch   TACTGGGCAT CCTGATGTTT GCGTGGATTT TGTGATCTTT TCTCTACATT
Dren74_Cb_Co   TACTGGTCAT CCTGATGTTT GCGTGGATTT TGTGATCTTT TCTCTACATT
Dren75_Cb_Co   TACTGGGCAT CCTGATGTTT GCGTGGATTT TGTGATCTTT TCTCTACATT
Dren80_Cf_SF   TACTGGGCAT CCTGATGTTT GCGTGGATTT TGTGATCTTT TCTCTACATT
Dren81_Cf_SF   TACTGGGCAT CCTGATGTTT GCGTGGATTT TGTGATCTTT TCTCTACATT
Dren82_Cb_Co   TACTGGGCAT CCTGATGTTT GCGTGGATTT TGTGATCTTT TCTCTACATT
Dren83_Cb_Ch   TACTGGGCAT CCTGATGTTT GCGTGGATTT TGTGATCTTT TCTCTACATT
Dren84_Cf_SF   TACTGGGCAT CCTGATGTTT GCGTGGATTT TGTGATCTTT TCTCTACATT
Dren85_Cb_SF   TACTGGGCAT CCTGATGTTT GCGTGGATTT TGTGATCTTT TCTCTACATT
Dren86_Cb_Co   TACTGGGCAT CCTGATGTTT GCGTGGATTT TGTGATCTTT TCTCTACATT
Dren87_Cf_BA   TACTGGGCAT CCTGATGTTT GCGTGGATTT TGTGATCTTT TCTCTACATT
Dren88_Lg_SF   TACTGGGCAT CCTGATGTTT GCGTGGATTT TGTGATCTTT TCTCTACATT

               ....|....| ....|....| ....|....| ....|....| ....|....|
                     405        415        425        435        445
Dren01_Cf_BA   TGGCGGGGGT GTCTTCTCTA TTAAGAAGTG TAAATTTTGT AACGACGTGT
Dren02_Cf_BA   TGGCGGGGGT GTCTTCTCTA TTAAGAAGTG TAAATTTTGT AACGACGTGT
Dren03_Cf_BA   TGGCGGGGGT GTCTTCTCTA TTAAGAAGTG TAAATTTTGT AACGACGTGT
Dren05_Cf_BA   TGGCGGGGGT GTCTTCTCTA TTAAGAAGTG TAAATTTTGT AACGACGTGT
Dren06_Cf_BA   TAGCGGGGGT GTCTTCTCTA TTAAGAAGTG TAAATTTTGT AACGACGTGT
Dren07_Cf_BA   TGGCGGGGGT GTCTTCTCTA TTAAGAAGTG TAAATTTTGT AACGACGTGT
Dren08_Cf_BA   TGGCGGGGGT GTCTTCTCTA TTAAGAAGTG TAAATTTTGT AACGACGTGT
Dren09_Cf_BA   TGGCGGGGGT GTCTTCTCTA TTAAGAAGTG TAAATTTTGT AACGACGTGT
Dren10_Cf_BA   TGGCGGGGGT GTCTTCTCTA TTAAGAAGTG TAAATTTTGT AACGACGTGT
Dren11_Cf_BA   TGGCGGGGGT GTCTTCTCTA TTAAGAAGTG TAAATTTTGT AACGACGTGT
Dren18_Cf_BA   TGGCGGGGGT GTCTTCTCTA TTAAGAAGTG TAAATTTTGT AACGACGTGT
```

```
Dren19_Cf_BA   TGGCGGGGGT GTCTTCTCTA TTAAGAAGTG TAAATTTTGT AACGACGTGT
Dren23_Cf_BA   TGGCGGGGGT GTCTTCTCTA TTAAGAAGTG TAAATTTTGT AACGACGTGT
Dren25_Cf_SF   TGGCGGGGGT GTCTTCTCTA TTAAGAAGTG TAAATTTTGT AACGACGTGT
Dren32_Cf_SC   TGGCGGGGGT GTCTTCTCTA TTAAGAAGTG TAAATTTTGT AACGACGTGT
Dren33_Cf_SC   TGGCGGGGGT GTCTTCTCTA TTAAGAAGTG TAAATTTTGT AACGACGTGT
Dren34_Cf_Pa   TGGCGGGGGT GTCTTCTCTA TTAAGAAGTG TAAATTTTGT AACGACGTGT
Dren35_Cf_Pa   TGGCGGGGGT GTCTTCTCTA TTAAGAAGTG TAAATTTTGT AACGACGTGT
Dren36_Cf_SC   TGGCGGGGGT GTCTTCTCTA TTAAGAAGTG TAAATTTTGT AACGACGTGT
Dren40_Cf_RG   TGGCGGGGGT GTCTTCTCTA TTAAGAAGTG TAAATTTTGT AACGACGTGT
Dren41_Cf_RG   TGGCGGGGGT GTCTTCTCTA TTAAGAAGTG TAAATTTTGT AACGACGTGT
Dren42_Cf_RG   TGGCGGGGGT GTCTTCTCTA TTAAGAAGTG TAAATTTTGT AACGACGTGT
Dren43_Cf_RG   TGGCGGGGGT GTCTTCTCTA TTAAGAAGTG TAAATTTTGT AACGACGTGT
Dren45_Cf_RG   TGGCGGGGGT GTCTTCTCTA TTAAGAAGTG TAAATTTTGT AACGACGTGT
Dren46_Cf_SF   TGGCGGGGGT GTCTTCTCTA TTAAGAAGTG TAAATTTTGT AACGACGTGT
Dren47_Cb_SF   TGGCGGGGGT GTCTTCTCTA TTAAGAAGTG TAAATTTTGT AACGACGTGT
Dren48_Cf_BA   TGGCGGGGGT GTCTTCTCTA TTAAGAAGTG TAAATTTTGT AACGACGTGT
Dren49_Cf_BA   TGGCGGGGGT GTCTTCTCTA TTAAGAAGTG TAAATTTTGT AACGACGTGT
Dren50_Cf_Ch   TGGCGGGGGT GTCTTCTCTA TTAAGAAGTG TAAATTTTGT AACGACGTGT
Dren53_Cf_BA   TGGCGGGGGT GTCTTCTCTA TTAAGAAGTG TAAATTTTGT AACGACGTGT
Dren58_Cb_SF   TGGCGGGGGT GTCTTCTCTA TTAAGAAGTG TAAATTTTGT AACGACGTGT
Dren66_Cf_SF   TGGCGGGGGT GTCTTCTCTA TTAAGAAGTG TAAATTTTGT AACGACGTGT
Dren67_Cf_SF   TGGCGGGGGT GTCTTCTCTA TTAAGAAGTG TAAATTTTGT AACGACGTGT
Dren68_Cf_SF   TGGCGGGGGT GTCTTCTCTA TTAAGAAGTG TAAATTTTGT AACGACGTGT
Dren69_Cf_SF   TGGCGGGGGT GTCTTCTCTA TTAAGAAGTG TAAATTTTGT AACGACGTGT
Dren70_Cf_SF   TGGCGGGGGT GTCTTCTCTA TTAAGAAGTG TAAATTTTGT AACGACGTGT
Dren72_Cb_SF   TGGCGGGGGT GTCTTCTCTA TTAAGAAGTG TAAATTTTGT AACGACGTGT
Dren73_Cb_Ch   TGGCGGGGGT GTCTTCTCTA TTAAGAAGTG TAAATTTTGT AACGACGTGT
Dren74_Cb_Co   TGGCGGGGGT GTCTTCTCTA TTAAGAAGTG TAAATTTTGT AACGACGTGT
Dren75_Cb_Co   TGGCGGGGGT GTCTTCTCTA TTAAGAAGTG TAAATTTTGT AACGACGTGT
Dren80_Cf_SF   TGGCGGGGGT GTCTTCTCTA TTAAGAAGTG TAAATTTTGT AACGACGTGT
Dren81_Cf_SF   TGGCGGGGGT GTCTTCTCTA TTAAGAAGTG TAAATTTTGT AACGACGTGT
Dren82_Cb_Co   TGGCGGGGGT GTCTTCTCTA TTAAGAAGTG TAAATTTTGT AACGACGTGT
Dren83_Cb_Ch   TGGCGGGGGT GTCTTCTCTA TTAAGAAGTG TAAATTTTGT AACGACGTGT
Dren84_Cf_SF   TGGCGGGGGT GTCTTCTCTA TTAAGAAGTG TAAATTTTGT AACGACGTGT
Dren85_Cb_SF   TGGCGGGGGT GTCTTCTCTA TTAAGAAGTG TAAATTTTGT AACGACGTGT
Dren86_Cb_Co   TGGCGGGGGT GTCTTCTCTA TTAAGAAGTG TAAATTTTGT AACGACGTGT
Dren87_Cf_BA   TGGCGGGGGT GTCTTCTCTA TTAAGAAGTG TAAATTTTGT AACGACGTGT
Dren88_Lg_SF   TGGCGGGGGT GTCTTCTCTA TTAAGAAGTG TAAATTTTGT AACGACGTGT

               ....|....| ....|....| ....|....| ....|....| ....|....|
                    455        465        475        485        495
Dren01_Cf_BA   TTTTCTGTTT GTGATTCGGT TTCTTCATTA GAAGATATGC CAATGTTTGC
Dren02_Cf_BA   TTTTCTGTTT GTGATTCGGT TTCTTCATTA GAAGATATGC CAATGTTTGC
Dren03_Cf_BA   TTTTCTGTTT GTGATTCGGT TTCTTCATTA GAAGATATGC CAATGTTTGC
Dren05_Cf_BA   TTTTCTGTTT GTGATTCGGT TTCTTCATTA GAAGATATGC CAATGTTTGC
Dren06_Cf_BA   TTTTCTGTTT GTGATTCGGT TTCTTCATTA GAAGATATGC CAATGTTTGC
Dren07_Cf_BA   TTTTCTGTTT GTGATTCGGT TTCTTCATTA GAAGATATGC CAATGTTTGC
Dren08_Cf_BA   TTTTCTGTTT GTGATTCGGT TTCTTCATTA GAAGATATGC CAATGTTTGC
Dren09_Cf_BA   TTTTCTGTTT GTGGTTCGGT TTCTTCATTA GAAGATATGC CAATGTTTGC
Dren10_Cf_BA   TTTTCTGTTT GTGATTCGGT TTCTTCATTA GAAGATATGC CAATGTTTGC
Dren11_Cf_BA   TTTTCTGTTT GTGATTCGGT TTCTTCATTA GAAGATATGC CAATGTTTGC
Dren18_Cf_BA   TTTTCTGTTT GTGATTCGGT TTCTTCATTA GAAGATATGC CAATGTTTGC
Dren19_Cf_BA   TTTTCTGTTT GTGATTCGGT TTCTTCATTA GAAGATATGC CAATGTTTGC
Dren23_Cf_BA   TTTTCTGTTT GTGATTCGGT TTCTTCATTA GAAGATATGC CAATGTTTGC
```

```
Dren25_Cf_SF   TTTTCTGTTT GTGATTCGGT TTCTTCATTA GAAGATATGC CAATGTTTGC
Dren32_Cf_SC   TTTTCTGTTT GTGATTCGGT TTCTTCATTA GAAGATATGC CAATGTTTGC
Dren33_Cf_SC   TTTTCTGTTT GTGATTCGGT TTCTTCATTA GAAGATATGC CAATGTTTGC
Dren34_Cf_Pa   TTTTCTGTTT GTGATTCGGT TTCTTCATTA GAAGATATGC CAATGTTTGC
Dren35_Cf_Pa   TTTTCTGTTT GTGATTCGGT TTCTTCATTA GAAGATATGC CAATGTTTGC
Dren36_Cf_SC   TTTTCTGTTT GTGATTCGGT TTCTTCATTA GAAGATATGC CAATGTTTGC
Dren40_Cf_RG   TTTTCTGTTT GTGATTCGGT TTCTTCATTA GAAGATATGC CAATGTTTGC
Dren41_Cf_RG   TTTTCTGTTT GTGATTCGGT TTCTTCATTA GAAGATATGC CAATGTTTGC
Dren42_Cf_RG   TTTTCTGTTT GTGATTCGGT TTCTTCATTA GAAGATATGC CAATGTTTGC
Dren43_Cf_RG   TTTTCTGTTT GTGATTCGGT TTCTTCATTA GAAGATATGC CAATGTTTGC
Dren45_Cf_RG   TTTTCTGTTT GTGATTCGGT TTCTTCATTA GAAGATATGC CAATGTTTGC
Dren46_Cf_SF   TTTTCTGTTT GTGATTCGGT TTCTTCATTA GAAGATATGC CAATGTTTGC
Dren47_Cb_SF   TTTTCTGTTT GTGATTCGGT TTCTTCATTA GAAGATATGC CAATGTTTGC
Dren48_Cf_BA   TTTTCTGTTT GTGATTCGGT TTCTTCATTA GAAGATATGC CAATGTTTGC
Dren49_Cf_BA   TTTTCTGTTT GTGATTCGGT TTCTTCATTA GAAGATATGC CAATGTTTGC
Dren50_Cf_Ch   TTTTCTGTTT GTGATTCGGT TTCTTCATTA GAAGATATGC CAATGTTTGC
Dren53_Cf_BA   TTTTCTGTTT GTGATTCGGT TTCTTCATTA GAAGATATGC CAATGTTTGC
Dren58_Cb_SF   TTTTCTGTTT GTGATTCGGT TTCTTCATTA GAAGATATGC CAATGTTTGC
Dren66_Cf_SF   TTTTCTGTTT GTGATTCGGT TTCTTCATTA GAAGATATGC CAATGTTTGC
Dren67_Cf_SF   TTTTCTGTTT GTGATTCGGT TTCTTCATTA GAAGATATGC CAATGTTTGC
Dren68_Cf_SF   TTTTCTGTTT GTGATTCGGT TTCTTCATTA GAAGATATGC CAATGTTTGC
Dren69_Cf_SF   TTTTCTGTTT GTGATTCGGT TTCTTCATTA GAAGATATGC CAATGTTTGC
Dren70_Cf_SF   TTTTCTGTTT GTGATTCGGT TTCTTCATTA GAAGATATGC CAATGTTTGC
Dren72_Cb_SF   TTTTCTGTTT GTGATTCGGT TTCTTCATTA GAAGATATGC CAATGTTTGC
Dren73_Cb_Ch   TTTTCTGTTT GTGATTCGGT TTCTTCATTA GAAGATATGC CAATGTTTGC
Dren74_Cb_Co   TTTTCTGTTT GTGATTCGGT TTCTTCATTA GAAGATATGC CAATGTTTGC
Dren75_Cb_Co   TTTTCTGTTT GTGATTCGGT TTCTTCATTA GAAGATATGC CAATGTTTGC
Dren80_Cf_SF   TTTTCTGTTT GTGATTCGGT TTCTTCATTA GAAGATATGC CAATGTTTGC
Dren81_Cf_SF   TTTTCTGTTT GTGATTCGGT TTCTTCATTA GAAGATATGC CAATGTTTGC
Dren82_Cb_Co   TTTTCTGTTT GTGATTCGGT TTCTTCATTA GAAGATATGC CAATGTTTGC
Dren83_Cb_Ch   TTTTCTGTTT GTGATTCGGT TTCTTCATTA GAAGATATGC CAATGTTTGC
Dren84_Cf_SF   TTTTCTGTTT GTGATTCGGT TTCTTCATTA GAAGATATGC CAATGTTTGC
Dren85_Cb_SF   TTTTCTGTTT GTGATTCGGT TTCTTCATTA GAAGATATGC CAATGTTTGC
Dren86_Cb_Co   TTTTCTGTTT GTGATTCGGT TTCTTCATTA GAAGATATGC CAATGTTTGC
Dren87_Cf_BA   TTTTCTGTTT GTGATTCGGT TTCTTCATTA GAAGATATGC CAATGTTTGC
Dren88_Lg_SF   TTTTCTGTTT GTGATTCGGT TTCTTCATTA GAAGATATGC CAATGTTTGC

               ....|....| ....|....| ....|....| ....|....| ....|....|
                   505        515        525        535        545
Dren01_Cf_BA   GTGAAGCATT GTGGTGACGT CCTTCATACT AATTGTTTCT CTTCCTGTTT
Dren02_Cf_BA   GTGAAGCATT GTGGTGACGT CCTTCATACT AATTGTTTCT CTTCCTGTTT
Dren03_Cf_BA   GTGAAGCATT GTGGTGACGT CCTTCATACT AATTGTTTCT CTTCCTGTTT
Dren05_Cf_BA   GTGAAGCATT GTGGTGACGT CCTTCATACT AATTGTTTCT CTTCCTGTTT
Dren06_Cf_BA   GTGAAGCATT GTGGTGACGT CCTTCATACT AATTGTTTCT CTTCCTGTTT
Dren07_Cf_BA   GTGAAGCATT GTGGTGACGT CCTTCATACT AATTGTTTCT CTTCCTGTTT
Dren08_Cf_BA   GTGAAGCATT GTGGTGACGT CCTTCATACT AATTGTTTCT CTTCCTGTTT
Dren09_Cf_BA   GTGAAGCATT GTGGTGACGT CCTTCATACT AATTGTTTCT CTTCCTGTTT
Dren10_Cf_BA   GTGAAGCATT GTGGTGACGT CCTTCATACT AATTGTTTCT CTTCCTGTTT
Dren11_Cf_BA   GTGAAGCATT GTGGTGACGT CCTTCATACT AATTGTTTCT CTTCCTGTTT
Dren18_Cf_BA   GTGAAGCATT GTGGTGACGT CCTTCATACT AATTGTTTCT CTTCCTGTTT
Dren19_Cf_BA   GTGAAGCATT GTGGTGACGT CCTTCATACT AATTGTTTCT CTTCCTGTTT
Dren23_Cf_BA   GTGAAGCATT GTGGTGACGT CCTTCATACT AATTGTCTCT CTTCCTGTTT
Dren25_Cf_SF   GTGAAGCATT GTGGTGACGT CCTTCATACT AATTGTTTCT CTTCCTGTTT
Dren32_Cf_SC   GTGAAGCATT GTGGTGACGT CCTTCATACT AATTGTTTCT CTTCCTGTTT
```

```
Dren33_Cf_SC   GTGAAGCATT GTGGTGACGT CCTTCATACT AATTGTTTCT CTTCCTGTTT
Dren34_Cf_Pa   GTGAAGCATT GTGGTGACGT CCTTCATACT AATTGTTTCT CTTCCTGTTT
Dren35_Cf_Pa   GTGAAGCATT GTGGTGACGT CCTTCATACT AATTGTTTCT CTTCCTGTTT
Dren36_Cf_SC   GTGAAGCATT GTGGTGACGT CCTTCATACT AATTGTTTCT CTTCCTGTTT
Dren40_Cf_RG   GTGAAGCATT GTGGTGACGT CCTTCATACT AATTGTTTCT CTTCCTGTTT
Dren41_Cf_RG   GTGAAGCATT GTGGTGACGT CCTTCATACT AATTGTTTCT CTTCCTGTTT
Dren42_Cf_RG   GTGAAGCATT GTGGTGACGT CCTTCATACT AATTGTTTCT CTTCCTGTTT
Dren43_Cf_RG   GTGAAGCATT GTGGTGACGT CCTTCATACT AATTGTTTCT CTTCCTGTTT
Dren45_Cf_RG   GTGAAGCATT GTGGTGACGT CCTTCATACT AATTGTTTCT CTTCCTGTTT
Dren46_Cf_SF   GTGAAGCATT GTGGTGACGT CCTTCATACT AATTGTTTCT CTTCCTGTTT
Dren47_Cb_SF   GTGAAGCATT GTGGTGACGT CCTTCATACT AATTGTTTCT CTTCCTGTTT
Dren48_Cf_BA   GTGAAGCATT GTGGTGACGT CCTTCATACT AATTGTTTCT CTTCCTGTTT
Dren49_Cf_BA   GTGAAGCATT GTGGTGACGT CCTTCATACT AATTGTTTCT CTTCCTGTTT
Dren50_Cf_Ch   GTGAAGCATT GTGGTGACGT CCTTCATACT AATTGTTTCT CTTCCTGTTT
Dren53_Cf_BA   GTGAAGCATT GTGGTGACGT CCTTCATACT AATTGTTTCT CTTCCTGTTT
Dren58_Cb_SF   GTGAAGCATT GTGGTGACGT CCTTCATACT AATTGTTTCT CTTCCTGTTT
Dren66_Cf_SF   GTGAAGCATT GTGGTGACGT CCTTCATACT AATTGTTTCT CTTCCTGTTT
Dren67_Cf_SF   GTGAAGCATT GTGGTGACGT CCTTCATACT AATTGTTTCT CTTCCTGTTT
Dren68_Cf_SF   GTGAAGCATT GTGGTGACGT CCTTCATACT AATTGTTTCT CTTCCTGTTT
Dren69_Cf_SF   GTGAAGCATT GTGGTGACGT CCTTCATACT AATTGTTTCT CTTCCTGTTT
Dren70_Cf_SF   GTGAAGCATT GTGGTGACGT CCTTCATACT AATTGTTTCT CTTCCTGTTT
Dren72_Cb_SF   GTGAAGCATT GTGGTGACGT CCTTCATACT AATTGTTTCT CTTCCTGTTT
Dren73_Cb_Ch   GTGAAGCATT GTGGTGACGT CCTTCATACT AATTGTTTCT CTTCCTGTTT
Dren74_Cb_Co   GTGAAGCATT GTGGTGACGT CCTTCATACT AATTGTTTCT CTTCCTGTTT
Dren75_Cb_Co   GTGAAGCATT GTGGTGACGT CCTTCATACT AATTGTTTCT CTTCCTGTTT
Dren80_Cf_SF   GTGAAGCATT GTGGTGACGT CCTTCATACT AATTGTTTCT CTTCCTGTTT
Dren81_Cf_SF   GTGAAGCATT GTGGTGACGT CCTTCATACT AATTGTTTCT CTTCCTGTTT
Dren82_Cb_Co   GTGAAGCATT GTGGTGACGT CCTTCATACT AATTGTTTCT CTTCCTGTTT
Dren83_Cb_Ch   GTGAAGCATT GTGGTGACGT CCTTCATACT AATTGTTTCT CTTCCTGTTT
Dren84_Cf_SF   GTGAAGCATT GTGGTGACGT CCTTCATACT AATTGTTTCT CTTCCTGTTT
Dren85_Cb_SF   GTGAAGCATT GTGGTGACGT CCTTCATACT AATTGTTTCT CTTCCTGTTT
Dren86_Cb_Co   GTGAAGCATT GTGGTGACGT CCTTCATACT AATTGTTTCT CTTCCTGTTT
Dren87_Cf_BA   GTGAAGCATT GTGGTAACGT CCTTCATACT AATTGTTTCT CTTCCTGTTT
Dren88_Lg_SF   GTGAAGCATT GTGGTGACGT CCTTCATACT AATTGTTTCT CTTCCTGTTT

               ....|....| ....|....| ....|....| ....|.
                  555        565        575        585
Dren01_Cf_BA   TAGCAGGGGG TATTACAATA CTTTTAACTG ATCGGA
Dren02_Cf_BA   TGGCAGGGGG TATCACAATA CTTTTAACTG ATCGGA
Dren03_Cf_BA   TAGCAGGGGG TATTACAATA CTTTTAACTG ATCGGA
Dren05_Cf_BA   TAGCAGGGGG TATTACAATA CTTTTAACTG ATCGGA
Dren06_Cf_BA   TAGCAGGGGG TATTACAATA CTTTTAACTG ATCGGA
Dren07_Cf_BA   TAGCAGGGGG TATTACAATA CTTTTAACTG ATCGGA
Dren08_Cf_BA   TAGCAGGGGG TATTACAATA CTTTTAACTG ATCGGA
Dren09_Cf_BA   TGGCAGGGGG TATCACAATA CTTTTAACTG ATCGGA
Dren10_Cf_BA   TAGCAGGGGG TATTACAATA CTTTTAACTG ATCGGA
Dren11_Cf_BA   TGGCAGGGGG TATCACAATA CTTTTAACTG ATCGGA
Dren18_Cf_BA   TAGCAGGGGG TATTACAATA CTTTTAACTG ATCGGA
Dren19_Cf_BA   TAGCAGGGGG TATTACAATA CTTTTAACTG ATCGGA
Dren23_Cf_BA   TGGCAGGGGG TATCACAATA CTTTTAACTG ATCGGA
Dren25_Cf_SF   TGGCAGGGGG TATCACAATA CTTTTAACTG ATCGGA
Dren32_Cf_SC   TGGCAGGGGG TATCACAATA CTTTTAACTG ATCGGA
Dren33_Cf_SC   TGGCAGGGGG TATCACAATA CTTTTAACTG ATCGGA
Dren34_Cf_Pa   TGGCAGGGGG TATCACAATA CTTTTAACTG ATCGGA
```

```
Dren35_Cf_Pa   TGGCAGGGGG TATCACAATA CTTTTAACTG ATCGGA
Dren36_Cf_SC   TGGCAGGGGG TATCACAATA CTTTTAACTG ATCGGA
Dren40_Cf_RG   TAGCAGGGGG TATTACAATA CTTTTAACTG ATCGGA
Dren41_Cf_RG   TAGCAGGGGG TATTACAATA CTTTTAACTG ATCGGA
Dren42_Cf_RG   TAGCAGGGGG TATTACAATA CTTTTAACTG ATCGGA
Dren43_Cf_RG   TAGCAGGGGG TATCACAATA CTTTTAACTG ATCGGA
Dren45_Cf_RG   TAGCAGGGGG TATCACAATA CTTTTAACTG ATCGGA
Dren46_Cf_SF   TGGCAGGGGG TATCACAATA CTTTTAACTG ATCGGA
Dren47_Cb_SF   TAGCAGGGGG TATTACAATA CTTTTAACTG ATCGGA
Dren48_Cf_BA   TAGCAGGGGG TATTACAATA CTTTTAACTG ATCGGA
Dren49_Cf_BA   TGGCAGGGGG TATCACAATA CTTTTAACTG ATCGGA
Dren50_Cf_Ch   TAGCAGGGGG TATTACAATA CTTTTAACTG ATCGGA
Dren53_Cf_BA   TAGCAGGGGG TATTACAATA CTTTTAACTG ATCGGA
Dren58_Cb_SF   TAGCAGGGGG TATTACAATA CTTTTAACTG ATCGGA
Dren66_Cf_SF   TAGCAGGGGG TATTACAATA CTTTTAACTG ATCGGA
Dren67_Cf_SF   TAGCAGGGGG TATTACAATA CTTTTAACTG ATCGGA
Dren68_Cf_SF   TAGCAGGGGG TATTACAATA CTTTTAACTG ATCGGA
Dren69_Cf_SF   TAGCAGGGGG TATTACAATA CTTTTAACTG ATCGGA
Dren70_Cf_SF   TGGCAGGGGG TATCACAATA CTTTTAACTG ATCGGA
Dren72_Cb_SF   TAGCAGGGGG TATTACAATA CTTTTAACTG ATCGGA
Dren73_Cb_Ch   TAGCAGGGGG TATTACAATA CTTTTAACTG ATCGGA
Dren74_Cb_Co   TGGCAGGGGG TATCACAATA CTTTTAACTG ATCGGA
Dren75_Cb_Co   TAGCAGGGGG TATTACAATA CTTTTAACTG ATCGGA
Dren80_Cf_SF   TGGCAGGGGG TATCACAATA CTTTTAACTG ATCGGA
Dren81_Cf_SF   TGGCAGGGGG TATCACAATA CTTTTAACTG ATCGGA
Dren82_Cb_Co   TGGCAGGGGG TATCACAATA CTTTTAACTG ATCGGA
Dren83_Cb_Ch   TAGCAGGGGG TATTACAATA CTTTTAACTG ATCGGA
Dren84_Cf_SF   TGGCAGGGGG TATCACAATA CTTTTAACTG ATCGGA
Dren85_Cb_SF   TGGCAGGGGG TATCACAATA CTTTTAACTG ATCGGA
Dren86_Cb_Co   TAGCAGGGGG TATTACAATA CTTTTAACTG ATCGGA
Dren87_Cf_BA   TAGCAGGGGG TATTACAATA CTTTTAACTG ATCGGA
Dren88_Lg_SF   TAGCAGGGGG TATTACAATA CTTTTAACTG ATCGGA
```

COX1-XL Multiple Sequence Alignment

```
                 ....|....| ....|....| ....|....| ....|....| ....|....|
                      5          15         25         35         45
Dren01_Cf_BA     GGTGTGTGAG CAGGGTTTGT TGGGTTGGGG TTATCAGTTA TTATTCGGTA
Dren02_Cf_BA     GGTGTGTGAG CAGGGTTTGT TGGGTTGGGG TTATCAGTTA TTATTCGGTA
Dren03_Cf_BA     GGTGTGTGAG CAGGGTTTGT TGGGTTGGGG TTATCAGTTA TTATTCGGTA
Dren05_Cf_BA     GGTGTGTGAG CAGGGTTTGT TGGGTTGGGG TTATCAGTTA TTATTCGGTA
Dren06_Cf_BA     GGTGTGTGAG CAGGGTTTGT TGGGTTGGGG TTATCAGTTA TTATTCGGTA
Dren07_Cf_BA     GGTGTGTGAG CAGGGTTTGT TGGGTTGGGG TTATCAGTTA TTATTCGGTA
Dren08_Cf_BA     GGTGTGTGAG CAGGGTTTGT TGGGTTGGGG TTATCAGTTA TTATTCGGTA
Dren09_Cf_BA     GGTGTGTGAG CAGGGTTTGT TGGGTTGGGG TTATCAGTTA TTATTCGGTA
Dren10_Cf_BA     GGTGTGTGAG CAGGGTTTGT TGGGTTGGGG TTATCAGTTA TTATTCGGTA
Dren11_Cf_BA     GGTGTGTGAG CAGGGTTTGT TGGGTTGGGG TTATCAGTTA TTATTCGGTA
Dren18_Cf_BA     GGTGTGTGAG CAGGGTTTGT TGGGTTGGGG TTATCAGTTA TTATTCGGTA
Dren19_Cf_BA     GGTGTGTGAG CAGGGTTTGT TGGGTTGGGG TTATCAGTTA TTATTCGGTA
Dren23_Cf_BA     GGTGTGTGAG CAGGGTTTGT TGGGTTGGGG TTATCAGTTA TTATTCGGTA
Dren25_Cf_SF     GGTGTGTGAG CAGGGTTTGT TGGGTTGGGG TTATCAGTTA TTATTCGGTA
Dren32_Cf_SC     GGTGTGTGAG CAGGGTTTGT TGGGTTGGGG TTATCAGTTA TTATTCGGTA
Dren33_Cf_SC     GGTGTGTGAG CAGGGTTTGT TGGGTTGGGG TTATCAGTTA TTATTCGGTA
Dren34_Cf_Pa     GGTGTGTGAG CAGGGTTTGT TGGGTTGGGG TTATCAGTTA TTATTCGGTA
Dren35_Cf_Pa     GGTGTGTGAG CAGGGTTTGT TGGGTTGGGG TTATCAGTTA TTATTCGGTA
Dren40_Cf_RG     GGTGTGTGAG CAGGGTTTGT TGGGTTGGGG TTATCAGTTA TTATTCGGTA
Dren41_Cf_RG     GGTGTGTGAG CAGGGTTTGT TGGGTTGGGG TTATCAGTTA TTATTCGGTA
Dren42_Cf_RG     GGTGTGTGAG CAGGGTTTGT TGGGTTGGGG TTATCAGTTA TTATTCGGTA
Dren43_Cf_RG     GGTGTGTGAG CAGGGTTTGT TGGGTTGGGG TTATCAGTTA TTATTCGGTA
Dren45_Cf_RG     GGTGTGTGAG CAGGGTTTGT TGGGTTGGGG TTATCAGTTA TTATTCGGTA
Dren46_Cf_SF     GGTGTGTGAG CAGGGTTTGT TGGGTTGGGG TTATCAGTTA TTATTCGGTA
Dren47_Cb_SF     GGTGTGTGAG CAGGGTTTGT TGGGTTGGGG TTATCAGTTA TTATTCGGTA
Dren48_Cf_BA     GGTGTGTGAG CAGGGTTTGT TGGGTTGGGG TTATCAGTTA TTATTCGGTA
Dren49_Cf_BA     GGTGTGTGAG CAGGGTTTGT TGGGTTGGGG TTATCAGTTA TTATTCGGTA
Dren50_Cf_Ch     GGTGTGTGAG CAGGGTTTGT TGGGTTGGGG TTATCAGTTA TTATTCGGTA
Dren53_Cf_BA     GGTGTGTGAG CAGGGTTTGT TGGGTTGGGG TTATCAGTTA TTATTCGGTA
Dren58_Cb_SF     GGTGTGTGAG CAGGGTTTGT TGGGTTGGGG TTATCAGTTA TTATTCGGTA
Dren66_Cf_SF     GGTGTGTGAG CAGGGTTTGT TGGGTTGGGG TTATCAGTTA TTATTCGGTA
Dren67_Cf_SF     GGTGTGTGAG CAGGGTTTGT TGGGTTGGGG TTATCAGTTA TTATTCGGTA
Dren68_Cf_SF     GGTGTGTGAG CAGGGTTTGT TGGGTTGGGG TTATCAGTTA TTATTCGGTA
Dren69_Cf_SF     GGTGTGTGAG CAGGGTTTGT TGGGTTGGGG TTATCAGTTA TTATTCGGTA
Dren70_Cf_SF     GGTGTGTGAG CAGGGTTTGT TGGGTTGGGG TTATCAGTTA TTATTCGGTA
Dren72_Cb_SF     GGTGTGTGAG CAGGGTTTGT TGGGTTGGGG TTATCAGTTA TTATTCGGTA
Dren73_Cb_Ch     GGTGTGTGAG CAGGGTTTGT TGGGTTGGGG TTATCAGTTA TTATTCGGTA
Dren74_Cb_Co     GGTGTGTGAG CAGGGTTTGT TGGGTTGGGG TTATCAGTTA TTATTCGGTA
Dren75_Cb_Co     GGTGTGTGAG CAGGGTTTGT TGGGTTGGGG TTATCAGTTA TTATTCGGTA
Dren80_Cf_SF     GGTGTGTGAG CAGGGTTTGT TGGGTTGGGG TTATCAGTTA TTATTCGGTA
Dren81_Cf_SF     GGTGTGTGAG CAGGGTTTGT TGGGTTGGGG TTATCAGTTA TTATTCGGTA
Dren82_Cb_Co     GGTGTGTGAG CAGGGTTTGT TGGGTTGGGG TTATCAGTTA TTATTCGGTA
Dren83_Cb_Ch     GGTGTGTGAG CAGGGTTTGT TGGGTTGGGG TTATCAGTTA TTATTCGGTA
Dren84_Cf_SF     GGTGTGTGAG CAGGGTTTGT TGGGTTGGGG TTATCAGTTA TTATTCGGTA
Dren85_Cb_SF     GGTGTGTGAG CAGGGTTTGT TGGGTTGGGG TTATCAGTTA TTATTCGGTA
Dren86_Cb_Co     GGTGTGTGAG CAGGGTTTGT TGGGTTGGGG TTATCAGTTA TTATTCGGTA
Dren87_Cf_BA     GGTGTGTGAG CAGGGTTTGT TGGGTTGGGG TTATCAGTTA TTATTCGGTA
Dren88_Lg_SF     GGTGTGTGAG CAGGGTTTGT TGGGTTGGGG TTATCAGTTA TTATTCGGTA
```

```
                ....|....| ....|....| ....|....| ....|....| ....|....|
                     55         65         75         85         95
Dren01_Cf_BA    TGTGTTGGCA TCACCGTGGT CTATAGGGTG AAGGCCTTTT ATTAGGCAGT
Dren02_Cf_BA    TGTGTTGGCA TCACCGTGGT CTATAGGGTG AAGGCCTTTT ATTAGGCAGT
Dren03_Cf_BA    TGTGTTGGCA TCACCGTGGT CTATAGGGTG AAGGCCTTTT ATTAGGCAGT
Dren05_Cf_BA    TGTGTTGGCA TCACCGTGGT CTATAGGGTG AAGGCCTTTT ATTAGGCAGT
Dren06_Cf_BA    TGTGTTGGCA TCACCGTGGT CTATAGGGTG AAGGCCTTTT ATTAGGCAGT
Dren07_Cf_BA    TGTGTTGGCA TCACCGTGGT CTATAGGGTG AAGGCCTTTT ATTAGGCAGT
Dren08_Cf_BA    TGTGTTGGCA TCACCGTGGT CTATAGGGTG AAGGCCTTTT ATTAGGCAGT
Dren09_Cf_BA    TGTGTTGGCA TCACCGTGGT CTATAGGGTG AAGGCCTTTT ATTAGGCAGT
Dren10_Cf_BA    TGTGTTGGCA TCACCGTGGT CTATAGGGTG AAGGCCTTTT ATTAGGCAGT
Dren11_Cf_BA    TGTGTTGGCA TCACCGTGGT CTATAGGGTG AAGGCCTTTT ATTAGGCAGT
Dren18_Cf_BA    TGTGTTGGCA TCACCGTGGT CTATAGGGTG AAGGCCTTTT ATTAGGCAGT
Dren19_Cf_BA    TGTGTTGGCA TCACCGTGGT CTATAGGGTG AAGGCCTTTT ATTAGGCAGT
Dren23_Cf_BA    TGTGTTGGCA TCACCGTGGT CTATAGGGTG AAGGCCTTTT ATTAGGCAGT
Dren25_Cf_SF    TGTGTTGGCA TCACCGTGGT CTATAGGGTG AAGGCCTTTT ATTAGGCAGT
Dren32_Cf_SC    TGTGTTGGCA TCACCGTGGT CTATAGGGTG AAGGCCTTTT ATTAGGCAGT
Dren33_Cf_SC    TGTGTTGGCA TCACCGTGGT CTATAGGGTG AAGGCCTTTT ATTAGGCAGT
Dren34_Cf_Pa    TGTGTTGGCA TCACCGTGGT CTATAGGGTG AAGGCCTTTT ATTAGGCAGT
Dren35_Cf_Pa    TGTGTTGGCA TCACCGTGGT CTATAGGGTG AAGGCCTTTT ATTAGGCAGT
Dren40_Cf_RG    CGTGTTGGCA TCACCATGGT CTATAGGGTG AAGGCCTTTT ATTAGGCAGT
Dren41_Cf_RG    CGTGTTGGCA TCACCATGGT CTATAGGGTG AAGGCCTTTT ATTAGGCAGT
Dren42_Cf_RG    TGTGTTGGCA TCACCGTGGT CTATAGGGTG AAGGCCTTTT ATTAGGCAGT
Dren43_Cf_RG    TGTGTTGGCA TCACCGTGGT CTATAGGGTG AAGGCCTTTT ATTAGGCAGT
Dren45_Cf_RG    TGTGTTGGCA TCACCGTGGT CTATAGGGTG AAGGCCTTTT ATTAGGCAGT
Dren46_Cf_SF    TGTGTTGGCA TCACCGTGGT CTATAGGGTG AAGGCCTTTT ATTAGGCAGT
Dren47_Cb_SF    TGTGTTGGCA TCACCGTGGT CTATAGGGTG AAGGCCTTTT ATTAGGCAGT
Dren48_Cf_BA    TGTGTTGGCA TCACCGTGGT CTATAGGGTG AAGGCCTTTT ATTAGGCAGT
Dren49_Cf_BA    TGTGTTGGCA TCACCGTGGT CTATAGGGTG AAGGCCTTTT ATTAGGCAGT
Dren50_Cf_Ch    TGTGTTGGCA TCACCGTGGT CTATAGGGTG AAGGCCTTTT ATTAGGCAGT
Dren53_Cf_BA    TGTGTTGGCA TCACCGTGGT CTATAGGGTG AAGGCCTTTT ATTAGGCAGT
Dren58_Cb_SF    CGTGTTGGCA TCACCATGGT CTATAGGGTG AAGGCCTTTT ATTAGGCAGT
Dren66_Cf_SF    TGTGTTGGCA TCACCGTGGT CTATAGGGTG AAGGCCTTTT ATTAGGCAGT
Dren67_Cf_SF    TGTGTTGGCA TCACCGTGGT CTATAGGGTG AAGGCCTTTT ATTAGGCAGT
Dren68_Cf_SF    TGTGTTGGCA TCACCGTGGT CTATAGGGTG AAGGCCTTTT ATTAGGCAGT
Dren69_Cf_SF    TGTGTTGGCA TCACCGTGGT CTATAGGGTG AAGGCCTTTT ATTAGGCAGT
Dren70_Cf_SF    TGTGTTGGCA TCACCGTGGT CTATAGGGTG AAGGCCTTTT ATTAGGCAGT
Dren72_Cb_SF    TGTGTTGGCA TCACCGTGGT CTATAGGGTG AAGGCCTTTT ATTAGGCAGT
Dren73_Cb_Ch    TGTGTTGGCA TCACCGTGGT CTATAGGGTG AAGGCCTTTT ATTAGGCAGT
Dren74_Cb_Co    TGTGTTGGCA TCACCGTGGT CTATAGGGTG AAGGCCTTTT ATTAGGCAGT
Dren75_Cb_Co    TGTGTTGGCA TCACCGTGGT CTATAGGGTG AAGGCCTTTT ATTAGGCAGT
Dren80_Cf_SF    TGTGTTGGCA TCACCGTGGT CTATAGGGTG AAGGCCTTTT ATTAGGCAGT
Dren81_Cf_SF    TGTGTTGGCA TCACCGTGGT CTATAGGGTG AAGGCCTTTT ATTAGGCAGT
Dren82_Cb_Co    TGTGTTGGCA TCACCGTGGT CTATAGGGTG AAGGCCTTTT ATTAGGCAGT
Dren83_Cb_Ch    TGTGTTGGCA TCACCGTGGT CTATAGGGTG AAGGCCTTTT ATTAGGCAGT
Dren84_Cf_SF    TGTGTTGGCA TCACCGTGGT CTATAGGGTG AAGGCCTTTT ATTAGGCAGT
Dren85_Cb_SF    TGTGTTGGCA TCACCGTGGT CTATAGGGTG AAGGCCTTTT ATTAGGCAGT
Dren86_Cb_Co    TGTGTTGGCA TCACCGTGGT CTATAGGGTG AAGGCCTTTT ATTAGGCAGT
Dren87_Cf_BA    TGTGTTGGCA TCACCGTGGT CTATAGGGTG AAGGCCTTTT ATTAGGCAGT
Dren88_Lg_SF    TGTGTTGGCA TCACCGTGGT CTATAGGGTG AAGGCCTTTT ATTAGGCAGT

                ....|....| ....|....| ....|....| ....|....| ....|....|
                    105        115        125        135        145
```

```
Dren01_Cf_BA   ATTATAATGC TATTATTTCC GCGCATGGCA TTATTATAAT CTTTTATATA
Dren02_Cf_BA   ATTATAATGC TATTATTTCC GCGCATGGCA TTATTATAAT CTTTTATATA
Dren03_Cf_BA   ATTATAATGC TATTATTTCC GCGCATGGCA TTATTATAAT CTTTTATATA
Dren05_Cf_BA   ATTATAATGC TATTATTTCC GCGCATGGCA TTATTATAAT CTTTTATATA
Dren06_Cf_BA   ATTATAATGC TATTATTTCC GCGCATGGCA TTATTATAAT CTTTTATATA
Dren07_Cf_BA   ATTATAATGC TATTATTTCC GCGCACGGCA TTATTATAAT CTTTTATATA
Dren08_Cf_BA   ATTATAATGC TATTATTTCC GCGCATGGCA TTATTATAAT CTTTTATATA
Dren09_Cf_BA   ATTATAATGC TATTATTTCC GCGCATGGCA TTATTATAAT CTTTTATATA
Dren10_Cf_BA   ATTATAATGC TATTATTTCC GCGCATGGCA TTATTATAAT CTTTTATATA
Dren11_Cf_BA   ATTATAATGC TATTATTTCC GCGCATGGCA TTATTATAAT CTTTTATATA
Dren18_Cf_BA   ATTATAATGC TATTATTTCC GCGCATGGCA TTATTATAAT CTTTTATATA
Dren19_Cf_BA   ATTATAATGC TATTATTTCC GCGCATGGCA TTATTATAAT CTTTTATATA
Dren23_Cf_BA   ATTATAATGC TATTATTTCC GCGCATGGCA TTATTATAAT CTTTTATATA
Dren25_Cf_SF   ATTATAATGC TATTATTTCC GCGCATGGCA TTATTATAAT CTTTTATATA
Dren32_Cf_SC   ATTATAATGC TATTATTTCC GCGCATGGCA TTATTATAAT CTTTTATATA
Dren33_Cf_SC   ATTATAATGC TATTATTTCC GCGCATGGCA TTATTATAAT CTTTTATATA
Dren34_Cf_Pa   ATTATAATGC TATTATTTCC GCGCATGGCA TTATTATAAT CTTTTATATA
Dren35_Cf_Pa   ATTATAATGC TATTATTTCC GCGCATGGCA TTATTATAAT CTTTTATATA
Dren40_Cf_RG   ATTATAATGC TATTATTTCC GCGCATGGCA TTATTATAAT CTTTTATATA
Dren41_Cf_RG   ATTATAATGC TATTATTTCC GCGCATGGCA TTATTATAAT CTTTTATATA
Dren42_Cf_RG   ATTATAATGC TATTATTTCC GCGCACGGCA TTATTATAAT CTTTTATATA
Dren43_Cf_RG   ATTATAATGC TATTATTTCC GCGCATGGCA TTATTATAAT CTTTTATATA
Dren45_Cf_RG   ATTATAATGC TATTATTTCC GCGCATGGCA TTATTATAAT CTTTTATATA
Dren46_Cf_SF   ATTATAATGC TATTATTTCC GCGCATGGCA TTATTATAAT CTTTTATATA
Dren47_Cb_SF   ATTATAATGC TATTATTTCC GCGCATGGCA TTATTATAAT CTTTTATATA
Dren48_Cf_BA   ATTATAATGC TATTATTTCC GCGCATGGCA TTATTATAAT CTTTTATATA
Dren49_Cf_BA   ATTATAATGC TATTATTTCC GCGCATGGCA TTATTATAAT CTTTTATATA
Dren50_Cf_Ch   ATTATAATGC TATTATTTCC GCGCATGGCA TTATTATAAT CTTTTATATA
Dren53_Cf_BA   ATTATAATGC TATTATTTCC GCGCATGGCA TTATTATAAT CTTTTATATA
Dren58_Cb_SF   ATTATAATGC TATTATTTCC GCGCATGGCA TTATTATAAT CTTTTATATA
Dren66_Cf_SF   ATTATAATGC TATTATTTCC GCGCATGGCA TTATTATAAT CTTTTATATA
Dren67_Cf_SF   ATTATAATGC TATTATTTCC GCGCATGGCA TTATTATAAT CTTTTATATA
Dren68_Cf_SF   ATTATAATGC TATTATTTCC GCGCATGGCA TTATTATAAT CTTTTATATA
Dren69_Cf_SF   ATTATAATGC TATTATTTCC GCGCATGGCA TTATTATAAT CTTTTATATA
Dren70_Cf_SF   ATTATAATGC TATTATTTCC GCGCATGGCA TTATTATAAT CTTTTATATA
Dren72_Cb_SF   ATTATAATGC TATTATTTCC GCGCATGGCA TTATTATAAT CTTTTATATA
Dren73_Cb_Ch   ATTATAATGC TATTATTTCC GCGCATGGCA TTATTATAAT CTTTTATATA
Dren74_Cb_Co   ATTATAATGC TATTATTTCC GCGCATGGCA TTATTATAAT CTTTTATATA
Dren75_Cb_Co   ATTATAATGC TATTATTTCC GCGCATGGCA TTATTATAAT CTTTTATATA
Dren80_Cf_SF   ATTATAATGC TATTATTTCC GCGCATGGCA TTATTATAAT CTTTTATATA
Dren81_Cf_SF   ATTATAATGC TATTATTTCC GCGCATGGCA TTATTATAAT CTTTTATATA
Dren82_Cb_Co   ATTATAATGC TATTATTTCC GCGCATGGCA TTATTATAAT CTTTTATATA
Dren83_Cb_Ch   ATTATAATGC TATTATTTCC GCGCATGGCA TTATTATAAT CTTTTATATA
Dren84_Cf_SF   ATTATAATGC TATTATTTCC GCGCATGGCA TTATTATAAT CTTTTATATA
Dren85_Cb_SF   ATTATAATGC TATTATTTCC GCGCATGGCA TTATTATAAT CTTTTATATA
Dren86_Cb_Co   ATTATAATGC TATTATTTCC GCGCACGGCA TTATTATAAT CTTTTATATA
Dren87_Cf_BA   ATTATAATGC TATTATTTCC GCGCATGGCA TTATTATAAT CTTTTATATA
Dren88_Lg_SF   ATTATAATGC TATTATTTCC GCGCATGGCA TTATTATAAT CTTTTATATA

               ....|....| ....|....| ....|....| ....|....| ....|....|
                   155        165        175        185        195
Dren01_Cf_BA   ATGATGCCTA TTATAATAGG TGGGATGGGT AATATTATAG TGCCTGTTAT
Dren02_Cf_BA   ATGATGCCTA TTATAATAGG TGGGATGGGT AATATTATAG TGCCTGTTAT
Dren03_Cf_BA   ATGATGCCTA TTATAATAGG TGGGATGGGT AATATTATAG TGCCTGTTAT
```

```
Dren05_Cf_BA   ATAATGCCTA TTATAATAGG TGGGATGGGT AATATTATAG TGCCTGTTAT
Dren06_Cf_BA   ATGATGCCTA TTATAATAGG TGGGATGGGT AATATTATAG TGCCTGTTAT
Dren07_Cf_BA   ATGATGCCTA TTATAATAGG TGGGATGGGT AATATTATAG TGCCTGTTAT
Dren08_Cf_BA   ATGATGCCTA TTATAATAGG TGGGATGGGT AATATTATAG TGCCTGTTAT
Dren09_Cf_BA   ATGATGCCTA TTATAATAGG TGGGATGGGT AATATTATAG TGCCTGTTAT
Dren10_Cf_BA   ATGATGCCTA TTATAATAGG TGGGATGGGT AATATTATAG TGCCTGTTAT
Dren11_Cf_BA   ATGATGCCTA TTATAATAGG TGGGATGGGT AATATTATAG TGCCTGTTAT
Dren18_Cf_BA   ATGATGCCTA TTATAATAGG TGGGATGGGT AATATTATAG TGCCTGTTAT
Dren19_Cf_BA   ATGATGCCTA TTATAATAGG TGGGATGGGT AATATTATAG TGCCTGTTAT
Dren23_Cf_BA   ATGATGCCTA TTATAATAGG TGGGGTGGGT AATATTATAG TGCCTGTTAT
Dren25_Cf_SF   ATGATGCCTA TTATAATAGG TGGGATGGGT AATATTATAG TGCCTGTTAT
Dren32_Cf_SC   ATGATGCCTA TTATAATAGG TGGGATGGGT AATATTATAG TGCCTGTTAT
Dren33_Cf_SC   ATGATGCCTA TTATAATAGG TGGGATGGGT AATATTATAG TGCCTGTTAT
Dren34_Cf_Pa   ATGATGCCTA TTATAATAGG TGGGATGGGT AATATTATAG TGCCTGTTAT
Dren35_Cf_Pa   ATGATGCCTA TTATAATAGG TGGGATGGGT AATATTATAG TGCCTGTTAT
Dren40_Cf_RG   ATGATGCCTA TTATAATAGG TGGGATGGGT AATATTATAG TGCCTGTTAT
Dren41_Cf_RG   ATGATGCCTA TTATAATAGG TGGGATGGGT AATATTATAG TGCCTGTTAT
Dren42_Cf_RG   ATGATGCCTA TTATAATAGG TGGGATGGGT AATATTATAG TGCCTGTTAT
Dren43_Cf_RG   ATGATGCCTA TTATAATAGG TGGGATGGGT AATATTATAG TGCCTGTTAT
Dren45_Cf_RG   ATGATGCCTA TTATAATAGG TGGGATGGGT AATATTATAG TGCCTGTTAT
Dren46_Cf_SF   ATGATGCCTA TTATAATAGG TGGGATGGGT AATATTATAG TGCCTGTTAT
Dren47_Cb_SF   ATAATGCCTA TTATAATAGG TGGGATGGGT AATATTATAG TGCCTGTTAT
Dren48_Cf_BA   ATGATGCCTA TTATAATAGG TGGGATGGGT AATATTATAG TGCCTGTTAT
Dren49_Cf_BA   ATGATGCCTA TTATAATAGG TGGGGTGGGT AATATTATAG TGCCTGTTAT
Dren50_Cf_Ch   ATGATGCCTA TTATAATAGG TGGGATGGGT AATATTATAG TGCCTGTTAT
Dren53_Cf_BA   ATGATGCCTA TTATAATAGG TGGGATGGGT AATATTATAG TGCCTGTTAT
Dren58_Cb_SF   ATGATGCCTA TTATAATAGG TGGGGTGGGT AATGTTATAG TGCCTGTTAT
Dren66_Cf_SF   ATGATGCCTA TTATAATAGG TGGGATGGGT AATATTATAG TGCCTGTTAT
Dren67_Cf_SF   ATAATGCCTA TTATAATAGG TGGGATGGGT AATATTATAG TGCCTGTTAT
Dren68_Cf_SF   ATAATGCCTA TTATAATAGG TGGGATGGGT AATATTATAG TGCCTGTTAT
Dren69_Cf_SF   ATAATGCCTA TTATAATAGG TGGGATGGGT AATATTATAG TGCCTGTTAT
Dren70_Cf_SF   ATGATGCCTA TTATAATAGG TGGGATGGGT AATATTATAG TGCCTGTTAT
Dren72_Cb_SF   ATGATGCCTA TTATAATAGG TGGGATGGGT AATATTATAG TGCCTGTTAT
Dren73_Cb_Ch   ATAATGCCTA TTATAATAGG TGGGATGGGT AATATTATAG TGCCTGTTAT
Dren74_Cb_Co   ATGATGCCTA TTATAATAGG TGGGATGGGT AATATTATAG TGCCTGTTAT
Dren75_Cb_Co   ATGATGCCTA TTATAATAGG TGGGATGGGT AATATTATAG TGCCTGTTAT
Dren80_Cf_SF   ATGATGCCTA TTATAATAGG TGGGATGGGT AATATTATAG TGCCTGTTAT
Dren81_Cf_SF   ATGATGCCTA TTATAATAGG TGGGATGGGT AATATTATAG TGCCTGTTAT
Dren82_Cb_Co   ATGATGCCTA TTATAATAGG TGGGATGGGT AATATTATAG TGCCTGTTAT
Dren83_Cb_Ch   ATGATGCCTA TTATAATAGG TGGGATGGGT AATATTATAG TGCCTGTTAT
Dren84_Cf_SF   ATGATGCCTA TTATAATAGG TGGGATGGGT AATATTATAG TGCCTGTTAT
Dren85_Cb_SF   ATGATGCCTA TTATAATAGG TGGGGTGGGT AATATTATAG TGCCTGTTAT
Dren86_Cb_Co   ATGATGCCTA TTATAATAGG TGGGATGGGT AATATTATAG TGCCTGTTAT
Dren87_Cf_BA   ATAATGCCTA TTATAATAGG TGGGATGGGT AATGTTATAG TGCCTGTTAT
Dren88_Lg_SF   ATAATGCCTA TTATAATAGG TGGGATGGGT AATATTATAG TGCCTGTTAT

               ....|....| ....|....| ....|....| ....|....| ....|....|
                    205        215        225        235        245
Dren01_Cf_BA   GTTGGGGCTT CCTGATATAG CTTTTCCACG TCTTAATAAT TTGGGGTTTT
Dren02_Cf_BA   GTTGGGGCTT CCTGATATAG CTTTTCCACG TCTTAATAAT TTGGGGTTTT
Dren03_Cf_BA   GTTGGGGCTT CCTGATATAG CTTTTCCACG TCTTAATAAT TTGGGGTTTT
Dren05_Cf_BA   GTTGGGGCTT CCTGATATAG CTTTTCCACG TCTTAATAAT TTGGGGTTTT
Dren06_Cf_BA   GTTGGGGCTT CCTGATATAG CTTTTCCACG TCTTAATAAT TTGGGGTTTT
Dren07_Cf_BA   GTTGGGGCTT CCTGATATAG CTTTTCCACG TCTTAATAAT TTGGGGTTTT
```

```
Dren08_Cf_BA   GTTGGGGCTT CCTGATATAG CTTTTCCACG TCTTAATAAT TTGGGGTTTT
Dren09_Cf_BA   GTTGGGGCTT CCTGATATAG CTTTTCCACG TCTTAATAAT TTGGGGTTTT
Dren10_Cf_BA   GTTGGGGCTT CCTGATATAG CTTTTCCACG TCTTAATAAT TTGGGGTTTT
Dren11_Cf_BA   GTTGGGGCTT CCTGATATAG CTTTTCCACG TCTTAATAAT TTGGGGTTTT
Dren18_Cf_BA   GTTGGGGCTT CCTGATATAG CTTTTCCACG TCTTAATAAT TTGGGGTTTT
Dren19_Cf_BA   GTTGGGGCTT CCTGATATAG CTTTTCCACG TCTTAATAAT TTGGGGTTTT
Dren23_Cf_BA   GTTGGGGCTT CCTGATATAG CTTTTCCACG TCTTAATAAT TTGGGGTTTT
Dren25_Cf_SF   GTTGGGGCTT CCTGATATAG CTTTTCCACG TCTTAATAAT TTGGGGTTTT
Dren32_Cf_SC   GTTGGGGCTT CCTGATATAG CTTTTCCACG TCTTAATAAT TTGGGGTTTT
Dren33_Cf_SC   GTTGGGGCTT CCTGATATAG CTTTTCCACG TCTTAATAAT TTGGGGTTTT
Dren34_Cf_Pa   GTTGGGGCTT CCTGATATAG CTTTTCCACG TCTTAATAAT TTGGGGTTTT
Dren35_Cf_Pa   GTTGGGGCTT CCTGATATAG CTTTTCCACG TCTTAATAAT TTGGGGTTTT
Dren40_Cf_RG   GTTGGGGCTT CCTGATATAG CTTTTCCACG TCTTAATAAT TTGGGGTTTT
Dren41_Cf_RG   GTTGGGGCTT CCTGATATAG CTTTTCCACG TCTTAATAAT TTGGGGTTTT
Dren42_Cf_RG   GTTGGGGCTT CCTGATATAG CTTTTCCACG TCTTAATAAT TTGGGGTTTT
Dren43_Cf_RG   GTTGGGGCTT CCTGATATAG CTTTTCCACG TCTTAATAAT TTGGGGTTTT
Dren45_Cf_RG   GTTGGGGCTT CCTGATATAG CTTTTCCACG TCTTAATAAT TTGGGGTTTT
Dren46_Cf_SF   GTTGGGGCTT CCTGATATAG CTTTTCCACG TCTTAATAAT TTGGGGTTTT
Dren47_Cb_SF   GTTGGGGCTT CCTGATATAG CTTTTCCACG TCTTAATAAT TTGGGGTTTT
Dren48_Cf_BA   GTTGGGGCTT CCTGATATAG CTTTTCCACG TCTTAATAAT TTGGGGTTTT
Dren49_Cf_BA   GTTGGGGCTT CCTGATATAG CTTTTCCACG TCTTAATAAT TTGGGGTTTT
Dren50_Cf_Ch   GTTGGGGCTT CCTGATATAG CTTTTCCACG TCTTAATAAT TTGGGGTTTT
Dren53_Cf_BA   GTTGGGGCTT CCTGATATAG CTTTTCCACG TCTTAATAAT TTGGGGTTTT
Dren58_Cb_SF   GTTGGGGCTT CCTGATATAG CTTTTCCACG TCTTAATAAT TTGGGGTTTT
Dren66_Cf_SF   GTTGGGGCTT CCTGATATAG CTTTTCCACG TCTTAATAAT TTGGGGTTTT
Dren67_Cf_SF   GTTGGGGCTT CCTGATATAG CTTTTCCACG TCTTAATAAT TTGGGGTTTT
Dren68_Cf_SF   GTTGGGGCTT CCTGATATAG CTTTTCCACG TCTTAATAAT TTGGGGTTTT
Dren69_Cf_SF   GTTGGGGCTT CCTGATATAG CTTTTCCACG TCTTAATAAT TTGGGGTTTT
Dren70_Cf_SF   GTTGGGGCTT CCTGATATAG CTTTTCCACG TCTTAATAAT TTGGGGTTTT
Dren72_Cb_SF   GTTGGGGCTT CCTGATATAG CTTTTCCACG TCTTAATAAT TTGGGGTTTT
Dren73_Cb_Ch   GTTGGGGCTT CCTGATATAG CTTTTCCACG TCTTAATAAT TTGGGGTTTT
Dren74_Cb_Co   GTTGGGGCTT CCTGATATAG CTTTTCCACG TCTTAATAAT TTGGGGTTTT
Dren75_Cb_Co   GTTGGGGCTT CCTGATATAG CTTTTCCACG TCTTAATAAT TTGGGGTTTT
Dren80_Cf_SF   GTTGGGGCTT CCTGATATAG CTTTTCCACG TCTTAATAAT TTGGGGTTTT
Dren81_Cf_SF   GTTGGGGCTT CCTGATATAG CTTTTCCACG TCTTAATAAT TTGGGGTTTT
Dren82_Cb_Co   GTTGGGGCTT CCTGATATAG CTTTTCCACG TCTTAATAAT TTGGGGTTTT
Dren83_Cb_Ch   GTTGGGGCTT CCTGATATAG CTTTTCCACG TCTTAATAAT TTGGGGTTTT
Dren84_Cf_SF   GTTGGGGCTT CCTGATATAG CTTTTCCACG TCTTAATAAT TTGGGGTTTT
Dren85_Cb_SF   GTTGGGGCTT CCTGATATAG CTTTTCCACG TCTTAATAAT TTGGGGTTTT
Dren86_Cb_Co   GTTGGGGCTT CCTGATATAG CTTTTCCACG TCTTAATAAT TTGGGGTTTT
Dren87_Cf_BA   GTTGGGGCTT CCTGATATAG CTTTTCCACG TCTTAATAAT TTGGGGTTTT
Dren88_Lg_SF   GTTGGGGCTT CCTGATATAG CTTTTCCACG TCTTAATAAT TTGGGGTTTT

               ....|....| ....|....| ....|....| ....|....| ....|....|
                    255        265        275        285        295
Dren01_Cf_BA   GGTTGTTGCC TCCTTCATTC TTATTGTTTG TAAGTTCCAT AATGTTTTCG
Dren02_Cf_BA   GGTTATTGCC TCCTTCATTC TTATTGTTTG TAAGTTCCAT AATGTTTTCG
Dren03_Cf_BA   GGTTGTTGCC TCCTTCATTC TTATTGTTTG TAAGTTCCAT AATGTTTTCG
Dren05_Cf_BA   GGTTATTGCC TCCTTCATTC TTATTGTTTG TAAGTTCCAT AATGTTTTCG
Dren06_Cf_BA   GGTTGTTGCC TCCTTCATTC TTATTGTTTG TAAGTTCCAT AATGTTTTCG
Dren07_Cf_BA   GGTTATTGCC TCCTTCATTC TTATTGTTTG TAAGTTCCAT AATGTTTTCG
Dren08_Cf_BA   GGTTGTTGCC TCCTTCATTC TTATTGTTTG TAAGTTCCAT AATGTTTTCG
Dren09_Cf_BA   GGTTATTGCC TCCTTCATTC TTATTGTTTG TAAGTTCCAT AATGTTTTCG
Dren10_Cf_BA   GGTTGTTGCC TCCTTCATTC TTATTGTTTG TAAGTTCCAT AATGTTTTCG
```

```
Dren11_Cf_BA   GGTTATTGCC TCCTTCATTC TTATTGTTTG TAAGTTCCAT AATGTTTTCG
Dren18_Cf_BA   GGTTATTGCC TCCTTCATTC TTATTGTTTG TAAGTTCCAT AATGTTTTCG
Dren19_Cf_BA   GGTTATTGCC TCCTTCATTC TTATTGTTTG TAAGTTCCAT AATGTTTTCG
Dren23_Cf_BA   GGTTATTGCC TCCTTCATTC TTATTGTTTG TAAGTTCCAT AATGTTTTCG
Dren25_Cf_SF   GGTTATTGCC TCCTTCATTC TTATTGTTTG TAAGTTCCAT AATGTTTTCG
Dren32_Cf_SC   GGTTATTGCC TCCTTCATTC TTATTGTTTG TAAGTTCCAT AATGTTTTCG
Dren33_Cf_SC   GGTTATTGCC TCCTTCATTC TTATTGTTTG TAAGTTCCAT AATGTTTTCG
Dren34_Cf_Pa   GGTTATTGCC TCCTTCATTC TTATTGTTTG TAAGTTCCAT AATGTTTTCG
Dren35_Cf_Pa   GGTTATTGCC TCCTTCATTC TTATTGTTTG TAAGTTCCAT AATGTTTTCG
Dren40_Cf_RG   GGTTATTGCC TCCTTCATTC TTATTGTTTG TAAGTTCCAC GATGTTTTCG
Dren41_Cf_RG   GGTTATTGCC TCCTTCATTC TTATTGTTTG TAAGTTCCAC GATGTTTTCG
Dren42_Cf_RG   GGTTATTGCC TCCTTCATTC TTATTGTTTG TAAGTTCCAT AATGTTTTCG
Dren43_Cf_RG   GGTTATTGCC TCCTTCATTC TTATTGTTTG TAAGTTCCAT AATGTTTTCG
Dren45_Cf_RG   GGTTATTGCC TCCTTCATTC TTATTGTTTG TAAGTTCCAT AATGTTTTCG
Dren46_Cf_SF   GGTTATTGCC TCCTTCATTC TTATTGTTTG TAAGTTCCAT AATGTTTTCG
Dren47_Cb_SF   GGTTATTGCC TCCTTCATTC TTATTGTTTG TAAGTTCCAT AATGTTTTCG
Dren48_Cf_BA   GGTTGTTGCC TCCTTCATTC TTATTGTTTG TAAGTTCCAT AATGTTTTCG
Dren49_Cf_BA   GGTTATTGCC TCCTTCATTC TTATTGTTTG TAAGTTCCAT AATGTTTTCG
Dren50_Cf_Ch   GGTTGTTGCC TCCTTCATTC TTATTGTTTG TAAGTTCCAT AATGTTTTCG
Dren53_Cf_BA   GGTTGTTGCC TCCTTCATTC TTATTGTTTG TAAGTTCCAT AATGTTTTCG
Dren58_Cb_SF   GGTTATTGCC TCCTTCATTC TTATTGTTTG TAAGTTCCAT GATGTTTTCG
Dren66_Cf_SF   GGTTATTGCC TCCTTCATTC TTATTGTTTG TAAGTTCCAT AATGTTTTCG
Dren67_Cf_SF   GGTTATTGCC TCCTTCATTC TTATTGTTTG TAAGTTCCAT AATGTTTTCG
Dren68_Cf_SF   GGTTATTGCC TCCTTCATTC TTATTGTTTG TAAGTTCCAT AATGTTTTCG
Dren69_Cf_SF   GGTTATTGCC TCCTTCATTC TTATTGTTTG TAAGTTCCAT AATGTTTTCG
Dren70_Cf_SF   GGTTATTGCC TCCTTCATTC TTATTGTTTG TAAGTTCCAT AATGTTTTCG
Dren72_Cb_SF   GGTTGTTGCC TCCTTCATTC TTATTGTTTG TAAGTTCCAT AATGTTTTCG
Dren73_Cb_Ch   GGTTATTGCC TCCTTCATTC TTATTGTTTG TAAGTTCCAT AATGTTTTCG
Dren74_Cb_Co   GGTTATTGCC TCCTTCATTC TTATTGTTTG TAAGTTCCAT AATGTTTTCG
Dren75_Cb_Co   GGTTGTTGCC TCCTTCATTC TTATTGTTTG TAAGTTCCAT AATGTTTTCG
Dren80_Cf_SF   GGTTATTGCC TCCTTCATTC TTATTGTTTG TAAGTTCCAT AATGTTTTCG
Dren81_Cf_SF   GGTTATTGCC TCCTTCATTC TTATTGTTTG TAAGTTCCAT AATGTTTTCG
Dren82_Cb_Co   GGTTATTGCC TCCTTCATTC TTATTGTTTG TAAGTTCCAT AATGTTTTCG
Dren83_Cb_Ch   GGTTGTTGCC TCCTTCATTC TTATTGTTTG TAAGTTCCAT AATGTTTTCG
Dren84_Cf_SF   GGTTATTGCC TCCTTCATTC TTATTGTTTG TAAGTTCCAT AATGTTTTCG
Dren85_Cb_SF   GGTTATTGCC TCCTTCATTC TTATTGTTTG TAAGTTCCAT AATGTTTTCG
Dren86_Cb_Co   GGTTATTGCC TCCTTCATTC TTATTGTTTG TAAGTTCCAT AATGTTTTCG
Dren87_Cf_BA   GGTTATTGCC TCCTTCATTC TTATTGTTTG TAAGTTCCAT AATGTTTTCG
Dren88_Lg_SF   GGTTATTGCC TCCTTCATTC TTATTGTTTG TAAGTTCCAT AATGTTTTCG

               ....|....| ....|....| ....|....| ....|....| ....|....|
                   305        315        325        335        345
Dren01_Cf_BA   GTTTCTGGCC CGGGCACAGG TTGAACCCTT TACCCTCCTC TATCTTTATT
Dren02_Cf_BA   GTTTCTGGCC CGGGCACAGG TTGAACCCTT TACCCTCCTT TATCTTTATT
Dren03_Cf_BA   GTTTCTGGCC CGGGCACAGG TTGAACCCTT TACCCTCCTC TATCTTTATT
Dren05_Cf_BA   GTTTCTGGCC CGGGCACAGG TTGAACCCTT TACCCTCCTC TATCTTTATT
Dren06_Cf_BA   GTTTCTGGCC CGGGCACAGG TTGAACCCTT TACCCTCCTC TATCTTTATT
Dren07_Cf_BA   GTTTCTGGCC CGGGCACAGG TTGAACCCTT TACCCTCCTC TATCTTTATT
Dren08_Cf_BA   GTTTCTGGCC CGGGCACAGG TTGAACCCTT TACCCTCCTC TATCTTTATT
Dren09_Cf_BA   GTTTCTGGCC CGGGCACAGG TTGGACCCTT TACCCTCCTC TATCTTTATT
Dren10_Cf_BA   GTTTCTGGCC CGGGCACAGG TTGAACCCTT TACCCTCCTC TATCTTTATT
Dren11_Cf_BA   GTTTCTGGCC CGGGCACAGG TTGGACCCTT TACCCTCCTC TATCTTTATT
Dren18_Cf_BA   GTTTCTGGCC CGGGCACAGG TTGAACCCTT TACCCTCCTC TATCTTTATT
Dren19_Cf_BA   GTTTCTGGCC CGGGCACAGG TTGAACCCTT TACCCTCCTC TATCTTTATT
```

```
Dren23_Cf_BA   GTTTCTGGCC CGGGCACAGG TTGGACCCTC TACCCTCCTC TATCTTTATT
Dren25_Cf_SF   GTTTCTGGCC CGGGCACAGG TTGGACCCTT TACCCTCCTC TATCTTTATT
Dren32_Cf_SC   GTTTCTGGCC CGGGCACAGG TTGGACCCTT TACCCTCCTC TATCTTTATT
Dren33_Cf_SC   GTTTCTGGCC CGGGCACAGG TTGGACCCTT TACCCTCCTC TATCTTTATT
Dren34_Cf_Pa   GTTTCTGGCC CGGGCACAGG TTGGACCCTT TACCCTCCTC TATCTTTATT
Dren35_Cf_Pa   GTTTCTGGCC CGGGCACAGG TTGGACCCTT TACCCTCCTC TATCTTTATT
Dren40_Cf_RG   GTTTCTGGCC CGGGCACAGG TTGAACCCTT TACCCTCCTC TATCTTTATT
Dren41_Cf_RG   GTTTCTGGCC CGGGCACAGG TTGAACCCTT TACCCTCCTC TATCTTTATT
Dren42_Cf_RG   GTTTCTGGCC CGGGCACAGG TTGAACCCTT TACCCTCCTC TATCTTTATT
Dren43_Cf_RG   GTTTCTGGCC CGGGCACAGG TTGAACCCTT TACCCTCCTC TATCTTTATT
Dren45_Cf_RG   GTTTCTGGCC CGGGCACAGG TTGAACCCTT TACCCTCCTC TATCTTTATT
Dren46_Cf_SF   GTTTCTGGCC CGGGCACAGG TTGGACCCTT TACCCTCCTC TATCTTTATT
Dren47_Cb_SF   GTTTCTGGCC CGGGCACAGG TTGAACCCTT TACCCTCCTC TATCTTTATT
Dren48_Cf_BA   GTTTCTGGCC CGGGCACAGG TTGAACCCTT TACCCTCCTC TATCTTTATT
Dren49_Cf_BA   GTTTCTGGCC CGGGCACAGG TTGGACCCTC TACCCTCCTC TATCTTTATT
Dren50_Cf_Ch   GTTTCTGGCC CGGGCACAGG TTGAACCCTT TACCCTCCTC TATCTTTATT
Dren53_Cf_BA   GTTTCTGGCC CGGGCACAGG TTGAACCCTT TACCCTCCTC TATCTTTATT
Dren58_Cb_SF   GTTTCTGGCC CGGGCACAGG TTGAACCCTT TACCCTCCTC TATCTTTATT
Dren66_Cf_SF   GTTTCTGGCC CGGGCACAGG TTGAACCCTT TACCCTCCTC TATCTTTATT
Dren67_Cf_SF   GTTTCTGGCC CGGGCACAGG TTGAACCCTT TACCCTCCTC TATCTTTATT
Dren68_Cf_SF   GTTTCTGGCC CGGGCACAGG TTGAACCCTT TACCCTCCTC TATCTTTATT
Dren69_Cf_SF   GTTTCTGGCC CGGGCACAGG TTGAACCCTT TACCCTCCTC TATCTTTATT
Dren70_Cf_SF   GTTTCTGGCC CGGGCACAGG TTGGACCCTT TACCCTCCTC TATCTTTATT
Dren72_Cb_SF   GTTTCTGGCC CGGGCACAGG TTGAACCCTT TACCCTCCTC TATCTTTATT
Dren73_Cb_Ch   GTTTCTGGCC CGGGCACAGG TTGAACCCTT TACCCTCCTC TATCTTTATT
Dren74_Cb_Co   GTTTCTGGCC CGGGCACAGG TTGGACCCTT TACCCTCCTC TATCTTTATT
Dren75_Cb_Co   GTTTCTGGCC CGGGCACAGG TTGAACCCTT TACCCTCCTC TATCTTTATT
Dren80_Cf_SF   GTTTCTGGCC CGGGCACAGG TTGGACCCTT TACCCTCCTC TATCTTTATT
Dren81_Cf_SF   GTTTCTGGCC CGGGCACAGG TTGGACCCTT TACCCTCCTC TATCTTTATT
Dren82_Cb_Co   GTTTCTGGCC CGGGCACAGG TTGGACCCTT TACCCTCCTC TATCTTTATT
Dren83_Cb_Ch   GTTTCTGGCC CGGGCACAGG TTGAACCCTT TACCCTCCTC TATCTTTATT
Dren84_Cf_SF   GTTTCTGGCC CGGGCACAGG TTGGACCCTT TACCCTCCTC TATCTTTATT
Dren85_Cb_SF   GTTTCTGGCC CGGGCACAGG TTGGACCCTC TACCCTCCTC TATCTTTATT
Dren86_Cb_Co   GTTTCTGGCC CGGGCACAGG TTGAACCCTT TACCCTCCTC TATCTTTATT
Dren87_Cf_BA   GTTTCTGGCC CGGGCACAGG TTGAACCCTT TACCCTCCTC TATCTTTATT
Dren88_Lg_SF   GTTTCTGGCC CGGGCACAGG TTGAACCCTT TACCCTCCTC TATCTTTATT

               ....|....| ....|....| ....|....| ....|....| ....|....|
                    355        365        375        385        395
Dren01_Cf_BA   TACTGGGCAT CCTGATGTTT GCGTGGATTT TGTGATCTTT TCTCTACATT
Dren02_Cf_BA   TACTGGGCAT CCTGATGTTT GCGTGGATTT TGTGATCTTT TCTCTACATT
Dren03_Cf_BA   TACTGGGCAT CCTGATGTTT GCGTGGATTT TGTGATCTTT TCTCTACATT
Dren05_Cf_BA   TACTGGGCAT CCTGATGTTT GCGTGGATTT TGTGATCTTT TCTCTACATT
Dren06_Cf_BA   TACTGGGCAT CCTGATGTTT GCGTGGATTT TGTGATCTTT TCTCTACATT
Dren07_Cf_BA   TACTGGGCAT CCTGATGTTT GCGTGGATTT TGTGATCTTT TCTCTACATT
Dren08_Cf_BA   TACTGGGCAT CCTGATGTTT GCGTGGATTT TGTGATCTTT TCTCTACATT
Dren09_Cf_BA   TACTGGGCAT CCTGATGTTT GCGTGGATTT TGTGATCTTT TCTCTACATT
Dren10_Cf_BA   TACTGGGCAT CCTGATGTTT GCGTGGATTT TGTGATCTTT TCTCTACATT
Dren11_Cf_BA   TACTGGGCAT CCTGATGTTT GCGTGGATTT TGTGATCTTT TCTCTACATT
Dren18_Cf_BA   TACTGGACAT CCTGATGTCT GCGTGGATTT TGTGATCTTT TCTCTACATT
Dren19_Cf_BA   TACTGGACAT CCTGATGTCT GCGTGGATTT TGTGATCTTT TCTCTACATT
Dren23_Cf_BA   TACTGGGCAT CCTGATGTTT GCGTGGATTT TGTGATCTTT TCTCTACATT
Dren25_Cf_SF   TACTGGGCAT CCTGATGTTT GCGTGGATTT TGTGATCTTT TCTCTACATT
Dren32_Cf_SC   TACTGGGCAT CCTGATGTTT GCGTGGATTT TGTGATCTTT TCTCTACATT
```

```
Dren33_Cf_SC   TACTGGGCAT CCTGATGTTT GCGTGGATTT TGTGATCTTT TCTCTACATT
Dren34_Cf_Pa   TACTGGGCAT CCTGATGTTT GCGTGGATTT TGTGATCTTT TCTCTACATT
Dren35_Cf_Pa   TACTGGGCAT CCTGATGTTT GCGTGGATTT TGTGATCTTT TCTCTACATT
Dren40_Cf_RG   TACTGGGCAT CCTGATGTTT GCGTGGATTT TGTGATCTTT TCTCTACATT
Dren41_Cf_RG   TACTGGGCAT CCTGATGTTT GCGTGGATTT TGTGATCTTT TCTCTACATT
Dren42_Cf_RG   TACTGGGCAT CCTGATGTTT GCGTGGATTT TGTGATCTTT TCTCTACATT
Dren43_Cf_RG   TACTGGGCAT CCTGATGTTT GCGTGGATTT TGTGATCTTT TCTCTACATT
Dren45_Cf_RG   TACTGGGCAT CCTGATGTTT GCGTGGATTT TGTGATCTTT TCTCTACATT
Dren46_Cf_SF   TACTGGGCAT CCTGATGTTT GCGTGGATTT TGTGATCTTT TCTCTACATT
Dren47_Cb_SF   TACTGGGCAT CCTGATGTTT GCGTGGATTT TGTGATCTTT TCTCTACATT
Dren48_Cf_BA   TACTGGGCAT CCTGATGTTT GCGTGGATTT TGTGATCTTT TCTCTACATT
Dren49_Cf_BA   TACTGGGCAT CCTGATGTTT GCGTGGATTT TGTGATCTTT TCTCTACATT
Dren50_Cf_Ch   TACTGGGCAT CCTGATGTTT GCGTGGATTT TGTGATCTTT TCTCTACATT
Dren53_Cf_BA   TACTGGACAT CCTGATGTTT GCGTGGATTT TGTGATCTTT TCTCTACATT
Dren58_Cb_SF   TACTGGGCAT CCTGATGTTT GCGTGGATTT TGTGATCTTT TCTCTACATT
Dren66_Cf_SF   TACTGGACAT CCTGATGTCT GCGTGGATTT TGTGATCTTT TCTCTACATT
Dren67_Cf_SF   TACTGGGCAT CCTGATGTTT GCGTGGATTT TGTGATCTTT TCTCTACATT
Dren68_Cf_SF   TACTGGGCAT CCTGATGTTT GCGTGGATTT TGTGATCTTT TCTCTACATT
Dren69_Cf_SF   TACTGGGCAT CCTGATGTTT GCGTGGATTT TGTGATCTTT TCTCTACATT
Dren70_Cf_SF   TACTGGGCAT CCTGATGTTT GCGTGGATTT TGTGATCTTT TCTCTACATT
Dren72_Cb_SF   TACTGGGCAT CCTGATGTTT GCGTGGATTT TGTGATCTTT TCTCTACATT
Dren73_Cb_Ch   TACTGGGCAT CCTGATGTTT GCGTGGATTT TGTGATCTTT TCTCTACATT
Dren74_Cb_Co   TACTGGTCAT CCTGATGTTT GCGTGGATTT TGTGATCTTT TCTCTACATT
Dren75_Cb_Co   TACTGGGCAT CCTGATGTTT GCGTGGATTT TGTGATCTTT TCTCTACATT
Dren80_Cf_SF   TACTGGGCAT CCTGATGTTT GCGTGGATTT TGTGATCTTT TCTCTACATT
Dren81_Cf_SF   TACTGGGCAT CCTGATGTTT GCGTGGATTT TGTGATCTTT TCTCTACATT
Dren82_Cb_Co   TACTGGGCAT CCTGATGTTT GCGTGGATTT TGTGATCTTT TCTCTACATT
Dren83_Cb_Ch   TACTGGGCAT CCTGATGTTT GCGTGGATTT TGTGATCTTT TCTCTACATT
Dren84_Cf_SF   TACTGGGCAT CCTGATGTTT GCGTGGATTT TGTGATCTTT TCTCTACATT
Dren85_Cb_SF   TACTGGGCAT CCTGATGTTT GCGTGGATTT TGTGATCTTT TCTCTACATT
Dren86_Cb_Co   TACTGGGCAT CCTGATGTTT GCGTGGATTT TGTGATCTTT TCTCTACATT
Dren87_Cf_BA   TACTGGGCAT CCTGATGTTT GCGTGGATTT TGTGATCTTT TCTCTACATT
Dren88_Lg_SF   TACTGGGCAT CCTGATGTTT GCGTGGATTT TGTGATCTTT TCTCTACATT

               ....|....| ....|....| ....|....| ....|....| ....|....|
                   405        415        425        435        445
Dren01_Cf_BA   TGGCGGGGGT GTCTTCTCTA TTAAGAAGTG TAAATTTTGT AACGACGTGT
Dren02_Cf_BA   TGGCGGGGGT GTCTTCTCTA TTAAGAAGTG TAAATTTTGT AACGACGTGT
Dren03_Cf_BA   TGGCGGGGGT GTCTTCTCTA TTAAGAAGTG TAAATTTTGT AACGACGTGT
Dren05_Cf_BA   TGGCGGGGGT GTCTTCTCTA TTAAGAAGTG TAAATTTTGT AACGACGTGT
Dren06_Cf_BA   TAGCGGGGGT GTCTTCTCTA TTAAGAAGTG TAAATTTTGT AACGACGTGT
Dren07_Cf_BA   TGGCGGGGGT GTCTTCTCTA TTAAGAAGTG TAAATTTTGT AACGACGTGT
Dren08_Cf_BA   TGGCGGGGGT GTCTTCTCTA TTAAGAAGTG TAAATTTTGT AACGACGTGT
Dren09_Cf_BA   TGGCGGGGGT GTCTTCTCTA TTAAGAAGTG TAAATTTTGT AACGACGTGT
Dren10_Cf_BA   TGGCGGGGGT GTCTTCTCTA TTAAGAAGTG TAAATTTTGT AACGACGTGT
Dren11_Cf_BA   TGGCGGGGGT GTCTTCTCTA TTAAGAAGTG TAAATTTTGT AACGACGTGT
Dren18_Cf_BA   TGGCGGGGGT GTCTTCTCTA TTAAGAAGTG TAAATTTTGT AACGACGTGT
Dren19_Cf_BA   TGGCGGGGGT GTCTTCTCTA TTAAGAAGTG TAAATTTTGT AACGACGTGT
Dren23_Cf_BA   TGGCGGGGGT GTCTTCTCTA TTAAGAAGTG TAAATTTTGT AACGACGTGT
Dren25_Cf_SF   TGGCGGGGGT GTCTTCTCTA TTAAGAAGTG TAAATTTTGT AACGACGTGT
Dren32_Cf_SC   TGGCGGGGGT GTCTTCTCTA TTAAGAAGTG TAAATTTTGT AACGACGTGT
Dren33_Cf_SC   TGGCGGGGGT GTCTTCTCTA TTAAGAAGTG TAAATTTTGT AACGACGTGT
Dren34_Cf_Pa   TGGCGGGGGT GTCTTCTCTA TTAAGAAGTG TAAATTTTGT AACGACGTGT
Dren35_Cf_Pa   TGGCGGGGGT GTCTTCTCTA TTAAGAAGTG TAAATTTTGT AACGACGTGT
```

```
Dren40_Cf_RG   TGGCGGGGGT GTCTTCTCTA TTAAGAAGTG TAAATTTTGT AACGACGTGT
Dren41_Cf_RG   TGGCGGGGGT GTCTTCTCTA TTAAGAAGTG TAAATTTTGT AACGACGTGT
Dren42_Cf_RG   TGGCGGGGGT GTCTTCTCTA TTAAGAAGTG TAAATTTTGT AACGACGTGT
Dren43_Cf_RG   TGGCGGGGGT GTCTTCTCTA TTAAGAAGTG TAAATTTTGT AACGACGTGT
Dren45_Cf_RG   TGGCGGGGGT GTCTTCTCTA TTAAGAAGTG TAAATTTTGT AACGACGTGT
Dren46_Cf_SF   TGGCGGGGGT GTCTTCTCTA TTAAGAAGTG TAAATTTTGT AACGACGTGT
Dren47_Cb_SF   TGGCGGGGGT GTCTTCTCTA TTAAGAAGTG TAAATTTTGT AACGACGTGT
Dren48_Cf_BA   TGGCGGGGGT GTCTTCTCTA TTAAGAAGTG TAAATTTTGT AACGACGTGT
Dren49_Cf_BA   TGGCGGGGGT GTCTTCTCTA TTAAGAAGTG TAAATTTTGT AACGACGTGT
Dren50_Cf_Ch   TGGCGGGGGT GTCTTCTCTA TTAAGAAGTG TAAATTTTGT AACGACGTGT
Dren53_Cf_BA   TGGCGGGGGT GTCTTCTCTA TTAAGAAGTG TAAATTTTGT AACGACGTGT
Dren58_Cb_SF   TGGCGGGGGT GTCTTCTCTA TTAAGAAGTG TAAATTTTGT AACGACGTGT
Dren66_Cf_SF   TGGCGGGGGT GTCTTCTCTA TTAAGAAGTG TAAATTTTGT AACGACGTGT
Dren67_Cf_SF   TGGCGGGGGT GTCTTCTCTA TTAAGAAGTG TAAATTTTGT AACGACGTGT
Dren68_Cf_SF   TGGCGGGGGT GTCTTCTCTA TTAAGAAGTG TAAATTTTGT AACGACGTGT
Dren69_Cf_SF   TGGCGGGGGT GTCTTCTCTA TTAAGAAGTG TAAATTTTGT AACGACGTGT
Dren70_Cf_SF   TGGCGGGGGT GTCTTCTCTA TTAAGAAGTG TAAATTTTGT AACGACGTGT
Dren72_Cb_SF   TGGCGGGGGT GTCTTCTCTA TTAAGAAGTG TAAATTTTGT AACGACGTGT
Dren73_Cb_Ch   TGGCGGGGGT GTCTTCTCTA TTAAGAAGTG TAAATTTTGT AACGACGTGT
Dren74_Cb_Co   TGGCGGGGGT GTCTTCTCTA TTAAGAAGTG TAAATTTTGT AACGACGTGT
Dren75_Cb_Co   TGGCGGGGGT GTCTTCTCTA TTAAGAAGTG TAAATTTTGT AACGACGTGT
Dren80_Cf_SF   TGGCGGGGGT GTCTTCTCTA TTAAGAAGTG TAAATTTTGT AACGACGTGT
Dren81_Cf_SF   TGGCGGGGGT GTCTTCTCTA TTAAGAAGTG TAAATTTTGT AACGACGTGT
Dren82_Cb_Co   TGGCGGGGGT GTCTTCTCTA TTAAGAAGTG TAAATTTTGT AACGACGTGT
Dren83_Cb_Ch   TGGCGGGGGT GTCTTCTCTA TTAAGAAGTG TAAATTTTGT AACGACGTGT
Dren84_Cf_SF   TGGCGGGGGT GTCTTCTCTA TTAAGAAGTG TAAATTTTGT AACGACGTGT
Dren85_Cb_SF   TGGCGGGGGT GTCTTCTCTA TTAAGAAGTG TAAATTTTGT AACGACGTGT
Dren86_Cb_Co   TGGCGGGGGT GTCTTCTCTA TTAAGAAGTG TAAATTTTGT AACGACGTGT
Dren87_Cf_BA   TGGCGGGGGT GTCTTCTCTA TTAAGAAGTG TAAATTTTGT AACGACGTGT
Dren88_Lg_SF   TGGCGGGGGT GTCTTCTCTA TTAAGAAGTG TAAATTTTGT AACGACGTGT

               ....|....| ....|....| ....|....| ....|....| ....|....|
                    455        465        475        485        495
Dren01_Cf_BA   TTTTCTGTTT GTGATTCGGT TTCTTCATTA GAAGATATGC CAATGTTTGC
Dren02_Cf_BA   TTTTCTGTTT GTGATTCGGT TTCTTCATTA GAAGATATGC CAATGTTTGC
Dren03_Cf_BA   TTTTCTGTTT GTGATTCGGT TTCTTCATTA GAAGATATGC CAATGTTTGC
Dren05_Cf_BA   TTTTCTGTTT GTGATTCGGT TTCTTCATTA GAAGATATGC CAATGTTTGC
Dren06_Cf_BA   TTTTCTGTTT GTGATTCGGT TTCTTCATTA GAAGATATGC CAATGTTTGC
Dren07_Cf_BA   TTTTCTGTTT GTGATTCGGT TTCTTCATTA GAAGATATGC CAATGTTTGC
Dren08_Cf_BA   TTTTCTGTTT GTGATTCGGT TTCTTCATTA GAAGATATGC CAATGTTTGC
Dren09_Cf_BA   TTTTCTGTTT GTGGTTCGGT TTCTTCATTA GAAGATATGC CAATGTTTGC
Dren10_Cf_BA   TTTTCTGTTT GTGATTCGGT TTCTTCATTA GAAGATATGC CAATGTTTGC
Dren11_Cf_BA   TTTTCTGTTT GTGATTCGGT TTCTTCATTA GAAGATATGC CAATGTTTGC
Dren18_Cf_BA   TTTTCTGTTT GTGATTCGGT TTCTTCATTA GAAGATATGC CAATGTTTGC
Dren19_Cf_BA   TTTTCTGTTT GTGATTCGGT TTCTTCATTA GAAGATATGC CAATGTTTGC
Dren23_Cf_BA   TTTTCTGTTT GTGATTCGGT TTCTTCATTA GAAGATATGC CAATGTTTGC
Dren25_Cf_SF   TTTTCTGTTT GTGATTCGGT TTCTTCATTA GAAGATATGC CAATGTTTGC
Dren32_Cf_SC   TTTTCTGTTT GTGATTCGGT TTCTTCATTA GAAGATATGC CAATGTTTGC
Dren33_Cf_SC   TTTTCTGTTT GTGATTCGGT TTCTTCATTA GAAGATATGC CAATGTTTGC
Dren34_Cf_Pa   TTTTCTGTTT GTGATTCGGT TTCTTCATTA GAAGATATGC CAATGTTTGC
Dren35_Cf_Pa   TTTTCTGTTT GTGATTCGGT TTCTTCATTA GAAGATATGC CAATGTTTGC
Dren40_Cf_RG   TTTTCTGTTT GTGATTCGGT TTCTTCATTA GAAGATATGC CAATGTTTGC
Dren41_Cf_RG   TTTTCTGTTT GTGATTCGGT TTCTTCATTA GAAGATATGC CAATGTTTGC
Dren42_Cf_RG   TTTTCTGTTT GTGATTCGGT TTCTTCATTA GAAGATATGC CAATGTTTGC
```

```
Dren43_Cf_RG   TTTTCTGTTT GTGATTCGGT TTCTTCATTA GAAGATATGC CAATGTTTGC
Dren45_Cf_RG   TTTTCTGTTT GTGATTCGGT TTCTTCATTA GAAGATATGC CAATGTTTGC
Dren46_Cf_SF   TTTTCTGTTT GTGATTCGGT TTCTTCATTA GAAGATATGC CAATGTTTGC
Dren47_Cb_SF   TTTTCTGTTT GTGATTCGGT TTCTTCATTA GAAGATATGC CAATGTTTGC
Dren48_Cf_BA   TTTTCTGTTT GTGATTCGGT TTCTTCATTA GAAGATATGC CAATGTTTGC
Dren49_Cf_BA   TTTTCTGTTT GTGATTCGGT TTCTTCATTA GAAGATATGC CAATGTTTGC
Dren50_Cf_Ch   TTTTCTGTTT GTGATTCGGT TTCTTCATTA GAAGATATGC CAATGTTTGC
Dren53_Cf_BA   TTTTCTGTTT GTGATTCGGT TTCTTCATTA GAAGATATGC CAATGTTTGC
Dren58_Cb_SF   TTTTCTGTTT GTGATTCGGT TTCTTCATTA GAAGATATGC CAATGTTTGC
Dren66_Cf_SF   TTTTCTGTTT GTGATTCGGT TTCTTCATTA GAAGATATGC CAATGTTTGC
Dren67_Cf_SF   TTTTCTGTTT GTGATTCGGT TTCTTCATTA GAAGATATGC CAATGTTTGC
Dren68_Cf_SF   TTTTCTGTTT GTGATTCGGT TTCTTCATTA GAAGATATGC CAATGTTTGC
Dren69_Cf_SF   TTTTCTGTTT GTGATTCGGT TTCTTCATTA GAAGATATGC CAATGTTTGC
Dren70_Cf_SF   TTTTCTGTTT GTGATTCGGT TTCTTCATTA GAAGATATGC CAATGTTTGC
Dren72_Cb_SF   TTTTCTGTTT GTGATTCGGT TTCTTCATTA GAAGATATGC CAATGTTTGC
Dren73_Cb_Ch   TTTTCTGTTT GTGATTCGGT TTCTTCATTA GAAGATATGC CAATGTTTGC
Dren74_Cb_Co   TTTTCTGTTT GTGATTCGGT TTCTTCATTA GAAGATATGC CAATGTTTGC
Dren75_Cb_Co   TTTTCTGTTT GTGATTCGGT TTCTTCATTA GAAGATATGC CAATGTTTGC
Dren80_Cf_SF   TTTTCTGTTT GTGATTCGGT TTCTTCATTA GAAGATATGC CAATGTTTGC
Dren81_Cf_SF   TTTTCTGTTT GTGATTCGGT TTCTTCATTA GAAGATATGC CAATGTTTGC
Dren82_Cb_Co   TTTTCTGTTT GTGATTCGGT TTCTTCATTA GAAGATATGC CAATGTTTGC
Dren83_Cb_Ch   TTTTCTGTTT GTGATTCGGT TTCTTCATTA GAAGATATGC CAATGTTTGC
Dren84_Cf_SF   TTTTCTGTTT GTGATTCGGT TTCTTCATTA GAAGATATGC CAATGTTTGC
Dren85_Cb_SF   TTTTCTGTTT GTGATTCGGT TTCTTCATTA GAAGATATGC CAATGTTTGC
Dren86_Cb_Co   TTTTCTGTTT GTGATTCGGT TTCTTCATTA GAAGATATGC CAATGTTTGC
Dren87_Cf_BA   TTTTCTGTTT GTGATTCGGT TTCTTCATTA GAAGATATGC CAATGTTTGC
Dren88_Lg_SF   TTTTCTGTTT GTGATTCGGT TTCTTCATTA GAAGATATGC CAATGTTTGC

               ....|....| ....|....| ....|....| ....|....| ....|....|
                   505        515        525        535        545
Dren01_Cf_BA   GTGAAGCATT GTGGTGACGT CCTTCATACT AATTGTTTCT CTTCCTGTTT
Dren02_Cf_BA   GTGAAGCATT GTGGTGACGT CCTTCATACT AATTGTTTCT CTTCCTGTTT
Dren03_Cf_BA   GTGAAGCATT GTGGTGACGT CCTTCATACT AATTGTTTCT CTTCCTGTTT
Dren05_Cf_BA   GTGAAGCATT GTGGTGACGT CCTTCATACT AATTGTTTCT CTTCCTGTTT
Dren06_Cf_BA   GTGAAGCATT GTGGTGACGT CCTTCATACT AATTGTTTCT CTTCCTGTTT
Dren07_Cf_BA   GTGAAGCATT GTGGTGACGT CCTTCATACT AATTGTTTCT CTTCCTGTTT
Dren08_Cf_BA   GTGAAGCATT GTGGTGACGT CCTTCATACT AATTGTTTCT CTTCCTGTTT
Dren09_Cf_BA   GTGAAGCATT GTGGTGACGT CCTTCATACT AATTGTTTCT CTTCCTGTTT
Dren10_Cf_BA   GTGAAGCATT GTGGTGACGT CCTTCATACT AATTGTTTCT CTTCCTGTTT
Dren11_Cf_BA   GTGAAGCATT GTGGTGACGT CCTTCATACT AATTGTTTCT CTTCCTGTTT
Dren18_Cf_BA   GTGAAGCATT GTGGTGACGT CCTTCATACT AATTGTTTCT CTTCCTGTTT
Dren19_Cf_BA   GTGAAGCATT GTGGTGACGT CCTTCATACT AATTGTTTCT CTTCCTGTTT
Dren23_Cf_BA   GTGAAGCATT GTGGTGACGT CCTTCATACT AATTGTCTCT CTTCCTGTTT
Dren25_Cf_SF   GTGAAGCATT GTGGTGACGT CCTTCATACT AATTGTTTCT CTTCCTGTTT
Dren32_Cf_SC   GTGAAGCATT GTGGTGACGT CCTTCATACT AATTGTTTCT CTTCCTGTTT
Dren33_Cf_SC   GTGAAGCATT GTGGTGACGT CCTTCATACT AATTGTTTCT CTTCCTGTTT
Dren34_Cf_Pa   GTGAAGCATT GTGGTGACGT CCTTCATACT AATTGTTTCT CTTCCTGTTT
Dren35_Cf_Pa   GTGAAGCATT GTGGTGACGT CCTTCATACT AATTGTTTCT CTTCCTGTTT
Dren40_Cf_RG   GTGAAGCATT GTGGTGACGT CCTTCATACT AATTGTTTCT CTTCCTGTTT
Dren41_Cf_RG   GTGAAGCATT GTGGTGACGT CCTTCATACT AATTGTTTCT CTTCCTGTTT
Dren42_Cf_RG   GTGAAGCATT GTGGTGACGT CCTTCATACT AATTGTTTCT CTTCCTGTTT
Dren43_Cf_RG   GTGAAGCATT GTGGTGACGT CCTTCATACT AATTGTTTCT CTTCCTGTTT
Dren45_Cf_RG   GTGAAGCATT GTGGTGACGT CCTTCATACT AATTGTTTCT CTTCCTGTTT
Dren46_Cf_SF   GTGAAGCATT GTGGTGACGT CCTTCATACT AATTGTTTCT CTTCCTGTTT
```

```
Dren47_Cb_SF   GTGAAGCATT GTGGTGACGT CCTTCATACT AATTGTTTCT CTTCCTGTTT
Dren48_Cf_BA   GTGAAGCATT GTGGTGACGT CCTTCATACT AATTGTTTCT CTTCCTGTTT
Dren49_Cf_BA   GTGAAGCATT GTGGTGACGT CCTTCATACT AATTGTTTCT CTTCCTGTTT
Dren50_Cf_Ch   GTGAAGCATT GTGGTGACGT CCTTCATACT AATTGTTTCT CTTCCTGTTT
Dren53_Cf_BA   GTGAAGCATT GTGGTGACGT CCTTCATACT AATTGTTTCT CTTCCTGTTT
Dren58_Cb_SF   GTGAAGCATT GTGGTGACGT CCTTCATACT AATTGTTTCT CTTCCTGTTT
Dren66_Cf_SF   GTGAAGCATT GTGGTGACGT CCTTCATACT AATTGTTTCT CTTCCTGTTT
Dren67_Cf_SF   GTGAAGCATT GTGGTGACGT CCTTCATACT AATTGTTTCT CTTCCTGTTT
Dren68_Cf_SF   GTGAAGCATT GTGGTGACGT CCTTCATACT AATTGTTTCT CTTCCTGTTT
Dren69_Cf_SF   GTGAAGCATT GTGGTGACGT CCTTCATACT AATTGTTTCT CTTCCTGTTT
Dren70_Cf_SF   GTGAAGCATT GTGGTGACGT CCTTCATACT AATTGTTTCT CTTCCTGTTT
Dren72_Cb_SF   GTGAAGCATT GTGGTGACGT CCTTCATACT AATTGTTTCT CTTCCTGTTT
Dren73_Cb_Ch   GTGAAGCATT GTGGTGACGT CCTTCATACT AATTGTTTCT CTTCCTGTTT
Dren74_Cb_Co   GTGAAGCATT GTGGTGACGT CCTTCATACT AATTGTTTCT CTTCCTGTTT
Dren75_Cb_Co   GTGAAGCATT GTGGTGACGT CCTTCATACT AATTGTTTCT CTTCCTGTTT
Dren80_Cf_SF   GTGAAGCATT GTGGTGACGT CCTTCATACT AATTGTTTCT CTTCCTGTTT
Dren81_Cf_SF   GTGAAGCATT GTGGTGACGT CCTTCATACT AATTGTTTCT CTTCCTGTTT
Dren82_Cb_Co   GTGAAGCATT GTGGTGACGT CCTTCATACT AATTGTTTCT CTTCCTGTTT
Dren83_Cb_Ch   GTGAAGCATT GTGGTGACGT CCTTCATACT AATTGTTTCT CTTCCTGTTT
Dren84_Cf_SF   GTGAAGCATT GTGGTGACGT CCTTCATACT AATTGTTTCT CTTCCTGTTT
Dren85_Cb_SF   GTGAAGCATT GTGGTGACGT CCTTCATACT AATTGTTTCT CTTCCTGTTT
Dren86_Cb_Co   GTGAAGCATT GTGGTGACGT CCTTCATACT AATTGTTTCT CTTCCTGTTT
Dren87_Cf_BA   GTGAAGCATT GTGGTAACGT CCTTCATACT AATTGTTTCT CTTCCTGTTT
Dren88_Lg_SF   GTGAAGCATT GTGGTGACGT CCTTCATACT AATTGTTTCT CTTCCTGTTT

               ....|....| ....|....| ....|....| ....|....| ....|....|
                     555        565        575        585        595
Dren01_Cf_BA   TAGCAGGGGG TATTACAATA CTTTTAACTG ATCGGAATTT TAACACAGCT
Dren02_Cf_BA   TGGCAGGGGG TATCACAATA CTTTTAACTG ATCGGA---- ----------
Dren03_Cf_BA   TAGCAGGGGG TATTACAATA CTTTTAACTG ATCGGA---- ----------
Dren05_Cf_BA   TAGCAGGGGG TATTACAATA CTTTTAACTG ATCGGA---- ----------
Dren06_Cf_BA   TAGCAGGGGG TATTACAATA CTTTTAACTG ATCGGA---- ----------
Dren07_Cf_BA   TAGCAGGGGG TATTACAATA CTTTTAACTG ATCGGA---- ----------
Dren08_Cf_BA   TAGCAGGGGG TATTACAATA CTTTTAACTG ATCGGA---- ----------
Dren09_Cf_BA   TGGCAGGGGG TATCACAATA CTTTTAACTG ATCGGA---- ----------
Dren10_Cf_BA   TAGCAGGGGG TATTACAATA CTTTTAACTG ATCGGA---- ----------
Dren11_Cf_BA   TGGCAGGGGG TATCACAATA CTTTTAACTG ATCGGA---- ----------
Dren18_Cf_BA   TAGCAGGGGG TATTACAATA CTTTTAACTG ATCGGA---- ----------
Dren19_Cf_BA   TAGCAGGGGG TATTACAATA CTTTTAACTG ATCGGA---- ----------
Dren23_Cf_BA   TGGCAGGGGG TATCACAATA CTTTTAACTG ATCGGA---- ----------
Dren25_Cf_SF   TGGCAGGGGG TATCACAATA CTTTTAACTG ATCGGA---- ----------
Dren32_Cf_SC   TGGCAGGGGG TATCACAATA CTTTTAACTG ATCGGA---- ----------
Dren33_Cf_SC   TGGCAGGGGG TATCACAATA CTTTTAACTG ATCGGA---- ----------
Dren34_Cf_Pa   TGGCAGGGGG TATCACAATA CTTTTAACTG ATCGGA---- ----------
Dren35_Cf_Pa   TGGCAGGGGG TATCACAATA CTTTTAACTG ATCGGA---- ----------
Dren40_Cf_RG   TAGCAGGGGG TATTACAATA CTTTTAACTG ATCGGAATTT TAACACAGCT
Dren41_Cf_RG   TAGCAGGGGG TATTACAATA CTTTTAACTG ATCGGA---- ----------
Dren42_Cf_RG   TAGCAGGGGG TATTACAATA CTTTTAACTG ATCGGA---- ----------
Dren43_Cf_RG   TAGCAGGGGG TATCACAATA CTTTTAACTG ATCGGA---- ----------
Dren45_Cf_RG   TAGCAGGGGG TATCACAATA CTTTTAACTG ATCGGA---- ----------
Dren46_Cf_SF   TGGCAGGGGG TATCACAATA CTTTTAACTG ATCGGAATTT TAACACAGCT
Dren47_Cb_SF   TAGCAGGGGG TATTACAATA CTTTTAACTG ATCGGA---- ----------
Dren48_Cf_BA   TAGCAGGGGG TATTACAATA CTTTTAACTG ATCGGAATTT TAACACANCT
Dren49_Cf_BA   TGGCAGGGGG TATCACAATA CTTTTAACTG ATCGGA---- ----------
```

```
Dren50_Cf_Ch    TAGCAGGGGG TATTACAATA CTTTTAACTG ATCGGAATTT TAACACAGCT
Dren53_Cf_BA    TAGCAGGGGG TATTACAATA CTTTTAACTG ATCGGA---- ----------
Dren58_Cb_SF    TAGCAGGGGG TATTACAATA CTTTTAACTG ATCGGA---- ----------
Dren66_Cf_SF    TAGCAGGGGG TATTACAATA CTTTTAACTG ATCGGAATTT TAACACAGCT
Dren67_Cf_SF    TAGCAGGGGG TATTACAATA CTTTTAACTG ATCGGA---- ----------
Dren68_Cf_SF    TAGCAGGGGG TATTACAATA CTTTTAACTG ATCGGAATTT TAACACAGCT
Dren69_Cf_SF    TAGCAGGGGG TATTACAATA CTTTTAACTG ATCGGA---- ----------
Dren70_Cf_SF    TGGCAGGGGG TATCACAATA CTTTTAACTG ATCGGAATTT TAACACAGCT
Dren72_Cb_SF    TAGCAGGGGG TATTACAATA CTTTTAACTG ATCGGAATTT TAACACAGCT
Dren73_Cb_Ch    TAGCAGGGGG TATTACAATA CTTTTAACTG ATCGGAATTT TAACACAGCT
Dren74_Cb_Co    TGGCAGGGGG TATCACAATA CTTTTAACTG ATCGGAATTT TAACACAGCT
Dren75_Cb_Co    TAGCAGGGGG TATTACAATA CTTTTAACTG ATCGGAATTT TAACACAGCT
Dren80_Cf_SF    TGGCAGGGGG TATCACAATA CTTTTAACTG ATCGGAATTT TAACACAGCT
Dren81_Cf_SF    TGGCAGGGGG TATCACAATA CTTTTAACTG ATCGGAATTT TAACACAGCT
Dren82_Cb_Co    TGGCAGGGGG TATCACAATA CTTTTAACTG ATCGGAATTT TAACACAGCT
Dren83_Cb_Ch    TAGCAGGGGG TATTACAATA CTTTTAACTG ATCGGAATTT TAACACAGCT
Dren84_Cf_SF    TGGCAGGGGG TATCACAATA CTTTTAACTG ATCGGAATTT TAACACAGCT
Dren85_Cb_SF    TGGCAGGGGG TATCACAATA CTTTTAACTG ATCGGAATTT TAACACAGCT
Dren86_Cb_Co    TAGCAGGGGG TATTACAATA CTTTTAACTG ATCGGAATTT TAACACAGCT
Dren87_Cf_BA    TAGCAGGGGG TATTACAATA CTTTTAACTG ATCGGAATTT TAACACAGCT
Dren88_Lg_SF    TAGCAGGGGG TATTACAATA CTTTTAACTG ATCGGAATTT TAACACAGCT

                ....|....| ....|....| ....|....| ....|....| ....|....|
                    605        615        625        635        645
Dren01_Cf_BA    TTTTTTGAAA ---------- ---------- ---------- ----------
Dren02_Cf_BA    ---------- ---------- ---------- ---------- ----------
Dren03_Cf_BA    ---------- ---------- ---------- ---------- ----------
Dren05_Cf_BA    ---------- ---------- ---------- ---------- ----------
Dren06_Cf_BA    ---------- ---------- ---------- ---------- ----------
Dren07_Cf_BA    ---------- ---------- ---------- ---------- ----------
Dren08_Cf_BA    ---------- ---------- ---------- ---------- ----------
Dren09_Cf_BA    ---------- ---------- ---------- ---------- ----------
Dren10_Cf_BA    ---------- ---------- ---------- ---------- ----------
Dren11_Cf_BA    ---------- ---------- ---------- ---------- ----------
Dren18_Cf_BA    ---------- ---------- ---------- ---------- ----------
Dren19_Cf_BA    ---------- ---------- ---------- ---------- ----------
Dren23_Cf_BA    ---------- ---------- ---------- ---------- ----------
Dren25_Cf_SF    ---------- ---------- ---------- ---------- ----------
Dren32_Cf_SC    ---------- ---------- ---------- ---------- ----------
Dren33_Cf_SC    ---------- ---------- ---------- ---------- ----------
Dren34_Cf_Pa    ---------- ---------- ---------- ---------- ----------
Dren35_Cf_Pa    ---------- ---------- ---------- ---------- ----------
Dren40_Cf_RG    TTTTTTGAAA CTTCGGGAGG AGGTGACCCT GTTCTATTCC AACATATTTT
Dren41_Cf_RG    ---------- ---------- ---------- ---------- ----------
Dren42_Cf_RG    ---------- ---------- ---------- ---------- ----------
Dren43_Cf_RG    ---------- ---------- ---------- ---------- ----------
Dren45_Cf_RG    ---------- ---------- ---------- ---------- ----------
Dren46_Cf_SF    TTTTTTGAAA CTTCGGGAGG AGGTGACCCT GTTCTATTCC AACATATTTT
Dren47_Cb_SF    ---------- ---------- ---------- ---------- ----------
Dren48_Cf_BA    TTTTTTGAAA CTTCGGGAGG AGG------- ---------- ----------
Dren49_Cf_BA    ---------- ---------- ---------- ---------- ----------
Dren50_Cf_Ch    TTTTTTGAAA CTTCGGGAGG AGGTGACCCT GTTCTATTCC AACATATTTT
Dren53_Cf_BA    ---------- ---------- ---------- ---------- ----------
Dren58_Cb_SF    ---------- ---------- ---------- ---------- ----------
```

```
Dren66_Cf_SF   TTTTTTGAAA CTTCGGGAGG AGGTGACCCT GTTCTATTCC AACATATTTT
Dren67_Cf_SF   ---------- ---------- ---------- ---------- ----------
Dren68_Cf_SF   TTTTTTGAAA CTTCGGGAGG AGGTGACCCT GTTCTATTCC AACATATTTT
Dren69_Cf_SF   ---------- ---------- ---------- ---------- ----------
Dren70_Cf_SF   TTTTTTGAAA CTTCGGGAGG AGGTGACCCT GTTCTATTCC AACATATTTT
Dren72_Cb_SF   TTTTTTGAAA CTTCGGGAGG AGGTGACCCT GTTCTATTCC AACATATTTT
Dren73_Cb_Ch   TTTTTTGAAA CTTCGGGAGG AGGTGACCCT GTTCTATTCC AACATATTTT
Dren74_Cb_Co   TTTTTTGAAA CTTCGGGAGG AGGTGACCCT GTTCTATTCC AACATATTTT
Dren75_Cb_Co   TTTTTTGAAA CTTCGGGAGG AGGTGACCCT GTTCTATTCC AACATATTTT
Dren80_Cf_SF   TTTTTTGAAA CTTCGGGAGG AGGTGACCCT GTTCTATTCC AACATATTTT
Dren81_Cf_SF   TTTTTTGAAA CTTCGGGAGG AGGTGACCCT GTTCTATTCC AACATATTTT
Dren82_Cb_Co   TTTTTTGAAA CTTCGGGAGG AGGTGACCCT GTTCTATTCC AACATATTTT
Dren83_Cb_Ch   TTTTTTGAAA CTTCGGGAGG AGGTGACCCT GTTCTATTCC AACATATTTT
Dren84_Cf_SF   TTTTTTGAAA CTTCGGGAGG AGGTGACCCT GTTCTATTCC AACATATTTT
Dren85_Cb_SF   TTTTTTGAAA CTTCGGGAGG AGGTGACCCT GTTCTATTCC AACATATTTT
Dren86_Cb_Co   TTTTTTGAAA CTTCGGGAGG AGGTGACCCT GTTCTATTCC AACATATTTT
Dren87_Cf_BA   TTTTTTGAAA CTTCGGGAGG AGGTGACCCT GTTCTATTCC AACATATTTT
Dren88_Lg_SF   TTTTTTGAAA CTTCGGGAGG AGGTGACCCT GTTCTATTCC AACATATTTT

               ....|....| ....|....| ....|....| ....|....| ....|....|
                     655        665        675        685        695
Dren01_Cf_BA   ---------- ---------- ---------- ---------- ----------
Dren02_Cf_BA   ---------- ---------- ---------- ---------- ----------
Dren03_Cf_BA   ---------- ---------- ---------- ---------- ----------
Dren05_Cf_BA   ---------- ---------- ---------- ---------- ----------
Dren06_Cf_BA   ---------- ---------- ---------- ---------- ----------
Dren07_Cf_BA   ---------- ---------- ---------- ---------- ----------
Dren08_Cf_BA   ---------- ---------- ---------- ---------- ----------
Dren09_Cf_BA   ---------- ---------- ---------- ---------- ----------
Dren10_Cf_BA   ---------- ---------- ---------- ---------- ----------
Dren11_Cf_BA   ---------- ---------- ---------- ---------- ----------
Dren18_Cf_BA   ---------- ---------- ---------- ---------- ----------
Dren19_Cf_BA   ---------- ---------- ---------- ---------- ----------
Dren23_Cf_BA   ---------- ---------- ---------- ---------- ----------
Dren25_Cf_SF   ---------- ---------- ---------- ---------- ----------
Dren32_Cf_SC   ---------- ---------- ---------- ---------- ----------
Dren33_Cf_SC   ---------- ---------- ---------- ---------- ----------
Dren34_Cf_Pa   ---------- ---------- ---------- ---------- ----------
Dren35_Cf_Pa   ---------- ---------- ---------- ---------- ----------
Dren40_Cf_RG   CTGATTTTTT GGCCATCCAG AGGTTTATAT TTTAGCTTTG CCTGCTTTTG
Dren41_Cf_RG   ---------- ---------- ---------- ---------- ----------
Dren42_Cf_RG   ---------- ---------- ---------- ---------- ----------
Dren43_Cf_RG   ---------- ---------- ---------- ---------- ----------
Dren45_Cf_RG   ---------- ---------- ---------- ---------- ----------
Dren46_Cf_SF   CTGATTTTTT GGCCATCCAG AGGTTTATAT TTTAGCTTTG CCTGCTTTTG
Dren47_Cb_SF   ---------- ---------- ---------- ---------- ----------
Dren48_Cf_BA   ---------- ---------- ---------- ---------- ----------
Dren49_Cf_BA   ---------- ---------- ---------- ---------- ----------
Dren50_Cf_Ch   CTGATTTTTT GGCCATCCAG AGGTTTATAT TTTAGCTTTG CCTGCTTTTG
Dren53_Cf_BA   ---------- ---------- ---------- ---------- ----------
Dren58_Cb_SF   ---------- ---------- ---------- ---------- ----------
Dren66_Cf_SF   CTGATTTTTT GGTCACCCAG AGGTTTATAT TTTAGCTTTG CCTGCTTTTG
Dren67_Cf_SF   ---------- ---------- ---------- ---------- ----------
Dren68_Cf_SF   CTGATTTTTT GGCCATCCAG AGGTTTATAT TTTAGCTTTG CCTGCTTTTG
```

```
Dren69_Cf_SF   ---------- ---------- ---------- ---------- ----------
Dren70_Cf_SF   CTGATTTTTT GGCCATCCAG AGGTTTATAT TTTAGCTTTG CCTGCTTTTG
Dren72_Cb_SF   CTGATTTTTT GGCCATCCAG AGGTTTATAT TTTAGCTTTG CCTGCTTTTG
Dren73_Cb_Ch   CTGATTTTTT GGCCATCCAG AGGTTTATAT TTTAGCTTTG CCTGCTTTTG
Dren74_Cb_Co   CTGATTTTTT GGCCATCCAG AGGTTTATAT TTTAGCTTTG CCTGCTTTTG
Dren75_Cb_Co   CTGATTTTTT GGCCATCCAG AGGTTTATAT TTTAGCTTTG CCTGCTTTTG
Dren80_Cf_SF   CTGATTTTTT GGCCATCCAG AGGTTTATAT TTTAGCTTTG CCTGCTTTTG
Dren81_Cf_SF   CTGATTTTTT GGCCATCCAG AGGTTTATAT TTTAGCTTTG CCTGCTTTTG
Dren82_Cb_Co   CTGATTTTTT GGCCATCCAG AGGTTTATAT TTTAGCTTTG CCTGCTTTTG
Dren83_Cb_Ch   CTGATTTTTT GGCCATCCAG AGGTTTATAT TTTAGCTTTG CCTGCTTTTG
Dren84_Cf_SF   CTGATTTTTT GGCCATCCAG AGGTTTATAT TTTAGCTTTG CCTGCTTTTG
Dren85_Cb_SF   CTGATTTTTT GGCCATCCAG AGGTTTATAT TTTAGCTTTG CCTGCTTTTG
Dren86_Cb_Co   CTGATTTTTT GGCCATCCAG AGGTTTATAT TTTAGCTTTG CCTGCTTTTG
Dren87_Cf_BA   CTGATTTTTT GGCCATCCAG AGGTTTATAT TTTAGCTTTG CCTGCTTTTG
Dren88_Lg_SF   CTGATTTTTT GGCCATCCAG AGGTTTATAT TTTAGCTTTG CCTGCTTTTG

               ....|....| ....|....| ....|....| ....|....| ....|....|
                  705        715        725        735        745
Dren01_Cf_BA   ---------- ---------- --------TA GAGGCAAGCC AAAGGTGTTT
Dren02_Cf_BA   ---------- ---------- --------TA GTGGCAAGCC AAAGGTGTTT
Dren03_Cf_BA   ---------- ---------- --------TA GAGGCAAGCC AAAGGTGTTT
Dren05_Cf_BA   ---------- ---------- --------TA GTGGCAAGCC AAAGGTGTTT
Dren06_Cf_BA   ---------- ---------- --------TA GAGGCAAGCC AAAGGTGTTT
Dren07_Cf_BA   ---------- ---------- --------TA GTGGCAAGCC AAAGGTGTTT
Dren08_Cf_BA   ---------- ---------- --------TA GAGGCAAGCC AAAGGTGTTT
Dren09_Cf_BA   ---------- ---------- --------TA GTGGCAAGCC AAAGGTGTTT
Dren10_Cf_BA   ---------- ---------- --------TA GAGGCAAGCC AAAGGTGTTT
Dren11_Cf_BA   ---------- ---------- --------TA GTGGCAAGCC AAAGGTGTTT
Dren18_Cf_BA   ---------- ---------- --------TA GTGGTAAGCC AAAGGTGTTT
Dren19_Cf_BA   ---------- ---------- --------TA GTGGTAAGCC AAAGGTGTTT
Dren23_Cf_BA   ---------- ---------- --------TA GTGGCAAGCC AAAGGTGTTT
Dren25_Cf_SF   ---------- ---------- --------TA GTGGCAAGCC AAAGGTGTTT
Dren32_Cf_SC   ---------- ---------- --------TA GTGGCAAGCC AAAGGTGTTT
Dren33_Cf_SC   ---------- ---------- --------TA GTGGCAAGCC AAAGGTGTTT
Dren34_Cf_Pa   ---------- ---------- --------TA GTGGCAAGCC AAAGGTGTTT
Dren35_Cf_Pa   ---------- ---------- --------TA GTGGCAAGCC AAAGGTGTTT
Dren40_Cf_RG   GTGTAATTTC AGAAAGAGTA ACAGTTCTTA GTGGCAAGCC AAAGGTGTTT
Dren41_Cf_RG   ---------- ---------- --------TA GTGGCAAGCC AAAGGTGTTT
Dren42_Cf_RG   ---------- ---------- --------TA GTGGCAAGCC AAAGGTGTTT
Dren43_Cf_RG   ---------- ---------- --------TA GCGGTAAGCC AAAGGTGTTT
Dren45_Cf_RG   ---------- ---------- --------TA GCGGTAAGCC AAAGGTGTTT
Dren46_Cf_SF   GTGTAATTTC AGAAAGGGTA ACAGTTCTTA GTGGCAAGCC AAAGGTGTTT
Dren47_Cb_SF   ---------- ---------- --------TA GTGGCAAGCC AAAGGTGTTT
Dren48_Cf_BA   ---------- ---------- --------TA GAGGCAAGCC AAAGGTGTTT
Dren49_Cf_BA   ---------- ---------- --------TA GTGGCAAGCC AAAGGTGTTT
Dren50_Cf_Ch   GTGTAATTTC AGAAAGAGTA ACAGTTCTTA GAGGCAAGCC AAAGGTGTTT
Dren53_Cf_BA   ---------- ---------- --------TA GAGGCAAGCC AAAGGTGTTT
Dren58_Cb_SF   ---------- ---------- --------TA GTGGCAAGCC AAAGGTGTTT
Dren66_Cf_SF   GTGTAATTTC AGAAAGAGTA ACAGTTCTTA GTGGTAAGCC AAAGGTGTTT
Dren67_Cf_SF   ---------- ---------- --------TA GTGGCAAGCC AAAGGTGTTT
Dren68_Cf_SF   GTGTAATTTC AGAAAGAGTA ACAGTTCTTA GTGGCAAGCC AAAGGTGTTT
Dren69_Cf_SF   ---------- ---------- --------TA GTGGCAAGCC AAAGGTGTTT
Dren70_Cf_SF   GTGTAATTTC AGAAAGAGTA ACAGTTCTTA GTGGCAAGCC AAAGGTGTTT
Dren72_Cb_SF   GTGTAATTTC AGAAAGAGTA ACAGTTCTTA GAGGCAAGCC AAAGGTGTTT
```

```
Dren73_Cb_Ch   GTGTAATTTC AGAAAGAGTA ACAGTTCTTA GTGGCAAGCC AAAGGTGTTT
Dren74_Cb_Co   GTGTAATTTC AGAAAGGGTA ACAGTTCTTA GTGGCAAGCC AAAGGTGTTT
Dren75_Cb_Co   GTGTAATTTC AGAAAGAGTA ACAGTTCTTA GAGGCAAGCC AAAGGTGTTT
Dren80_Cf_SF   GTGTAATTTC AGAAAGGGTA ACAGTTCTTA GTGGCAAGCC AAAGGTGTTT
Dren81_Cf_SF   GTGTAATTTC AGAAAGGGTA ACAGTTCTTA GTGGCAAGCC AAAGGTGTTT
Dren82_Cb_Co   GTGTAATTTC AGAAAGGGTA ACAGTTCTTA GTGGCAAGCC AAAGGTGTTT
Dren83_Cb_Ch   GTGTAATTTC AGAAAGAGTA ACAGTTCTTA GAGGCAAGCC AAAGGTGTTT
Dren84_Cf_SF   GTGTAATTTC AGAAAGGGTA ACAGTTCTTA GTGGCAAGCC AAAGGTGTTT
Dren85_Cb_SF   GTGTAATTTC AGAAAGAGTA ACAGTTCTTA GTGGCAAGCC AAAGGTGTTT
Dren86_Cb_Co   GCGTAATTTC AGAAAGAGTA ACAGTTCTTA GTGGCAAGCC AAAGGTGTTT
Dren87_Cf_BA   GTGTAATTTC AGAAAGAGTA ACAGTTCTTA GTGGCAAGCC AAAGGTGTTT
Dren88_Lg_SF   GTGTAATTTC AGAAAGAGTA ACAGTTCTTA GTGGCAAGCC AAAGGTGTTT

               ....|....| ....|....| ....|....| ....|....| ....|....|
                    755        765        775        785        795
Dren01_Cf_BA   GGACCTTTGG GTATAATCTA CGCTATAATT AGAATTGGCG TGCTTGGTTG
Dren02_Cf_BA   GGACCTTTGG GTATAATTTA CGCTATAATT AGAATTGGCG TGCTTGGTTG
Dren03_Cf_BA   GGACCTTTGG GTATAATCTA CGCTATAATT AGAATTGGCG TGCTTGGTTG
Dren05_Cf_BA   GGGCCTTTGG GCATAATCTA CGCTATAATT AGAATTGGCG TGCTTGGTTG
Dren06_Cf_BA   GGACCTTTGG GTATAATCTA CGCTATAATT AGAATTGGCG TGCTTGGTTG
Dren07_Cf_BA   GGGCCTTTGG GTATAATCTA CGCTATAATT AGAATTGGCG TGCTTGGTTG
Dren08_Cf_BA   GGACCTTTGG GTATAATCTA CGCTATAATT AGAATTGGCG TGCTTGGTTG
Dren09_Cf_BA   GGACCTTTGG GTATAATCTA CGCTATAATT AGAATTGGCG TGCTTGGTTG
Dren10_Cf_BA   GGACCTTTGG GTATAATCTA CGCTATAATT AGAATTGGCG TGCTTGGTTG
Dren11_Cf_BA   GGACCTTTGG GTATAATCTA CGCTATAATT AGAATTGGCG TGCTTGGTTG
Dren18_Cf_BA   GGACCTTTGG GCATAATCTA CGCTATAATT AGAATTGGCG TGCTTGGTTG
Dren19_Cf_BA   GGACCTTTGG GCATAATCTA CGCTATAATT AGAATTGGCG TGCTTGGTTG
Dren23_Cf_BA   GGACCTTTGG GTATAATCTA CGCTATAATT AGAATTGGCG TGCTTGGTTG
Dren25_Cf_SF   GGACCTTTGG GTATAATCTA CGCTATAATT AGAATTGGCG TGCTTGGTTG
Dren32_Cf_SC   GGACCTTTGG GTATAATCTA CGCTATAATT AGAATTGGCG TGCTTGGTTG
Dren33_Cf_SC   GGACCTTTGG GTATAATCTA CGCTATAATT AGAATTGGCG TGCTTGGTTG
Dren34_Cf_Pa   GGACCTTTGG GTATAATCTA CGCTATAATT AGAATTGGCG TGCTTGGTTG
Dren35_Cf_Pa   GGACCTTTGG GTATAATCTA CGCTATAATT AGAATTGGCG TGCTTGGTTG
Dren40_Cf_RG   GGACCTTTGG GTATAATCTA CGCTATAATT AGAATTGGCG TGCTTGGTTG
Dren41_Cf_RG   GGACCTTTGG GTATAATCTA CGCTATAATT AGAATTGGCG TGCTTGGTTG
Dren42_Cf_RG   GGGCCTTTGG GCATAATCTA CGCTATAATT AGAATTGGCG TGCTTGGTTG
Dren43_Cf_RG   GGACCTTTGG GTATAATCTA CGCTATAATT AGAATTGGCG TGCTTGGTTG
Dren45_Cf_RG   GGACCTTTGG GTATAATCTA CGCTATAATT AGAATTGGCG TGCTTGGTTG
Dren46_Cf_SF   GGACCTTTGG GTATAATCTA CGCTATAATT AGAATTGGCG TGCTTGGTTG
Dren47_Cb_SF   GGGCCTTTGG GCATAATCTA CGCTATAATT AGAATTGGCG TGCTTGGTTG
Dren48_Cf_BA   GGACCTTTGG GTATAATCTA CGCTATAATT AGAATTGGCG TGCTTGGTTG
Dren49_Cf_BA   GGACCTTTGG GTATAATCTA CGCTATAATT AGAATTGGCG TGCTTGGTTG
Dren50_Cf_Ch   GGACCTTTGG GTATAATCTA CGCTATAATT AGAATTGGCG TGCTTGGTTG
Dren53_Cf_BA   GGACCTTTGG GTATAATCTA CGCTATAATT AGAATTGGCG TGCTTGGTTG
Dren58_Cb_SF   GGACCTTTGG GTATAATCTA CGCTATAATT AGAATTGGCG TGCTTGGTTG
Dren66_Cf_SF   GGACCTTTGG GCATAATCTA CGCTATAATT AGAATTGGCG TGCTTGGTTG
Dren67_Cf_SF   GGGCCTTTGG GCATAATCTA CGCTATAATT AGAATTGGCG TGCTTGGTTG
Dren68_Cf_SF   GGGCCTTTGG GCATAATCTA CGCTATAATT AGAATTGGCG TGCTTGGTTG
Dren69_Cf_SF   GGGCCTTTGG GCATAATCTA CGCTATAATT AGAATTGGCG TGCTTGGTTG
Dren70_Cf_SF   GGACCTTTGG GTATAATCTA CGCTATAATT AGAATTGGCG TGCTTGGTTG
Dren72_Cb_SF   GGACCTTTGG GTATAATCTA CGCTATAATT AGAATTGGCG TGCTTGGTTG
Dren73_Cb_Ch   GGGCCTTTGG GCATAATCTA CGCTATAATT AGAATTGGCG TGCTTGGTTG
Dren74_Cb_Co   GGACCTTTGG GTATAATCTA CGCTATAATT AGAATTGGCG TGCTTGGTTG
Dren75_Cb_Co   GGACCTTTGG GTATAATCTA CGCTATAATT AGAATTGGCG TGCTTGGTTG
```

```
Dren80_Cf_SF   GGACCTTTGG GTATAATCTA CGCTATAATT AGAATTGGCG TGCTTGGTTG
Dren81_Cf_SF   GGACCTTTGG GTATAATCTA CGCTATAATT AGAATTGGCG TGCTTGGTTG
Dren82_Cb_Co   GGACCTTTGG GTATAATCTA CGCTATAATT AGAATTGGCG TGCTTGGTTG
Dren83_Cb_Ch   GGACCTTTGG GTATAATCTA TGCTATAATT AGAATTGGCG TGCTTGGTTG
Dren84_Cf_SF   GGACCTTTGG GTATAATCTA CGCTATAATT AGAATTGGCG TGCTTGGTTG
Dren85_Cb_SF   GGACCTTTGG GTATAATCTA CGCTATAATT AGAATTGGCG TGCTTGGTTG
Dren86_Cb_Co   GGGCCTTTGG GCATAATCTA CGCTATAATT AGAATTGGCG TGCTTGGTTG
Dren87_Cf_BA   GGGCCTTTGG GCATAATCTA CGCTATAATT AGAATTGGCG TGCTTGGTTG
Dren88_Lg_SF   GGGCCTTTGG GCATA-TCTA CGCTATAATT AGAATTGGCG TGCTTGGTTG

               ....|....| ....|....| ....|....| ....|....| ....|....|
                   805        815        825        835        845
Dren01_Cf_BA   TTTTGTGTGG GTACATCACA TGTTTACTGT AGGTTTAGAT GTTGATACGC
Dren02_Cf_BA   TTTTGTGTGG GTACATCACA TGTTTACTGT AGGTTTAGAT GTTGATACGC
Dren03_Cf_BA   TTTTGTGTGG GTACATCACA TGTTTACTGT AGGTTTAGAT GTTGATACGC
Dren05_Cf_BA   TTTTGTGTGG GTACATCACA TGTTTACTGT AGGTTTAGAT GTTGATACGC
Dren06_Cf_BA   TTTTGTGTGG GTACATCACA TGTTTACTGT AGGTTTAGAT GTTGATACGC
Dren07_Cf_BA   TTTTGTGTGG GTACATCACA TGTTTACTGT AGGTTTAGAT GTTGATACGC
Dren08_Cf_BA   TTTTGTGTGG GTACATCACA TGTTTACTGT AGGTTTAGAT GTTGATACGC
Dren09_Cf_BA   TTTTGTGTGG GTGCATCACA TGTTTACTGT AGGTTTAGAT GTTGATACGC
Dren10_Cf_BA   TTTTGTGTGG GTACATCACA TGTTTACTGT AGGTTTAGAT GTTGATACGC
Dren11_Cf_BA   TTTTGTGTGG GTACATCACA TGTTTACTGT AGGTTTAGAT GTTGATACGC
Dren18_Cf_BA   TTTTGTGTGG GTACATCACA TGTTTACTGT AGGTTTAGAT GTTGATACGC
Dren19_Cf_BA   TTTTGTGTGG GTACATCACA TGTTTACTGT AGGTTTAGAT GTTGATACGC
Dren23_Cf_BA   TTTTGTGTGG GTGCATCACA TGTTTACTGT AGGTTTAGAT GTTGATACGC
Dren25_Cf_SF   TTTTGTGTGG GTACATCACA TGTTTACTGT AGGTTTAGAT GTTGATACGC
Dren32_Cf_SC   TTTTGTGTGG GTACATCACA TGTTTACTGT AGGTTTAGAT GTTGATACGC
Dren33_Cf_SC   TTTTGTGTGG GTACATCACA TGTTTACTGT AGGTTTAGAT GTTGATACGC
Dren34_Cf_Pa   TTTTGTGTGG GTACATCACA TGTTTACTGT AGGTTTAGAT GTTGATACGC
Dren35_Cf_Pa   TTTTGTGTGG GTACATCACA TGTTTACTGT AGGTTTAGAT GTTGATACGC
Dren40_Cf_RG   TTTTGTGTGG GTACATCACA TGTTTACTGT AGGTTTAGAT GTTGATACGC
Dren41_Cf_RG   TTTTGTGTGG GTACATCACA TGTTTACTGT AGGTTTAGAT GTTGATACGC
Dren42_Cf_RG   TTTTGTGTGG GTACATCACA TGTTTACTGT AGGTTTAGAT GTTGATACGC
Dren43_Cf_RG   TTTTGTGTGG GTACATCACA TGTTTACTGT AGGTTTAGAT GTTGATACGC
Dren45_Cf_RG   TTTTGTGTGG GTACATCACA TGTTTACTGT AGGTTTAGAT GTTGATACGC
Dren46_Cf_SF   TTTTGTGTGG GTACATCACA TGTTTACTGT AGGTTTAGAT GTTGATACGC
Dren47_Cb_SF   TTTTGTGTGG GTACATCACA TGTTTACTGT AGGTTTAGAT GTTGATACGC
Dren48_Cf_BA   TTTTGTGTGG GTACATCACA TGTTTACTGT AGGTTTAGAT GTTGATACGC
Dren49_Cf_BA   TTTTGTGTGG GTGCATCACA TGTTTACTGT AGGTTTAGAT GTTGATACGC
Dren50_Cf_Ch   TTTTGTGTGG GTACATCACA TGTTTACTGT AGGTTTAGAT GTTGATACGC
Dren53_Cf_BA   TTTTGTGTGG GTACATCACA TGTTTACTGT AGGTTTAGAT GTTGATACGC
Dren58_Cb_SF   TTTTGTGTGG GTACATCACA TGTTTACTGT AGGTTTAGAT GTTGATACGC
Dren66_Cf_SF   TTTTGTGTGG GTACATCACA TGTTTACTGT AGGTTTAGAT GTTGATACGC
Dren67_Cf_SF   TTTTGTGTGG GTGCATCACA TGTTTACTGT AGGTTTAGAT GTTGATACGC
Dren68_Cf_SF   TTTTGTGTGG GTGCATCACA TGTTTACTGT AGGTTTAGAT GTTGATACGC
Dren69_Cf_SF   TTTTGTGTGG GTGCATCACA TGTTTACTGT AGGTTTAGAT GTTGATACGC
Dren70_Cf_SF   TTTTGTGTGG GTACATCACA TGTTTACTGT AGGTTTAGAT GTTGATACGC
Dren72_Cb_SF   TTTTGTGTGG GTACATCACA TGTTTACTGT AGGTTTAGAT GTTGATACGC
Dren73_Cb_Ch   TTTTGTGTGG GTACATCACA TGTTTACTGT AGGTTTAGAT GTTGATACGC
Dren74_Cb_Co   TTTTGTGTGG GTACATCACA TGTTTACTGT AGGTTTAGAT GTTGATACGC
Dren75_Cb_Co   TTTTGTGTGG GTACATCACA TGTTTACTGT AGGTTTAGAT GTTGATACGC
Dren80_Cf_SF   TTTTGTGTGG GTACATCACA TGTTTACTGT AGGTTTAGAT GTTGATACGC
Dren81_Cf_SF   TTTTGTGTGG GTACATCACA TGTTTACTGT AGGTTTAGAT GTTGATACGC
Dren82_Cb_Co   TTTTGTGTGG GTACATCACA TGTTTACTGT AGGTTTAGAT GTTGATACGC
```

```
Dren83_Cb_Ch   TTTTGTGTGG GTACATCACA TGTTTACTGT AGGTTTAGAT GTTGATACGC
Dren84_Cf_SF   TTTTGTGTGG GTACATCACA TGTTTACTGT AGGTTTAGAT GTTGATACGC
Dren85_Cb_SF   TTTTGTGTGG GTGCATCACA TGTTTACTGT AGGTTTAGAT GTTGATACGC
Dren86_Cb_Co   TTTTGTGTGG GTACATCACA TGTTTACTGT AGGTTTAGAT GTTGATACGC
Dren87_Cf_BA   TTTTGTGTGG GTACATCACA TGTTTACTGT AGGTTTAGAT GTTGATACGC
Dren88_Lg_SF   TTTTGTGTGG GTGCATCACA TGTTTACTGT AGGTTTAGAT GTTGATACGC

               ....|....| ....|....| ....|....| ....|....| ....|....|
                   855        865        875        885        895
Dren01_Cf_BA   GTGCGTATTT TGCTGCTGCG AGTATAATCA TCGGTATTCC GACGGGGGTA
Dren02_Cf_BA   GTGCGTATTT TGCTGCTGCG AGTATAATCA TCGGTATTCC GACGGGGGTA
Dren03_Cf_BA   GTGCGTATTT TGCTGCTGCG AGTATAATCA TCGGTATTCC GACGGGGGTA
Dren05_Cf_BA   GTGCGTATTT TGCTGCTGCG AGTATAATCA TCGGTATTCC GACAGGGGTA
Dren06_Cf_BA   GTGCGTATTT TGCTGCTGCG AGTATAATCA TCGGTATTCC GACGGGGGTA
Dren07_Cf_BA   GTGCGTATTT TGCTGCTGCG AGTATAATCA TCGGTATTCC GACAGGGGTA
Dren08_Cf_BA   GTGCGTATTT TGCTGCTGCG AGTATAATCA TCGGTATTCC GACGGGGGTA
Dren09_Cf_BA   GTGCGTATTT TGCTGCTGCG AGTATAATCA TCGGTATTCC GACGGGGGTA
Dren10_Cf_BA   GTGCGTATTT TGCTGCTGCG AGTATAATCA TCGGTATTCC GACGGGGGTA
Dren11_Cf_BA   GTGCGTATTT TGCTGCTGCG AGTATAATCA TCGGTATTCC GACGGGGGTA
Dren18_Cf_BA   GTGCGTATTT TGCTGCTGCG AGTATAATCA TCGGTATTCC GACAGGGGTA
Dren19_Cf_BA   GTGCGTATTT TGCTGCTGCG AGTATAATCA TCGGTATTCC GACAGGGGTA
Dren23_Cf_BA   GTGCGTATTT TGCTGCTGCG AGTATAATCA TCGGTATTCC GACGGGGGTA
Dren25_Cf_SF   GTGCGTATTT TGCTGCTGCG AGTATAATCA TCGGTATTCC GACGGGGGTA
Dren32_Cf_SC   GTGCGTATTT TGCTGCTGCG AGTATAATCA TCGGTATTCC GACGGGGGTA
Dren33_Cf_SC   GTGCGTATTT TGCTGCTGCG AGTATAATCA TCGGTATTCC GACGGGGGTA
Dren34_Cf_Pa   GTGCGTATTT TGCTGCTGCG AGTATAATCA TCGGTATTCC GACGGGGGTA
Dren35_Cf_Pa   GTGCGTATTT TGCTGCTGCG AGTATAATCA TCGGTATTCC GACGGGGGTA
Dren40_Cf_RG   GTGCGTATTT TGCTGCTGCG AGTATAATCA TCGGTATCCC GACGGGGGTA
Dren41_Cf_RG   GTGCGTATTT TGCTGCTGCG AGTATAATCA TCGGTATCCC GACGGGGGTA
Dren42_Cf_RG   GTGCGTATTT TGCTGCTGCG AGTATAATCA TCGGTATTCC GACAGGGGTA
Dren43_Cf_RG   GTGCGTATTT TGCTGCTGCG AGTATAATCA TCGGTATTCC GACGGGGGTA
Dren45_Cf_RG   GTGCGTATTT TGCTGCTGCG AGTATAATCA TCGGTATTCC GACGGGGGTA
Dren46_Cf_SF   GTGCGTATTT TGCTGCTGCG AGTATAATCA TCGGTATTCC GACGGGGGTA
Dren47_Cb_SF   GTGCGTATTT TGCTGCTGCG AGTATAATCA TCGGTATTCC GACAGGGGTA
Dren48_Cf_BA   GTGCGTATTT TGCTGCTGCG AGTATAATCA TCGGTATTCC GACGGGGGTA
Dren49_Cf_BA   GTGCGTATTT TGCTGCTGCG AGTATAATCA TCGGTATTCC GACGGGGGTA
Dren50_Cf_Ch   GTGCGTATTT TGCTGCTGCG AGTATAATCA TCGGTATTCC GACGGGGGTA
Dren53_Cf_BA   GTGCGTATTT TGCTGCTGCG AGTATAATCA TCGGTATTCC GACGGGGGTA
Dren58_Cb_SF   GTGCGTATTT TGCTGCTGCG AGTATAATCA TCGGTATCCC GACGGGGGTA
Dren66_Cf_SF   GTGCGTATTT TGCTGCTGCG AGTATAATCA TCGGTATTCC GACAGGGGTA
Dren67_Cf_SF   GTGCGTATTT TGCTGCTGCG AGTATAATCA TCGGTATTCC GACAGGGGTA
Dren68_Cf_SF   GTGCGTATTT TGCTGCTGCG AGTATAATCA TCGGTATTCC GACAGGGGTA
Dren69_Cf_SF   GTGCGTATTT TGCTGCTGCG AGTATAATCA TCGGTATTCC GACAGGGGTA
Dren70_Cf_SF   GTGCGTATTT TGCTGCTGCG AGTATAATCA TCGGTATTCC GACGGGGGTA
Dren72_Cb_SF   GTGCGTATTT TGCTGCTGCG AGTATAATCA TCGGTATTCC GACGGGGGTA
Dren73_Cb_Ch   GTGCGTATTT TGCTGCTGCG AGTATAATCA TCGGTATTCC GACAGGGGTA
Dren74_Cb_Co   GTGCGTATTT TGCTGCTGCG AGTATAATCA TCGGTATTCC GACGGGGGTA
Dren75_Cb_Co   GTGCGTATTT TGCTGCTGCG AGTATAATCA TCGGTATTCC GACGGGGGTA
Dren80_Cf_SF   GTGCGTATTT TGCTGCTGCG AGTATAATCA TCGGTATTCC GACGGGGGTA
Dren81_Cf_SF   GTGCGTATTT TGCTGCTGCG AGTATAATCA TCGGTATTCC GACGGGGGTA
Dren82_Cb_Co   GTGCGTATTT TGCTGCTGCG AGTATAATCA TCGGTATTCC GACGGGGGTA
Dren83_Cb_Ch   GTGCGTATTT TGCTGCTGCG AGTATAATCA TCGGTATTCC GACGGGGGTA
Dren84_Cf_SF   GTGCGTATTT TGCTGCTGCG AGTATAATCA TCGGTATTCC GACGGGGGTA
Dren85_Cb_SF   GTGCGTATTT TGCTGCTGCG AGTATAATCA TCGGTATTCC GACGGGGGTA
```

```
Dren86_Cb_Co   GTGCGTATTT TGCTGCTGCG AGTATAATCA TCGGTATTCC GACAGGGGTA
Dren87_Cf_BA   GTGCGTATTT TGCTGCTGCG AGTATAATCA TCGGTATCCC GACAGGGGTA
Dren88_Lg_SF   GTGCGTATTT TGCTGCTGCG AGTATAATCA TCGGTATTCC GACAGGGGTA

               ....|....| ....|....| ....|....| ....|....| ....|....|
                   905        915        925        935        945
Dren01_Cf_BA   AAAGTATTTA GTTGATTAGC TACTTTGTAT GGTGGTCAGG TTATCTTTTC
Dren02_Cf_BA   AAAGTATTTA GTTGATTAGC TACTTTGTAT GGTGGTCAGG TTATCTTTTC
Dren03_Cf_BA   AAAGTATTTA GTTGGTTAGC TACTTTGTAT GGTGGTCAGG TTATCTTTTC
Dren05_Cf_BA   AAAGTATTTA GTTGATTAGC TACTTTGTAT GGTGGTCAGG TTATCTTTTC
Dren06_Cf_BA   AAAGTATTTA GTTGATTAGC TACTTTGTAT GGTGGTCAGG TTATCTTTTC
Dren07_Cf_BA   AAAGTATTTA GTTGATTAGC TACTTTGTAT GGTGGTCAGG TTATCTTTTC
Dren08_Cf_BA   AAAGTATTTA GTTGATTAGC TACTTTGTAT GGTGGTCAGG TTATCTTTTC
Dren09_Cf_BA   AAAGTATTTA GCTGATTAGC TACTTTGTAT GGTGGTCAGG TTATCTTTTC
Dren10_Cf_BA   AAAGTATTTA GTTGATTAGC TACTTTGTAT GGTGGTCAGG TTATCTTTTC
Dren11_Cf_BA   AAAGTATTTA GTTGATTAGC TACTTTGTAT GGTGGTCAGG TTATCTTTTC
Dren18_Cf_BA   AAAGTATTTA GTTGATTAGC TACTTTGTAT GGTGGTCAGG TTATCTTTTC
Dren19_Cf_BA   AAAGTATTTA GTTGATTAGC TACTTTGTAT GGTGGTCAGG TTATCTTTTC
Dren23_Cf_BA   AAAGTATTTA GCTGATTAGC TACTTTGTAT GGTGGTCAGG TTATCTTTTC
Dren25_Cf_SF   AAAGTATTTA GTTGATTAGC TACTTTGTAT GGTGGTCAGG TTATCTTTTC
Dren32_Cf_SC   AAAGTATTTA GTTGATTAGC TACTTTGTAT GGTGGTCAGG TTATCTTTTC
Dren33_Cf_SC   AAAGTATTTA GTTGATTAGC TACTTTGTAT GGTGGTCAGG TTATCTTTTC
Dren34_Cf_Pa   AAAGTATTTA GTTGATTAGC TACTTTGTAT GGTGGTCAGG TTATCTTTTC
Dren35_Cf_Pa   AAAGTATTTA GTTGATTAGC TACTTTGTAT GGTGGTCAGG TTATCTTTTC
Dren40_Cf_RG   AAAGTATTTA GTTGATTAGC TACTTTGTAT GGTGGTCAGG TTATCTTTTC
Dren41_Cf_RG   AAAGTATTTA GTTGATTAGC TACTTTGTAT GGTGGTCAGG TTATCTTTTC
Dren42_Cf_RG   AAAGTATTTA GTTGATTAGC TACTTTGTAT GGTGGTCAGG TTATCTTTTC
Dren43_Cf_RG   AAAGTATTTA GTTGATTAGC TACTTTGTAT GGTGGTCAGG TTATCTTTTC
Dren45_Cf_RG   AAAGTATTTA GTTGATTAGC TACTTTGTAT GGTGGTCAGG TTATCTTTTC
Dren46_Cf_SF   AAAGTATTTA GTTGATTAGC TACTTTGTAT GGTGGTCAGG TTATCTTTTC
Dren47_Cb_SF   AAAGTATTTA GTTGATTAGC TACTTTGTAT GGTGGTCAGG TTATCTTTTC
Dren48_Cf_BA   AAAGTATTTA GTTGATTAGC TACTTTGTAT GGTGGTCAGG TTATCTTTTC
Dren49_Cf_BA   AAAGTATTTA GCTGATTAGC TACTTTGTAT GGTGGTCAGG TTATCTTTTC
Dren50_Cf_Ch   AAAGTATTTA GTTGATTAGC TACTTTGTAT GGTGGTCAGG TTATCTTTTC
Dren53_Cf_BA   AAAGTATTTA GTTGATTAGC TACTTTGTAT GGTGGTCAGG TTATCTTTTC
Dren58_Cb_SF   AAAGTATTTA GTTGATTAGC TACTTTGTAT GGTGGTCAGG TTATCTTTTC
Dren66_Cf_SF   AAAGTATTTA GTTGATTAGC TACTTTGTAT GGTGGTCAGG TTATCTTTTC
Dren67_Cf_SF   AAAGTATTTA GTTGATTAGC TACTTTGTAT GGTGGTCAGG TTATCTTTTC
Dren68_Cf_SF   AAAGTATTTA GTTGATTAGC TACTTTGTAT GGTGGTCAGG TTATCTTTTC
Dren69_Cf_SF   AAAGTATTTA GTTGATTAGC TACTTTGTAT GGTGGTCAGG TTATCTTTTC
Dren70_Cf_SF   AAAGTATTTA GTTGATTAGC TACTTTGTAT GGTGGTCAGG TTATCTTTTC
Dren72_Cb_SF   AAAGTATTTA GTTGATTAGC TACTTTGTAT GGTGGTCAGG TTATCTTTTC
Dren73_Cb_Ch   AAAGTATTTA GTTGATTAGC TACTTTGTAT GGTGGTCAGG TTATCTTTTC
Dren74_Cb_Co   AAAGTATTTA GTTGATTAGC TACTTTGTAT GGTGGTCAGG TTATCTTTTC
Dren75_Cb_Co   AAAGTATTTA GTTGATTAGC TACTTTGTAT GGTGGTCAGG TTATCTTTTC
Dren80_Cf_SF   AAAGTATTTA GTTGATTAGC TACTTTGTAT GGTGGTCAGG TTATCTTTTC
Dren81_Cf_SF   AAAGTATTTA GTTGATTAGC TACTTTGTAT GGTGGTCAGG TTATCTTTTC
Dren82_Cb_Co   AAAGTATTTA GTTGATTAGC TACTTTGTAT GGTGGTCAGG TTATCTTTTC
Dren83_Cb_Ch   AAAGTATTTA GTTGGTTAGC TACTTTGTAT GGTGGTCAGG TTATCTTTTC
Dren84_Cf_SF   AAAGTATTTA GTTGATTAGC TACTTTGTAT GGTGGTCAGG TTATCTTTTC
Dren85_Cb_SF   AAAGTATTTA GCTGATTAGC TACTTTGTAT GGTGGTCAGG TTATCTTTTC
Dren86_Cb_Co   AAAGTATTTA GTTGATTAGC TACTTTGTAT GGTGGTCAGG TTATCTTTTC
Dren87_Cf_BA   AAAGTATTTA GTTGATTAGC TACTTTGTAT GGTGGTCAGG TTATCTTTTC
Dren88_Lg_SF   AAAGTATTTA GTTGATTAGC TACTTTGTAT GGTGGTCAGG TTATCTTTTC
```

```
                ....|....| ....|....| ....|....| ....|..
                  955        965        975        985
Dren01_Cf_BA   ACCTTTGTAT TGTTGGACTG TAGGGTTTAT TTCTGTG
Dren02_Cf_BA   ACCTTTGTAT TGTTGGACTG TAGGGTTTAT TTCTGTG
Dren03_Cf_BA   ACCTTTGTAT TGTTGGACTG TAGGGTTTAT TTCTGTG
Dren05_Cf_BA   ACCTTTGTAT TGTTGGACTG TAGGGTTTAT TTCTGTG
Dren06_Cf_BA   ACCTTTGTAT TGTTGGACTG TAGGGTTTAT TTCTGTG
Dren07_Cf_BA   ACCTTTGTAT TGTTGGACTG TAGGGTTTAT TTCTGTG
Dren08_Cf_BA   ACCTTTGTAT TGTTGGACTG TAGGGTTTAT TTCTGTG
Dren09_Cf_BA   ACCTTTGTAT TGTTGGACTG TAGGGTTTAT TTCTGTG
Dren10_Cf_BA   ACCTTTGTAT TGTTGGACTG TAGGGTTTAT TTCTGTG
Dren11_Cf_BA   ACCTTTGTAT TGTTGGACTG TAGGGTTTAT TTCTGTG
Dren18_Cf_BA   ACCTTTGTAT TGTTGGACTG TAGGGTTTAT TTCTGTG
Dren19_Cf_BA   ACCTTTGTAT TGTTGGACTG TAGGGTTTAT TTCTGTG
Dren23_Cf_BA   ACCTTTGTAT TGTTGGACTG TAGGGTTTAT TTCTGTG
Dren25_Cf_SF   ACCTTTGTAT TGTTGGACTG TAGGGTTTAT TTCTGTG
Dren32_Cf_SC   ACCTTTGTAT TGTTGGACTG TAGGGTTTAT TTCTGTG
Dren33_Cf_SC   ACCTTTGTAT TGTTGGACTG TAGGGTTTAT TTCTGTG
Dren34_Cf_Pa   ACCTTTGTAT TGTTGGACTG TAGGGTTTAT TTCTGTG
Dren35_Cf_Pa   ACCTTTGTAT TGTTGGACTG TAGGGTTTAT TTCTGTG
Dren40_Cf_RG   ACCTTTGTAT TGTTGGACTG TAGGGTTTAT TTCTGTG
Dren41_Cf_RG   ACCTTTGTAT TGTTGGACTG TAGGGTTTAT TTCTGTG
Dren42_Cf_RG   ACCTTTGTAT TGTTGGACTG TAGGGTTTAT TTCTGTG
Dren43_Cf_RG   ACCCTTGTAT TGTTGGACTG TAGGGTTTAT TTCTGTG
Dren45_Cf_RG   ACCCTTGTAT TGTTGGACTG TAGGGTTTAT TTCTGTG
Dren46_Cf_SF   ACCTTTGTAT TGTTGGACTG TAGGGTTTAT TTCTGTG
Dren47_Cb_SF   ACCTTTGTAT TGTTGGACTG TAGGGTTTAT TTCTGTG
Dren48_Cf_BA   ACCTTTGTAT TGTTGGACTG TAGGGTTTAT TTCTGTG
Dren49_Cf_BA   ACCTTTGTAT TGTTGGACTG TAGGGTTTAT TTCTGTG
Dren50_Cf_Ch   ACCTTTGTAT TGTTGGACTG TAGGGTTTAT TTCTGTG
Dren53_Cf_BA   ACCTTTGTAT TGTTGGACTG TAGGGTTTAT TTCTGTG
Dren58_Cb_SF   ACCTTTGTAT TGTTGGACTG TAGGGTTTAT TTCTGTG
Dren66_Cf_SF   ACCTTTGTAT TGTTGGACTG TAGGGTTTAT TTCTGTG
Dren67_Cf_SF   ACCTTTGTAT TGTTGGACTG TAGGGTTTAT TTCTGTG
Dren68_Cf_SF   ACCTTTGTAT TGTTGGACTG TAGGGTTTAT TTCTGTG
Dren69_Cf_SF   ACCTTTGTAT TGTTGGACTG TAGGGTTTAT TTCTGTG
Dren70_Cf_SF   ACCTTTGTAT TGTTGGACTG TAGGGTTTAT TTCTGTG
Dren72_Cb_SF   ACCTTTGTAT TGTTGGACTG TAGGGTTTAT TTCTGTG
Dren73_Cb_Ch   ACCTTTGTAT TGTTGGACTG TAGGGTTTAT TTCTGTG
Dren74_Cb_Co   ACCTTTGTAT TGTTGGACTG TAGGGTTTAT TTCTGTG
Dren75_Cb_Co   ACCTTTGTAT TGTTGGACTG TAGGGTTTAT TTCTGTG
Dren80_Cf_SF   ACCTTTGTAT TGTTGGACTG TAGGGTTTAT TTCTGTG
Dren81_Cf_SF   ACCTTTGTAT TGTTGGACTG TAGGGTTTAT TTCTGTG
Dren82_Cb_Co   ACCTTTGTAT TGTTGGACTG TAGGGTTTAT TTCTGTG
Dren83_Cb_Ch   ACCTTTGTAT TGTTGGACTG TAGGGTTTAT TTCTGTG
Dren84_Cf_SF   ACCTTTGTAT TGTTGGACTG TAGGGTTTAT TTCTGTG
Dren85_Cb_SF   ACCTTTGTAT TGTTGGACTG TAGGGTTTAT TTCTGTG
Dren86_Cb_Co   ACCTTTGTAT TGTTGGACTG TAGGGTTTAT TTCTGTG
Dren87_Cf_BA   ACCTTTGTAT TGTTGGACTG TAGGGTTTAT TTCTGTG
Dren88_Lg_SF   ACCTTTGTAT TGTTGGACTG TAGGGTTTAT TTCTGTG
```

ND4 Multiple Sequence Alignment

```
                ....|....| ....|....| ....|....| ....|....| ....|....|
                    5         15         25         35         45

Dren02_Cf_BA   GCTTTATACT TTAAGCTTAT CAGTCCCGTT ATTTGTACTG CTACTGAACC
Dren07_Cf_BA   GCTTTATACT TTGAGCTTAT CAGTCCCGTT ATTTGTACTG CTACTGAACC
Dren08_Cf_BA   GCTTTATACT TTAAGCTTAT CAGTCCCGTT ATTTGTACTG CTACTGAACC
Dren09_Cf_BA   GCTTTATACT TTGAGCTTAT CAGTCCCGTT ATTTGTACTG CTACTGAACC
Dren10_Cf_BA   GCTTTATACT TTGAGCTTAT CAATCCCGTT ATTTGTATTG CTACTGAACC
Dren18_Cf_BA   GCTTTATACT TTGAGCTTAT CAGTCCCGTT ATTTGTACTG CTACTGAACC
Dren19_Cf_BA   GCTTTATACT TTGAGCTTAT CAGTCCCGTT ATTTGTACTG CTACTGAACC
Dren23_Cf_BA   GCTTTATACT TTGAGCTTAT CAGTCCCGTT ATTTGTACTG CTACTGAACC
Dren25_Cf_SF   GCTTTATACT TTGAGCTTAT CAGTCCCGTT ATTTGTACTG CTACTGAACC
Dren32_Cf_SC   GCTTTATACT TTGAGCTTAT CAGTCCCGTT ATTTGTACTG CTACTGAACC
Dren35_Cf_Pa   GCTTTATACT TTGAGCTTAT CAGTCCCGTT ATTTGTACTG CTACTGAACC
Dren40_Cf_RG   GCTTTATACT TTGAGCTTAT CAGTTCCGTT ATTTGTTCTG CTACTGAACC
Dren41_Cf_RG   GCTTTATACT TTGAGCTTAT CAGTTCCGTT ATTTGTTCTG CTACTGAACC
Dren42_Cf_RG   GCTTTATACT TTGAGCTTAT CAGTCCCGTT ATTTGTACTG CTACTGAACC
Dren43_Cf_RG   GCTTTATACT TTGAGCTTAT CAGTCCCGTT ATTTGTACTA CTACTGAACC
Dren45_Cf_RG   GCTTTATACT TTGAGCTTAT CAGTCCCGTT ATTTGTACTA CTACTGAACC
Dren46_Cf_SF   GCTTTATACT TTGAGCTTAT CAGTCCCGTT ATTTGTACTG CTACTGAACC
Dren47_Cb_SF   GCTTTATACT TTGAGCTTAT CAGTCCCGTT ATTTGTATTG CTACTGAACC
Dren48_Cf_BA   GCTTTATACT TTAAGCTTAT CAGTCCCGTT ATTTGTACTG CTACTGAACC
Dren49_Cf_BA   GCTTTATACT TTGAGCTTAT CAGTCCCGTT ATTTGTACTG CTACTGAACC
Dren50_Cf_Ch   GCTTTATACT TTAAGCTTAT CAGTCCCGTT ATTTGTACTG CTACTGAACC
Dren53_Cf_BA   GCTTTATACT TTAAGCTTAT CAGTCCCGTT ATTTGTACTG CTACTGAACC
Dren58_Cb_SF   GCTTTATACT TTGAGCTTAT CAGTTCCGTT ATTTGTTCTG CTACTGAACC
Dren66_Cf_SF   GCTTTATACT TTGAGCTTAT CAGTCCCGTT ATTTGTACTG CTACTGAACC
Dren68_Cf_SF   GCTTTATACT TTGAGCTTAT CAGTCCCGTT ATTTGTATTG CTACTGAACC
Dren70_Cf_SF   GCTTTATACT TTGAGCTTAT CAGTCCCGTT ATTTGTACTG CTACTGAACC
Dren72_Cb_SF   GCTTTATACT TTAAGCTTAT CAGTCCCGTT ATTTGTACTG CTACTGAACC
Dren73_Cb_Ch   GCTTTATACT TTGAGCTTAT CAGTCCCGTT ATTTGTATTG CTACTGAACC
Dren74_Cb_Co   GCTTTATACT TTGAGCTTAT CAGTCCCGTT ATTTGTACTG CTACTGAACC
Dren75_Cb_Co   GCTTTATACT TTAAGCTTAT CAGTCCCGTT ATTTGTACTG CTACTGAACC
Dren80_Cf_SF   GCTTTATACT TTGAGCTTAT CAGTCCCGTT ATTTGTACTG CTACTGAACC
Dren81_Cf_SF   GCTTTATACT TTGAGCTTAT CAGTCCCGTT ATTTGTACTG CTACTGAACC
Dren82_Cb_Co   GCTTTATACT TTGAGCTTAT CAGTCCCGTT ATTTGTACTG CTACTGAACC
Dren83_Cb_Ch   GCTTTATACT TTAAGCTTAT CAGTCCCGTT ATTTGTACTG CTACTGAACC
Dren84_Cf_SF   GCTTTATACT TTGAGCTTAT CAGTCCCGTT ATTTGTACTG CTACTGAACC
Dren85_Cb_SF   GCTTTATACT TTGAGCTTAT CAGTCCCGTT ATTTGTACTG CTACTGAACC
Dren67_Cf_SF   GCTTTATACT TTGAGCTTAT CAGTCCCGTT ATTTGTATTG CTACTGAACC
Dren69_Cf_SF   GCTTTATACT TTGAGCTTAT CAGTCCCGTT ATTTGTATTG CTACTGAACC
Dren86_Cb_Co   GCTTTATACT TTGAGCTTAT CAGTCCCGTT ATTTGTACTG CTACTGAACC
Dren87_Cf_BA   GCTTTATACT TTGAGCTTAT CAGTCCCGTT ATTTGTACTG CTACTGAACC
Dren88_Lg_SF   GCTTTATACT TTGAGCTTAT CAGTCCCGTT ATTTGTATTG CTACTGAACC

                ....|....| ....|....| ....|....| ....|....| ....|....|
                    55         65         75         85         95

Dren02_Cf_BA   TAAATAATTT TAGCTGTCAA AACAGCTGAC CATCAAACCG CAATCTTTCA
Dren07_Cf_BA   TAAATAATTT TAGCTGTCAA AACAGCTGAC CATCAAACCG CAATCTTTCA
Dren08_Cf_BA   TAAATAATTT TAGCTGTCAA AACAGCTGAC CATCAAACCG CAATCTTTCA
Dren09_Cf_BA   TAAATAATTT TAGCTGTCAA AACAGCTGAC CATCAAACCG CAATCTTTCA
Dren10_Cf_BA   TAAATAATTT TAGCTGTCAA AACAGCTGAC CATCAAACCG CAATCTTTCA
Dren18_Cf_BA   TAAATAATTT TAGCTGTCAA AACAGCTGAC CATCAAACCG CAATCTTTCA
Dren19_Cf_BA   TAAATAATTT TAGCTGTCAA AACAGCTGAC CATCAAACCG CAATCTTTCA
Dren23_Cf_BA   TAAATAATTT TAGCTGTCAA AACAGCTGAC CATCAAACCG CAATCTTTCA
Dren25_Cf_SF   TAAATAATTT TAGCTGTCAA AACAGCTGAC CATCAAACCG CAATCTTTCA
Dren32_Cf_SC   TAAATAATTT TAGCTGTCAA AACAGCTGAC CATCAAACCG CAATCTTTCA
Dren35_Cf_Pa   TAAATAATTT TAGCTGTCAA AACAGCTGAC CATCAAACCG CAATCTTTCA
Dren40_Cf_RG   TAAATAATTT TAGCTGTCAA AACAGCTGAC CATCAAACCG CAATCTTTCA
```

```
Dren41_Cf_RG   TAAATAATTT TAGCTGTCAA AACAGCTGAC CATCAAACCG CAATCTTTCA
Dren42_Cf_RG   TAAATAATTT TAGCTGTCAA AACAGCTGAC CATCAAACCG CAATCTTTCA
Dren43_Cf_RG   TAAATAATTT TAGCTGTCAA AACAGCTGAC CATCAAACCG CAATCTTTCA
Dren45_Cf_RG   TAAATAATTT TAGCTGTCAA AACAGCTGAC CATCAAACCG CAATCTTTCA
Dren46_Cf_SF   TAAATAATTT TAGCTGTCAA AACAGCTGAC CATCAAACCG CAATCTTTCA
Dren47_Cb_SF   TAAATAATTT TAGCTGTCAA AACAGCTGAC CATCAAACCG CAATCTTTCA
Dren48_Cf_BA   TAAATAATTT TAGCTGTCAA AACAGCTGAC CATCAAACCG CAATCTTTCA
Dren49_Cf_BA   TAAATAATTT TAGCTGTCAA AACAGCTGAC CATCAAACCG CAATCTTTCA
Dren50_Cf_Ch   TAAATAATTT TAGCTGTCAA AACAGCTGAC CATCAAACCG CAATCTTTCA
Dren53_Cf_BA   TAAATAATTT TAGCTGTCAA AACAGCTGAC CATCAAACCG CAATCTTTCA
Dren58_Cb_SF   TAAATAATTT TAGCTGTCAA AACAGCTGAC CATCAAACCG CAATCTTTCA
Dren66_Cf_SF   TAAATAATTT TAGCTGTCAA AACAGCTGAC CATCAAACCG CAATCTTTCA
Dren68_Cf_SF   TAAATAATTT TAGCTGTCAA AACAGCTGAC CATCAAACCG CAATCTTTCA
Dren70_Cf_SF   TAAATAATTT TAGCTGTCAA AACAGCTGAC CATCAAACCG CAATCTTTCA
Dren72_Cb_SF   TAAATAATTT TAGCTGTCAA AACAGCTGAC CATCAAACCG CAATCTTTCA
Dren73_Cb_Ch   TAAATAATTT TAGCTGTCAA AACAGCTGAC CATCAAACCG CAATCTTTCA
Dren74_Cb_Co   TAAATAATTT TAGCTGTCAA AACAGCTGAC CATCAAACCG CAATCTTTCA
Dren75_Cb_Co   TAAATAATTT TAGCTGTCAA AACAGCTGAC CATCAAACCG CAATCTTTCA
Dren80_Cf_SF   TAAATAATTT TAGCTGTCAA AACAGCTGAC CATCAAACCG CAATCTTTCA
Dren81_Cf_SF   TAAATAATTT TAGCTGTCAA AACAGCTGAC CATCAAACCG CAATCTTTCA
Dren82_Cb_Co   TAAATAATTT TAGCTGTCAA AACAGCTGAC CATCAAACCG CAATCTTTCA
Dren83_Cb_Ch   TAAATAATTT TAGCTGTCAA AACAGCTGAC CATCAAACCG CAATCTTTCA
Dren84_Cf_SF   TAAATAATTT TAGCTGTCAA AACAGCTGAC CATCAAACCG CAATCTTTCA
Dren85_Cb_SF   TAAATAATTT TAGCTGTCAA AACAGCTGAC CATCAAACCG CAATCTTTCA
Dren67_Cf_SF   TAAATAATTT TAGCTGTCAA AACAGCTGAC CATCAAACCG CAATCTTTCA
Dren69_Cf_SF   TAAATAATTT TAGCTGTCAA AACAGCTGAC CATCAAACCG CAATCTTTCA
Dren86_Cb_Co   TAAATAATTT TAGCTGTCAA AACAGCTGAC CATCAAACCG CAATCTTTCA
Dren87_Cf_BA   TAAATAATTT TAGCTGTCAA AACAGCTGAC CATCAAACCG CAATCTTTCA
Dren88_Lg_SF   TAAATAATTT TAGCTGTCAA AACAGCTGAC CATCAAACCG CAATCTTTCA

               ....|....| ....|....| ....|....| ....|....| ....|....|
                   105        115        125        135        145
Dren02_Cf_BA   TTTATTATGA TAACCATAAT CACCATACCC TTTTTAATTA AGATGCCACT
Dren07_Cf_BA   TTTATTATGA TAACCATAAT CACCATACCC TTTTTAATTA AGATGCCACT
Dren08_Cf_BA   TTTATTATGA TAACCATAAT CACCATACCC TTTTTAATTA AGATGCCACT
Dren09_Cf_BA   TTTATTATGA TAACCATAAT CACCATACCC TTTTTAATTA AGATGCCACT
Dren10_Cf_BA   TTTATTATGA TAATCATAAT CACCATACCC TTTTTAATTA AGATGCCACT
Dren18_Cf_BA   TTTATTATGA TAACCATAAT CACCATGCCC TTTTTAATTA AGATGCCACT
Dren19_Cf_BA   TTTATTATGA TAACCATAAT CACCATGCCC TTTTTAATTA AGATGCCACT
Dren23_Cf_BA   TTTATTATGA TAACCATAAT CACCATACCC TTTTTAATTA AGATGCCACT
Dren25_Cf_SF   TTTATTATGA TAACCATAAT CACCATACCC TTTTTAATTA AGATGCCACT
Dren32_Cf_SC   TTTATTATGA TAACCATAAT CACCATACCC TTTTTAATTA AGATGCCACT
Dren35_Cf_Pa   TTTATTATGA TAACCATAAT CACCATACCC TTTTTAATTA AGATGCCACT
Dren40_Cf_RG   TTTATCATGA TAACCATAAT CACCATACCC TTTCTAATTA AGATGCCACT
Dren41_Cf_RG   TTTATCATGA TAACCATAAT CACCATACCC TTTCTAATTA AGATGCCACT
Dren42_Cf_RG   TTTATTATGA CAATCATAAT CACCATACCC TTTTTAATTA AGATGCCACT
Dren43_Cf_RG   TTTATTATGA TAACCATAAT CACCATACCC TTTTTAATTA AGATGCCACT
Dren45_Cf_RG   TTTATTATGA TAACCATAAT CACCATACCC TTTTTAATTA AGATGCCACT
Dren46_Cf_SF   TTTATTATGA TAACCATAAT CACCATACCC TTTTTAATTA AGATGCCACT
Dren47_Cb_SF   TTTATTATGA TAATCATAAT CACCATACCC TTTTTAATTA AGATGCCACT
Dren48_Cf_BA   TTTATTATGA TAACCATAAT CACCATACCC TTTTTAATTA AGATGCCACT
Dren49_Cf_BA   TTTATTATGA TAACCATAAT CACCATACCC TTTTTAATTA AGATGCCACT
Dren50_Cf_Ch   TTTATTATGA TAACCATAAT CACCATACCC TTTTTAATTA AGATGCCACT
Dren53_Cf_BA   TTTATTATGA TAACCATAAT CACCATACCC TTTTTAATTA AGATGCCACT
Dren58_Cb_SF   TTTATCATGA TAACCATAAT CACCATACCC TTTCTAATTA AGATGCCACT
Dren66_Cf_SF   TTTATTATGA TAACCATAAT CACCATGCCC TTTTTAATTA AGATGCCACT
Dren68_Cf_SF   TTTATTATGA TAATCATAAT CACCATACCC TTTTTAATTA AGATGCCACT
Dren70_Cf_SF   TTTATTATGA TAACCATAAT CACCATACCC TTTTTAATTA AGATGCCACT
Dren72_Cb_SF   TTTATTATGA TAACCATAAT CACCATACCC TTTTTAATTA AGATGCCACT
Dren73_Cb_Ch   TTTATTATGA TAATCATAAT CACCATACCC TTTTTAATTA AGATGCCACT
Dren74_Cb_Co   TTTATTATGA TAACCATAAT CACCATACCC TTTTTAATTA AGATGCCACT
Dren75_Cb_Co   TTTATTATGA TAACCATAAT CACCATACCC TTTTTAATTA AGATGCCACT
Dren80_Cf_SF   TTTATTATGA TAACCATAAT CACCATACCC TTTTTAATTA AGATGCCACT
Dren81_Cf_SF   TTTATTATGA TAACCATAAT CACCATACCC TTTTTAATTA AGATGCCACT
```

```
Dren82_Cb_Co   TTTATTATGA TAACCATAAT CACCATACCC TTTTTAATTA AGATGCCACT
Dren83_Cb_Ch   TTTATTATGA TAACCATAAT CACCATACCC TTTTTAATTA AGATGCCACT
Dren84_Cf_SF   TTTATTATGA TAACCATAAT CACCATACCC TTTTTAATTA AGATGCCACT
Dren85_Cb_SF   TTTATTATGA TAACCATAAT CACCATACCC TTTTTAATTA AGATGCCACT
Dren67_Cf_SF   TTTATTATGA TAATCATAAT CACCATACCC TTTTTAATTA AGATGCCACT
Dren69_Cf_SF   TTTATTATGA TAATCATAAT CACCATACCC TTTTTAATTA AGATGCCACT
Dren86_Cb_Co   TTTATTATGA TAATCATAAT CACCATACCC TTTTTAATTA AGATGCCACT
Dren87_Cf_BA   TTTATTATGA TAATCATAAT CACCATACCC TTTTTAATTA AGATGCCACT
Dren88_Lg_SF   TTTATTATGA TAATCATAAT CACCATACCC TTTTTAATTA AGATGCCACT

               ....|....| ....|....| ....|....| ....|....| ....|....|
                   155        165        175        185        195
Dren02_Cf_BA   ATTTAGTGTC CATATATGAC TACCCAAAGC GCATGTGGAA AGACCAACAG
Dren07_Cf_BA   ATTTAGTGTC CATATATGAC TACCCAAAGC GCATGTGGAA AGACCAACAG
Dren08_Cf_BA   ATTTAGTGTC CATATATGAC TACCCAAAGC GCATGTGGAA AGACCAACAG
Dren09_Cf_BA   ATTTAGTGTT CATATATGAC TACCCAAAGC GCATGTGGAA AGACCAACAG
Dren10_Cf_BA   ATTTAGTGTC CACATATGAC TACCCAAAGC GCATGTGGAA AGACCAACAG
Dren18_Cf_BA   ATTTAGTGTC CATATATGAC TACCCAAAGC GCATGTGGAA AGACCAACAG
Dren19_Cf_BA   ATTTAGTGTC CATATATGAC TACCCAAAGC GCATGTGGAA AGACCAACAG
Dren23_Cf_BA   ATTTAGTGTT CATATATGAC TACCCAAAGC GCATGTGGAA AGACCAACAG
Dren25_Cf_SF   ATTTAGTGTC CATATATGAC TACCCAAAGC GCATGTGGAA AGACCAACAG
Dren32_Cf_SC   ATTTAGTGTC CATATATGAC TACCCAAAGC GCATGTGGAA AGACCAACAG
Dren35_Cf_Pa   ATTTAGTGTC CATATATGAC TACCCAAAGC GCATGTGGAA AGACCAACAG
Dren40_Cf_RG   ATTTAGTGTC CATATATGAC TACCCAAAGC GCATGTGGAA AGACCAACAG
Dren41_Cf_RG   ATTTAGTGTC CATATATGAC TACCCAAAGC GCATGTGGAA AGACCAACAG
Dren42_Cf_RG   ATTTAGTGTC CATATATGAC TACCCAAAGC GCATGTGGAA AGACCAACAG
Dren43_Cf_RG   ATTTAGTGTC CATATATGAC TACCCAAAGC GCATGTGGAA AGACCAACAG
Dren45_Cf_RG   ATTTAGTGTC CATATATGAC TACCCAAAGC GCATGTGGAA AGACCAACAG
Dren46_Cf_SF   ATTTAGTGTC CATATATGAC TACCCAAAGC GCATGTGGAA AGACCAACAG
Dren47_Cb_SF   ATTTAGTGTC CACATATGAC TACCCAAAGC GCATGTGGAA AGACCAACAG
Dren48_Cf_BA   ATTTAGTGTC CATATATGAC TACCCAAAGC GCATGTGGAA AGACCAACAG
Dren49_Cf_BA   ATTTAGTGTT CATATATGAC TACCCAAAGC GCATGTGGAA AGACCAACAG
Dren50_Cf_Ch   ATTTAGTGTC CATATATGAC TACCCAAAGC GCATGTGGAA AGACCAACAG
Dren53_Cf_BA   ATTTAGTGTC CATATATGAC TACCCAAAGC GCATGTGGAA AGACCAACAG
Dren58_Cb_SF   ATTTAGTGTC CATATATGAC TACCCAAAGC GCATGTGGAA AGACCAACAG
Dren66_Cf_SF   ATTTAGTGTC CATATATGAC TACCCAAAGC GCATGTGGAA AGACCAACAG
Dren68_Cf_SF   ATTTAGTGTC CACATATGAC TACCCAAAGC GCATGTGGAA AGACCAACAG
Dren70_Cf_SF   ATTTAGTGTC CATATATGAC TACCCAAAGC GCATGTGGAA AGACCAACAG
Dren72_Cb_SF   ATTTAGTGTC CATATATGAC TACCCAAAGC GCATGTGGAA AGACCAACAG
Dren73_Cb_Ch   ATTTAGTGTC CACATATGAC TACCCAAAGC GCATGTGGAA AGACCAACAG
Dren74_Cb_Co   ATTTAGTGTC CATATATGAC TACCCAAAGC GCATGTGGAA AGACCAACAG
Dren75_Cb_Co   ATTTAGTGTC CATATATGAC TACCCAAAGC GCATGTGGAA AGACCAACAG
Dren80_Cf_SF   ATTTAGTGTC CATATATGAC TACCCAAAGC GCATGTGGAA AGACCAACAG
Dren81_Cf_SF   ATTTAGTGTC CATATATGAC TACCCAAAGC GCATGTGGAA AGACCAACAG
Dren82_Cb_Co   ATTTAGTGTC CATATATGAC TACCCAAAGC GCATGTGGAA AGACCAACAG
Dren83_Cb_Ch   ATTTAGTGTC CATATATGAC TACCCAAAGC GCATGTGGAA AGACCAACAG
Dren84_Cf_SF   ATTTAGTGTC CATATATGAC TACCCAAAGC GCATGTGGAA AGACCAACAG
Dren85_Cb_SF   ATTTAGTGTT CATATATGAC TACCCAAAGC GCATGTGGAA AGACCAACAG
Dren67_Cf_SF   ATTTAGTGTC CACATATGAC TACCCAAAGC GCATGTGGAA AGACCAACAG
Dren69_Cf_SF   ATTTAGTGTC CACATATGAC TACCCAAAGC GCATGTGGAA AGACCAACAG
Dren86_Cb_Co   ATTTAGTGTC CATATATGAC TACCCAAAGC GCATGTGGAA AGACCAACAG
Dren87_Cf_BA   ATTTAGTGTC CATATATGAC TACCCAAAGC GCATGTGGAA AGACCAACAG
Dren88_Lg_SF   ATTTAGTGTC CACATATGAC TACCCAAAGC GCATGTGGAA AGACCAACAG

               ....|....| ....|....| ....|....| ....|....| ....|....|
                   205        215        225        235        245
Dren02_Cf_BA   TAGGAAGTAT GATCCTGGCC GGGGCGCTAC TAAAAACAGG AGGGTATGGG
Dren07_Cf_BA   TAGGAAGTAT GATCCTGGCC GGGGCGCTAC TAAAAACAGG AGGGTATGGG
Dren08_Cf_BA   TAGGAAGTAT GATCCTGGCC GGGGCGCTAC TAAAAACAGG AGGGTATGGG
Dren09_Cf_BA   TAGGAAGTAT GATCCTGGCC GGGGCGCTAC TAAAAACAGG AGGGTATGGG
Dren10_Cf_BA   TAGGAAGTAT GATCCTGGCC GGGGCGCTAC TAAAAACAGG AGGGTATGGG
Dren18_Cf_BA   TAGGAAGTAT GATCCTGGCC GGGGCGCTAC TAAAAACAGG AGGGTATGGG
Dren19_Cf_BA   TAGGAAGTAT GATCCTGGCC GGGGCGCTAC TAAAAACAGG AGGGTATGGG
Dren23_Cf_BA   TAGGAAGTAT GATCCTGGCC GGGGCGCTAC TAAAAACAGG AGGGTATGGG
```

```
Dren25_Cf_SF   TAGGAAGTAT GATCCTGGCC GGGGCGCTAC TAAAAACAGG AGGGTATGGG
Dren32_Cf_SC   TAGGAAGTAT GATCCTGGCC GGGGCGCTAC TAAAAACAGG AGGGTATGGG
Dren35_Cf_Pa   TAGGAAGTAT GATCCTGGCC GGGGCGCTAC TAAAAACAGG AGGGTATGGG
Dren40_Cf_RG   TAGGAAGTAT GATCCTGGCC GGGGCTTTAC TAAAAACAGG GGGTTATGGG
Dren41_Cf_RG   TAGGAAGTAT GATCCTGGCC GGGGCTTTAC TAAAAACAGG GGGTTATGGG
Dren42_Cf_RG   TAGGAAGTAT GATCCTGGCC GGGGTGCTAC TAAAAACAGG AGGGTATGGG
Dren43_Cf_RG   TAGGAAGTAT GATCCTGGCC GGGGCGCTAC TAAAAACAGG AGGGTATGGG
Dren45_Cf_RG   TAGGAAGTAT GATCCTGGCC GGGGCGCTAC TAAAAACAGG AGGGTATGGG
Dren46_Cf_SF   TAGGAAGTAT GATCCTGGCC GGGGCGCTAC TAAAAACAGG AGGGTATGGG
Dren47_Cb_SF   TAGGAAGTAT GATCCTGGCC GGGGCGCTAC TAAAAACAGG AGGGTATGGG
Dren48_Cf_BA   TAGGAAGTAT GATCCTGGCC GGGGCGCTAC TAAAAACAGG AGGGTATGGG
Dren49_Cf_BA   TAGGAAGTAT GATCCTGGCC GGGGCGCTAC TAAAAACAGG AGGGTATGGG
Dren50_Cf_Ch   TAGGAAGTAT GATCCTGGCC GGGGCGCTAC TAAAAACAGG AGGGTATGGG
Dren53_Cf_BA   TAGGAAGTAT GATCCTGGCC GGGGCGCTAC TAAAAACAGG AGGGTATGGG
Dren58_Cb_SF   TAGGAAGTAT GATCCTGGCC GGGGCTTTAC TAAAAACAGG GGGTTATGGG
Dren66_Cf_SF   TAGGAAGTAT GATCCTGGCC GGGGCGCTAC TAAAAACAGG AGGGTATGGG
Dren68_Cf_SF   TAGGAAGTAT GATCCTGGCC GGGGCGCTAC TAAAAACAGG AGGGTATGGG
Dren70_Cf_SF   TAGGAAGTAT GATCCTGGCC GGGGCGCTAC TAAAAACAGG AGGGTATGGG
Dren72_Cb_SF   TAGGAAGTAT GATCCTGGCC GGGGCGCTAC TAAAAACAGG AGGGTATGGG
Dren73_Cb_Ch   TAGGAAGTAT GATCCTGGCC GGGGCGCTAC TAAAAACAGG AGGGTATGGG
Dren74_Cb_Co   TAGGAAGTAT GATCCTGGCC GGGGCGCTAC TAAAAACAGG AGGGTATGGG
Dren75_Cb_Co   TAGGAAGTAT GATCCTGGCC GGGGCGCTAC TAAAAACAGG AGGGTATGGG
Dren80_Cf_SF   TAGGAAGTAT GATCCTGGCC GGGGCGCTAC TAAAAACAGG AGGGTATGGG
Dren81_Cf_SF   TAGGAAGTAT GATCCTGGCC GGGGCGCTAC TAAAAACAGG AGGGTATGGG
Dren82_Cb_Co   TAGGAAGTAT GATCCTGGCC GGGGCGCTAC TAAAAACAGG AGGGTATGGG
Dren83_Cb_Ch   TAGGAAGTAT GATCCTGGCC GGGGCGCTAC TAAAAACAGG AGGGTATGGG
Dren84_Cf_SF   TAGGAAGTAT GATCCTGGCC GGGGCGCTAC TAAAAACAGG AGGGTATGGG
Dren85_Cb_SF   TAGGAAGTAT GATCCTGGCC GGGGCGCTAC TAAAAACAGG AGGGTATGGG
Dren67_Cf_SF   TAGGAAGTAT GATCCTGGCC GGGGCGCTAC TAAAAACAGG AGGGTATGGG
Dren69_Cf_SF   TAGGAAGTAT GATCCTGGCC GGGGCGCTAC TAAAAACAGG AGGGTATGGG
Dren86_Cb_Co   TAGGAAGTAT GATCCTGGCC GGGGCGCTAC TAAAAACAGG AGGGTATGGG
Dren87_Cf_BA   TAGGAAGTAT GATCCTGGCC GGGGCGCTAC TAAAAACAGG AGGGTATGGG
Dren88_Lg_SF   TAGGAAGTAT GATCCTGGCC GGGGCGCTAC TAAAAACAGG AGGGTATGGG

               ....|....| ....|....| ....|....| ....|....| ....|....|
                   255        265        275        285        295
Dren02_Cf_BA   CTATATAAAA TAAGCCTGTT CATTAGAAGA AGGCTAGCGT GTCTAATAAG
Dren07_Cf_BA   CTATATAAAA TAAGCCTGTT CATTAGAAGA AGGCTAGCGT GTCTAATAAG
Dren08_Cf_BA   CTATATAAAA TAAGCCTGTT CATTAGAAGA AGGCTAGCGT GTCTAGTAAG
Dren09_Cf_BA   CTATATAAAA TAAGCCTGTT CATTAGAAGA AGGCTAGCGT GTCTAATAAG
Dren10_Cf_BA   CTATATAAAA TAAGCCTGTT CATTAGAAGA AGGCTAGCGT GTCTAATAAG
Dren18_Cf_BA   CTATATAAAA TAAGCCTGTT CATTAGAAGA AGGCTAGCGT GTCTAATAAG
Dren19_Cf_BA   CTATATAAAA TAAGCCTGTT CATTAGAAGA AGGCTAGCGT GTCTAATAAG
Dren23_Cf_BA   CTATATAAAA TAAGCCTGTT CATTAGAAGA AGGCTAGCGT GTCTAATAAG
Dren25_Cf_SF   CTATATAAAA TAAGCCTGTT CATTAGAAGA AGGCTAGCGT GTCTAATAAG
Dren32_Cf_SC   CTATATAAAA TAAGCCTGTT CATTAGAAGA AGGCTAGCGT GTCTAATAAG
Dren35_Cf_Pa   CTATATAAAA TAAGCCTGTT CATTAGAAGA AGGCTAGCGT GTCTAATAAG
Dren40_Cf_RG   CTATATAAAA TAAGCCTGTT CATTAGAAGA AGGCTAGCGT GTCTAATAAG
Dren41_Cf_RG   CTATATAAAA TAAGCCTGTT CATTAGAAGA AGGCTAGCGT GTCTAATAAG
Dren42_Cf_RG   CTATATAAAA TAAGCCTGTT CATTAGAAGA AGGCTAGCGT GTCTAATAAG
Dren43_Cf_RG   CTATATAAAA TAAGCCTGTT CATTAGAAGA AGGCTAGCGT GTCTAATAAG
Dren45_Cf_RG   CTATATAAAA TAAGCCTGTT CATTAGAAGA AGGCTAGCGT GTCTAATAAG
Dren46_Cf_SF   CTATATAAAA TAAGCCTGTT CATTAGAAGA AGGCTAGCGT GTCTAATAAG
Dren47_Cb_SF   CTATATAAAA TAAGCCTGTT CATTAGAAGA AGGCTAGCGT GTCTAATAAG
Dren48_Cf_BA   CTATATAAAA TAAGCCTGTT CATTAGAAGA AGGCTAGCGT GTCTAATAAG
Dren49_Cf_BA   CTATATAAAA TAAGCCTGTT CATTAGAAGA AGGCTAGCGT GTCTAATAAG
Dren50_Cf_Ch   CTATATAAAA TAAGCCTGTT CATTAGAAGA AGGCTAGCGT GTCTAATAAG
Dren53_Cf_BA   CTATATAAAA TAAGCCTGTT CATTAGAAGA AGGCTAGCGT GTCTAATAAG
Dren58_Cb_SF   CTATATAAAA TAAGCCTGTT CATTAGAAGA AGGCTAGCGT GTCTAATAAG
Dren66_Cf_SF   CTATATAAAA TAAGCCTGTT CATTAGAAGA AGGCTAGCGT GTCTAATAAG
Dren68_Cf_SF   CTATATAAAA TAAGCCTGTT CATTAGAAGA AGGCTAGCGT GTCTAATAAG
Dren70_Cf_SF   CTATATAAAA TAAGCCTGTT CATTAGAAGA AGGCTAGCGT GTCTAATAAG
Dren72_Cb_SF   CTATATAAAA TAAGCCTGTT CATTAGAAGA AGGCTAGCGT GTCTAATAAG
Dren73_Cb_Ch   CTATATAAAA TAAGCCTGTT CATTAGAAGA AGGCTAGCGT GTCTAATAAG
```

```
Dren74_Cb_Co   CTATATAAAA TAAGCCTGTT CATTAGAAGA AGGCTAGCGT GTCTAATAAG
Dren75_Cb_Co   CTATATAAAA TAAGCCTGTT CATTAGAAGA AGGCTAGCGT GTCTAATAAG
Dren80_Cf_SF   CTATATAAAA TAAGCCTGTT CATTAGAAGA AGGCTAGCGT GTCTAATAAG
Dren81_Cf_SF   CTATATAAAA TAAGCCTGTT CATTAGAAGA AGGCTAGCGT GTCTAATAAG
Dren82_Cb_Co   CTATATAAAA TAAGCCTGTT CATTAGAAGA AGGCTAGCGT GTCTAATAAG
Dren83_Cb_Ch   CTATATAAAA TAAGCCTGTT CATTAGAAGA AGGCTAGCGT GTCTAGTAAG
Dren84_Cf_SF   CTATATAAAA TAAGCCTGTT CATTAGAAGA AGGCTAGCGT GTCTAATAAG
Dren85_Cb_SF   CTATATAAAA TAAGCCTGTT CATTAGAAGA AGGCTAGCGT GTCTAATAAG
Dren67_Cf_SF   CTATATAAAA TAAGCCTGTT CATTAGAAGA AGGCTAGCGT GTCTAATAAG
Dren69_Cf_SF   CTATATAAAA TAAGCCTGTT CATTAGAAGA AGGCTAGCGT GTCTAATAAG
Dren86_Cb_Co   CTATATAAAA TAAGCCTGTT CATTAGAAGA AGGCTAGCGT GTCTAATAAG
Dren87_Cf_BA   CTATATAAAA TAAGCCTGTT CATTAGAAGA AGGCTAGCGT GTCTAATAAG
Dren88_Lg_SF   CTATATAAAA TAAGCCTGTT CATTAGAAGA AGGCTAGCGT GTCTAATAAG

               ....|....| ....|....| ....|....| ....|....| ....|....|
                   305        315        325        335        345
Dren02_Cf_BA   AAGAGTAGGA ATCCTGTTAG TAAGAAGATC AATTCTAACA GGGGTGATAC
Dren07_Cf_BA   AAGAGTAGGA ATCCTGTTAG TAAGAAGATC AATTCTAACA GGGGTGATAC
Dren08_Cf_BA   AAGAGTAGGA ATCCTGTTAG TAAGAAGATC AATTCTAACA GGGGTGATAC
Dren09_Cf_BA   AAGAGTAGGA ATCCTGTTAG TAAGAAGATC AATTCTAACA GGGGTGATAC
Dren10_Cf_BA   AAGAGTAGGA ATCCTGTTAG TAAGAAGATC AATTCTGACA GGGGTGATAC
Dren18_Cf_BA   AAGGGTAGGA ATCCTGTTAG TAAGAAGATC AATTCTAACA GGGGTGATAC
Dren19_Cf_BA   AAGGGTAGGA ATCCTGTTAG TAAGAAGATC AATTCTAACA GGGGTGATAC
Dren23_Cf_BA   AAGAGTAGGA ATCCTGTTAG TAAGAAGATC AATTCTAACA GGGGTGATAC
Dren25_Cf_SF   AAGAGTAGGA ATCCTGTTAG TAAGAAGATC AATTCTAACA GGGGTGATAC
Dren32_Cf_SC   AAGAGTAGGA ATCCTGTTAG TAAGAAGATC AATTCTAACA GGGGTGATAC
Dren35_Cf_Pa   AAGAGTAGGA ATCCTGTTAG TAAGAAGATC AATTCTAACA GGGGTGATAC
Dren40_Cf_RG   AAGAGTAGGA GTCCTGTTAG TAAGAAGATC AATTCTAACA GGGGTGATAC
Dren41_Cf_RG   AAGAGTAGGA GTCCTGTTAG TAAGAAGATC AATTCTAACA GGGGTGATAC
Dren42_Cf_RG   AAGAGTAGGA ATCCTGTTAG TAAGAAGATC AATTCTGACA GGGGTGATAC
Dren43_Cf_RG   AAGAGTAGGA ATCCTGTTAG TAAGAAGATC AATTCTAACA GGGGTGATAC
Dren45_Cf_RG   AAGAGTAGGA ATCCTGTTAG TAAGAAGATC AATTCTAACA GGGGTGATAC
Dren46_Cf_SF   AAGAGTAGGA ATCCTGTTAG TAAGAAGATC AATTCTAACA GGGGTGATAC
Dren47_Cb_SF   AAGAGTAGGA ATCCTGTTAG TAAGAAGATC AATTCTGACA GGGGTGATAC
Dren48_Cf_BA   AAGAGTAGGA ATCCTGTTAG TAAGAAGATC AATTCTAACA GGGGTGATAC
Dren49_Cf_BA   AAGAGTAGGA ATCCTGTTAG TAAGAAGATC AATTCTAACA GGGGTGATAC
Dren50_Cf_Ch   AAGAGTAGGA ATCCTGTTAG TAAGAAGATC AATTCTAACA GGGGTGATAC
Dren53_Cf_BA   AAGAGTAGGA ATCCTGTTAG TAAGAAGATC AATTCTAACA GGGGTGATAC
Dren58_Cb_SF   AAGAGTAGGA GTCCTGTTAG TAAGAAGATC AATTCTAACA GGGGTGATAC
Dren66_Cf_SF   AAGAGTAGGA ATCCTGTTAG TAAGAAGATC AATTCTAACA GGGGTGATAC
Dren68_Cf_SF   AAGAGTAGGA ATCCTGTTAG TAAGAAGATC AATTCTGACA GGGGTGATAC
Dren70_Cf_SF   AAGAGTAGGA ATCCTGTTAG TAAGAAGATC AATTCTAACA GGAGTGATAC
Dren72_Cb_SF   AAGAGTAGGA ATCCTGTTAG TAAGAAGATC AATTCTAACA GGGGTGATAC
Dren73_Cb_Ch   AAGAGTAGGA ATCCTGTTAG TAAGAAGATC AATTCTGACA GGGGTGATAC
Dren74_Cb_Co   AAGAGTAGGA ATCCTGTTAG TAAGAAGATC AATTCTAACA GGGGTGATAC
Dren75_Cb_Co   AAGAGTAGGA ATCCTGTTAG TAAGAAGATC AATTCTAACA GGGGTGATAC
Dren80_Cf_SF   AAGAGTAGGA ATCCTGTTAG TAAGAAGATC AATTCTAACA GGGGTGATAC
Dren81_Cf_SF   AAGAGTAGGA ATCCTGTTAG TAAGAAGATC AATTCTAACA GGGGTGATAC
Dren82_Cb_Co   AAGAGTAGGA ATCCTGTTAG TAAGAAGATC AATTCTAACA GGGGTGATAC
Dren83_Cb_Ch   AAGAGTAGGA ATCCTGTTAG TAAGAAGATC AATTCTAACA GGGGTGATAC
Dren84_Cf_SF   AAGAGTAGGA ATCCTGTTAG TAAGAAGATC AATTCTAACA GGGGTGATAC
Dren85_Cb_SF   AAGAGTAGGA ATCCTGTTAG TAAGAAGATC AATTCTAACA GGGGTGATAC
Dren67_Cf_SF   AAGAGTAGGA ATCCTGTTAG TAAGAAGATC AATTCTGACA GGGGTGATAC
Dren69_Cf_SF   AAGAGTAGGA ATCCTGTTAG TAAGAAGATC AATTCTGACA GGGGTGATAC
Dren86_Cb_Co   AAGAGTAGGA ATCCTGTTAG TAAGAAGATC AATTCTGACA GGGGTGATAC
Dren87_Cf_BA   AAGAGTAGGA ATCCTGTTAG TAAGAAGATC AATTCTGACA GGGGTGATAC
Dren88_Lg_SF   AAGAGTAGGA ATCCTGTTAG TAAGAAGATC AATTCTGACA GGGGTGATAC

               ....

Dren02_Cf_BA   AAAG
Dren07_Cf_BA   AAAG
Dren08_Cf_BA   AAAG
Dren09_Cf_BA   AAAG
```

```
Dren10_Cf_BA   AAAG
Dren18_Cf_BA   AAAG
Dren19_Cf_BA   AAAG
Dren23_Cf_BA   AAAG
Dren25_Cf_SF   AAAG
Dren32_Cf_SC   AAAG
Dren35_Cf_Pa   AAAG
Dren40_Cf_RG   AAAG
Dren41_Cf_RG   AAAG
Dren42_Cf_RG   AAAG
Dren43_Cf_RG   AAAG
Dren45_Cf_RG   AAAG
Dren46_Cf_SF   AAAG
Dren47_Cb_SF   AAAG
Dren48_Cf_BA   AAAG
Dren49_Cf_BA   AAAG
Dren50_Cf_Ch   AAAG
Dren53_Cf_BA   AAAG
Dren58_Cb_SF   AAAG
Dren66_Cf_SF   AAAG
Dren68_Cf_SF   AAAG
Dren70_Cf_SF   AAAG
Dren72_Cb_SF   AAAG
Dren73_Cb_Ch   AAAG
Dren74_Cb_Co   AAAG
Dren75_Cb_Co   AAAG
Dren80_Cf_SF   AAAG
Dren81_Cf_SF   AAAG
Dren82_Cb_Co   AAAG
Dren83_Cb_Ch   AAAG
Dren84_Cf_SF   AAAG
Dren85_Cb_SF   AAAG
Dren67_Cf_SF   AAAG
Dren69_Cf_SF   AAAG
Dren86_Cb_Co   AAAG
Dren87_Cf_BA   AAAG
Dren88_Lg_SF   AAAG
```

Concatenated ND4-COX1-XL Multiple Sequence Alignment

```
                 ....|....| ....|....| ....|....| ....|....| ....|....|
                     5         15         25         35         45
Dren02_Cf_BA     CTTTATACTT TAAGCTTATC AGTCCCGTTA TTTGTACTGC TACTGAACCT
Dren07_Cf_BA     CTTTATACTT TGAGCTTATC AGTCCCGTTA TTTGTACTGC TACTGAACCT
Dren08_Cf_BA     CTTTATACTT TAAGCTTATC AGTCCCGTTA TTTGTACTGC TACTGAACCT
Dren09_Cf_BA     CTTTATACTT TGAGCTTATC AGTCCCGTTA TTTGTACTGC TACTGAACCT
Dren10_Cf_BA     CTTTATACTT TGAGCTTATC AATCCCGTTA TTTGTATTGC TACTGAACCT
Dren18_Cf_BA     CTTTATACTT TGAGCTTATC AGTCCCGTTA TTTGTACTGC TACTGAACCT
Dren19_Cf_BA     CTTTATACTT TGAGCTTATC AGTCCCGTTA TTTGTACTGC TACTGAACCT
Dren23_Cf_BA     CTTTATACTT TGAGCTTATC AGTCCCGTTA TTTGTACTGC TACTGAACCT
Dren25_Cf_SF     CTTTATACTT TGAGCTTATC AGTCCCGTTA TTTGTACTGC TACTGAACCT
Dren32_Cf_SC     CTTTATACTT TGAGCTTATC AGTCCCGTTA TTTGTACTGC TACTGAACCT
Dren35_Cf_Pa     CTTTATACTT TGAGCTTATC AGTCCCGTTA TTTGTACTGC TACTGAACCT
Dren40_Cf_RG     CTTTATACTT TGAGCTTATC AGTTCCGTTA TTTGTTCTGC TACTGAACCT
Dren41_Cf_RG     CTTTATACTT TGAGCTTATC AGTTCCGTTA TTTGTTCTGC TACTGAACCT
Dren42_Cf_RG     CTTTATACTT TGAGCTTATC AGTCCCGTTA TTTGTACTGC TACTGAACCT
Dren43_Cf_RG     CTTTATACTT TGAGCTTATC AGTCCCGTTA TTTGTACTAC TACTGAACCT
Dren45_Cf_RG     CTTTATACTT TGAGCTTATC AGTCCCGTTA TTTGTACTAC TACTGAACCT
Dren46_Cf_SF     CTTTATACTT TGAGCTTATC AGTCCCGTTA TTTGTACTGC TACTGAACCT
Dren47_Cb_SF     CTTTATACTT TGAGCTTATC AGTCCCGTTA TTTGTATTGC TACTGAACCT
Dren48_Cf_BA     CTTTATACTT TAAGCTTATC AGTCCCGTTA TTTGTACTGC TACTGAACCT
Dren49_Cf_BA     CTTTATACTT TGAGCTTATC AGTCCCGTTA TTTGTACTGC TACTGAACCT
Dren50_Cf_Ch     CTTTATACTT TAAGCTTATC AGTCCCGTTA TTTGTACTGC TACTGAACCT
Dren53_Cf_BA     CTTTATACTT TAAGCTTATC AGTCCCGTTA TTTGTACTGC TACTGAACCT
Dren58_Cb_SF     CTTTATACTT TGAGCTTATC AGTTCCGTTA TTTGTTCTGC TACTGAACCT
Dren66_Cf_SF     CTTTATACTT TGAGCTTATC AGTCCCGTTA TTTGTACTGC TACTGAACCT
Dren67_Cf_SF     CTTTATACTT TGAGCTTATC AGTCCCGTTA TTTGTATTGC TACTGAACCT
Dren68_Cf_SF     CTTTATACTT TGAGCTTATC AGTCCCGTTA TTTGTATTGC TACTGAACCT
Dren69_Cf_SF     CTTTATACTT TGAGCTTATC AGTCCCGTTA TTTGTATTGC TACTGAACCT
Dren70_Cf_SF     CTTTATACTT TGAGCTTATC AGTCCCGTTA TTTGTACTGC TACTGAACCT
Dren72_Cb_SF     CTTTATACTT TAAGCTTATC AGTCCCGTTA TTTGTACTGC TACTGAACCT
Dren73_Cb_Ch     CTTTATACTT TGAGCTTATC AGTCCCGTTA TTTGTATTGC TACTGAACCT
Dren74_Cb_Co     CTTTATACTT TGAGCTTATC AGTCCCGTTA TTTGTACTGC TACTGAACCT
Dren75_Cb_Co     CTTTATACTT TAAGCTTATC AGTCCCGTTA TTTGTACTGC TACTGAACCT
Dren80_Cf_SF     CTTTATACTT TGAGCTTATC AGTCCCGTTA TTTGTACTGC TACTGAACCT
Dren81_Cf_SF     CTTTATACTT TGAGCTTATC AGTCCCGTTA TTTGTACTGC TACTGAACCT
Dren82_Cb_Co     CTTTATACTT TGAGCTTATC AGTCCCGTTA TTTGTACTGC TACTGAACCT
Dren83_Cb_Ch     CTTTATACTT TAAGCTTATC AGTCCCGTTA TTTGTACTGC TACTGAACCT
Dren84_Cf_SF     CTTTATACTT TGAGCTTATC AGTCCCGTTA TTTGTACTGC TACTGAACCT
Dren85_Cb_SF     CTTTATACTT TGAGCTTATC AGTCCCGTTA TTTGTACTGC TACTGAACCT
Dren86_Cb_Co     CTTTATACTT TGAGCTTATC AGTCCCGTTA TTTGTACTGC TACTGAACCT
Dren87_Cf_BA     CTTTATACTT TGAGCTTATC AGTCCCGTTA TTTGTACTGC TACTGAACCT
Dren88_Lg_SF     CTTTATACTT TGAGCTTATC AGTCCCGTTA TTTGTATTGC TACTGAACCT

                 ....|....| ....|....| ....|....| ....|....| ....|....|
                    55         65         75         85         95
Dren02_Cf_BA     AAATAATTTT AGCTGTCAAA ACAGCTGACC ATCAAACCGC AATCTTTCAT
Dren07_Cf_BA     AAATAATTTT AGCTGTCAAA ACAGCTGACC ATCAAACCGC AATCTTTCAT
Dren08_Cf_BA     AAATAATTTT AGCTGTCAAA ACAGCTGACC ATCAAACCGC AATCTTTCAT
Dren09_Cf_BA     AAATAATTTT AGCTGTCAAA ACAGCTGACC ATCAAACCGC AATCTTTCAT
```

```
Dren10_Cf_BA   AAATAATTTT AGCTGTCAAA ACAGCTGACC ATCAAACCGC AATCTTTCAT
Dren18_Cf_BA   AAATAATTTT AGCTGTCAAA ACAGCTGACC ATCAAACCGC AATCTTTCAT
Dren19_Cf_BA   AAATAATTTT AGCTGTCAAA ACAGCTGACC ATCAAACCGC AATCTTTCAT
Dren23_Cf_BA   AAATAATTTT AGCTGTCAAA ACAGCTGACC ATCAAACCGC AATCTTTCAT
Dren25_Cf_SF   AAATAATTTT AGCTGTCAAA ACAGCTGACC ATCAAACCGC AATCTTTCAT
Dren32_Cf_SC   AAATAATTTT AGCTGTCAAA ACAGCTGACC ATCAAACCGC AATCTTTCAT
Dren35_Cf_Pa   AAATAATTTT AGCTGTCAAA ACAGCTGACC ATCAAACCGC AATCTTTCAT
Dren40_Cf_RG   AAATAATTTT AGCTGTCAAA ACAGCTGACC ATCAAACCGC AATCTTTCAT
Dren41_Cf_RG   AAATAATTTT AGCTGTCAAA ACAGCTGACC ATCAAACCGC AATCTTTCAT
Dren42_Cf_RG   AAATAATTTT AGCTGTCAAA ACAGCTGACC ATCAAACCGC AATCTTTCAT
Dren43_Cf_RG   AAATAATTTT AGCTGTCAAA ACAGCTGACC ATCAAACCGC AATCTTTCAT
Dren45_Cf_RG   AAATAATTTT AGCTGTCAAA ACAGCTGACC ATCAAACCGC AATCTTTCAT
Dren46_Cf_SF   AAATAATTTT AGCTGTCAAA ACAGCTGACC ATCAAACCGC AATCTTTCAT
Dren47_Cb_SF   AAATAATTTT AGCTGTCAAA ACAGCTGACC ATCAAACCGC AATCTTTCAT
Dren48_Cf_BA   AAATAATTTT AGCTGTCAAA ACAGCTGACC ATCAAACCGC AATCTTTCAT
Dren49_Cf_BA   AAATAATTTT AGCTGTCAAA ACAGCTGACC ATCAAACCGC AATCTTTCAT
Dren50_Cf_Ch   AAATAATTTT AGCTGTCAAA ACAGCTGACC ATCAAACCGC AATCTTTCAT
Dren53_Cf_BA   AAATAATTTT AGCTGTCAAA ACAGCTGACC ATCAAACCGC AATCTTTCAT
Dren58_Cb_SF   AAATAATTTT AGCTGTCAAA ACAGCTGACC ATCAAACCGC AATCTTTCAT
Dren66_Cf_SF   AAATAATTTT AGCTGTCAAA ACAGCTGACC ATCAAACCGC AATCTTTCAT
Dren67_Cf_SF   AAATAATTTT AGCTGTCAAA ACAGCTGACC ATCAAACCGC AATCTTTCAT
Dren68_Cf_SF   AAATAATTTT AGCTGTCAAA ACAGCTGACC ATCAAACCGC AATCTTTCAT
Dren69_Cf_SF   AAATAATTTT AGCTGTCAAA ACAGCTGACC ATCAAACCGC AATCTTTCAT
Dren70_Cf_SF   AAATAATTTT AGCTGTCAAA ACAGCTGACC ATCAAACCGC AATCTTTCAT
Dren72_Cb_SF   AAATAATTTT AGCTGTCAAA ACAGCTGACC ATCAAACCGC AATCTTTCAT
Dren73_Cb_Ch   AAATAATTTT AGCTGTCAAA ACAGCTGACC ATCAAACCGC AATCTTTCAT
Dren74_Cb_Co   AAATAATTTT AGCTGTCAAA ACAGCTGACC ATCAAACCGC AATCTTTCAT
Dren75_Cb_Co   AAATAATTTT AGCTGTCAAA ACAGCTGACC ATCAAACCGC AATCTTTCAT
Dren80_Cf_SF   AAATAATTTT AGCTGTCAAA ACAGCTGACC ATCAAACCGC AATCTTTCAT
Dren81_Cf_SF   AAATAATTTT AGCTGTCAAA ACAGCTGACC ATCAAACCGC AATCTTTCAT
Dren82_Cb_Co   AAATAATTTT AGCTGTCAAA ACAGCTGACC ATCAAACCGC AATCTTTCAT
Dren83_Cb_Ch   AAATAATTTT AGCTGTCAAA ACAGCTGACC ATCAAACCGC AATCTTTCAT
Dren84_Cf_SF   AAATAATTTT AGCTGTCAAA ACAGCTGACC ATCAAACCGC AATCTTTCAT
Dren85_Cb_SF   AAATAATTTT AGCTGTCAAA ACAGCTGACC ATCAAACCGC AATCTTTCAT
Dren86_Cb_Co   AAATAATTTT AGCTGTCAAA ACAGCTGACC ATCAAACCGC AATCTTTCAT
Dren87_Cf_BA   AAATAATTTT AGCTGTCAAA ACAGCTGACC ATCAAACCGC AATCTTTCAT
Dren88_Lg_SF   AAATAATTTT AGCTGTCAAA ACAGCTGACC ATCAAACCGC AATCTTTCAT

               ....|....| ....|....| ....|....| ....|....| ....|....|
                   105        115        125        135        145
Dren02_Cf_BA   TTATTATGAT AACCATAATC ACCATACCCT TTTTAATTAA GATGCCACTA
Dren07_Cf_BA   TTATTATGAT AACCATAATC ACCATACCCT TTTTAATTAA GATGCCACTA
Dren08_Cf_BA   TTATTATGAT AACCATAATC ACCATACCCT TTTTAATTAA GATGCCACTA
Dren09_Cf_BA   TTATTATGAT AACCATAATC ACCATACCCT TTTTAATTAA GATGCCACTA
Dren10_Cf_BA   TTATTATGAT AATCATAATC ACCATACCCT TTTTAATTAA GATGCCACTA
Dren18_Cf_BA   TTATTATGAT AACCATAATC ACCATGCCCT TTTTAATTAA GATGCCACTA
Dren19_Cf_BA   TTATTATGAT AACCATAATC ACCATGCCCT TTTTAATTAA GATGCCACTA
Dren23_Cf_BA   TTATTATGAT AACCATAATC ACCATACCCT TTTTAATTAA GATGCCACTA
Dren25_Cf_SF   TTATTATGAT AACCATAATC ACCATACCCT TTTTAATTAA GATGCCACTA
Dren32_Cf_SC   TTATTATGAT AACCATAATC ACCATACCCT TTTTAATTAA GATGCCACTA
Dren35_Cf_Pa   TTATTATGAT AACCATAATC ACCATACCCT TTTTAATTAA GATGCCACTA
Dren40_Cf_RG   TTATCATGAT AACCATAATC ACCATACCCT TTCTAATTAA GATGCCACTA
Dren41_Cf_RG   TTATCATGAT AACCATAATC ACCATACCCT TTCTAATTAA GATGCCACTA
Dren42_Cf_RG   TTATTATGAC AATCATAATC ACCATACCCT TTTTAATTAA GATGCCACTA
```

```
Dren43_Cf_RG   TTATTATGAT AACCATAATC ACCATACCCT TTTTAATTAA GATGCCACTA
Dren45_Cf_RG   TTATTATGAT AACCATAATC ACCATACCCT TTTTAATTAA GATGCCACTA
Dren46_Cf_SF   TTATTATGAT AACCATAATC ACCATACCCT TTTTAATTAA GATGCCACTA
Dren47_Cb_SF   TTATTATGAT AATCATAATC ACCATACCCT TTTTAATTAA GATGCCACTA
Dren48_Cf_BA   TTATTATGAT AACCATAATC ACCATACCCT TTTTAATTAA GATGCCACTA
Dren49_Cf_BA   TTATTATGAT AACCATAATC ACCATACCCT TTTTAATTAA GATGCCACTA
Dren50_Cf_Ch   TTATTATGAT AACCATAATC ACCATACCCT TTTTAATTAA GATGCCACTA
Dren53_Cf_BA   TTATTATGAT AACCATAATC ACCATACCCT TTTTAATTAA GATGCCACTA
Dren58_Cb_SF   TTATCATGAT AACCATAATC ACCATACCCT TTCTAATTAA GATGCCACTA
Dren66_Cf_SF   TTATTATGAT AACCATAATC ACCATGCCCT TTTTAATTAA GATGCCACTA
Dren67_Cf_SF   TTATTATGAT AATCATAATC ACCATACCCT TTTTAATTAA GATGCCACTA
Dren68_Cf_SF   TTATTATGAT AATCATAATC ACCATACCCT TTTTAATTAA GATGCCACTA
Dren69_Cf_SF   TTATTATGAT AATCATAATC ACCATACCCT TTTTAATTAA GATGCCACTA
Dren70_Cf_SF   TTATTATGAT AACCATAATC ACCATACCCT TTTTAATTAA GATGCCACTA
Dren72_Cb_SF   TTATTATGAT AACCATAATC ACCATACCCT TTTTAATTAA GATGCCACTA
Dren73_Cb_Ch   TTATTATGAT AATCATAATC ACCATACCCT TTTTAATTAA GATGCCACTA
Dren74_Cb_Co   TTATTATGAT AACCATAATC ACCATACCCT TTTTAATTAA GATGCCACTA
Dren75_Cb_Co   TTATTATGAT AACCATAATC ACCATACCCT TTTTAATTAA GATGCCACTA
Dren80_Cf_SF   TTATTATGAT AACCATAATC ACCATACCCT TTTTAATTAA GATGCCACTA
Dren81_Cf_SF   TTATTATGAT AACCATAATC ACCATACCCT TTTTAATTAA GATGCCACTA
Dren82_Cb_Co   TTATTATGAT AACCATAATC ACCATACCCT TTTTAATTAA GATGCCACTA
Dren83_Cb_Ch   TTATTATGAT AACCATAATC ACCATACCCT TTTTAATTAA GATGCCACTA
Dren84_Cf_SF   TTATTATGAT AACCATAATC ACCATACCCT TTTTAATTAA GATGCCACTA
Dren85_Cb_SF   TTATTATGAT AACCATAATC ACCATACCCT TTTTAATTAA GATGCCACTA
Dren86_Cb_Co   TTATTATGAT AATCATAATC ACCATACCCT TTTTAATTAA GATGCCACTA
Dren87_Cf_BA   TTATTATGAT AATCATAATC ACCATACCCT TTTTAATTAA GATGCCACTA
Dren88_Lg_SF   TTATTATGAT AATCATAATC ACCATACCCT TTTTAATTAA GATGCCACTA

               ....|....| ....|....| ....|....| ....|....| ....|....|
                   155        165        175        185        195
Dren02_Cf_BA   TTTAGTGTCC ATATATGACT ACCCAAAGCG CATGTGGAAA GACCAACAGT
Dren07_Cf_BA   TTTAGTGTCC ATATATGACT ACCCAAAGCG CATGTGGAAA GACCAACAGT
Dren08_Cf_BA   TTTAGTGTCC ATATATGACT ACCCAAAGCG CATGTGGAAA GACCAACAGT
Dren09_Cf_BA   TTTAGTGTTC ATATATGACT ACCCAAAGCG CATGTGGAAA GACCAACAGT
Dren10_Cf_BA   TTTAGTGTCC ACATATGACT ACCCAAAGCG CATGTGGAAA GACCAACAGT
Dren18_Cf_BA   TTTAGTGTCC ATATATGACT ACCCAAAGCG CATGTGGAAA GACCAACAGT
Dren19_Cf_BA   TTTAGTGTCC ATATATGACT ACCCAAAGCG CATGTGGAAA GACCAACAGT
Dren23_Cf_BA   TTTAGTGTTC ATATATGACT ACCCAAAGCG CATGTGGAAA GACCAACAGT
Dren25_Cf_SF   TTTAGTGTCC ATATATGACT ACCCAAAGCG CATGTGGAAA GACCAACAGT
Dren32_Cf_SC   TTTAGTGTCC ATATATGACT ACCCAAAGCG CATGTGGAAA GACCAACAGT
Dren35_Cf_Pa   TTTAGTGTCC ATATATGACT ACCCAAAGCG CATGTGGAAA GACCAACAGT
Dren40_Cf_RG   TTTAGTGTCC ATATATGACT ACCCAAAGCG CATGTGGAAA GACCAACAGT
Dren41_Cf_RG   TTTAGTGTCC ATATATGACT ACCCAAAGCG CATGTGGAAA GACCAACAGT
Dren42_Cf_RG   TTTAGTGTCC ATATATGACT ACCCAAAGCG CATGTGGAAA GACCAACAGT
Dren43_Cf_RG   TTTAGTGTCC ATATATGACT ACCCAAAGCG CATGTGGAAA GACCAACAGT
Dren45_Cf_RG   TTTAGTGTCC ATATATGACT ACCCAAAGCG CATGTGGAAA GACCAACAGT
Dren46_Cf_SF   TTTAGTGTCC ATATATGACT ACCCAAAGCG CATGTGGAAA GACCAACAGT
Dren47_Cb_SF   TTTAGTGTCC ACATATGACT ACCCAAAGCG CATGTGGAAA GACCAACAGT
Dren48_Cf_BA   TTTAGTGTCC ATATATGACT ACCCAAAGCG CATGTGGAAA GACCAACAGT
Dren49_Cf_BA   TTTAGTGTTC ATATATGACT ACCCAAAGCG CATGTGGAAA GACCAACAGT
Dren50_Cf_Ch   TTTAGTGTCC ATATATGACT ACCCAAAGCG CATGTGGAAA GACCAACAGT
Dren53_Cf_BA   TTTAGTGTCC ATATATGACT ACCCAAAGCG CATGTGGAAA GACCAACAGT
Dren58_Cb_SF   TTTAGTGTCC ATATATGACT ACCCAAAGCG CATGTGGAAA GACCAACAGT
Dren66_Cf_SF   TTTAGTGTCC ATATATGACT ACCCAAAGCG CATGTGGAAA GACCAACAGT
```

```
Dren67_Cf_SF   TTTAGTGTCC ACATATGACT ACCCAAAGCG CATGTGGAAA GACCAACAGT
Dren68_Cf_SF   TTTAGTGTCC ACATATGACT ACCCAAAGCG CATGTGGAAA GACCAACAGT
Dren69_Cf_SF   TTTAGTGTCC ACATATGACT ACCCAAAGCG CATGTGGAAA GACCAACAGT
Dren70_Cf_SF   TTTAGTGTCC ATATATGACT ACCCAAAGCG CATGTGGAAA GACCAACAGT
Dren72_Cb_SF   TTTAGTGTCC ATATATGACT ACCCAAAGCG CATGTGGAAA GACCAACAGT
Dren73_Cb_Ch   TTTAGTGTCC ACATATGACT ACCCAAAGCG CATGTGGAAA GACCAACAGT
Dren74_Cb_Co   TTTAGTGTCC ATATATGACT ACCCAAAGCG CATGTGGAAA GACCAACAGT
Dren75_Cb_Co   TTTAGTGTCC ATATATGACT ACCCAAAGCG CATGTGGAAA GACCAACAGT
Dren80_Cf_SF   TTTAGTGTCC ATATATGACT ACCCAAAGCG CATGTGGAAA GACCAACAGT
Dren81_Cf_SF   TTTAGTGTCC ATATATGACT ACCCAAAGCG CATGTGGAAA GACCAACAGT
Dren82_Cb_Co   TTTAGTGTCC ATATATGACT ACCCAAAGCG CATGTGGAAA GACCAACAGT
Dren83_Cb_Ch   TTTAGTGTCC ATATATGACT ACCCAAAGCG CATGTGGAAA GACCAACAGT
Dren84_Cf_SF   TTTAGTGTCC ATATATGACT ACCCAAAGCG CATGTGGAAA GACCAACAGT
Dren85_Cb_SF   TTTAGTGTTC ATATATGACT ACCCAAAGCG CATGTGGAAA GACCAACAGT
Dren86_Cb_Co   TTTAGTGTCC ATATATGACT ACCCAAAGCG CATGTGGAAA GACCAACAGT
Dren87_Cf_BA   TTTAGTGTCC ATATATGACT ACCCAAAGCG CATGTGGAAA GACCAACAGT
Dren88_Lg_SF   TTTAGTGTCC ACATATGACT ACCCAAAGCG CATGTGGAAA GACCAACAGT

               ....|....| ....|....| ....|....| ....|....| ....|....|
                   205        215        225        235        245
Dren02_Cf_BA   AGGAAGTATG ATCCTGGCCG GGGCGCTACT AAAAACAGGA GGGTATGGGC
Dren07_Cf_BA   AGGAAGTATG ATCCTGGCCG GGGCGCTACT AAAAACAGGA GGGTATGGGC
Dren08_Cf_BA   AGGAAGTATG ATCCTGGCCG GGGCGCTACT AAAAACAGGA GGGTATGGGC
Dren09_Cf_BA   AGGAAGTATG ATCCTGGCCG GGGCGCTACT AAAAACAGGA GGGTATGGGC
Dren10_Cf_BA   AGGAAGTATG ATCCTGGCCG GGGCGCTACT AAAAACAGGA GGGTATGGGC
Dren18_Cf_BA   AGGAAGTATG ATCCTGGCCG GGGCGCTACT AAAAACAGGA GGGTATGGGC
Dren19_Cf_BA   AGGAAGTATG ATCCTGGCCG GGGCGCTACT AAAAACAGGA GGGTATGGGC
Dren23_Cf_BA   AGGAAGTATG ATCCTGGCCG GGGCGCTACT AAAAACAGGA GGGTATGGGC
Dren25_Cf_SF   AGGAAGTATG ATCCTGGCCG GGGCGCTACT AAAAACAGGA GGGTATGGGC
Dren32_Cf_SC   AGGAAGTATG ATCCTGGCCG GGGCGCTACT AAAAACAGGA GGGTATGGGC
Dren35_Cf_Pa   AGGAAGTATG ATCCTGGCCG GGGCGCTACT AAAAACAGGA GGGTATGGGC
Dren40_Cf_RG   AGGAAGTATG ATCCTGGCCG GGGCTTTACT AAAAACAGGG GGTTATGGGC
Dren41_Cf_RG   AGGAAGTATG ATCCTGGCCG GGGCTTTACT AAAAACAGGG GGTTATGGGC
Dren42_Cf_RG   AGGAAGTATG ATCCTGGCCG GGGTGCTACT AAAAACAGGA GGGTATGGGC
Dren43_Cf_RG   AGGAAGTATG ATCCTGGCCG GGGCGCTACT AAAAACAGGA GGGTATGGGC
Dren45_Cf_RG   AGGAAGTATG ATCCTGGCCG GGGCGCTACT AAAAACAGGA GGGTATGGGC
Dren46_Cf_SF   AGGAAGTATG ATCCTGGCCG GGGCGCTACT AAAAACAGGA GGGTATGGGC
Dren47_Cb_SF   AGGAAGTATG ATCCTGGCCG GGGCGCTACT AAAAACAGGA GGGTATGGGC
Dren48_Cf_BA   AGGAAGTATG ATCCTGGCCG GGGCGCTACT AAAAACAGGA GGGTATGGGC
Dren49_Cf_BA   AGGAAGTATG ATCCTGGCCG GGGCGCTACT AAAAACAGGA GGGTATGGGC
Dren50_Cf_Ch   AGGAAGTATG ATCCTGGCCG GGGCGCTACT AAAAACAGGA GGGTATGGGC
Dren53_Cf_BA   AGGAAGTATG ATCCTGGCCG GGGCGCTACT AAAAACAGGA GGGTATGGGC
Dren58_Cb_SF   AGGAAGTATG ATCCTGGCCG GGGCTTTACT AAAAACAGGG GGTTATGGGC
Dren66_Cf_SF   AGGAAGTATG ATCCTGGCCG GGGCGCTACT AAAAACAGGA GGGTATGGGC
Dren67_Cf_SF   AGGAAGTATG ATCCTGGCCG GGGCGCTACT AAAAACAGGA GGGTATGGGC
Dren68_Cf_SF   AGGAAGTATG ATCCTGGCCG GGGCGCTACT AAAAACAGGA GGGTATGGGC
Dren69_Cf_SF   AGGAAGTATG ATCCTGGCCG GGGCGCTACT AAAAACAGGA GGGTATGGGC
Dren70_Cf_SF   AGGAAGTATG ATCCTGGCCG GGGCGCTACT AAAAACAGGA GGGTATGGGC
Dren72_Cb_SF   AGGAAGTATG ATCCTGGCCG GGGCGCTACT AAAAACAGGA GGGTATGGGC
Dren73_Cb_Ch   AGGAAGTATG ATCCTGGCCG GGGCGCTACT AAAAACAGGA GGGTATGGGC
Dren74_Cb_Co   AGGAAGTATG ATCCTGGCCG GGGCGCTACT AAAAACAGGA GGGTATGGGC
Dren75_Cb_Co   AGGAAGTATG ATCCTGGCCG GGGCGCTACT AAAAACAGGA GGGTATGGGC
Dren80_Cf_SF   AGGAAGTATG ATCCTGGCCG GGGCGCTACT AAAAACAGGA GGGTATGGGC
Dren81_Cf_SF   AGGAAGTATG ATCCTGGCCG GGGCGCTACT AAAAACAGGA GGGTATGGGC
```

```
Dren82_Cb_Co    AGGAAGTATG ATCCTGGCCG GGGCGCTACT AAAAACAGGA GGGTATGGGC
Dren83_Cb_Ch    AGGAAGTATG ATCCTGGCCG GGGCGCTACT AAAAACAGGA GGGTATGGGC
Dren84_Cf_SF    AGGAAGTATG ATCCTGGCCG GGGCGCTACT AAAAACAGGA GGGTATGGGC
Dren85_Cb_SF    AGGAAGTATG ATCCTGGCCG GGGCGCTACT AAAAACAGGA GGGTATGGGC
Dren86_Cb_Co    AGGAAGTATG ATCCTGGCCG GGGCGCTACT AAAAACAGGA GGGTATGGGC
Dren87_Cf_BA    AGGAAGTATG ATCCTGGCCG GGGCGCTACT AAAAACAGGA GGGTATGGGC
Dren88_Lg_SF    AGGAAGTATG ATCCTGGCCG GGGCGCTACT AAAAACAGGA GGGTATGGGC

                ....|....| ....|....| ....|....| ....|....| ....|....|
                     255        265        275        285        295
Dren02_Cf_BA    TATATAAAAT AAGCCTGTTC ATTAGAAGAA GGCTAGCGTG TCTAATAAGA
Dren07_Cf_BA    TATATAAAAT AAGCCTGTTC ATTAGAAGAA GGCTAGCGTG TCTAATAAGA
Dren08_Cf_BA    TATATAAAAT AAGCCTGTTC ATTAGAAGAA GGCTAGCGTG TCTAGTAAGA
Dren09_Cf_BA    TATATAAAAT AAGCCTGTTC ATTAGAAGAA GGCTAGCGTG TCTAATAAGA
Dren10_Cf_BA    TATATAAAAT AAGCCTGTTC ATTAGAAGAA GGCTAGCGTG TCTAATAAGA
Dren18_Cf_BA    TATATAAAAT AAGCCTGTTC ATTAGAAGAA GGCTAGCGTG TCTAATAAGA
Dren19_Cf_BA    TATATAAAAT AAGCCTGTTC ATTAGAAGAA GGCTAGCGTG TCTAATAAGA
Dren23_Cf_BA    TATATAAAAT AAGCCTGTTC ATTAGAAGAA GGCTAGCGTG TCTAATAAGA
Dren25_Cf_SF    TATATAAAAT AAGCCTGTTC ATTAGAAGAA GGCTAGCGTG TCTAATAAGA
Dren32_Cf_SC    TATATAAAAT AAGCCTGTTC ATTAGAAGAA GGCTAGCGTG TCTAATAAGA
Dren35_Cf_Pa    TATATAAAAT AAGCCTGTTC ATTAGAAGAA GGCTAGCGTG TCTAATAAGA
Dren40_Cf_RG    TATATAAAAT AAGCCTGTTC ATTAGAAGAA GGCTAGCGTG TCTAATAAGA
Dren41_Cf_RG    TATATAAAAT AAGCCTGTTC ATTAGAAGAA GGCTAGCGTG TCTAATAAGA
Dren42_Cf_RG    TATATAAAAT AAGCCTGTTC ATTAGAAGAA GGCTAGCGTG TCTAATAAGA
Dren43_Cf_RG    TATATAAAAT AAGCCTGTTC ATTAGAAGAA GGCTAGCGTG TCTAATAAGA
Dren45_Cf_RG    TATATAAAAT AAGCCTGTTC ATTAGAAGAA GGCTAGCGTG TCTAATAAGA
Dren46_Cf_SF    TATATAAAAT AAGCCTGTTC ATTAGAAGAA GGCTAGCGTG TCTAATAAGA
Dren47_Cb_SF    TATATAAAAT AAGCCTGTTC ATTAGAAGAA GGCTAGCGTG TCTAATAAGA
Dren48_Cf_BA    TATATAAAAT AAGCCTGTTC ATTAGAAGAA GGCTAGCGTG TCTAATAAGA
Dren49_Cf_BA    TATATAAAAT AAGCCTGTTC ATTAGAAGAA GGCTAGCGTG TCTAATAAGA
Dren50_Cf_Ch    TATATAAAAT AAGCCTGTTC ATTAGAAGAA GGCTAGCGTG TCTAATAAGA
Dren53_Cf_BA    TATATAAAAT AAGCCTGTTC ATTAGAAGAA GGCTAGCGTG TCTAATAAGA
Dren58_Cb_SF    TATATAAAAT AAGCCTGTTC ATTAGAAGAA GGCTAGCGTG TCTAATAAGA
Dren66_Cf_SF    TATATAAAAT AAGCCTGTTC ATTAGAAGAA GGCTAGCGTG TCTAATAAGA
Dren67_Cf_SF    TATATAAAAT AAGCCTGTTC ATTAGAAGAA GGCTAGCGTG TCTAATAAGA
Dren68_Cf_SF    TATATAAAAT AAGCCTGTTC ATTAGAAGAA GGCTAGCGTG TCTAATAAGA
Dren69_Cf_SF    TATATAAAAT AAGCCTGTTC ATTAGAAGAA GGCTAGCGTG TCTAATAAGA
Dren70_Cf_SF    TATATAAAAT AAGCCTGTTC ATTAGAAGAA GGCTAGCGTG TCTAATAAGA
Dren72_Cb_SF    TATATAAAAT AAGCCTGTTC ATTAGAAGAA GGCTAGCGTG TCTAATAAGA
Dren73_Cb_Ch    TATATAAAAT AAGCCTGTTC ATTAGAAGAA GGCTAGCGTG TCTAATAAGA
Dren74_Cb_Co    TATATAAAAT AAGCCTGTTC ATTAGAAGAA GGCTAGCGTG TCTAATAAGA
Dren75_Cb_Co    TATATAAAAT AAGCCTGTTC ATTAGAAGAA GGCTAGCGTG TCTAATAAGA
Dren80_Cf_SF    TATATAAAAT AAGCCTGTTC ATTAGAAGAA GGCTAGCGTG TCTAATAAGA
Dren81_Cf_SF    TATATAAAAT AAGCCTGTTC ATTAGAAGAA GGCTAGCGTG TCTAATAAGA
Dren82_Cb_Co    TATATAAAAT AAGCCTGTTC ATTAGAAGAA GGCTAGCGTG TCTAATAAGA
Dren83_Cb_Ch    TATATAAAAT AAGCCTGTTC ATTAGAAGAA GGCTAGCGTG TCTAGTAAGA
Dren84_Cf_SF    TATATAAAAT AAGCCTGTTC ATTAGAAGAA GGCTAGCGTG TCTAATAAGA
Dren85_Cb_SF    TATATAAAAT AAGCCTGTTC ATTAGAAGAA GGCTAGCGTG TCTAATAAGA
Dren86_Cb_Co    TATATAAAAT AAGCCTGTTC ATTAGAAGAA GGCTAGCGTG TCTAATAAGA
Dren87_Cf_BA    TATATAAAAT AAGCCTGTTC ATTAGAAGAA GGCTAGCGTG TCTAATAAGA
Dren88_Lg_SF    TATATAAAAT AAGCCTGTTC ATTAGAAGAA GGCTAGCGTG TCTAATAAGA

                ....|....| ....|....| ....|....| ....|....| ....|....|
                     305        315        325        335        345
```

```
Dren02_Cf_BA   AGAGTAGGAA TCCTGTTAGT AAGAAGATCA ATTCTAACAG GGGTGATACA
Dren07_Cf_BA   AGAGTAGGAA TCCTGTTAGT AAGAAGATCA ATTCTAACAG GGGTGATACA
Dren08_Cf_BA   AGAGTAGGAA TCCTGTTAGT AAGAAGATCA ATTCTAACAG GGGTGATACA
Dren09_Cf_BA   AGAGTAGGAA TCCTGTTAGT AAGAAGATCA ATTCTAACAG GGGTGATACA
Dren10_Cf_BA   AGAGTAGGAA TCCTGTTAGT AAGAAGATCA ATTCTGACAG GGGTGATACA
Dren18_Cf_BA   AGGGTAGGAA TCCTGTTAGT AAGAAGATCA ATTCTAACAG GGGTGATACA
Dren19_Cf_BA   AGGGTAGGAA TCCTGTTAGT AAGAAGATCA ATTCTAACAG GGGTGATACA
Dren23_Cf_BA   AGAGTAGGAA TCCTGTTAGT AAGAAGATCA ATTCTAACAG GGGTGATACA
Dren25_Cf_SF   AGAGTAGGAA TCCTGTTAGT AAGAAGATCA ATTCTAACAG GGGTGATACA
Dren32_Cf_SC   AGAGTAGGAA TCCTGTTAGT AAGAAGATCA ATTCTAACAG GGGTGATACA
Dren35_Cf_Pa   AGAGTAGGAA TCCTGTTAGT AAGAAGATCA ATTCTAACAG GGGTGATACA
Dren40_Cf_RG   AGAGTAGGAG TCCTGTTAGT AAGAAGATCA ATTCTAACAG GGGTGATACA
Dren41_Cf_RG   AGAGTAGGAG TCCTGTTAGT AAGAAGATCA ATTCTAACAG GGGTGATACA
Dren42_Cf_RG   AGAGTAGGAA TCCTGTTAGT AAGAAGATCA ATTCTGACAG GGGTGATACA
Dren43_Cf_RG   AGAGTAGGAA TCCTGTTAGT AAGAAGATCA ATTCTAACAG GGGTGATACA
Dren45_Cf_RG   AGAGTAGGAA TCCTGTTAGT AAGAAGATCA ATTCTAACAG GGGTGATACA
Dren46_Cf_SF   AGAGTAGGAA TCCTGTTAGT AAGAAGATCA ATTCTAACAG GGGTGATACA
Dren47_Cb_SF   AGAGTAGGAA TCCTGTTAGT AAGAAGATCA ATTCTGACAG GGGTGATACA
Dren48_Cf_BA   AGAGTAGGAA TCCTGTTAGT AAGAAGATCA ATTCTAACAG GGGTGATACA
Dren49_Cf_BA   AGAGTAGGAA TCCTGTTAGT AAGAAGATCA ATTCTAACAG GGGTGATACA
Dren50_Cf_Ch   AGAGTAGGAA TCCTGTTAGT AAGAAGATCA ATTCTAACAG GGGTGATACA
Dren53_Cf_BA   AGAGTAGGAA TCCTGTTAGT AAGAAGATCA ATTCTAACAG GGGTGATACA
Dren58_Cb_SF   AGAGTAGGAG TCCTGTTAGT AAGAAGATCA ATTCTAACAG GGGTGATACA
Dren66_Cf_SF   AGAGTAGGAA TCCTGTTAGT AAGAAGATCA ATTCTAACAG GGGTGATACA
Dren67_Cf_SF   AGAGTAGGAA TCCTGTTAGT AAGAAGATCA ATTCTGACAG GGGTGATACA
Dren68_Cf_SF   AGAGTAGGAA TCCTGTTAGT AAGAAGATCA ATTCTGACAG GGGTGATACA
Dren69_Cf_SF   AGAGTAGGAA TCCTGTTAGT AAGAAGATCA ATTCTGACAG GGGTGATACA
Dren70_Cf_SF   AGAGTAGGAA TCCTGTTAGT AAGAAGATCA ATTCTAACAG GAGTGATACA
Dren72_Cb_SF   AGAGTAGGAA TCCTGTTAGT AAGAAGATCA ATTCTAACAG GGGTGATACA
Dren73_Cb_Ch   AGAGTAGGAA TCCTGTTAGT AAGAAGATCA ATTCTGACAG GGGTGATACA
Dren74_Cb_Co   AGAGTAGGAA TCCTGTTAGT AAGAAGATCA ATTCTAACAG GGGTGATACA
Dren75_Cb_Co   AGAGTAGGAA TCCTGTTAGT AAGAAGATCA ATTCTAACAG GGGTGATACA
Dren80_Cf_SF   AGAGTAGGAA TCCTGTTAGT AAGAAGATCA ATTCTAACAG GGGTGATACA
Dren81_Cf_SF   AGAGTAGGAA TCCTGTTAGT AAGAAGATCA ATTCTAACAG GGGTGATACA
Dren82_Cb_Co   AGAGTAGGAA TCCTGTTAGT AAGAAGATCA ATTCTAACAG GGGTGATACA
Dren83_Cb_Ch   AGAGTAGGAA TCCTGTTAGT AAGAAGATCA ATTCTAACAG GGGTGATACA
Dren84_Cf_SF   AGAGTAGGAA TCCTGTTAGT AAGAAGATCA ATTCTAACAG GGGTGATACA
Dren85_Cb_SF   AGAGTAGGAA TCCTGTTAGT AAGAAGATCA ATTCTAACAG GGGTGATACA
Dren86_Cb_Co   AGAGTAGGAA TCCTGTTAGT AAGAAGATCA ATTCTGACAG GGGTGATACA
Dren87_Cf_BA   AGAGTAGGAA TCCTGTTAGT AAGAAGATCA ATTCTGACAG GGGTGATACA
Dren88_Lg_SF   AGAGTAGGAA TCCTGTTAGT AAGAAGATCA ATTCTGACAG GGGTGATACA

               ....|....| ....|....| ....|....| ....|....| ....|....|
                    355        365        375        385        395
Dren02_Cf_BA   AAG---GGTG TGTGAGCAGG GTTTGTTGGG TTGGGGTTAT CAGTTATTAT
Dren07_Cf_BA   AAG---GGTG TGTGAGCAGG GTTTGTTGGG TTGGGGTTAT CAGTTATTAT
Dren08_Cf_BA   AAG---GGTG TGTGAGCAGG GTTTGTTGGG TTGGGGTTAT CAGTTATTAT
Dren09_Cf_BA   AAG---GGTG TGTGAGCAGG GTTTGTTGGG TTGGGGTTAT CAGTTATTAT
Dren10_Cf_BA   AAG---GGTG TGTGAGCAGG GTTTGTTGGG TTGGGGTTAT CAGTTATTAT
Dren18_Cf_BA   AAG---GGTG TGTGAGCAGG GTTTGTTGGG TTGGGGTTAT CAGTTATTAT
Dren19_Cf_BA   AAG---GGTG TGTGAGCAGG GTTTGTTGGG TTGGGGTTAT CAGTTATTAT
Dren23_Cf_BA   AAG---GGTG TGTGAGCAGG GTTTGTTGGG TTGGGGTTAT CAGTTATTAT
Dren25_Cf_SF   AAG---GGTG TGTGAGCAGG GTTTGTTGGG TTGGGGTTAT CAGTTATTAT
Dren32_Cf_SC   AAG---GGTG TGTGAGCAGG GTTTGTTGGG TTGGGGTTAT CAGTTATTAT
```

```
Dren35_Cf_Pa   AAG---GGTG TGTGAGCAGG GTTTGTTGGG TTGGGGTTAT CAGTTATTAT
Dren40_Cf_RG   AAG---GGTG TGTGAGCAGG GTTTGTTGGG TTGGGGTTAT CAGTTATTAT
Dren41_Cf_RG   AAG---GGTG TGTGAGCAGG GTTTGTTGGG TTGGGGTTAT CAGTTATTAT
Dren42_Cf_RG   AAG---GGTG TGTGAGCAGG GTTTGTTGGG TTGGGGTTAT CAGTTATTAT
Dren43_Cf_RG   AAG---GGTG TGTGAGCAGG GTTTGTTGGG TTGGGGTTAT CAGTTATTAT
Dren45_Cf_RG   AAG---GGTG TGTGAGCAGG GTTTGTTGGG TTGGGGTTAT CAGTTATTAT
Dren46_Cf_SF   AAG---GGTG TGTGAGCAGG GTTTGTTGGG TTGGGGTTAT CAGTTATTAT
Dren47_Cb_SF   AAG---GGTG TGTGAGCAGG GTTTGTTGGG TTGGGGTTAT CAGTTATTAT
Dren48_Cf_BA   AAG---GGTG TGTGAGCAGG GTTTGTTGGG TTGGGGTTAT CAGTTATTAT
Dren49_Cf_BA   AAG---GGTG TGTGAGCAGG GTTTGTTGGG TTGGGGTTAT CAGTTATTAT
Dren50_Cf_Ch   AAG---GGTG TGTGAGCAGG GTTTGTTGGG TTGGGGTTAT CAGTTATTAT
Dren53_Cf_BA   AAG---GGTG TGTGAGCAGG GTTTGTTGGG TTGGGGTTAT CAGTTATTAT
Dren58_Cb_SF   AAG---GGTG TGTGAGCAGG GTTTGTTGGG TTGGGGTTAT CAGTTATTAT
Dren66_Cf_SF   AAG---GGTG TGTGAGCAGG GTTTGTTGGG TTGGGGTTAT CAGTTATTAT
Dren67_Cf_SF   AAG---GGTG TGTGAGCAGG GTTTGTTGGG TTGGGGTTAT CAGTTATTAT
Dren68_Cf_SF   AAG---GGTG TGTGAGCAGG GTTTGTTGGG TTGGGGTTAT CAGTTATTAT
Dren69_Cf_SF   AAG---GGTG TGTGAGCAGG GTTTGTTGGG TTGGGGTTAT CAGTTATTAT
Dren70_Cf_SF   AAG---GGTG TGTGAGCAGG GTTTGTTGGG TTGGGGTTAT CAGTTATTAT
Dren72_Cb_SF   AAG---GGTG TGTGAGCAGG GTTTGTTGGG TTGGGGTTAT CAGTTATTAT
Dren73_Cb_Ch   AAG---GGTG TGTGAGCAGG GTTTGTTGGG TTGGGGTTAT CAGTTATTAT
Dren74_Cb_Co   AAG---GGTG TGTGAGCAGG GTTTGTTGGG TTGGGGTTAT CAGTTATTAT
Dren75_Cb_Co   AAG---GGTG TGTGAGCAGG GTTTGTTGGG TTGGGGTTAT CAGTTATTAT
Dren80_Cf_SF   AAG---GGTG TGTGAGCAGG GTTTGTTGGG TTGGGGTTAT CAGTTATTAT
Dren81_Cf_SF   AAG---GGTG TGTGAGCAGG GTTTGTTGGG TTGGGGTTAT CAGTTATTAT
Dren82_Cb_Co   AAG---GGTG TGTGAGCAGG GTTTGTTGGG TTGGGGTTAT CAGTTATTAT
Dren83_Cb_Ch   AAG---GGTG TGTGAGCAGG GTTTGTTGGG TTGGGGTTAT CAGTTATTAT
Dren84_Cf_SF   AAG---GGTG TGTGAGCAGG GTTTGTTGGG TTGGGGTTAT CAGTTATTAT
Dren85_Cb_SF   AAG---GGTG TGTGAGCAGG GTTTGTTGGG TTGGGGTTAT CAGTTATTAT
Dren86_Cb_Co   AAG---GGTG TGTGAGCAGG GTTTGTTGGG TTGGGGTTAT CAGTTATTAT
Dren87_Cf_BA   AAG---GGTG TGTGAGCAGG GTTTGTTGGG TTGGGGTTAT CAGTTATTAT
Dren88_Lg_SF   AAG---GGTG TGTGAGCAGG GTTTGTTGGG TTGGGGTTAT CAGTTATTAT

               ....|....| ....|....| ....|....| ....|....| ....|....|
                   405        415        425        435        445
Dren02_Cf_BA   TCGGTATGTG TTGGCATCAC CGTGGTCTAT AGGGTGAAGG CCTTTTATTA
Dren07_Cf_BA   TCGGTATGTG TTGGCATCAC CGTGGTCTAT AGGGTGAAGG CCTTTTATTA
Dren08_Cf_BA   TCGGTATGTG TTGGCATCAC CGTGGTCTAT AGGGTGAAGG CCTTTTATTA
Dren09_Cf_BA   TCGGTATGTG TTGGCATCAC CGTGGTCTAT AGGGTGAAGG CCTTTTATTA
Dren10_Cf_BA   TCGGTATGTG TTGGCATCAC CGTGGTCTAT AGGGTGAAGG CCTTTTATTA
Dren18_Cf_BA   TCGGTATGTG TTGGCATCAC CGTGGTCTAT AGGGTGAAGG CCTTTTATTA
Dren19_Cf_BA   TCGGTATGTG TTGGCATCAC CGTGGTCTAT AGGGTGAAGG CCTTTTATTA
Dren23_Cf_BA   TCGGTATGTG TTGGCATCAC CGTGGTCTAT AGGGTGAAGG CCTTTTATTA
Dren25_Cf_SF   TCGGTATGTG TTGGCATCAC CGTGGTCTAT AGGGTGAAGG CCTTTTATTA
Dren32_Cf_SC   TCGGTATGTG TTGGCATCAC CGTGGTCTAT AGGGTGAAGG CCTTTTATTA
Dren35_Cf_Pa   TCGGTATGTG TTGGCATCAC CGTGGTCTAT AGGGTGAAGG CCTTTTATTA
Dren40_Cf_RG   TCGGTACGTG TTGGCATCAC CATGGTCTAT AGGGTGAAGG CCTTTTATTA
Dren41_Cf_RG   TCGGTACGTG TTGGCATCAC CATGGTCTAT AGGGTGAAGG CCTTTTATTA
Dren42_Cf_RG   TCGGTATGTG TTGGCATCAC CGTGGTCTAT AGGGTGAAGG CCTTTTATTA
Dren43_Cf_RG   TCGGTATGTG TTGGCATCAC CGTGGTCTAT AGGGTGAAGG CCTTTTATTA
Dren45_Cf_RG   TCGGTATGTG TTGGCATCAC CGTGGTCTAT AGGGTGAAGG CCTTTTATTA
Dren46_Cf_SF   TCGGTATGTG TTGGCATCAC CGTGGTCTAT AGGGTGAAGG CCTTTTATTA
Dren47_Cb_SF   TCGGTATGTG TTGGCATCAC CGTGGTCTAT AGGGTGAAGG CCTTTTATTA
Dren48_Cf_BA   TCGGTATGTG TTGGCATCAC CGTGGTCTAT AGGGTGAAGG CCTTTTATTA
Dren49_Cf_BA   TCGGTATGTG TTGGCATCAC CGTGGTCTAT AGGGTGAAGG CCTTTTATTA
```

```
Dren50_Cf_Ch   TCGGTATGTG TTGGCATCAC CGTGGTCTAT AGGGTGAAGG CCTTTTATTA
Dren53_Cf_BA   TCGGTATGTG TTGGCATCAC CGTGGTCTAT AGGGTGAAGG CCTTTTATTA
Dren58_Cb_SF   TCGGTACGTG TTGGCATCAC CATGGTCTAT AGGGTGAAGG CCTTTTATTA
Dren66_Cf_SF   TCGGTATGTG TTGGCATCAC CGTGGTCTAT AGGGTGAAGG CCTTTTATTA
Dren67_Cf_SF   TCGGTATGTG TTGGCATCAC CGTGGTCTAT AGGGTGAAGG CCTTTTATTA
Dren68_Cf_SF   TCGGTATGTG TTGGCATCAC CGTGGTCTAT AGGGTGAAGG CCTTTTATTA
Dren69_Cf_SF   TCGGTATGTG TTGGCATCAC CGTGGTCTAT AGGGTGAAGG CCTTTTATTA
Dren70_Cf_SF   TCGGTATGTG TTGGCATCAC CGTGGTCTAT AGGGTGAAGG CCTTTTATTA
Dren72_Cb_SF   TCGGTATGTG TTGGCATCAC CGTGGTCTAT AGGGTGAAGG CCTTTTATTA
Dren73_Cb_Ch   TCGGTATGTG TTGGCATCAC CGTGGTCTAT AGGGTGAAGG CCTTTTATTA
Dren74_Cb_Co   TCGGTATGTG TTGGCATCAC CGTGGTCTAT AGGGTGAAGG CCTTTTATTA
Dren75_Cb_Co   TCGGTATGTG TTGGCATCAC CGTGGTCTAT AGGGTGAAGG CCTTTTATTA
Dren80_Cf_SF   TCGGTATGTG TTGGCATCAC CGTGGTCTAT AGGGTGAAGG CCTTTTATTA
Dren81_Cf_SF   TCGGTATGTG TTGGCATCAC CGTGGTCTAT AGGGTGAAGG CCTTTTATTA
Dren82_Cb_Co   TCGGTATGTG TTGGCATCAC CGTGGTCTAT AGGGTGAAGG CCTTTTATTA
Dren83_Cb_Ch   TCGGTATGTG TTGGCATCAC CGTGGTCTAT AGGGTGAAGG CCTTTTATTA
Dren84_Cf_SF   TCGGTATGTG TTGGCATCAC CGTGGTCTAT AGGGTGAAGG CCTTTTATTA
Dren85_Cb_SF   TCGGTATGTG TTGGCATCAC CGTGGTCTAT AGGGTGAAGG CCTTTTATTA
Dren86_Cb_Co   TCGGTATGTG TTGGCATCAC CGTGGTCTAT AGGGTGAAGG CCTTTTATTA
Dren87_Cf_BA   TCGGTATGTG TTGGCATCAC CGTGGTCTAT AGGGTGAAGG CCTTTTATTA
Dren88_Lg_SF   TCGGTATGTG TTGGCATCAC CGTGGTCTAT AGGGTGAAGG CCTTTTATTA

               ....|....| ....|....| ....|....| ....|....| ....|....|
                   455        465        475        485        495
Dren02_Cf_BA   GGCAGTATTA TAATGCTATT ATTTCCGCGC ATGGCATTAT TATAATCTTT
Dren07_Cf_BA   GGCAGTATTA TAATGCTATT ATTTCCGCGC ACGGCATTAT TATAATCTTT
Dren08_Cf_BA   GGCAGTATTA TAATGCTATT ATTTCCGCGC ATGGCATTAT TATAATCTTT
Dren09_Cf_BA   GGCAGTATTA TAATGCTATT ATTTCCGCGC ATGGCATTAT TATAATCTTT
Dren10_Cf_BA   GGCAGTATTA TAATGCTATT ATTTCCGCGC ATGGCATTAT TATAATCTTT
Dren18_Cf_BA   GGCAGTATTA TAATGCTATT ATTTCCGCGC ATGGCATTAT TATAATCTTT
Dren19_Cf_BA   GGCAGTATTA TAATGCTATT ATTTCCGCGC ATGGCATTAT TATAATCTTT
Dren23_Cf_BA   GGCAGTATTA TAATGCTATT ATTTCCGCGC ATGGCATTAT TATAATCTTT
Dren25_Cf_SF   GGCAGTATTA TAATGCTATT ATTTCCGCGC ATGGCATTAT TATAATCTTT
Dren32_Cf_SC   GGCAGTATTA TAATGCTATT ATTTCCGCGC ATGGCATTAT TATAATCTTT
Dren35_Cf_Pa   GGCAGTATTA TAATGCTATT ATTTCCGCGC ATGGCATTAT TATAATCTTT
Dren40_Cf_RG   GGCAGTATTA TAATGCTATT ATTTCCGCGC ATGGCATTAT TATAATCTTT
Dren41_Cf_RG   GGCAGTATTA TAATGCTATT ATTTCCGCGC ATGGCATTAT TATAATCTTT
Dren42_Cf_RG   GGCAGTATTA TAATGCTATT ATTTCCGCGC ACGGCATTAT TATAATCTTT
Dren43_Cf_RG   GGCAGTATTA TAATGCTATT ATTTCCGCGC ATGGCATTAT TATAATCTTT
Dren45_Cf_RG   GGCAGTATTA TAATGCTATT ATTTCCGCGC ATGGCATTAT TATAATCTTT
Dren46_Cf_SF   GGCAGTATTA TAATGCTATT ATTTCCGCGC ATGGCATTAT TATAATCTTT
Dren47_Cb_SF   GGCAGTATTA TAATGCTATT ATTTCCGCGC ATGGCATTAT TATAATCTTT
Dren48_Cf_BA   GGCAGTATTA TAATGCTATT ATTTCCGCGC ATGGCATTAT TATAATCTTT
Dren49_Cf_BA   GGCAGTATTA TAATGCTATT ATTTCCGCGC ATGGCATTAT TATAATCTTT
Dren50_Cf_Ch   GGCAGTATTA TAATGCTATT ATTTCCGCGC ATGGCATTAT TATAATCTTT
Dren53_Cf_BA   GGCAGTATTA TAATGCTATT ATTTCCGCGC ATGGCATTAT TATAATCTTT
Dren58_Cb_SF   GGCAGTATTA TAATGCTATT ATTTCCGCGC ATGGCATTAT TATAATCTTT
Dren66_Cf_SF   GGCAGTATTA TAATGCTATT ATTTCCGCGC ATGGCATTAT TATAATCTTT
Dren67_Cf_SF   GGCAGTATTA TAATGCTATT ATTTCCGCGC ATGGCATTAT TATAATCTTT
Dren68_Cf_SF   GGCAGTATTA TAATGCTATT ATTTCCGCGC ATGGCATTAT TATAATCTTT
Dren69_Cf_SF   GGCAGTATTA TAATGCTATT ATTTCCGCGC ATGGCATTAT TATAATCTTT
Dren70_Cf_SF   GGCAGTATTA TAATGCTATT ATTTCCGCGC ATGGCATTAT TATAATCTTT
Dren72_Cb_SF   GGCAGTATTA TAATGCTATT ATTTCCGCGC ATGGCATTAT TATAATCTTT
Dren73_Cb_Ch   GGCAGTATTA TAATGCTATT ATTTCCGCGC ATGGCATTAT TATAATCTTT
```

```
Dren74_Cb_Co   GGCAGTATTA TAATGCTATT ATTTCCGCGC ATGGCATTAT TATAATCTTT
Dren75_Cb_Co   GGCAGTATTA TAATGCTATT ATTTCCGCGC ATGGCATTAT TATAATCTTT
Dren80_Cf_SF   GGCAGTATTA TAATGCTATT ATTTCCGCGC ATGGCATTAT TATAATCTTT
Dren81_Cf_SF   GGCAGTATTA TAATGCTATT ATTTCCGCGC ATGGCATTAT TATAATCTTT
Dren82_Cb_Co   GGCAGTATTA TAATGCTATT ATTTCCGCGC ATGGCATTAT TATAATCTTT
Dren83_Cb_Ch   GGCAGTATTA TAATGCTATT ATTTCCGCGC ATGGCATTAT TATAATCTTT
Dren84_Cf_SF   GGCAGTATTA TAATGCTATT ATTTCCGCGC ATGGCATTAT TATAATCTTT
Dren85_Cb_SF   GGCAGTATTA TAATGCTATT ATTTCCGCGC ATGGCATTAT TATAATCTTT
Dren86_Cb_Co   GGCAGTATTA TAATGCTATT ATTTCCGCGC ACGGCATTAT TATAATCTTT
Dren87_Cf_BA   GGCAGTATTA TAATGCTATT ATTTCCGCGC ATGGCATTAT TATAATCTTT
Dren88_Lg_SF   GGCAGTATTA TAATGCTATT ATTTCCGCGC ATGGCATTAT TATAATCTTT

               ....|....| ....|....| ....|....| ....|....| ....|....|
                  505        515        525        535        545
Dren02_Cf_BA   TATATAATGA TGCCTATTAT AATAGGTGGG ATGGGTAATA TTATAGTGCC
Dren07_Cf_BA   TATATAATGA TGCCTATTAT AATAGGTGGG ATGGGTAATA TTATAGTGCC
Dren08_Cf_BA   TATATAATGA TGCCTATTAT AATAGGTGGG ATGGGTAATA TTATAGTGCC
Dren09_Cf_BA   TATATAATGA TGCCTATTAT AATAGGTGGG ATGGGTAATA TTATAGTGCC
Dren10_Cf_BA   TATATAATGA TGCCTATTAT AATAGGTGGG ATGGGTAATA TTATAGTGCC
Dren18_Cf_BA   TATATAATGA TGCCTATTAT AATAGGTGGG ATGGGTAATA TTATAGTGCC
Dren19_Cf_BA   TATATAATGA TGCCTATTAT AATAGGTGGG ATGGGTAATA TTATAGTGCC
Dren23_Cf_BA   TATATAATGA TGCCTATTAT AATAGGTGGG GTGGGTAATA TTATAGTGCC
Dren25_Cf_SF   TATATAATGA TGCCTATTAT AATAGGTGGG ATGGGTAATA TTATAGTGCC
Dren32_Cf_SC   TATATAATGA TGCCTATTAT AATAGGTGGG ATGGGTAATA TTATAGTGCC
Dren35_Cf_Pa   TATATAATGA TGCCTATTAT AATAGGTGGG ATGGGTAATA TTATAGTGCC
Dren40_Cf_RG   TATATAATGA TGCCTATTAT AATAGGTGGG ATGGGTAATA TTATAGTGCC
Dren41_Cf_RG   TATATAATGA TGCCTATTAT AATAGGTGGG ATGGGTAATA TTATAGTGCC
Dren42_Cf_RG   TATATAATGA TGCCTATTAT AATAGGTGGG ATGGGTAATA TTATAGTGCC
Dren43_Cf_RG   TATATAATGA TGCCTATTAT AATAGGTGGG ATGGGTAATA TTATAGTGCC
Dren45_Cf_RG   TATATAATGA TGCCTATTAT AATAGGTGGG ATGGGTAATA TTATAGTGCC
Dren46_Cf_SF   TATATAATGA TGCCTATTAT AATAGGTGGG ATGGGTAATA TTATAGTGCC
Dren47_Cb_SF   TATATAATAA TGCCTATTAT AATAGGTGGG ATGGGTAATA TTATAGTGCC
Dren48_Cf_BA   TATATAATGA TGCCTATTAT AATAGGTGGG ATGGGTAATA TTATAGTGCC
Dren49_Cf_BA   TATATAATGA TGCCTATTAT AATAGGTGGG GTGGGTAATA TTATAGTGCC
Dren50_Cf_Ch   TATATAATGA TGCCTATTAT AATAGGTGGG ATGGGTAATA TTATAGTGCC
Dren53_Cf_BA   TATATAATGA TGCCTATTAT AATAGGTGGG ATGGGTAATA TTATAGTGCC
Dren58_Cb_SF   TATATAATGA TGCCTATTAT AATAGGTGGG GTGGGTAATG TTATAGTGCC
Dren66_Cf_SF   TATATAATGA TGCCTATTAT AATAGGTGGG ATGGGTAATA TTATAGTGCC
Dren67_Cf_SF   TATATAATAA TGCCTATTAT AATAGGTGGG ATGGGTAATA TTATAGTGCC
Dren68_Cf_SF   TATATAATAA TGCCTATTAT AATAGGTGGG ATGGGTAATA TTATAGTGCC
Dren69_Cf_SF   TATATAATAA TGCCTATTAT AATAGGTGGG ATGGGTAATA TTATAGTGCC
Dren70_Cf_SF   TATATAATGA TGCCTATTAT AATAGGTGGG ATGGGTAATA TTATAGTGCC
Dren72_Cb_SF   TATATAATGA TGCCTATTAT AATAGGTGGG ATGGGTAATA TTATAGTGCC
Dren73_Cb_Ch   TATATAATAA TGCCTATTAT AATAGGTGGG ATGGGTAATA TTATAGTGCC
Dren74_Cb_Co   TATATAATGA TGCCTATTAT AATAGGTGGG ATGGGTAATA TTATAGTGCC
Dren75_Cb_Co   TATATAATGA TGCCTATTAT AATAGGTGGG ATGGGTAATA TTATAGTGCC
Dren80_Cf_SF   TATATAATGA TGCCTATTAT AATAGGTGGG ATGGGTAATA TTATAGTGCC
Dren81_Cf_SF   TATATAATGA TGCCTATTAT AATAGGTGGG ATGGGTAATA TTATAGTGCC
Dren82_Cb_Co   TATATAATGA TGCCTATTAT AATAGGTGGG ATGGGTAATA TTATAGTGCC
Dren83_Cb_Ch   TATATAATGA TGCCTATTAT AATAGGTGGG ATGGGTAATA TTATAGTGCC
Dren84_Cf_SF   TATATAATGA TGCCTATTAT AATAGGTGGG ATGGGTAATA TTATAGTGCC
Dren85_Cb_SF   TATATAATGA TGCCTATTAT AATAGGTGGG GTGGGTAATA TTATAGTGCC
Dren86_Cb_Co   TATATAATGA TGCCTATTAT AATAGGTGGG ATGGGTAATA TTATAGTGCC
Dren87_Cf_BA   TATATAATAA TGCCTATTAT AATAGGTGGG ATGGGTAATG TTATAGTGCC
```

```
Dren88_Lg_SF   TATATAATAA TGCCTATTAT AATAGGTGGG ATGGGTAATA TTATAGTGCC

               ....|....| ....|....| ....|....| ....|....| ....|....|
                   555        565        575        585        595
Dren02_Cf_BA   TGTTATGTTG GGGCTTCCTG ATATAGCTTT TCCACGTCTT AATAATTTGG
Dren07_Cf_BA   TGTTATGTTG GGGCTTCCTG ATATAGCTTT TCCACGTCTT AATAATTTGG
Dren08_Cf_BA   TGTTATGTTG GGGCTTCCTG ATATAGCTTT TCCACGTCTT AATAATTTGG
Dren09_Cf_BA   TGTTATGTTG GGGCTTCCTG ATATAGCTTT TCCACGTCTT AATAATTTGG
Dren10_Cf_BA   TGTTATGTTG GGGCTTCCTG ATATAGCTTT TCCACGTCTT AATAATTTGG
Dren18_Cf_BA   TGTTATGTTG GGGCTTCCTG ATATAGCTTT TCCACGTCTT AATAATTTGG
Dren19_Cf_BA   TGTTATGTTG GGGCTTCCTG ATATAGCTTT TCCACGTCTT AATAATTTGG
Dren23_Cf_BA   TGTTATGTTG GGGCTTCCTG ATATAGCTTT TCCACGTCTT AATAATTTGG
Dren25_Cf_SF   TGTTATGTTG GGGCTTCCTG ATATAGCTTT TCCACGTCTT AATAATTTGG
Dren32_Cf_SC   TGTTATGTTG GGGCTTCCTG ATATAGCTTT TCCACGTCTT AATAATTTGG
Dren35_Cf_Pa   TGTTATGTTG GGGCTTCCTG ATATAGCTTT TCCACGTCTT AATAATTTGG
Dren40_Cf_RG   TGTTATGTTG GGGCTTCCTG ATATAGCTTT TCCACGTCTT AATAATTTGG
Dren41_Cf_RG   TGTTATGTTG GGGCTTCCTG ATATAGCTTT TCCACGTCTT AATAATTTGG
Dren42_Cf_RG   TGTTATGTTG GGGCTTCCTG ATATAGCTTT TCCACGTCTT AATAATTTGG
Dren43_Cf_RG   TGTTATGTTG GGGCTTCCTG ATATAGCTTT TCCACGTCTT AATAATTTGG
Dren45_Cf_RG   TGTTATGTTG GGGCTTCCTG ATATAGCTTT TCCACGTCTT AATAATTTGG
Dren46_Cf_SF   TGTTATGTTG GGGCTTCCTG ATATAGCTTT TCCACGTCTT AATAATTTGG
Dren47_Cb_SF   TGTTATGTTG GGGCTTCCTG ATATAGCTTT TCCACGTCTT AATAATTTGG
Dren48_Cf_BA   TGTTATGTTG GGGCTTCCTG ATATAGCTTT TCCACGTCTT AATAATTTGG
Dren49_Cf_BA   TGTTATGTTG GGGCTTCCTG ATATAGCTTT TCCACGTCTT AATAATTTGG
Dren50_Cf_Ch   TGTTATGTTG GGGCTTCCTG ATATAGCTTT TCCACGTCTT AATAATTTGG
Dren53_Cf_BA   TGTTATGTTG GGGCTTCCTG ATATAGCTTT TCCACGTCTT AATAATTTGG
Dren58_Cb_SF   TGTTATGTTG GGGCTTCCTG ATATAGCTTT TCCACGTCTT AATAATTTGG
Dren66_Cf_SF   TGTTATGTTG GGGCTTCCTG ATATAGCTTT TCCACGTCTT AATAATTTGG
Dren67_Cf_SF   TGTTATGTTG GGGCTTCCTG ATATAGCTTT TCCACGTCTT AATAATTTGG
Dren68_Cf_SF   TGTTATGTTG GGGCTTCCTG ATATAGCTTT TCCACGTCTT AATAATTTGG
Dren69_Cf_SF   TGTTATGTTG GGGCTTCCTG ATATAGCTTT TCCACGTCTT AATAATTTGG
Dren70_Cf_SF   TGTTATGTTG GGGCTTCCTG ATATAGCTTT TCCACGTCTT AATAATTTGG
Dren72_Cb_SF   TGTTATGTTG GGGCTTCCTG ATATAGCTTT TCCACGTCTT AATAATTTGG
Dren73_Cb_Ch   TGTTATGTTG GGGCTTCCTG ATATAGCTTT TCCACGTCTT AATAATTTGG
Dren74_Cb_Co   TGTTATGTTG GGGCTTCCTG ATATAGCTTT TCCACGTCTT AATAATTTGG
Dren75_Cb_Co   TGTTATGTTG GGGCTTCCTG ATATAGCTTT TCCACGTCTT AATAATTTGG
Dren80_Cf_SF   TGTTATGTTG GGGCTTCCTG ATATAGCTTT TCCACGTCTT AATAATTTGG
Dren81_Cf_SF   TGTTATGTTG GGGCTTCCTG ATATAGCTTT TCCACGTCTT AATAATTTGG
Dren82_Cb_Co   TGTTATGTTG GGGCTTCCTG ATATAGCTTT TCCACGTCTT AATAATTTGG
Dren83_Cb_Ch   TGTTATGTTG GGGCTTCCTG ATATAGCTTT TCCACGTCTT AATAATTTGG
Dren84_Cf_SF   TGTTATGTTG GGGCTTCCTG ATATAGCTTT TCCACGTCTT AATAATTTGG
Dren85_Cb_SF   TGTTATGTTG GGGCTTCCTG ATATAGCTTT TCCACGTCTT AATAATTTGG
Dren86_Cb_Co   TGTTATGTTG GGGCTTCCTG ATATAGCTTT TCCACGTCTT AATAATTTGG
Dren87_Cf_BA   TGTTATGTTG GGGCTTCCTG ATATAGCTTT TCCACGTCTT AATAATTTGG
Dren88_Lg_SF   TGTTATGTTG GGGCTTCCTG ATATAGCTTT TCCACGTCTT AATAATTTGG

               ....|....| ....|....| ....|....| ....|....| ....|....|
                   605        615        625        635        645
Dren02_Cf_BA   GGTTTTGGTT ATTGCCTCCT TCATTCTTAT TGTTTGTAAG TTCCATAATG
Dren07_Cf_BA   GGTTTTGGTT ATTGCCTCCT TCATTCTTAT TGTTTGTAAG TTCCATAATG
Dren08_Cf_BA   GGTTTTGGTT GTTGCCTCCT TCATTCTTAT TGTTTGTAAG TTCCATAATG
Dren09_Cf_BA   GGTTTTGGTT ATTGCCTCCT TCATTCTTAT TGTTTGTAAG TTCCATAATG
Dren10_Cf_BA   GGTTTTGGTT GTTGCCTCCT TCATTCTTAT TGTTTGTAAG TTCCATAATG
Dren18_Cf_BA   GGTTTTGGTT ATTGCCTCCT TCATTCTTAT TGTTTGTAAG TTCCATAATG
```

```
Dren19_Cf_BA   GGTTTTGGTT ATTGCCTCCT TCATTCTTAT TGTTTGTAAG TTCCATAATG
Dren23_Cf_BA   GGTTTTGGTT ATTGCCTCCT TCATTCTTAT TGTTTGTAAG TTCCATAATG
Dren25_Cf_SF   GGTTTTGGTT ATTGCCTCCT TCATTCTTAT TGTTTGTAAG TTCCATAATG
Dren32_Cf_SC   GGTTTTGGTT ATTGCCTCCT TCATTCTTAT TGTTTGTAAG TTCCATAATG
Dren35_Cf_Pa   GGTTTTGGTT ATTGCCTCCT TCATTCTTAT TGTTTGTAAG TTCCATAATG
Dren40_Cf_RG   GGTTTTGGTT ATTGCCTCCT TCATTCTTAT TGTTTGTAAG TTCCACGATG
Dren41_Cf_RG   GGTTTTGGTT ATTGCCTCCT TCATTCTTAT TGTTTGTAAG TTCCACGATG
Dren42_Cf_RG   GGTTTTGGTT ATTGCCTCCT TCATTCTTAT TGTTTGTAAG TTCCATAATG
Dren43_Cf_RG   GGTTTTGGTT ATTGCCTCCT TCATTCTTAT TGTTTGTAAG TTCCATAATG
Dren45_Cf_RG   GGTTTTGGTT ATTGCCTCCT TCATTCTTAT TGTTTGTAAG TTCCATAATG
Dren46_Cf_SF   GGTTTTGGTT ATTGCCTCCT TCATTCTTAT TGTTTGTAAG TTCCATAATG
Dren47_Cb_SF   GGTTTTGGTT ATTGCCTCCT TCATTCTTAT TGTTTGTAAG TTCCATAATG
Dren48_Cf_BA   GGTTTTGGTT GTTGCCTCCT TCATTCTTAT TGTTTGTAAG TTCCATAATG
Dren49_Cf_BA   GGTTTTGGTT ATTGCCTCCT TCATTCTTAT TGTTTGTAAG TTCCATAATG
Dren50_Cf_Ch   GGTTTTGGTT GTTGCCTCCT TCATTCTTAT TGTTTGTAAG TTCCATAATG
Dren53_Cf_BA   GGTTTTGGTT GTTGCCTCCT TCATTCTTAT TGTTTGTAAG TTCCATAATG
Dren58_Cb_SF   GGTTTTGGTT ATTGCCTCCT TCATTCTTAT TGTTTGTAAG TTCCATGATG
Dren66_Cf_SF   GGTTTTGGTT ATTGCCTCCT TCATTCTTAT TGTTTGTAAG TTCCATAATG
Dren67_Cf_SF   GGTTTTGGTT ATTGCCTCCT TCATTCTTAT TGTTTGTAAG TTCCATAATG
Dren68_Cf_SF   GGTTTTGGTT ATTGCCTCCT TCATTCTTAT TGTTTGTAAG TTCCATAATG
Dren69_Cf_SF   GGTTTTGGTT ATTGCCTCCT TCATTCTTAT TGTTTGTAAG TTCCATAATG
Dren70_Cf_SF   GGTTTTGGTT ATTGCCTCCT TCATTCTTAT TGTTTGTAAG TTCCATAATG
Dren72_Cb_SF   GGTTTTGGTT GTTGCCTCCT TCATTCTTAT TGTTTGTAAG TTCCATAATG
Dren73_Cb_Ch   GGTTTTGGTT ATTGCCTCCT TCATTCTTAT TGTTTGTAAG TTCCATAATG
Dren74_Cb_Co   GGTTTTGGTT ATTGCCTCCT TCATTCTTAT TGTTTGTAAG TTCCATAATG
Dren75_Cb_Co   GGTTTTGGTT GTTGCCTCCT TCATTCTTAT TGTTTGTAAG TTCCATAATG
Dren80_Cf_SF   GGTTTTGGTT ATTGCCTCCT TCATTCTTAT TGTTTGTAAG TTCCATAATG
Dren81_Cf_SF   GGTTTTGGTT ATTGCCTCCT TCATTCTTAT TGTTTGTAAG TTCCATAATG
Dren82_Cb_Co   GGTTTTGGTT ATTGCCTCCT TCATTCTTAT TGTTTGTAAG TTCCATAATG
Dren83_Cb_Ch   GGTTTTGGTT GTTGCCTCCT TCATTCTTAT TGTTTGTAAG TTCCATAATG
Dren84_Cf_SF   GGTTTTGGTT ATTGCCTCCT TCATTCTTAT TGTTTGTAAG TTCCATAATG
Dren85_Cb_SF   GGTTTTGGTT ATTGCCTCCT TCATTCTTAT TGTTTGTAAG TTCCATAATG
Dren86_Cb_Co   GGTTTTGGTT ATTGCCTCCT TCATTCTTAT TGTTTGTAAG TTCCATAATG
Dren87_Cf_BA   GGTTTTGGTT ATTGCCTCCT TCATTCTTAT TGTTTGTAAG TTCCATAATG
Dren88_Lg_SF   GGTTTTGGTT ATTGCCTCCT TCATTCTTAT TGTTTGTAAG TTCCATAATG

               ....|....| ....|....| ....|....| ....|....| ....|....|
                  655        665        675        685        695
Dren02_Cf_BA   TTTTCGGTTT CTGGCCCGGG CACAGGTTGA ACCCTTTACC CTCCTTTATC
Dren07_Cf_BA   TTTTCGGTTT CTGGCCCGGG CACAGGTTGA ACCCTTTACC CTCCTCTATC
Dren08_Cf_BA   TTTTCGGTTT CTGGCCCGGG CACAGGTTGA ACCCTTTACC CTCCTCTATC
Dren09_Cf_BA   TTTTCGGTTT CTGGCCCGGG CACAGGTTGG ACCCTTTACC CTCCTCTATC
Dren10_Cf_BA   TTTTCGGTTT CTGGCCCGGG CACAGGTTGA ACCCTTTACC CTCCTCTATC
Dren18_Cf_BA   TTTTCGGTTT CTGGCCCGGG CACAGGTTGA ACCCTTTACC CTCCTCTATC
Dren19_Cf_BA   TTTTCGGTTT CTGGCCCGGG CACAGGTTGA ACCCTTTACC CTCCTCTATC
Dren23_Cf_BA   TTTTCGGTTT CTGGCCCGGG CACAGGTTGG ACCCTCTACC CTCCTCTATC
Dren25_Cf_SF   TTTTCGGTTT CTGGCCCGGG CACAGGTTGG ACCCTTTACC CTCCTCTATC
Dren32_Cf_SC   TTTTCGGTTT CTGGCCCGGG CACAGGTTGG ACCCTTTACC CTCCTCTATC
Dren35_Cf_Pa   TTTTCGGTTT CTGGCCCGGG CACAGGTTGG ACCCTTTACC CTCCTCTATC
Dren40_Cf_RG   TTTTCGGTTT CTGGCCCGGG CACAGGTTGA ACCCTTTACC CTCCTCTATC
Dren41_Cf_RG   TTTTCGGTTT CTGGCCCGGG CACAGGTTGA ACCCTTTACC CTCCTCTATC
Dren42_Cf_RG   TTTTCGGTTT CTGGCCCGGG CACAGGTTGA ACCCTTTACC CTCCTCTATC
Dren43_Cf_RG   TTTTCGGTTT CTGGCCCGGG CACAGGTTGA ACCCTTTACC CTCCTCTATC
Dren45_Cf_RG   TTTTCGGTTT CTGGCCCGGG CACAGGTTGA ACCCTTTACC CTCCTCTATC
```

```
Dren46_Cf_SF   TTTTCGGTTT CTGGCCCGGG CACAGGTTGG ACCCTTTACC CTCCTCTATC
Dren47_Cb_SF   TTTTCGGTTT CTGGCCCGGG CACAGGTTGA ACCCTTTACC CTCCTCTATC
Dren48_Cf_BA   TTTTCGGTTT CTGGCCCGGG CACAGGTTGA ACCCTTTACC CTCCTCTATC
Dren49_Cf_BA   TTTTCGGTTT CTGGCCCGGG CACAGGTTGG ACCCTCTACC CTCCTCTATC
Dren50_Cf_Ch   TTTTCGGTTT CTGGCCCGGG CACAGGTTGA ACCCTTTACC CTCCTCTATC
Dren53_Cf_BA   TTTTCGGTTT CTGGCCCGGG CACAGGTTGA ACCCTTTACC CTCCTCTATC
Dren58_Cb_SF   TTTTCGGTTT CTGGCCCGGG CACAGGTTGA ACCCTTTACC CTCCTCTATC
Dren66_Cf_SF   TTTTCGGTTT CTGGCCCGGG CACAGGTTGA ACCCTTTACC CTCCTCTATC
Dren67_Cf_SF   TTTTCGGTTT CTGGCCCGGG CACAGGTTGA ACCCTTTACC CTCCTCTATC
Dren68_Cf_SF   TTTTCGGTTT CTGGCCCGGG CACAGGTTGA ACCCTTTACC CTCCTCTATC
Dren69_Cf_SF   TTTTCGGTTT CTGGCCCGGG CACAGGTTGA ACCCTTTACC CTCCTCTATC
Dren70_Cf_SF   TTTTCGGTTT CTGGCCCGGG CACAGGTTGG ACCCTTTACC CTCCTCTATC
Dren72_Cb_SF   TTTTCGGTTT CTGGCCCGGG CACAGGTTGA ACCCTTTACC CTCCTCTATC
Dren73_Cb_Ch   TTTTCGGTTT CTGGCCCGGG CACAGGTTGA ACCCTTTACC CTCCTCTATC
Dren74_Cb_Co   TTTTCGGTTT CTGGCCCGGG CACAGGTTGG ACCCTTTACC CTCCTCTATC
Dren75_Cb_Co   TTTTCGGTTT CTGGCCCGGG CACAGGTTGA ACCCTTTACC CTCCTCTATC
Dren80_Cf_SF   TTTTCGGTTT CTGGCCCGGG CACAGGTTGG ACCCTTTACC CTCCTCTATC
Dren81_Cf_SF   TTTTCGGTTT CTGGCCCGGG CACAGGTTGG ACCCTTTACC CTCCTCTATC
Dren82_Cb_Co   TTTTCGGTTT CTGGCCCGGG CACAGGTTGG ACCCTTTACC CTCCTCTATC
Dren83_Cb_Ch   TTTTCGGTTT CTGGCCCGGG CACAGGTTGA ACCCTTTACC CTCCTCTATC
Dren84_Cf_SF   TTTTCGGTTT CTGGCCCGGG CACAGGTTGG ACCCTTTACC CTCCTCTATC
Dren85_Cb_SF   TTTTCGGTTT CTGGCCCGGG CACAGGTTGG ACCCTCTACC CTCCTCTATC
Dren86_Cb_Co   TTTTCGGTTT CTGGCCCGGG CACAGGTTGA ACCCTTTACC CTCCTCTATC
Dren87_Cf_BA   TTTTCGGTTT CTGGCCCGGG CACAGGTTGA ACCCTTTACC CTCCTCTATC
Dren88_Lg_SF   TTTTCGGTTT CTGGCCCGGG CACAGGTTGA ACCCTTTACC CTCCTCTATC

               ....|....| ....|....| ....|....| ....|....| ....|....|
                    705        715        725        735        745
Dren02_Cf_BA   TTTATTTACT GGGCATCCTG ATGTTTGCGT GGATTTTGTG ATCTTTTCTC
Dren07_Cf_BA   TTTATTTACT GGGCATCCTG ATGTTTGCGT GGATTTTGTG ATCTTTTCTC
Dren08_Cf_BA   TTTATTTACT GGGCATCCTG ATGTTTGCGT GGATTTTGTG ATCTTTTCTC
Dren09_Cf_BA   TTTATTTACT GGGCATCCTG ATGTTTGCGT GGATTTTGTG ATCTTTTCTC
Dren10_Cf_BA   TTTATTTACT GGGCATCCTG ATGTTTGCGT GGATTTTGTG ATCTTTTCTC
Dren18_Cf_BA   TTTATTTACT GGACATCCTG ATGTCTGCGT GGATTTTGTG ATCTTTTCTC
Dren19_Cf_BA   TTTATTTACT GGACATCCTG ATGTCTGCGT GGATTTTGTG ATCTTTTCTC
Dren23_Cf_BA   TTTATTTACT GGGCATCCTG ATGTTTGCGT GGATTTTGTG ATCTTTTCTC
Dren25_Cf_SF   TTTATTTACT GGGCATCCTG ATGTTTGCGT GGATTTTGTG ATCTTTTCTC
Dren32_Cf_SC   TTTATTTACT GGGCATCCTG ATGTTTGCGT GGATTTTGTG ATCTTTTCTC
Dren35_Cf_Pa   TTTATTTACT GGGCATCCTG ATGTTTGCGT GGATTTTGTG ATCTTTTCTC
Dren40_Cf_RG   TTTATTTACT GGGCATCCTG ATGTTTGCGT GGATTTTGTG ATCTTTTCTC
Dren41_Cf_RG   TTTATTTACT GGGCATCCTG ATGTTTGCGT GGATTTTGTG ATCTTTTCTC
Dren42_Cf_RG   TTTATTTACT GGGCATCCTG ATGTTTGCGT GGATTTTGTG ATCTTTTCTC
Dren43_Cf_RG   TTTATTTACT GGGCATCCTG ATGTTTGCGT GGATTTTGTG ATCTTTTCTC
Dren45_Cf_RG   TTTATTTACT GGGCATCCTG ATGTTTGCGT GGATTTTGTG ATCTTTTCTC
Dren46_Cf_SF   TTTATTTACT GGGCATCCTG ATGTTTGCGT GGATTTTGTG ATCTTTTCTC
Dren47_Cb_SF   TTTATTTACT GGGCATCCTG ATGTTTGCGT GGATTTTGTG ATCTTTTCTC
Dren48_Cf_BA   TTTATTTACT GGGCATCCTG ATGTTTGCGT GGATTTTGTG ATCTTTTCTC
Dren49_Cf_BA   TTTATTTACT GGGCATCCTG ATGTTTGCGT GGATTTTGTG ATCTTTTCTC
Dren50_Cf_Ch   TTTATTTACT GGGCATCCTG ATGTTTGCGT GGATTTTGTG ATCTTTTCTC
Dren53_Cf_BA   TTTATTTACT GGACATCCTG ATGTTTGCGT GGATTTTGTG ATCTTTTCTC
Dren58_Cb_SF   TTTATTTACT GGGCATCCTG ATGTTTGCGT GGATTTTGTG ATCTTTTCTC
Dren66_Cf_SF   TTTATTTACT GGACATCCTG ATGTCTGCGT GGATTTTGTG ATCTTTTCTC
Dren67_Cf_SF   TTTATTTACT GGGCATCCTG ATGTTTGCGT GGATTTTGTG ATCTTTTCTC
Dren68_Cf_SF   TTTATTTACT GGGCATCCTG ATGTTTGCGT GGATTTTGTG ATCTTTTCTC
```

```
Dren69_Cf_SF   TTTATTTACT GGGCATCCTG ATGTTTGCGT GGATTTTGTG ATCTTTTCTC
Dren70_Cf_SF   TTTATTTACT GGGCATCCTG ATGTTTGCGT GGATTTTGTG ATCTTTTCTC
Dren72_Cb_SF   TTTATTTACT GGGCATCCTG ATGTTTGCGT GGATTTTGTG ATCTTTTCTC
Dren73_Cb_Ch   TTTATTTACT GGGCATCCTG ATGTTTGCGT GGATTTTGTG ATCTTTTCTC
Dren74_Cb_Co   TTTATTTACT GGTCATCCTG ATGTTTGCGT GGATTTTGTG ATCTTTTCTC
Dren75_Cb_Co   TTTATTTACT GGGCATCCTG ATGTTTGCGT GGATTTTGTG ATCTTTTCTC
Dren80_Cf_SF   TTTATTTACT GGGCATCCTG ATGTTTGCGT GGATTTTGTG ATCTTTTCTC
Dren81_Cf_SF   TTTATTTACT GGGCATCCTG ATGTTTGCGT GGATTTTGTG ATCTTTTCTC
Dren82_Cb_Co   TTTATTTACT GGGCATCCTG ATGTTTGCGT GGATTTTGTG ATCTTTTCTC
Dren83_Cb_Ch   TTTATTTACT GGGCATCCTG ATGTTTGCGT GGATTTTGTG ATCTTTTCTC
Dren84_Cf_SF   TTTATTTACT GGGCATCCTG ATGTTTGCGT GGATTTTGTG ATCTTTTCTC
Dren85_Cb_SF   TTTATTTACT GGGCATCCTG ATGTTTGCGT GGATTTTGTG ATCTTTTCTC
Dren86_Cb_Co   TTTATTTACT GGGCATCCTG ATGTTTGCGT GGATTTTGTG ATCTTTTCTC
Dren87_Cf_BA   TTTATTTACT GGGCATCCTG ATGTTTGCGT GGATTTTGTG ATCTTTTCTC
Dren88_Lg_SF   TTTATTTACT GGGCATCCTG ATGTTTGCGT GGATTTTGTG ATCTTTTCTC

               ....|....| ....|....| ....|....| ....|....| ....|....|
                    755        765        775        785        795
Dren02_Cf_BA   TACATTTGGC GGGGGTGTCT TCTCTATTAA GAAGTGTAAA TTTTGTAACG
Dren07_Cf_BA   TACATTTGGC GGGGGTGTCT TCTCTATTAA GAAGTGTAAA TTTTGTAACG
Dren08_Cf_BA   TACATTTGGC GGGGGTGTCT TCTCTATTAA GAAGTGTAAA TTTTGTAACG
Dren09_Cf_BA   TACATTTGGC GGGGGTGTCT TCTCTATTAA GAAGTGTAAA TTTTGTAACG
Dren10_Cf_BA   TACATTTGGC GGGGGTGTCT TCTCTATTAA GAAGTGTAAA TTTTGTAACG
Dren18_Cf_BA   TACATTTGGC GGGGGTGTCT TCTCTATTAA GAAGTGTAAA TTTTGTAACG
Dren19_Cf_BA   TACATTTGGC GGGGGTGTCT TCTCTATTAA GAAGTGTAAA TTTTGTAACG
Dren23_Cf_BA   TACATTTGGC GGGGGTGTCT TCTCTATTAA GAAGTGTAAA TTTTGTAACG
Dren25_Cf_SF   TACATTTGGC GGGGGTGTCT TCTCTATTAA GAAGTGTAAA TTTTGTAACG
Dren32_Cf_SC   TACATTTGGC GGGGGTGTCT TCTCTATTAA GAAGTGTAAA TTTTGTAACG
Dren35_Cf_Pa   TACATTTGGC GGGGGTGTCT TCTCTATTAA GAAGTGTAAA TTTTGTAACG
Dren40_Cf_RG   TACATTTGGC GGGGGTGTCT TCTCTATTAA GAAGTGTAAA TTTTGTAACG
Dren41_Cf_RG   TACATTTGGC GGGGGTGTCT TCTCTATTAA GAAGTGTAAA TTTTGTAACG
Dren42_Cf_RG   TACATTTGGC GGGGGTGTCT TCTCTATTAA GAAGTGTAAA TTTTGTAACG
Dren43_Cf_RG   TACATTTGGC GGGGGTGTCT TCTCTATTAA GAAGTGTAAA TTTTGTAACG
Dren45_Cf_RG   TACATTTGGC GGGGGTGTCT TCTCTATTAA GAAGTGTAAA TTTTGTAACG
Dren46_Cf_SF   TACATTTGGC GGGGGTGTCT TCTCTATTAA GAAGTGTAAA TTTTGTAACG
Dren47_Cb_SF   TACATTTGGC GGGGGTGTCT TCTCTATTAA GAAGTGTAAA TTTTGTAACG
Dren48_Cf_BA   TACATTTGGC GGGGGTGTCT TCTCTATTAA GAAGTGTAAA TTTTGTAACG
Dren49_Cf_BA   TACATTTGGC GGGGGTGTCT TCTCTATTAA GAAGTGTAAA TTTTGTAACG
Dren50_Cf_Ch   TACATTTGGC GGGGGTGTCT TCTCTATTAA GAAGTGTAAA TTTTGTAACG
Dren53_Cf_BA   TACATTTGGC GGGGGTGTCT TCTCTATTAA GAAGTGTAAA TTTTGTAACG
Dren58_Cb_SF   TACATTTGGC GGGGGTGTCT TCTCTATTAA GAAGTGTAAA TTTTGTAACG
Dren66_Cf_SF   TACATTTGGC GGGGGTGTCT TCTCTATTAA GAAGTGTAAA TTTTGTAACG
Dren67_Cf_SF   TACATTTGGC GGGGGTGTCT TCTCTATTAA GAAGTGTAAA TTTTGTAACG
Dren68_Cf_SF   TACATTTGGC GGGGGTGTCT TCTCTATTAA GAAGTGTAAA TTTTGTAACG
Dren69_Cf_SF   TACATTTGGC GGGGGTGTCT TCTCTATTAA GAAGTGTAAA TTTTGTAACG
Dren70_Cf_SF   TACATTTGGC GGGGGTGTCT TCTCTATTAA GAAGTGTAAA TTTTGTAACG
Dren72_Cb_SF   TACATTTGGC GGGGGTGTCT TCTCTATTAA GAAGTGTAAA TTTTGTAACG
Dren73_Cb_Ch   TACATTTGGC GGGGGTGTCT TCTCTATTAA GAAGTGTAAA TTTTGTAACG
Dren74_Cb_Co   TACATTTGGC GGGGGTGTCT TCTCTATTAA GAAGTGTAAA TTTTGTAACG
Dren75_Cb_Co   TACATTTGGC GGGGGTGTCT TCTCTATTAA GAAGTGTAAA TTTTGTAACG
Dren80_Cf_SF   TACATTTGGC GGGGGTGTCT TCTCTATTAA GAAGTGTAAA TTTTGTAACG
Dren81_Cf_SF   TACATTTGGC GGGGGTGTCT TCTCTATTAA GAAGTGTAAA TTTTGTAACG
Dren82_Cb_Co   TACATTTGGC GGGGGTGTCT TCTCTATTAA GAAGTGTAAA TTTTGTAACG
Dren83_Cb_Ch   TACATTTGGC GGGGGTGTCT TCTCTATTAA GAAGTGTAAA TTTTGTAACG
```

```
Dren84_Cf_SF   TACATTTGGC GGGGGTGTCT TCTCTATTAA GAAGTGTAAA TTTTGTAACG
Dren85_Cb_SF   TACATTTGGC GGGGGTGTCT TCTCTATTAA GAAGTGTAAA TTTTGTAACG
Dren86_Cb_Co   TACATTTGGC GGGGGTGTCT TCTCTATTAA GAAGTGTAAA TTTTGTAACG
Dren87_Cf_BA   TACATTTGGC GGGGGTGTCT TCTCTATTAA GAAGTGTAAA TTTTGTAACG
Dren88_Lg_SF   TACATTTGGC GGGGGTGTCT TCTCTATTAA GAAGTGTAAA TTTTGTAACG

               ....|....| ....|....| ....|....| ....|....| ....|....|
                    805        815        825        835        845
Dren02_Cf_BA   ACGTGTTTTT CTGTTTGTGA TTCGGTTTCT TCATTAGAAG ATATGCCAAT
Dren07_Cf_BA   ACGTGTTTTT CTGTTTGTGA TTCGGTTTCT TCATTAGAAG ATATGCCAAT
Dren08_Cf_BA   ACGTGTTTTT CTGTTTGTGA TTCGGTTTCT TCATTAGAAG ATATGCCAAT
Dren09_Cf_BA   ACGTGTTTTT CTGTTTGTGG TTCGGTTTCT TCATTAGAAG ATATGCCAAT
Dren10_Cf_BA   ACGTGTTTTT CTGTTTGTGA TTCGGTTTCT TCATTAGAAG ATATGCCAAT
Dren18_Cf_BA   ACGTGTTTTT CTGTTTGTGA TTCGGTTTCT TCATTAGAAG ATATGCCAAT
Dren19_Cf_BA   ACGTGTTTTT CTGTTTGTGA TTCGGTTTCT TCATTAGAAG ATATGCCAAT
Dren23_Cf_BA   ACGTGTTTTT CTGTTTGTGA TTCGGTTTCT TCATTAGAAG ATATGCCAAT
Dren25_Cf_SF   ACGTGTTTTT CTGTTTGTGA TTCGGTTTCT TCATTAGAAG ATATGCCAAT
Dren32_Cf_SC   ACGTGTTTTT CTGTTTGTGA TTCGGTTTCT TCATTAGAAG ATATGCCAAT
Dren35_Cf_Pa   ACGTGTTTTT CTGTTTGTGA TTCGGTTTCT TCATTAGAAG ATATGCCAAT
Dren40_Cf_RG   ACGTGTTTTT CTGTTTGTGA TTCGGTTTCT TCATTAGAAG ATATGCCAAT
Dren41_Cf_RG   ACGTGTTTTT CTGTTTGTGA TTCGGTTTCT TCATTAGAAG ATATGCCAAT
Dren42_Cf_RG   ACGTGTTTTT CTGTTTGTGA TTCGGTTTCT TCATTAGAAG ATATGCCAAT
Dren43_Cf_RG   ACGTGTTTTT CTGTTTGTGA TTCGGTTTCT TCATTAGAAG ATATGCCAAT
Dren45_Cf_RG   ACGTGTTTTT CTGTTTGTGA TTCGGTTTCT TCATTAGAAG ATATGCCAAT
Dren46_Cf_SF   ACGTGTTTTT CTGTTTGTGA TTCGGTTTCT TCATTAGAAG ATATGCCAAT
Dren47_Cb_SF   ACGTGTTTTT CTGTTTGTGA TTCGGTTTCT TCATTAGAAG ATATGCCAAT
Dren48_Cf_BA   ACGTGTTTTT CTGTTTGTGA TTCGGTTTCT TCATTAGAAG ATATGCCAAT
Dren49_Cf_BA   ACGTGTTTTT CTGTTTGTGA TTCGGTTTCT TCATTAGAAG ATATGCCAAT
Dren50_Cf_Ch   ACGTGTTTTT CTGTTTGTGA TTCGGTTTCT TCATTAGAAG ATATGCCAAT
Dren53_Cf_BA   ACGTGTTTTT CTGTTTGTGA TTCGGTTTCT TCATTAGAAG ATATGCCAAT
Dren58_Cb_SF   ACGTGTTTTT CTGTTTGTGA TTCGGTTTCT TCATTAGAAG ATATGCCAAT
Dren66_Cf_SF   ACGTGTTTTT CTGTTTGTGA TTCGGTTTCT TCATTAGAAG ATATGCCAAT
Dren67_Cf_SF   ACGTGTTTTT CTGTTTGTGA TTCGGTTTCT TCATTAGAAG ATATGCCAAT
Dren68_Cf_SF   ACGTGTTTTT CTGTTTGTGA TTCGGTTTCT TCATTAGAAG ATATGCCAAT
Dren69_Cf_SF   ACGTGTTTTT CTGTTTGTGA TTCGGTTTCT TCATTAGAAG ATATGCCAAT
Dren70_Cf_SF   ACGTGTTTTT CTGTTTGTGA TTCGGTTTCT TCATTAGAAG ATATGCCAAT
Dren72_Cb_SF   ACGTGTTTTT CTGTTTGTGA TTCGGTTTCT TCATTAGAAG ATATGCCAAT
Dren73_Cb_Ch   ACGTGTTTTT CTGTTTGTGA TTCGGTTTCT TCATTAGAAG ATATGCCAAT
Dren74_Cb_Co   ACGTGTTTTT CTGTTTGTGA TTCGGTTTCT TCATTAGAAG ATATGCCAAT
Dren75_Cb_Co   ACGTGTTTTT CTGTTTGTGA TTCGGTTTCT TCATTAGAAG ATATGCCAAT
Dren80_Cf_SF   ACGTGTTTTT CTGTTTGTGA TTCGGTTTCT TCATTAGAAG ATATGCCAAT
Dren81_Cf_SF   ACGTGTTTTT CTGTTTGTGA TTCGGTTTCT TCATTAGAAG ATATGCCAAT
Dren82_Cb_Co   ACGTGTTTTT CTGTTTGTGA TTCGGTTTCT TCATTAGAAG ATATGCCAAT
Dren83_Cb_Ch   ACGTGTTTTT CTGTTTGTGA TTCGGTTTCT TCATTAGAAG ATATGCCAAT
Dren84_Cf_SF   ACGTGTTTTT CTGTTTGTGA TTCGGTTTCT TCATTAGAAG ATATGCCAAT
Dren85_Cb_SF   ACGTGTTTTT CTGTTTGTGA TTCGGTTTCT TCATTAGAAG ATATGCCAAT
Dren86_Cb_Co   ACGTGTTTTT CTGTTTGTGA TTCGGTTTCT TCATTAGAAG ATATGCCAAT
Dren87_Cf_BA   ACGTGTTTTT CTGTTTGTGA TTCGGTTTCT TCATTAGAAG ATATGCCAAT
Dren88_Lg_SF   ACGTGTTTTT CTGTTTGTGA TTCGGTTTCT TCATTAGAAG ATATGCCAAT

               ....|....| ....|....| ....|....| ....|....| ....|....|
                    855        865        875        885        895
Dren02_Cf_BA   GTTTGCGTGA AGCATTGTGG TGACGTCCTT CATACTAATT GTTTCTCTTC
Dren07_Cf_BA   GTTTGCGTGA AGCATTGTGG TGACGTCCTT CATACTAATT GTTTCTCTTC
```

```
Dren08_Cf_BA   GTTTGCGTGA AGCATTGTGG TGACGTCCTT CATACTAATT GTTTCTCTTC
Dren09_Cf_BA   GTTTGCGTGA AGCATTGTGG TGACGTCCTT CATACTAATT GTTTCTCTTC
Dren10_Cf_BA   GTTTGCGTGA AGCATTGTGG TGACGTCCTT CATACTAATT GTTTCTCTTC
Dren18_Cf_BA   GTTTGCGTGA AGCATTGTGG TGACGTCCTT CATACTAATT GTTTCTCTTC
Dren19_Cf_BA   GTTTGCGTGA AGCATTGTGG TGACGTCCTT CATACTAATT GTTTCTCTTC
Dren23_Cf_BA   GTTTGCGTGA AGCATTGTGG TGACGTCCTT CATACTAATT GTCTCTCTTC
Dren25_Cf_SF   GTTTGCGTGA AGCATTGTGG TGACGTCCTT CATACTAATT GTTTCTCTTC
Dren32_Cf_SC   GTTTGCGTGA AGCATTGTGG TGACGTCCTT CATACTAATT GTTTCTCTTC
Dren35_Cf_Pa   GTTTGCGTGA AGCATTGTGG TGACGTCCTT CATACTAATT GTTTCTCTTC
Dren40_Cf_RG   GTTTGCGTGA AGCATTGTGG TGACGTCCTT CATACTAATT GTTTCTCTTC
Dren41_Cf_RG   GTTTGCGTGA AGCATTGTGG TGACGTCCTT CATACTAATT GTTTCTCTTC
Dren42_Cf_RG   GTTTGCGTGA AGCATTGTGG TGACGTCCTT CATACTAATT GTTTCTCTTC
Dren43_Cf_RG   GTTTGCGTGA AGCATTGTGG TGACGTCCTT CATACTAATT GTTTCTCTTC
Dren45_Cf_RG   GTTTGCGTGA AGCATTGTGG TGACGTCCTT CATACTAATT GTTTCTCTTC
Dren46_Cf_SF   GTTTGCGTGA AGCATTGTGG TGACGTCCTT CATACTAATT GTTTCTCTTC
Dren47_Cb_SF   GTTTGCGTGA AGCATTGTGG TGACGTCCTT CATACTAATT GTTTCTCTTC
Dren48_Cf_BA   GTTTGCGTGA AGCATTGTGG TGACGTCCTT CATACTAATT GTTTCTCTTC
Dren49_Cf_BA   GTTTGCGTGA AGCATTGTGG TGACGTCCTT CATACTAATT GTTTCTCTTC
Dren50_Cf_Ch   GTTTGCGTGA AGCATTGTGG TGACGTCCTT CATACTAATT GTTTCTCTTC
Dren53_Cf_BA   GTTTGCGTGA AGCATTGTGG TGACGTCCTT CATACTAATT GTTTCTCTTC
Dren58_Cb_SF   GTTTGCGTGA AGCATTGTGG TGACGTCCTT CATACTAATT GTTTCTCTTC
Dren66_Cf_SF   GTTTGCGTGA AGCATTGTGG TGACGTCCTT CATACTAATT GTTTCTCTTC
Dren67_Cf_SF   GTTTGCGTGA AGCATTGTGG TGACGTCCTT CATACTAATT GTTTCTCTTC
Dren68_Cf_SF   GTTTGCGTGA AGCATTGTGG TGACGTCCTT CATACTAATT GTTTCTCTTC
Dren69_Cf_SF   GTTTGCGTGA AGCATTGTGG TGACGTCCTT CATACTAATT GTTTCTCTTC
Dren70_Cf_SF   GTTTGCGTGA AGCATTGTGG TGACGTCCTT CATACTAATT GTTTCTCTTC
Dren72_Cb_SF   GTTTGCGTGA AGCATTGTGG TGACGTCCTT CATACTAATT GTTTCTCTTC
Dren73_Cb_Ch   GTTTGCGTGA AGCATTGTGG TGACGTCCTT CATACTAATT GTTTCTCTTC
Dren74_Cb_Co   GTTTGCGTGA AGCATTGTGG TGACGTCCTT CATACTAATT GTTTCTCTTC
Dren75_Cb_Co   GTTTGCGTGA AGCATTGTGG TGACGTCCTT CATACTAATT GTTTCTCTTC
Dren80_Cf_SF   GTTTGCGTGA AGCATTGTGG TGACGTCCTT CATACTAATT GTTTCTCTTC
Dren81_Cf_SF   GTTTGCGTGA AGCATTGTGG TGACGTCCTT CATACTAATT GTTTCTCTTC
Dren82_Cb_Co   GTTTGCGTGA AGCATTGTGG TGACGTCCTT CATACTAATT GTTTCTCTTC
Dren83_Cb_Ch   GTTTGCGTGA AGCATTGTGG TGACGTCCTT CATACTAATT GTTTCTCTTC
Dren84_Cf_SF   GTTTGCGTGA AGCATTGTGG TGACGTCCTT CATACTAATT GTTTCTCTTC
Dren85_Cb_SF   GTTTGCGTGA AGCATTGTGG TGACGTCCTT CATACTAATT GTTTCTCTTC
Dren86_Cb_Co   GTTTGCGTGA AGCATTGTGG TGACGTCCTT CATACTAATT GTTTCTCTTC
Dren87_Cf_BA   GTTTGCGTGA AGCATTGTGG TAACGTCCTT CATACTAATT GTTTCTCTTC
Dren88_Lg_SF   GTTTGCGTGA AGCATTGTGG TGACGTCCTT CATACTAATT GTTTCTCTTC

               ....|....| ....|....| ....|....| ....|....| ....|....|
                    905        915        925        935        945
Dren02_Cf_BA   CTGTTTTGGC AGGGGGTATC ACAATACTTT TAACTGATCG GA----T---
Dren07_Cf_BA   CTGTTTTAGC AGGGGGTATT ACAATACTTT TAACTGATCG GA----T---
Dren08_Cf_BA   CTGTTTTAGC AGGGGGTATT ACAATACTTT TAACTGATCG GA----T---
Dren09_Cf_BA   CTGTTTTGGC AGGGGGTATC ACAATACTTT TAACTGATCG GA----T---
Dren10_Cf_BA   CTGTTTTAGC AGGGGGTATT ACAATACTTT TAACTGATCG GA----T---
Dren18_Cf_BA   CTGTTTTAGC AGGGGGTATT ACAATACTTT TAACTGATCG GA----T---
Dren19_Cf_BA   CTGTTTTAGC AGGGGGTATT ACAATACTTT TAACTGATCG GA----T---
Dren23_Cf_BA   CTGTTTTGGC AGGGGGTATC ACAATACTTT TAACTGATCG GA----T---
Dren25_Cf_SF   CTGTTTTGGC AGGGGGTATC ACAATACTTT TAACTGATCG GA----T---
Dren32_Cf_SC   CTGTTTTGGC AGGGGGTATC ACAATACTTT TAACTGATCG GA----T---
Dren35_Cf_Pa   CTGTTTTGGC AGGGGGTATC ACAATACTTT TAACTGATCG GA----T---
Dren40_Cf_RG   CTGTTTTAGC AGGGGGTATT ACAATACTTT TAACTGATCG GAATTTTAAC
```

```
Dren41_Cf_RG    CTGTTTTAGC AGGGGGTATT ACAATACTTT TAACTGATCG GA----T---
Dren42_Cf_RG    CTGTTTTAGC AGGGGGTATT ACAATACTTT TAACTGATCG GA----T---
Dren43_Cf_RG    CTGTTTTAGC AGGGGGTATC ACAATACTTT TAACTGATCG GA----T---
Dren45_Cf_RG    CTGTTTTAGC AGGGGGTATC ACAATACTTT TAACTGATCG GA----T---
Dren46_Cf_SF    CTGTTTTGGC AGGGGGTATC ACAATACTTT TAACTGATCG GAATTTTAAC
Dren47_Cb_SF    CTGTTTTAGC AGGGGGTATT ACAATACTTT TAACTGATCG GA----T---
Dren48_Cf_BA    CTGTTTTAGC AGGGGGTATT ACAATACTTT TAACTGATCG GAATTTTAAC
Dren49_Cf_BA    CTGTTTTGGC AGGGGGTATC ACAATACTTT TAACTGATCG GA----T---
Dren50_Cf_Ch    CTGTTTTAGC AGGGGGTATT ACAATACTTT TAACTGATCG GAATTTTAAC
Dren53_Cf_BA    CTGTTTTAGC AGGGGGTATT ACAATACTTT TAACTGATCG GA----T---
Dren58_Cb_SF    CTGTTTTAGC AGGGGGTATT ACAATACTTT TAACTGATCG GA----T---
Dren66_Cf_SF    CTGTTTTAGC AGGGGGTATT ACAATACTTT TAACTGATCG GAATTTTAAC
Dren67_Cf_SF    CTGTTTTAGC AGGGGGTATT ACAATACTTT TAACTGATCG GA--------
Dren68_Cf_SF    CTGTTTTAGC AGGGGGTATT ACAATACTTT TAACTGATCG GAATTTTAAC
Dren69_Cf_SF    CTGTTTTAGC AGGGGGTATT ACAATACTTT TAACTGATCG GA--------
Dren70_Cf_SF    CTGTTTTGGC AGGGGGTATC ACAATACTTT TAACTGATCG GAATTTTAAC
Dren72_Cb_SF    CTGTTTTAGC AGGGGGTATT ACAATACTTT TAACTGATCG GAATTTTAAC
Dren73_Cb_Ch    CTGTTTTAGC AGGGGGTATT ACAATACTTT TAACTGATCG GAATTTTAAC
Dren74_Cb_Co    CTGTTTTGGC AGGGGGTATC ACAATACTTT TAACTGATCG GAATTTTAAC
Dren75_Cb_Co    CTGTTTTAGC AGGGGGTATT ACAATACTTT TAACTGATCG GAATTTTAAC
Dren80_Cf_SF    CTGTTTTGGC AGGGGGTATC ACAATACTTT TAACTGATCG GAATTTTAAC
Dren81_Cf_SF    CTGTTTTGGC AGGGGGTATC ACAATACTTT TAACTGATCG GAATTTTAAC
Dren82_Cb_Co    CTGTTTTGGC AGGGGGTATC ACAATACTTT TAACTGATCG GAATTTTAAC
Dren83_Cb_Ch    CTGTTTTAGC AGGGGGTATT ACAATACTTT TAACTGATCG GAATTTTAAC
Dren84_Cf_SF    CTGTTTTGGC AGGGGGTATC ACAATACTTT TAACTGATCG GAATTTTAAC
Dren85_Cb_SF    CTGTTTTGGC AGGGGGTATC ACAATACTTT TAACTGATCG GAATTTTAAC
Dren86_Cb_Co    CTGTTTTAGC AGGGGGTATT ACAATACTTT TAACTGATCG GAATTTTAAC
Dren87_Cf_BA    CTGTTTTAGC AGGGGGTATT ACAATACTTT TAACTGATCG GAATTTTAAC
Dren88_Lg_SF    CTGTTTTAGC AGGGGGTATT ACAATACTTT TAACTGATCG GAATTTTAAC

                ....|....| ....|....| ....|....| ....|....| ....|....|
                     955        965        975        985        995
Dren02_Cf_BA    ---------- ---------- ---------- ---------- ----------
Dren07_Cf_BA    ---------- ---------- ---------- ---------- ----------
Dren08_Cf_BA    ---------- ---------- ---------- ---------- ----------
Dren09_Cf_BA    ---------- ---------- ---------- ---------- ----------
Dren10_Cf_BA    ---------- ---------- ---------- ---------- ----------
Dren18_Cf_BA    ---------- ---------- ---------- ---------- ----------
Dren19_Cf_BA    ---------- ---------- ---------- ---------- ----------
Dren23_Cf_BA    ---------- ---------- ---------- ---------- ----------
Dren25_Cf_SF    ---------- ---------- ---------- ---------- ----------
Dren32_Cf_SC    ---------- ---------- ---------- ---------- ----------
Dren35_Cf_Pa    ---------- ---------- ---------- ---------- ----------
Dren40_Cf_RG    ACAGCTTTTT TTGAAACTTC GGGAGGAGGT GACCCTGTTC TATTCCAACA
Dren41_Cf_RG    ---------- ---------- ---------- ---------- ----------
Dren42_Cf_RG    ---------- ---------- ---------- ---------- ----------
Dren43_Cf_RG    ---------- ---------- ---------- ---------- ----------
Dren45_Cf_RG    ---------- ---------- ---------- ---------- ----------
Dren46_Cf_SF    ACAGCTTTTT TTGAAACTTC GGGAGGAGGT GACCCTGTTC TATTCCAACA
Dren47_Cb_SF    ---------- ---------- ---------- ---------- ----------
Dren48_Cf_BA    ACANCTTTTT TTGAAACTTC GGGAGGAGG- --T------- ----------
Dren49_Cf_BA    ---------- ---------- ---------- ---------- ----------
Dren50_Cf_Ch    ACAGCTTTTT TTGAAACTTC GGGAGGAGGT GACCCTGTTC TATTCCAACA
Dren53_Cf_BA    ---------- ---------- ---------- ---------- ----------
```

```
Dren58_Cb_SF   ---------- ---------- ---------- ---------- ----------
Dren66_Cf_SF   ACAGCTTTTT TTGAAACTTC GGGAGGAGGT GACCCTGTTC TATTCCAACA
Dren67_Cf_SF   ---------- ---------- ---------- ---------- ----------
Dren68_Cf_SF   ACAGCTTTTT TTGAAACTTC GGGAGGAGGT GACCCTGTTC TATTCCAACA
Dren69_Cf_SF   ---------- ---------- ---------- ---------- ----------
Dren70_Cf_SF   ACAGCTTTTT TTGAAACTTC GGGAGGAGGT GACCCTGTTC TATTCCAACA
Dren72_Cb_SF   ACAGCTTTTT TTGAAACTTC GGGAGGAGGT GACCCTGTTC TATTCCAACA
Dren73_Cb_Ch   ACAGCTTTTT TTGAAACTTC GGGAGGAGGT GACCCTGTTC TATTCCAACA
Dren74_Cb_Co   ACAGCTTTTT TTGAAACTTC GGGAGGAGGT GACCCTGTTC TATTCCAACA
Dren75_Cb_Co   ACAGCTTTTT TTGAAACTTC GGGAGGAGGT GACCCTGTTC TATTCCAACA
Dren80_Cf_SF   ACAGCTTTTT TTGAAACTTC GGGAGGAGGT GACCCTGTTC TATTCCAACA
Dren81_Cf_SF   ACAGCTTTTT TTGAAACTTC GGGAGGAGGT GACCCTGTTC TATTCCAACA
Dren82_Cb_Co   ACAGCTTTTT TTGAAACTTC GGGAGGAGGT GACCCTGTTC TATTCCAACA
Dren83_Cb_Ch   ACAGCTTTTT TTGAAACTTC GGGAGGAGGT GACCCTGTTC TATTCCAACA
Dren84_Cf_SF   ACAGCTTTTT TTGAAACTTC GGGAGGAGGT GACCCTGTTC TATTCCAACA
Dren85_Cb_SF   ACAGCTTTTT TTGAAACTTC GGGAGGAGGT GACCCTGTTC TATTCCAACA
Dren86_Cb_Co   ACAGCTTTTT TTGAAACTTC GGGAGGAGGT GACCCTGTTC TATTCCAACA
Dren87_Cf_BA   ACAGCTTTTT TTGAAACTTC GGGAGGAGGT GACCCTGTTC TATTCCAACA
Dren88_Lg_SF   ACAGCTTTTT TTGAAACTTC GGGAGGAGGT GACCCTGTTC TATTCCAACA

               ....|....| ....|....| ....|....| ....|....| ....|....|
                   1005       1015       1025       1035       1045
Dren02_Cf_BA   ---------- ---------- ---------- ---------- ----------
Dren07_Cf_BA   ---------- ---------- ---------- ---------- ----------
Dren08_Cf_BA   ---------- ---------- ---------- ---------- ----------
Dren09_Cf_BA   ---------- ---------- ---------- ---------- ----------
Dren10_Cf_BA   ---------- ---------- ---------- ---------- ----------
Dren18_Cf_BA   ---------- ---------- ---------- ---------- ----------
Dren19_Cf_BA   ---------- ---------- ---------- ---------- ----------
Dren23_Cf_BA   ---------- ---------- ---------- ---------- ----------
Dren25_Cf_SF   ---------- ---------- ---------- ---------- ----------
Dren32_Cf_SC   ---------- ---------- ---------- ---------- ----------
Dren35_Cf_Pa   ---------- ---------- ---------- ---------- ----------
Dren40_Cf_RG   TATTTTCTGA TTTTTTGGCC ATCCAGAGGT TTATATTTTA GCTTTGCCTG
Dren41_Cf_RG   ---------- ---------- ---------- ---------- ----------
Dren42_Cf_RG   ---------- ---------- ---------- ---------- ----------
Dren43_Cf_RG   ---------- ---------- ---------- ---------- ----------
Dren45_Cf_RG   ---------- ---------- ---------- ---------- ----------
Dren46_Cf_SF   TATTTTCTGA TTTTTTGGCC ATCCAGAGGT TTATATTTTA GCTTTGCCTG
Dren47_Cb_SF   ---------- ---------- ---------- ---------- ----------
Dren48_Cf_BA   ---------- ---------- ---------- ---------- ----------
Dren49_Cf_BA   ---------- ---------- ---------- ---------- ----------
Dren50_Cf_Ch   TATTTTCTGA TTTTTTGGCC ATCCAGAGGT TTATATTTTA GCTTTGCCTG
Dren53_Cf_BA   ---------- ---------- ---------- ---------- ----------
Dren58_Cb_SF   ---------- ---------- ---------- ---------- ----------
Dren66_Cf_SF   TATTTTCTGA TTTTTTGGTC ACCCAGAGGT TTATATTTTA GCTTTGCCTG
Dren67_Cf_SF   ---------- ---------- ---------- ---------- ----------
Dren68_Cf_SF   TATTTTCTGA TTTTTTGGCC ATCCAGAGGT TTATATTTTA GCTTTGCCTG
Dren69_Cf_SF   ---------- ---------- ---------- ---------- ----------
Dren70_Cf_SF   TATTTTCTGA TTTTTTGGCC ATCCAGAGGT TTATATTTTA GCTTTGCCTG
Dren72_Cb_SF   TATTTTCTGA TTTTTTGGCC ATCCAGAGGT TTATATTTTA GCTTTGCCTG
Dren73_Cb_Ch   TATTTTCTGA TTTTTTGGCC ATCCAGAGGT TTATATTTTA GCTTTGCCTG
Dren74_Cb_Co   TATTTTCTGA TTTTTTGGCC ATCCAGAGGT TTATATTTTA GCTTTGCCTG
Dren75_Cb_Co   TATTTTCTGA TTTTTTGGCC ATCCAGAGGT TTATATTTTA GCTTTGCCTG
```

```
Dren80_Cf_SF   TATTTTCTGA TTTTTTGGCC ATCCAGAGGT TTATATTTTA GCTTTGCCTG
Dren81_Cf_SF   TATTTTCTGA TTTTTTGGCC ATCCAGAGGT TTATATTTTA GCTTTGCCTG
Dren82_Cb_Co   TATTTTCTGA TTTTTTGGCC ATCCAGAGGT TTATATTTTA GCTTTGCCTG
Dren83_Cb_Ch   TATTTTCTGA TTTTTTGGCC ATCCAGAGGT TTATATTTTA GCTTTGCCTG
Dren84_Cf_SF   TATTTTCTGA TTTTTTGGCC ATCCAGAGGT TTATATTTTA GCTTTGCCTG
Dren85_Cb_SF   TATTTTCTGA TTTTTTGGCC ATCCAGAGGT TTATATTTTA GCTTTGCCTG
Dren86_Cb_Co   TATTTTCTGA TTTTTTGGCC ATCCAGAGGT TTATATTTTA GCTTTGCCTG
Dren87_Cf_BA   TATTTTCTGA TTTTTTGGCC ATCCAGAGGT TTATATTTTA GCTTTGCCTG
Dren88_Lg_SF   TATTTTCTGA TTTTTTGGCC ATCCAGAGGT TTATATTTTA GCTTTGCCTG

               ....|....| ....|....| ....|....| ....|....| ....|....|
                   1055       1065       1075       1085       1095
Dren02_Cf_BA   ---------- ---------- ---------- -----AGTGG CAAGCCAAAG
Dren07_Cf_BA   ---------- ---------- ---------- -----AGTGG CAAGCCAAAG
Dren08_Cf_BA   ---------- ---------- ---------- -----AGAGG CAAGCCAAAG
Dren09_Cf_BA   ---------- ---------- ---------- -----AGTGG CAAGCCAAAG
Dren10_Cf_BA   ---------- ---------- ---------- -----AGAGG CAAGCCAAAG
Dren18_Cf_BA   ---------- ---------- ---------- -----AGTGG TAAGCCAAAG
Dren19_Cf_BA   ---------- ---------- ---------- -----AGTGG TAAGCCAAAG
Dren23_Cf_BA   ---------- ---------- ---------- -----AGTGG CAAGCCAAAG
Dren25_Cf_SF   ---------- ---------- ---------- -----AGTGG CAAGCCAAAG
Dren32_Cf_SC   ---------- ---------- ---------- -----AGTGG CAAGCCAAAG
Dren35_Cf_Pa   ---------- ---------- ---------- -----AGTGG CAAGCCAAAG
Dren40_Cf_RG   CTTTTGGTGT AATTTCAGAA AGAGTAACAG TTCTTAGTGG CAAGCCAAAG
Dren41_Cf_RG   ---------- ---------- ---------- -----AGTGG CAAGCCAAAG
Dren42_Cf_RG   ---------- ---------- ---------- -----AGTGG CAAGCCAAAG
Dren43_Cf_RG   ---------- ---------- ---------- -----AGCGG TAAGCCAAAG
Dren45_Cf_RG   ---------- ---------- ---------- -----AGCGG TAAGCCAAAG
Dren46_Cf_SF   CTTTTGGTGT AATTTCAGAA AGGGTAACAG TTCTTAGTGG CAAGCCAAAG
Dren47_Cb_SF   ---------- ---------- ---------- -----AGTGG CAAGCCAAAG
Dren48_Cf_BA   ---------- ---------- ---------- -----AGAGG CAAGCCAAAG
Dren49_Cf_BA   ---------- ---------- ---------- -----AGTGG CAAGCCAAAG
Dren50_Cf_Ch   CTTTTGGTGT AATTTCAGAA AGAGTAACAG TTCTTAGAGG CAAGCCAAAG
Dren53_Cf_BA   ---------- ---------- ---------- -----AGAGG CAAGCCAAAG
Dren58_Cb_SF   ---------- ---------- ---------- -----AGTGG CAAGCCAAAG
Dren66_Cf_SF   CTTTTGGTGT AATTTCAGAA AGAGTAACAG TTCTTAGTGG TAAGCCAAAG
Dren67_Cf_SF   ---------- ---------- ---------- ----TAGTGG CAAGCCAAAG
Dren68_Cf_SF   CTTTTGGTGT AATTTCAGAA AGAGTAACAG TTCTTAGTGG CAAGCCAAAG
Dren69_Cf_SF   ---------- ---------- ---------- ----TAGTGG CAAGCCAAAG
Dren70_Cf_SF   CTTTTGGTGT AATTTCAGAA AGAGTAACAG TTCTTAGTGG CAAGCCAAAG
Dren72_Cb_SF   CTTTTGGTGT AATTTCAGAA AGAGTAACAG TTCTTAGAGG CAAGCCAAAG
Dren73_Cb_Ch   CTTTTGGTGT AATTTCAGAA AGAGTAACAG TTCTTAGTGG CAAGCCAAAG
Dren74_Cb_Co   CTTTTGGTGT AATTTCAGAA AGGGTAACAG TTCTTAGTGG CAAGCCAAAG
Dren75_Cb_Co   CTTTTGGTGT AATTTCAGAA AGAGTAACAG TTCTTAGAGG CAAGCCAAAG
Dren80_Cf_SF   CTTTTGGTGT AATTTCAGAA AGGGTAACAG TTCTTAGTGG CAAGCCAAAG
Dren81_Cf_SF   CTTTTGGTGT AATTTCAGAA AGGGTAACAG TTCTTAGTGG CAAGCCAAAG
Dren82_Cb_Co   CTTTTGGTGT AATTTCAGAA AGGGTAACAG TTCTTAGTGG CAAGCCAAAG
Dren83_Cb_Ch   CTTTTGGTGT AATTTCAGAA AGAGTAACAG TTCTTAGAGG CAAGCCAAAG
Dren84_Cf_SF   CTTTTGGTGT AATTTCAGAA AGGGTAACAG TTCTTAGTGG CAAGCCAAAG
Dren85_Cb_SF   CTTTTGGTGT AATTTCAGAA AGAGTAACAG TTCTTAGTGG CAAGCCAAAG
Dren86_Cb_Co   CTTTTGGCGT AATTTCAGAA AGAGTAACAG TTCTTAGTGG CAAGCCAAAG
Dren87_Cf_BA   CTTTTGGTGT AATTTCAGAA AGAGTAACAG TTCTTAGTGG CAAGCCAAAG
Dren88_Lg_SF   CTTTTGGTGT AATTTCAGAA AGAGTAACAG TTCTTAGTGG CAAGCCAAAG
```

```
                 ....|....| ....|....| ....|....| ....|....| ....|....|
                     1105       1115       1125       1135       1145
Dren02_Cf_BA     GTGTTTGGAC CTTTGGGTAT AATTTACGCT ATAATTAGAA TTGGCGTGCT
Dren07_Cf_BA     GTGTTTGGGC CTTTGGGTAT AATCTACGCT ATAATTAGAA TTGGCGTGCT
Dren08_Cf_BA     GTGTTTGGAC CTTTGGGTAT AATCTACGCT ATAATTAGAA TTGGCGTGCT
Dren09_Cf_BA     GTGTTTGGAC CTTTGGGTAT AATCTACGCT ATAATTAGAA TTGGCGTGCT
Dren10_Cf_BA     GTGTTTGGAC CTTTGGGTAT AATCTACGCT ATAATTAGAA TTGGCGTGCT
Dren18_Cf_BA     GTGTTTGGAC CTTTGGGCAT AATCTACGCT ATAATTAGAA TTGGCGTGCT
Dren19_Cf_BA     GTGTTTGGAC CTTTGGGCAT AATCTACGCT ATAATTAGAA TTGGCGTGCT
Dren23_Cf_BA     GTGTTTGGAC CTTTGGGTAT AATCTACGCT ATAATTAGAA TTGGCGTGCT
Dren25_Cf_SF     GTGTTTGGAC CTTTGGGTAT AATCTACGCT ATAATTAGAA TTGGCGTGCT
Dren32_Cf_SC     GTGTTTGGAC CTTTGGGTAT AATCTACGCT ATAATTAGAA TTGGCGTGCT
Dren35_Cf_Pa     GTGTTTGGAC CTTTGGGTAT AATCTACGCT ATAATTAGAA TTGGCGTGCT
Dren40_Cf_RG     GTGTTTGGAC CTTTGGGTAT AATCTACGCT ATAATTAGAA TTGGCGTGCT
Dren41_Cf_RG     GTGTTTGGAC CTTTGGGTAT AATCTACGCT ATAATTAGAA TTGGCGTGCT
Dren42_Cf_RG     GTGTTTGGGC CTTTGGGCAT AATCTACGCT ATAATTAGAA TTGGCGTGCT
Dren43_Cf_RG     GTGTTTGGAC CTTTGGGTAT AATCTACGCT ATAATTAGAA TTGGCGTGCT
Dren45_Cf_RG     GTGTTTGGAC CTTTGGGTAT AATCTACGCT ATAATTAGAA TTGGCGTGCT
Dren46_Cf_SF     GTGTTTGGAC CTTTGGGTAT AATCTACGCT ATAATTAGAA TTGGCGTGCT
Dren47_Cb_SF     GTGTTTGGGC CTTTGGGCAT AATCTACGCT ATAATTAGAA TTGGCGTGCT
Dren48_Cf_BA     GTGTTTGGAC CTTTGGGTAT AATCTACGCT ATAATTAGAA TTGGCGTGCT
Dren49_Cf_BA     GTGTTTGGAC CTTTGGGTAT AATCTACGCT ATAATTAGAA TTGGCGTGCT
Dren50_Cf_Ch     GTGTTTGGAC CTTTGGGTAT AATCTACGCT ATAATTAGAA TTGGCGTGCT
Dren53_Cf_BA     GTGTTTGGAC CTTTGGGTAT AATCTACGCT ATAATTAGAA TTGGCGTGCT
Dren58_Cb_SF     GTGTTTGGAC CTTTGGGTAT AATCTACGCT ATAATTAGAA TTGGCGTGCT
Dren66_Cf_SF     GTGTTTGGAC CTTTGGGCAT AATCTACGCT ATAATTAGAA TTGGCGTGCT
Dren67_Cf_SF     GTGTTTGGGC CTTTGGGCAT AATCTACGCT ATAATTAGAA TTGGCGTGCT
Dren68_Cf_SF     GTGTTTGGGC CTTTGGGCAT AATCTACGCT ATAATTAGAA TTGGCGTGCT
Dren69_Cf_SF     GTGTTTGGGC CTTTGGGCAT AATCTACGCT ATAATTAGAA TTGGCGTGCT
Dren70_Cf_SF     GTGTTTGGAC CTTTGGGTAT AATCTACGCT ATAATTAGAA TTGGCGTGCT
Dren72_Cb_SF     GTGTTTGGAC CTTTGGGTAT AATCTACGCT ATAATTAGAA TTGGCGTGCT
Dren73_Cb_Ch     GTGTTTGGGC CTTTGGGCAT AATCTACGCT ATAATTAGAA TTGGCGTGCT
Dren74_Cb_Co     GTGTTTGGAC CTTTGGGTAT AATCTACGCT ATAATTAGAA TTGGCGTGCT
Dren75_Cb_Co     GTGTTTGGAC CTTTGGGTAT AATCTACGCT ATAATTAGAA TTGGCGTGCT
Dren80_Cf_SF     GTGTTTGGAC CTTTGGGTAT AATCTACGCT ATAATTAGAA TTGGCGTGCT
Dren81_Cf_SF     GTGTTTGGAC CTTTGGGTAT AATCTACGCT ATAATTAGAA TTGGCGTGCT
Dren82_Cb_Co     GTGTTTGGAC CTTTGGGTAT AATCTACGCT ATAATTAGAA TTGGCGTGCT
Dren83_Cb_Ch     GTGTTTGGAC CTTTGGGTAT AATCTATGCT ATAATTAGAA TTGGCGTGCT
Dren84_Cf_SF     GTGTTTGGAC CTTTGGGTAT AATCTACGCT ATAATTAGAA TTGGCGTGCT
Dren85_Cb_SF     GTGTTTGGAC CTTTGGGTAT AATCTACGCT ATAATTAGAA TTGGCGTGCT
Dren86_Cb_Co     GTGTTTGGGC CTTTGGGCAT AATCTACGCT ATAATTAGAA TTGGCGTGCT
Dren87_Cf_BA     GTGTTTGGGC CTTTGGGCAT AATCTACGCT ATAATTAGAA TTGGCGTGCT
Dren88_Lg_SF     GTGTTTGGGC CTTTGGGCAT A-TCTACGCT ATAATTAGAA TTGGCGTGCT

                 ....|....| ....|....| ....|....| ....|....| ....|....|
                     1155       1165       1175       1185       1195
Dren02_Cf_BA     TGGTTGTTTT GTGTGGGTAC ATCACATGTT TACTGTAGGT TTAGATGTTG
Dren07_Cf_BA     TGGTTGTTTT GTGTGGGTAC ATCACATGTT TACTGTAGGT TTAGATGTTG
Dren08_Cf_BA     TGGTTGTTTT GTGTGGGTAC ATCACATGTT TACTGTAGGT TTAGATGTTG
Dren09_Cf_BA     TGGTTGTTTT GTGTGGGTGC ATCACATGTT TACTGTAGGT TTAGATGTTG
Dren10_Cf_BA     TGGTTGTTTT GTGTGGGTAC ATCACATGTT TACTGTAGGT TTAGATGTTG
Dren18_Cf_BA     TGGTTGTTTT GTGTGGGTAC ATCACATGTT TACTGTAGGT TTAGATGTTG
Dren19_Cf_BA     TGGTTGTTTT GTGTGGGTAC ATCACATGTT TACTGTAGGT TTAGATGTTG
Dren23_Cf_BA     TGGTTGTTTT GTGTGGGTGC ATCACATGTT TACTGTAGGT TTAGATGTTG
```

```
Dren25_Cf_SF   TGGTTGTTTT GTGTGGGTAC ATCACATGTT TACTGTAGGT TTAGATGTTG
Dren32_Cf_SC   TGGTTGTTTT GTGTGGGTAC ATCACATGTT TACTGTAGGT TTAGATGTTG
Dren35_Cf_Pa   TGGTTGTTTT GTGTGGGTAC ATCACATGTT TACTGTAGGT TTAGATGTTG
Dren40_Cf_RG   TGGTTGTTTT GTGTGGGTAC ATCACATGTT TACTGTAGGT TTAGATGTTG
Dren41_Cf_RG   TGGTTGTTTT GTGTGGGTAC ATCACATGTT TACTGTAGGT TTAGATGTTG
Dren42_Cf_RG   TGGTTGTTTT GTGTGGGTAC ATCACATGTT TACTGTAGGT TTAGATGTTG
Dren43_Cf_RG   TGGTTGTTTT GTGTGGGTAC ATCACATGTT TACTGTAGGT TTAGATGTTG
Dren45_Cf_RG   TGGTTGTTTT GTGTGGGTAC ATCACATGTT TACTGTAGGT TTAGATGTTG
Dren46_Cf_SF   TGGTTGTTTT GTGTGGGTAC ATCACATGTT TACTGTAGGT TTAGATGTTG
Dren47_Cb_SF   TGGTTGTTTT GTGTGGGTAC ATCACATGTT TACTGTAGGT TTAGATGTTG
Dren48_Cf_BA   TGGTTGTTTT GTGTGGGTAC ATCACATGTT TACTGTAGGT TTAGATGTTG
Dren49_Cf_BA   TGGTTGTTTT GTGTGGGTGC ATCACATGTT TACTGTAGGT TTAGATGTTG
Dren50_Cf_Ch   TGGTTGTTTT GTGTGGGTAC ATCACATGTT TACTGTAGGT TTAGATGTTG
Dren53_Cf_BA   TGGTTGTTTT GTGTGGGTAC ATCACATGTT TACTGTAGGT TTAGATGTTG
Dren58_Cb_SF   TGGTTGTTTT GTGTGGGTAC ATCACATGTT TACTGTAGGT TTAGATGTTG
Dren66_Cf_SF   TGGTTGTTTT GTGTGGGTAC ATCACATGTT TACTGTAGGT TTAGATGTTG
Dren67_Cf_SF   TGGTTGTTTT GTGTGGGTGC ATCACATGTT TACTGTAGGT TTAGATGTTG
Dren68_Cf_SF   TGGTTGTTTT GTGTGGGTGC ATCACATGTT TACTGTAGGT TTAGATGTTG
Dren69_Cf_SF   TGGTTGTTTT GTGTGGGTGC ATCACATGTT TACTGTAGGT TTAGATGTTG
Dren70_Cf_SF   TGGTTGTTTT GTGTGGGTAC ATCACATGTT TACTGTAGGT TTAGATGTTG
Dren72_Cb_SF   TGGTTGTTTT GTGTGGGTAC ATCACATGTT TACTGTAGGT TTAGATGTTG
Dren73_Cb_Ch   TGGTTGTTTT GTGTGGGTAC ATCACATGTT TACTGTAGGT TTAGATGTTG
Dren74_Cb_Co   TGGTTGTTTT GTGTGGGTAC ATCACATGTT TACTGTAGGT TTAGATGTTG
Dren75_Cb_Co   TGGTTGTTTT GTGTGGGTAC ATCACATGTT TACTGTAGGT TTAGATGTTG
Dren80_Cf_SF   TGGTTGTTTT GTGTGGGTAC ATCACATGTT TACTGTAGGT TTAGATGTTG
Dren81_Cf_SF   TGGTTGTTTT GTGTGGGTAC ATCACATGTT TACTGTAGGT TTAGATGTTG
Dren82_Cb_Co   TGGTTGTTTT GTGTGGGTAC ATCACATGTT TACTGTAGGT TTAGATGTTG
Dren83_Cb_Ch   TGGTTGTTTT GTGTGGGTAC ATCACATGTT TACTGTAGGT TTAGATGTTG
Dren84_Cf_SF   TGGTTGTTTT GTGTGGGTAC ATCACATGTT TACTGTAGGT TTAGATGTTG
Dren85_Cb_SF   TGGTTGTTTT GTGTGGGTGC ATCACATGTT TACTGTAGGT TTAGATGTTG
Dren86_Cb_Co   TGGTTGTTTT GTGTGGGTAC ATCACATGTT TACTGTAGGT TTAGATGTTG
Dren87_Cf_BA   TGGTTGTTTT GTGTGGGTAC ATCACATGTT TACTGTAGGT TTAGATGTTG
Dren88_Lg_SF   TGGTTGTTTT GTGTGGGTGC ATCACATGTT TACTGTAGGT TTAGATGTTG

               ....|....| ....|....| ....|....| ....|....| ....|....|
                   1205       1215       1225       1235       1245
Dren02_Cf_BA   ATACGCGTGC GTATTTTGCT GCTGCGAGTA TAATCATCGG TATTCCGACG
Dren07_Cf_BA   ATACGCGTGC GTATTTTGCT GCTGCGAGTA TAATCATCGG TATTCCGACA
Dren08_Cf_BA   ATACGCGTGC GTATTTTGCT GCTGCGAGTA TAATCATCGG TATTCCGACG
Dren09_Cf_BA   ATACGCGTGC GTATTTTGCT GCTGCGAGTA TAATCATCGG TATTCCGACG
Dren10_Cf_BA   ATACGCGTGC GTATTTTGCT GCTGCGAGTA TAATCATCGG TATTCCGACG
Dren18_Cf_BA   ATACGCGTGC GTATTTTGCT GCTGCGAGTA TAATCATCGG TATTCCGACA
Dren19_Cf_BA   ATACGCGTGC GTATTTTGCT GCTGCGAGTA TAATCATCGG TATTCCGACA
Dren23_Cf_BA   ATACGCGTGC GTATTTTGCT GCTGCGAGTA TAATCATCGG TATTCCGACG
Dren25_Cf_SF   ATACGCGTGC GTATTTTGCT GCTGCGAGTA TAATCATCGG TATTCCGACG
Dren32_Cf_SC   ATACGCGTGC GTATTTTGCT GCTGCGAGTA TAATCATCGG TATTCCGACG
Dren35_Cf_Pa   ATACGCGTGC GTATTTTGCT GCTGCGAGTA TAATCATCGG TATTCCGACG
Dren40_Cf_RG   ATACGCGTGC GTATTTTGCT GCTGCGAGTA TAATCATCGG TATCCCGACG
Dren41_Cf_RG   ATACGCGTGC GTATTTTGCT GCTGCGAGTA TAATCATCGG TATCCCGACG
Dren42_Cf_RG   ATACGCGTGC GTATTTTGCT GCTGCGAGTA TAATCATCGG TATTCCGACA
Dren43_Cf_RG   ATACGCGTGC GTATTTTGCT GCTGCGAGTA TAATCATCGG TATTCCGACG
Dren45_Cf_RG   ATACGCGTGC GTATTTTGCT GCTGCGAGTA TAATCATCGG TATTCCGACG
Dren46_Cf_SF   ATACGCGTGC GTATTTTGCT GCTGCGAGTA TAATCATCGG TATTCCGACG
Dren47_Cb_SF   ATACGCGTGC GTATTTTGCT GCTGCGAGTA TAATCATCGG TATTCCGACA
```

```
Dren48_Cf_BA   ATACGCGTGC GTATTTTGCT GCTGCGAGTA TAATCATCGG TATTCCGACG
Dren49_Cf_BA   ATACGCGTGC GTATTTTGCT GCTGCGAGTA TAATCATCGG TATTCCGACG
Dren50_Cf_Ch   ATACGCGTGC GTATTTTGCT GCTGCGAGTA TAATCATCGG TATTCCGACG
Dren53_Cf_BA   ATACGCGTGC GTATTTTGCT GCTGCGAGTA TAATCATCGG TATTCCGACG
Dren58_Cb_SF   ATACGCGTGC GTATTTTGCT GCTGCGAGTA TAATCATCGG TATCCCGACG
Dren66_Cf_SF   ATACGCGTGC GTATTTTGCT GCTGCGAGTA TAATCATCGG TATTCCGACA
Dren67_Cf_SF   ATACGCGTGC GTATTTTGCT GCTGCGAGTA TAATCATCGG TATTCCGACA
Dren68_Cf_SF   ATACGCGTGC GTATTTTGCT GCTGCGAGTA TAATCATCGG TATTCCGACA
Dren69_Cf_SF   ATACGCGTGC GTATTTTGCT GCTGCGAGTA TAATCATCGG TATTCCGACA
Dren70_Cf_SF   ATACGCGTGC GTATTTTGCT GCTGCGAGTA TAATCATCGG TATTCCGACG
Dren72_Cb_SF   ATACGCGTGC GTATTTTGCT GCTGCGAGTA TAATCATCGG TATTCCGACG
Dren73_Cb_Ch   ATACGCGTGC GTATTTTGCT GCTGCGAGTA TAATCATCGG TATTCCGACA
Dren74_Cb_Co   ATACGCGTGC GTATTTTGCT GCTGCGAGTA TAATCATCGG TATTCCGACG
Dren75_Cb_Co   ATACGCGTGC GTATTTTGCT GCTGCGAGTA TAATCATCGG TATTCCGACG
Dren80_Cf_SF   ATACGCGTGC GTATTTTGCT GCTGCGAGTA TAATCATCGG TATTCCGACG
Dren81_Cf_SF   ATACGCGTGC GTATTTTGCT GCTGCGAGTA TAATCATCGG TATTCCGACG
Dren82_Cb_Co   ATACGCGTGC GTATTTTGCT GCTGCGAGTA TAATCATCGG TATTCCGACG
Dren83_Cb_Ch   ATACGCGTGC GTATTTTGCT GCTGCGAGTA TAATCATCGG TATTCCGACG
Dren84_Cf_SF   ATACGCGTGC GTATTTTGCT GCTGCGAGTA TAATCATCGG TATTCCGACG
Dren85_Cb_SF   ATACGCGTGC GTATTTTGCT GCTGCGAGTA TAATCATCGG TATTCCGACG
Dren86_Cb_Co   ATACGCGTGC GTATTTTGCT GCTGCGAGTA TAATCATCGG TATTCCGACA
Dren87_Cf_BA   ATACGCGTGC GTATTTTGCT GCTGCGAGTA TAATCATCGG TATCCCGACA
Dren88_Lg_SF   ATACGCGTGC GTATTTTGCT GCTGCGAGTA TAATCATCGG TATTCCGACA

               ....|....| ....|....| ....|....| ....|....| ....|....|
                   1255       1265       1275       1285       1295
Dren02_Cf_BA   GGGGTAAAAG TATTTAGTTG ATTAGCTACT TTGTATGGTG GTCAGGTTAT
Dren07_Cf_BA   GGGGTAAAAG TATTTAGTTG ATTAGCTACT TTGTATGGTG GTCAGGTTAT
Dren08_Cf_BA   GGGGTAAAAG TATTTAGTTG ATTAGCTACT TTGTATGGTG GTCAGGTTAT
Dren09_Cf_BA   GGGGTAAAAG TATTTAGCTG ATTAGCTACT TTGTATGGTG GTCAGGTTAT
Dren10_Cf_BA   GGGGTAAAAG TATTTAGTTG ATTAGCTACT TTGTATGGTG GTCAGGTTAT
Dren18_Cf_BA   GGGGTAAAAG TATTTAGTTG ATTAGCTACT TTGTATGGTG GTCAGGTTAT
Dren19_Cf_BA   GGGGTAAAAG TATTTAGTTG ATTAGCTACT TTGTATGGTG GTCAGGTTAT
Dren23_Cf_BA   GGGGTAAAAG TATTTAGCTG ATTAGCTACT TTGTATGGTG GTCAGGTTAT
Dren25_Cf_SF   GGGGTAAAAG TATTTAGTTG ATTAGCTACT TTGTATGGTG GTCAGGTTAT
Dren32_Cf_SC   GGGGTAAAAG TATTTAGTTG ATTAGCTACT TTGTATGGTG GTCAGGTTAT
Dren35_Cf_Pa   GGGGTAAAAG TATTTAGTTG ATTAGCTACT TTGTATGGTG GTCAGGTTAT
Dren40_Cf_RG   GGGGTAAAAG TATTTAGTTG ATTAGCTACT TTGTATGGTG GTCAGGTTAT
Dren41_Cf_RG   GGGGTAAAAG TATTTAGTTG ATTAGCTACT TTGTATGGTG GTCAGGTTAT
Dren42_Cf_RG   GGGGTAAAAG TATTTAGTTG ATTAGCTACT TTGTATGGTG GTCAGGTTAT
Dren43_Cf_RG   GGGGTAAAAG TATTTAGTTG ATTAGCTACT TTGTATGGTG GTCAGGTTAT
Dren45_Cf_RG   GGGGTAAAAG TATTTAGTTG ATTAGCTACT TTGTATGGTG GTCAGGTTAT
Dren46_Cf_SF   GGGGTAAAAG TATTTAGTTG ATTAGCTACT TTGTATGGTG GTCAGGTTAT
Dren47_Cb_SF   GGGGTAAAAG TATTTAGTTG ATTAGCTACT TTGTATGGTG GTCAGGTTAT
Dren48_Cf_BA   GGGGTAAAAG TATTTAGTTG ATTAGCTACT TTGTATGGTG GTCAGGTTAT
Dren49_Cf_BA   GGGGTAAAAG TATTTAGCTG ATTAGCTACT TTGTATGGTG GTCAGGTTAT
Dren50_Cf_Ch   GGGGTAAAAG TATTTAGTTG ATTAGCTACT TTGTATGGTG GTCAGGTTAT
Dren53_Cf_BA   GGGGTAAAAG TATTTAGTTG ATTAGCTACT TTGTATGGTG GTCAGGTTAT
Dren58_Cb_SF   GGGGTAAAAG TATTTAGTTG ATTAGCTACT TTGTATGGTG GTCAGGTTAT
Dren66_Cf_SF   GGGGTAAAAG TATTTAGTTG ATTAGCTACT TTGTATGGTG GTCAGGTTAT
Dren67_Cf_SF   GGGGTAAAAG TATTTAGTTG ATTAGCTACT TTGTATGGTG GTCAGGTTAT
Dren68_Cf_SF   GGGGTAAAAG TATTTAGTTG ATTAGCTACT TTGTATGGTG GTCAGGTTAT
Dren69_Cf_SF   GGGGTAAAAG TATTTAGTTG ATTAGCTACT TTGTATGGTG GTCAGGTTAT
Dren70_Cf_SF   GGGGTAAAAG TATTTAGTTG ATTAGCTACT TTGTATGGTG GTCAGGTTAT
```

```
Dren72_Cb_SF   GGGGTAAAAG TATTTAGTTG ATTAGCTACT TTGTATGGTG GTCAGGTTAT
Dren73_Cb_Ch   GGGGTAAAAG TATTTAGTTG ATTAGCTACT TTGTATGGTG GTCAGGTTAT
Dren74_Cb_Co   GGGGTAAAAG TATTTAGTTG ATTAGCTACT TTGTATGGTG GTCAGGTTAT
Dren75_Cb_Co   GGGGTAAAAG TATTTAGTTG ATTAGCTACT TTGTATGGTG GTCAGGTTAT
Dren80_Cf_SF   GGGGTAAAAG TATTTAGTTG ATTAGCTACT TTGTATGGTG GTCAGGTTAT
Dren81_Cf_SF   GGGGTAAAAG TATTTAGTTG ATTAGCTACT TTGTATGGTG GTCAGGTTAT
Dren82_Cb_Co   GGGGTAAAAG TATTTAGTTG ATTAGCTACT TTGTATGGTG GTCAGGTTAT
Dren83_Cb_Ch   GGGGTAAAAG TATTTAGTTG GTTAGCTACT TTGTATGGTG GTCAGGTTAT
Dren84_Cf_SF   GGGGTAAAAG TATTTAGTTG ATTAGCTACT TTGTATGGTG GTCAGGTTAT
Dren85_Cb_SF   GGGGTAAAAG TATTTAGCTG ATTAGCTACT TTGTATGGTG GTCAGGTTAT
Dren86_Cb_Co   GGGGTAAAAG TATTTAGTTG ATTAGCTACT TTGTATGGTG GTCAGGTTAT
Dren87_Cf_BA   GGGGTAAAAG TATTTAGTTG ATTAGCTACT TTGTATGGTG GTCAGGTTAT
Dren88_Lg_SF   GGGGTAAAAG TATTTAGTTG ATTAGCTACT TTGTATGGTG GTCAGGTTAT

               ....|....| ....|....| ....|....| ....|....| ...
                   1305       1315       1325       1335
Dren02_Cf_BA   CTTTTCACCT TTGTATTGTT GGACTGTAGG GTTTATTTCT GTG
Dren07_Cf_BA   CTTTTCACCT TTGTATTGTT GGACTGTAGG GTTTATTTCT GTG
Dren08_Cf_BA   CTTTTCACCT TTGTATTGTT GGACTGTAGG GTTTATTTCT GTG
Dren09_Cf_BA   CTTTTCACCT TTGTATTGTT GGACTGTAGG GTTTATTTCT GTG
Dren10_Cf_BA   CTTTTCACCT TTGTATTGTT GGACTGTAGG GTTTATTTCT GTG
Dren18_Cf_BA   CTTTTCACCT TTGTATTGTT GGACTGTAGG GTTTATTTCT GTG
Dren19_Cf_BA   CTTTTCACCT TTGTATTGTT GGACTGTAGG GTTTATTTCT GTG
Dren23_Cf_BA   CTTTTCACCT TTGTATTGTT GGACTGTAGG GTTTATTTCT GTG
Dren25_Cf_SF   CTTTTCACCT TTGTATTGTT GGACTGTAGG GTTTATTTCT GTG
Dren32_Cf_SC   CTTTTCACCT TTGTATTGTT GGACTGTAGG GTTTATTTCT GTG
Dren35_Cf_Pa   CTTTTCACCT TTGTATTGTT GGACTGTAGG GTTTATTTCT GTG
Dren40_Cf_RG   CTTTTCACCT TTGTATTGTT GGACTGTAGG GTTTATTTCT GTG
Dren41_Cf_RG   CTTTTCACCT TTGTATTGTT GGACTGTAGG GTTTATTTCT GTG
Dren42_Cf_RG   CTTTTCACCT TTGTATTGTT GGACTGTAGG GTTTATTTCT GTG
Dren43_Cf_RG   CTTTTCACCC TTGTATTGTT GGACTGTAGG GTTTATTTCT GTG
Dren45_Cf_RG   CTTTTCACCC TTGTATTGTT GGACTGTAGG GTTTATTTCT GTG
Dren46_Cf_SF   CTTTTCACCT TTGTATTGTT GGACTGTAGG GTTTATTTCT GTG
Dren47_Cb_SF   CTTTTCACCT TTGTATTGTT GGACTGTAGG GTTTATTTCT GTG
Dren48_Cf_BA   CTTTTCACCT TTGTATTGTT GGACTGTAGG GTTTATTTCT GTG
Dren49_Cf_BA   CTTTTCACCT TTGTATTGTT GGACTGTAGG GTTTATTTCT GTG
Dren50_Cf_Ch   CTTTTCACCT TTGTATTGTT GGACTGTAGG GTTTATTTCT GTG
Dren53_Cf_BA   CTTTTCACCT TTGTATTGTT GGACTGTAGG GTTTATTTCT GTG
Dren58_Cb_SF   CTTTTCACCT TTGTATTGTT GGACTGTAGG GTTTATTTCT GTG
Dren66_Cf_SF   CTTTTCACCT TTGTATTGTT GGACTGTAGG GTTTATTTCT GTG
Dren67_Cf_SF   CTTTTCACCT TTGTATTGTT GGACTGTAGG GTTTATTTCT GTG
Dren68_Cf_SF   CTTTTCACCT TTGTATTGTT GGACTGTAGG GTTTATTTCT GTG
Dren69_Cf_SF   CTTTTCACCT TTGTATTGTT GGACTGTAGG GTTTATTTCT GTG
Dren70_Cf_SF   CTTTTCACCT TTGTATTGTT GGACTGTAGG GTTTATTTCT GTG
Dren72_Cb_SF   CTTTTCACCT TTGTATTGTT GGACTGTAGG GTTTATTTCT GTG
Dren73_Cb_Ch   CTTTTCACCT TTGTATTGTT GGACTGTAGG GTTTATTTCT GTG
Dren74_Cb_Co   CTTTTCACCT TTGTATTGTT GGACTGTAGG GTTTATTTCT GTG
Dren75_Cb_Co   CTTTTCACCT TTGTATTGTT GGACTGTAGG GTTTATTTCT GTG
Dren80_Cf_SF   CTTTTCACCT TTGTATTGTT GGACTGTAGG GTTTATTTCT GTG
Dren81_Cf_SF   CTTTTCACCT TTGTATTGTT GGACTGTAGG GTTTATTTCT GTG
Dren82_Cb_Co   CTTTTCACCT TTGTATTGTT GGACTGTAGG GTTTATTTCT GTG
Dren83_Cb_Ch   CTTTTCACCT TTGTATTGTT GGACTGTAGG GTTTATTTCT GTG
Dren84_Cf_SF   CTTTTCACCT TTGTATTGTT GGACTGTAGG GTTTATTTCT GTG
Dren85_Cb_SF   CTTTTCACCT TTGTATTGTT GGACTGTAGG GTTTATTTCT GTG
```

```
Dren86_Cb_Co   CTTTTCACCT TTGTATTGTT GGACTGTAGG GTTTATTTCT GTG
Dren87_Cf_BA   CTTTTCACCT TTGTATTGTT GGACTGTAGG GTTTATTTCT GTG
Dren88_Lg_SF   CTTTTCACCT TTGTATTGTT GGACTGTAGG GTTTATTTCT GTG
```
